# Iron Corrole‐Catalyzed Intramolecular Amination Reactions of Alkyl Azides. Spectroscopic Characterization and Reactivity of [Fe^V^(Cor)(NAd)]

**DOI:** 10.1002/advs.202401420

**Published:** 2024-08-20

**Authors:** Tingjie You, Ka‐Pan Shing, Liangliang Wu, Kai Wu, Hua‐Hua Wang, Yungen Liu, Lili Du, Runhui Liang, David Lee Phillips, Xiao‐Yong Chang, Jie‐Sheng Huang, Chi‐Ming Che

**Affiliations:** ^1^ State Key Laboratory of Synthetic Chemistry Department of Chemistry The University of Hong Kong Pokfulam Road Hong Kong 000000 P. R. China; ^2^ Department of Chemistry Southern University of Science and Technology Shenzhen Guangdong 518055 P. R. China; ^3^ HKU Shenzhen Institute of Research and Innovation Shenzhen Guangdong 518057 P. R. China; ^4^ Laboratory for Synthetic Chemistry and Chemical Biology Limited Units 1503–1511, 15/F., Building 17 W, Hong Kong Science Park, New Territories Hong Kong 000000 P. R. China

**Keywords:** C─H activation, corrole, imido complexes, iron, N‐heterocycles

## Abstract

As nitrogen analogues of iron‐oxo species, high‐valent iron‐imido species have attracted great interest in the past decades. Fe^V^‐alkylimido species are generally considered to be key reaction intermediates in Fe(III)‐catalyzed C(sp^3^)─H bond aminations of alkyl azides but remain underexplored. Here, it is reported that iron‐corrole (Cor) complexes can catalyze a wide range of intramolecular C─H amination reactions of alkyl azides to afford a variety of 5‐, 6‐ and 7‐membered N‐heterocycles, including alkaloids and natural product derivatives, with up to 3880 turnover numbers (TONs) and excellent diastereoselectivity (>99:1 d.r.). Mechanistic studies including density functional theory (DFT) calculations and intermolecular hydrogen atom abstraction (HAA) reactions reveal key reactive Fe^V^‐alkylimido intermediates. The [Fe^V^(Cor)(NAd)] (Ad = adamantyl) complex is independently prepared and characterized through electron paramagnetic resonance (EPR), resonance Raman (rR) measurement, and X‐ray photoelectron spectroscopy (XPS). This complex is reactive toward HAA reactions with kinetic isotope effects (KIEs) similar to [Fe(Cor)]‐catalyzed intramolecular C─H amination of alkyl azides.

## Graphical abstract

Iron‐corrole‐catalyzed C–H aminations of alkyl azides can be used to prepare a variety of N‐heterocyclic compounds, possibly via high‐valent Fe^V^‐alkylimido species. [Fe^V^(T*p*‐OMePC)(NAd)] is prepared and characterized by various spectroscopic methods, and its HAA reactivity toward C(sp^3^)–H bonds is studied.

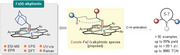

## 1 Introduction

**Figure 1:**
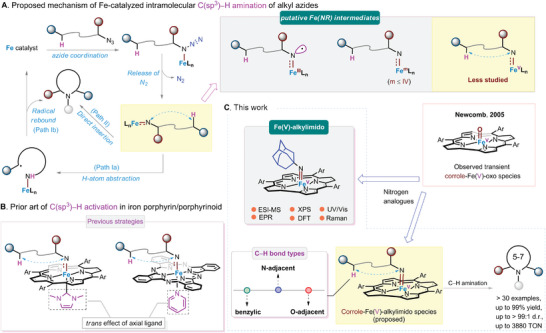
A) Proposed mechanism of Fe‐catalyzed intramolecular C(sp^3^)─H amination of alkyl azides. B) Prior art of C(sp^3^)─H activation in iron porphyrin/phthalocyanine systems. C) Catalytic intramolecular C(sp^3^)─H amination via proposed Fe(V)‐alkylimido corrole intermediates and spectroscopic characterization of [Fe^V^(Cor)(NAd)] complex.

Intramolecular C‒H amination via metal‐imido intermediates has attracted increasing interest as a method for the synthesis of cyclic compounds via C‒N bond formation.^[^ ^1^ ^]^ Iron‐catalyzed intramolecular C(sp^3^)─H amination of alkyl azides (N_3_R)^[^ ^2^, ^3^ ^]^ via iron‐alkylimido/alkylimidyl (Fe(NR), R = alkyl) intermediates (**Figure** **1**A) is an appealing strategy to access N‐heterocycles, which are ubiquitous in pharmaceuticals and natural products.^[^ ^4^ ^]^ Given the crucial role of high‐valent iron‐oxo (Fe(O)) species, including Fe^V^(O) species, in enzymatic and/or synthetic alkane oxidation reactions,^[^ ^5^ ^]^ and the isoelectronic properties of oxo and imido ligands, high‐valent Fe(NR) species are considered to be highly reactive toward C─H functionalization reactions. Previously, Fe^IV/V^(NAr) species^[^ ^6^ ^]^ were considered to be key intermediates in cyclization reactions catalyzed by cytochrome P‐450 enzymes.^[^ ^3^, ^6^, ^7^ ^]^ In addition, Fe^III^(NR)^[^ ^8^ ^]^ or Fe^IV^(NR)^[^ ^9^ ^]^ species as well as Fe^V^(NSO_2_Tol)^[^ ^10^ ^]^ species^[^ ^11^ ^]^ capable of activating C(sp^3^)─H bonds have been reported in the literature. Identified Fe^V^(NR) complexes are rare and include bisimido species [Fe(3,5‐*i*Pr_2_Ar*)(NAd)_2_]^[^ ^12^ ^]^ and monoimido species [Fe(TIMMN^Mes^)(NR)]^3+^ (R = Ad, Et; TIMMN = *N*‐anchored tris‐N‐heterocyclic carbene chelating neutral ligand).^[^ ^13^ ^]^ Fe^V^(NR) species capable of reacting with C(sp^3^)─H bonds or serving as active intermediates for the catalytic functionalization of C(sp^3^)─H bonds remain unexplored.

We recently exploited the *trans* effect of the axial ligand (L) to improve the catalytic efficiency of Fe(macrocyclic N_4_ ligand) catalyzed C(sp^3^)─H amination, possibly through [Fe(Por)(NR)(L)] (Por = porphyrinato dianion, L = N‐heterocyclic carbene)^[^ ^3f^ ^]^ or [Fe(Pc)(NR)(L)] (Pc = phthalocyaninato dianion, L = pyridine)^[^ ^3i^ ^]^ intermediates (Figure 1B). These Fe(NR) species supported by dianionic Por or Pc ligands have not been directly detected, unlike spectroscopically characterized [Fe^IV^(TBP_8_Cz^+•^)(NSO_2_Tol)] complex supported by related trianionic corrolazine ligand.^[^ ^14^ ^]^ To facilitate the formation/identification of reactive Fe^V^(NR) species, we turned our attention to the trianionic corrole (Cor) ligand, a porphyrin ligand analog with one meso carbon removed that stabilizes high‐valent species, such as the M^V^ (M = Cr, Mn) complex.^[^ ^15^ ^]^ Newcomb and co‐workers previously observed transient [Fe^V^(Cor)(O)] species by ultraviolet‐visible (UV/Vis) spectroscopy.^[^ ^16^ ^]^ We envision that [Fe^V^(Cor)(NR)] species can also be generated and be able to react with inert C(sp^3^)─H bonds. The synthesis, spectroscopic characterization, and reactivity studies of reactive [Fe^V^(Cor)(NR)] species are important and provide useful information for elucidating the structure‐reactivity relationships in Fe(III) catalyzed C─H aminations as well as other types of nitrene transfer reactions.

Here, we report a robust Fe(Cor) complex [Fe(T*p*‐OMePC)Cl]^[^ ^17^ ^]^ (**1**), which can catalyze the intramolecular C(sp^3^)─H (including benzylic, O‐ and N‐adjacent) amination reactions of various alkyl azides, affording a variety of 5‐, 6‐ and 7‐ membered N‐heterocycles with up to 3880 TONs and excellent diastereoselectivity. [Fe^III^(T*p*‐OMePC)] (**2**, in situ generated by reduction of complex **1**) showed similar performance in catalyzing the above‐mentioned intramolecular C(sp^3^)─H amination reactions. DFT calculations support Fe^V^‐alkylimido species as key reaction intermediates in Fe(Cor)‐catalyzed intramolecular C(sp^3^)─H aminations of alkyl azides. We prepared a corrole‐supported Fe^V^‐alkylimido species [Fe^V^(T*p*‐OMePC)(NAd)] reactive toward C(sp^3^)─H bonds (Figure 1C) and characterized it through various spectroscopic studies including UV/Vis, high‐resolution electrospray‐ionization mass spectrometry (HR‐ESI‐MS), EPR, XPS, and rR spectroscopy measurements.

## 2 Results and Discussion

### 2.1 Intramolecular C(sp^3^)─H Amination Catalyzed by Iron Corroles

The iron corrole complexes [Fe(Cor)Cl] (Cor = TPC,^[^ ^18^ ^]^ F_15_TPC,^[^ ^19^ ^]^ TDCPC^[^ ^20^ ^]^), [Fe(TPC)]_2_O^[^ ^18^, ^19^ ^]^ and [Fe(T*p*‐OMePC)Cl] (**1**)^[^ ^17^ ^]^ were prepared and characterized according to the literature. Complex **1** was also characterized by X‐ray crystal structure determination (see Supporting Information).^[^ ^21^ ^]^

We set out to explore Fe(Cor)‐catalyzed intramolecular C(sp^3^)─H amination reactions using (4‐azidobutyl)benzene (**3**) as a model substrate (**Table** **1**). Toluene was the best solvent compared to chlorobenzene (PhCl), dimethylformamide, dimethyl sulfoxide, and dioxane (Table S1, Supporting Information). Refluxing the toluene solution of the reaction mixture under argon for 3 h in the presence of [Fe(TPC)Cl] as catalyst and Boc_2_O as additive gave the desired amination product **4** in 68% yield (entry 1). Changing the axial ligand of the catalyst (from Cl^−^ to µ‐oxo) did not improve the results (entry 2). Tuning the electronic effect of the corrole ligand showed that changing the Ph groups of TPC to the more strongly electron‐withdrawing C_6_F_5_ or 2,6‐Cl_2_C_6_H_3_ group reduced the yield of **4** from 68% to 41% and 58%, respectively (entries 3 and 4). Interestingly, the catalyst [Fe(T*p*‐OMePC)Cl] (**1**) containing a corrole ligand with a stronger electron‐donating *p*‐OMeC_6_H_4_ group afforded the desired product **4** in 85% yield (entry 5). By reducing the catalyst loading of complex **1** to 0.05 mol%, the TON (1880) obtained in 24 h was 3 times higher than the TON (620) previously reported by van der Vlugt group^[^ ^3d^ ^]^ (turnover frequency: 78.3 h^−1^ vs 3.7 h^−1^). We also used **1** to generate in situ [Fe(T*p*‐OMePC)] (**2**); the in situ prepared complex **2** catalyzed the amination reaction with comparable product yield (70%, entry 7). Lowering the reaction temperature to 100 °C also ensured complete conversion of **3** despite a longer reaction time (entry 8). No conversion of **3** was detected using iron porphyrin catalyst at this temperature (100 °C) or using catalyst **1** at <80 °C.^[^ ^3f^ ^]^ Control experiments indicate that Boc_2_O is required for catalytic amination, as this compound reacts with the coordinated amine to form/release the corresponding bulky Boc‐protected counterpart. Substrate **3** alone did not decompose when heated under conditions similar to the Fe(Por)‐catalyzed reaction (Table S1, Supporting Information).

**Table 1:** Optimization of the reaction conditions^a)^

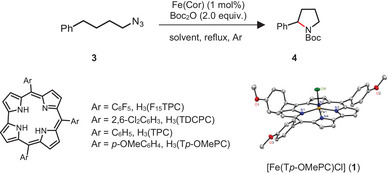					
Entry	Catalyst	Solvent	Time [h]	Conv. [%]	Yield [%]
1	[Fe(TPC)Cl]	toluene	3	>99	68
2	[Fe(TPC)]_2_O	toluene	3	>99	52
3	[Fe(F_15_TPC)Cl]	toluene	3	77	41
4	[Fe(TDCPC)Cl]	toluene	3	>99	58
5	[Fe(T*p*‐OMePC)Cl]	toluene	3	>99	85 [94]^b)^
6	[Fe(T*p*‐OMePC)Cl] (**1**)	PhCl	3	>99	75
7^c)^	[Fe(T*p*‐OMePC)] (**2**)	toluene	3	>99	70
8^d)^	[Fe(T*p*‐OMePC)Cl]	toluene	16	>99	80

^a)^ Reaction conditions: Azide **3** (0.2 mmol), Fe(Cor) (1 mol%), and Boc_2_O (0.4 mmol) in solvent (2.0 mL), reflux under argon;

^b)^ 0.05 mol% **1** was used for 24 h to achieve a TON of 1880;

^c)^ [Fe(T*p*‐OMePC)] (**2**) was in situ generated by reacting **1** with 5.0 equiv. Zn for 5 h before adding **3** and Boc_2_O;

^d)^ at 100 °C.

Various alkyl azide substrates were studied under the optimized conditions (alkyl azide (0.2 mmol), Boc_2_O (2 equiv.), toluene, reflux), using **1** (1 mol%) as catalyst (**Table** **2**). Substrates bearing Ph or substituted Ph groups undergo benzylic C(sp^3^)─H amination to give the corresponding 5‐membered‐ring pyrrolidines (**4**–**10**) in yields as high as 95%. Substituents with different electronic properties and patterns on the Ph ring are well‐accommodated, and electron‐donating *para*‐substituents have higher product yields than electron‐withdrawing substituents. In addition to 5‐membered‐ring products **4**–**10**, 7‐membered‐ring azepine analogues were also obtained by extending the reaction to substrates bearing biphenyl units, such as **11** in 50% yield. Substrates with substituted heteroaromatic groups were also tolerated, providing the desired indole and furan products (**12** and **13**) in 74–80% yields. Notably, this catalytic system was also suitable for the preparation of 5/6‐fused bicyclic pyrrolidines (a core skeleton from Martinellic acid), and product **14** was obtained in 81% yield. This method to generate **14** via metal‐catalyzed intramolecular C(sp^3^)─H amination has not been previously reported. Oxazolidines, imidazolidines, and imidazolidin‐4‐ones are important heterocyclic compounds in natural products and medicines.^[^ ^22^ ^]^ These types of compounds (**15**–**18**) can be obtained in 69–97% yields using substrates containing heteroatoms (N/O) adjacent to the benzylic amination site. As for product **18**, to our delight, this system reaches 3880 TONs, showing comparable efficiency to the Albrecht's catalyst, which has been reported to have excellent performance (up to several thousand product TONs) in the intramolecular C(sp^3^)─H amination of alkyl azides.^[^ ^3j^ ^]^ In order to diversify the substitution patterns on the 5‐membered‐ring products, a series of di‐substituted pyrrolidines (**19**–**24**) were prepared in 95–99% yields. No other products resulting from primary, secondary, and/or tertiary C─H bond amination were detected. The efficiency and practicality of catalyst **1** was also demonstrated through the gram scale synthesis of **19** at 0.2 mol% catalyst loading. We also explored the amination of N/O‐adjacent non‐benzylic C(sp^3^)─H bonds using substrates bearing amide or alkyl amine or alkyl ether groups, affording the corresponding products **25**–**27** (67–85% yield). In order to explore the application in the synthesis of structurally complex bioactive compounds and pharmaceuticals, derivatives of ibuprofen, estrone, nopol, citronellal, and geraniol were used as substrates to obtain the corresponding C─H amination products **28**–**32** (72–97% yield). The Fe(Cor)/N_3_R amination protocol can be used for the rapid synthesis of alkaloid‐related natural product derivatives. For example, Vesicare derivative **33**, Cryptostyline II derivative **34**, and Tadalafil's enantiomer precursor **35** were synthesized by this method from their precursor azides by forming a 6‐membered ring, with yields of 66–90%. Notably, the natural product derivative **34** can also be prepared with 1230 TON; the temperature can be reduced to 80 °C for both substrates (**15**, **32**) while maintaining efficiency. Interestingly, product **36** was aminated at the C7′─H site to form a 7‐membered ring, which was different from the previously reported 6‐membered ring aminated at the corresponding C6─H site,^[^ ^23^ ^]^ although the yield was lower (14%). The structure of **36** was determined by X‐ray crystallography.^[^ ^21^ ^]^

**Table 2:** Substrate scope.^a)^

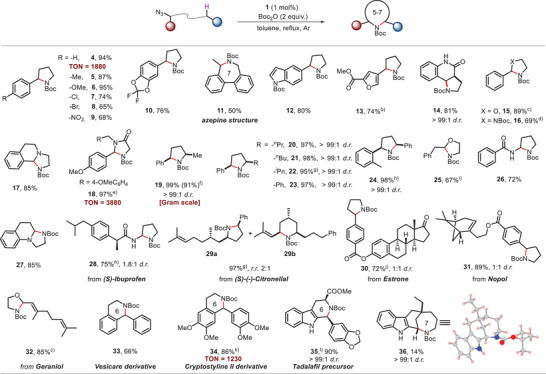

^a)^ Unless otherwise noted, reactions were conducted with azide (0.2 mmol), **1** (1 mol%), and Boc_2_O (0.4 mmol) in toluene (2.0 mL) under argon atmosphere for 2‒5 h. Isolated yields; diastereomeric ratios (*d.r*.: *syn*/*anti*) were determined by GC‐MS and NMR;

^b)^ reaction time = 10 h;

^c)^ at 80 °C for 48 h;

^d)^ 3 equiv. Boc_2_O was added;

^e)^ using 0.025 mol% **1** for 24 h;

^f)^ 1.2 g scale reaction using **1** (0.2 mol%) for 48 h;

^g)^ 5 mol% **1** for 24 h;

^h)^ reaction time = 24 h;

^i)^ at 100 °C for 16 h;

^j)^ using 3 mol% **1** for 8 h;

^k)^ using 0.07 mol% **1** for 44 h;

^l)^ 5 mol% **1** was used.

#### 2.1.1 Mechanistic Studies

We propose that [Fe^IV^(T*p*‐OMePC)Cl] (**1**) catalyzes the intramolecular C‒H amination reaction involving the Fe^III^‐Fe^V^ cycle and the Fe^V^‐imido intermediate capable of undergoing HAA with C(sp^3^)─H bonds. This is reminiscent of Ru^IV^(Por)‐catalyzed C─H oxidation or amination reactions involving the Ru^III^‐Ru^V^ cycle and the reactive Ru^V^‐oxo or ‐imido intermediates.^[^ ^24^ ^]^ One supporting evidence is that after reduction of **1** with Zn, the in situ generated Fe^III^(Cor) species **2** was found to catalyze the amination of **3** to **4** in yields comparable to those using catalyst **1** (entry 7, Table 1). Moreover, analysis of the reaction mixture containing catalyst **1**, alkyl azide **3**, and Boc_2_O in toluene at 100 °C by UV/Vis absorption spectroscopy showed the formation of Fe^III^(Cor) species within 0.5 h with λ_max_ (410, 570 nm) similar to that reported for related Fe^III^(Cor) species.^[^ ^25^ ^]^ The only Fe corrole species detected at and after reaction times of 2 h was the Fe^III^(Cor) species (Figure S3, Supporting Information). This is consistent with an Fe^III^‐Fe^V^ cycle and a reactive Fe^V^‐imido intermediate. Furthermore, an induction period was observed in the time‐course plot of the **1**‐catalyzed amination reaction of **3** at 100 °C (Figure S4, Supporting Information), which was ascribable to the reduction of **1** to Fe^III^(Cor) species prior to the Fe^III^‐Fe^V^ cycle. In contrast to **1**, no induction time was observed for the C─H amination reaction of **3** catalyzed by **2** under the same reaction conditions (Figure S4, Supporting Information). It was observed from the cyclic voltammogram that the reduction potential of the Fe(IV) corrole complex **1** is −0.05 V (vs SCE in CH_2_Cl_2_) (Figure S5, Supporting Information), and hence its reduction should be relatively easy. According to UV/Vis absorption spectroscopy, the reaction of complex **1** with **3** or 4‐phenylbutan‐1‐amine (a by‐product derived from **3**) produces Fe^III^(Cor) species (Figure S6, Supporting Information), revealing a possible reduction pathway. Control experiments excluded the possibility of reduction of complex **1** by organic solvent impurities.

**Scheme 1:**
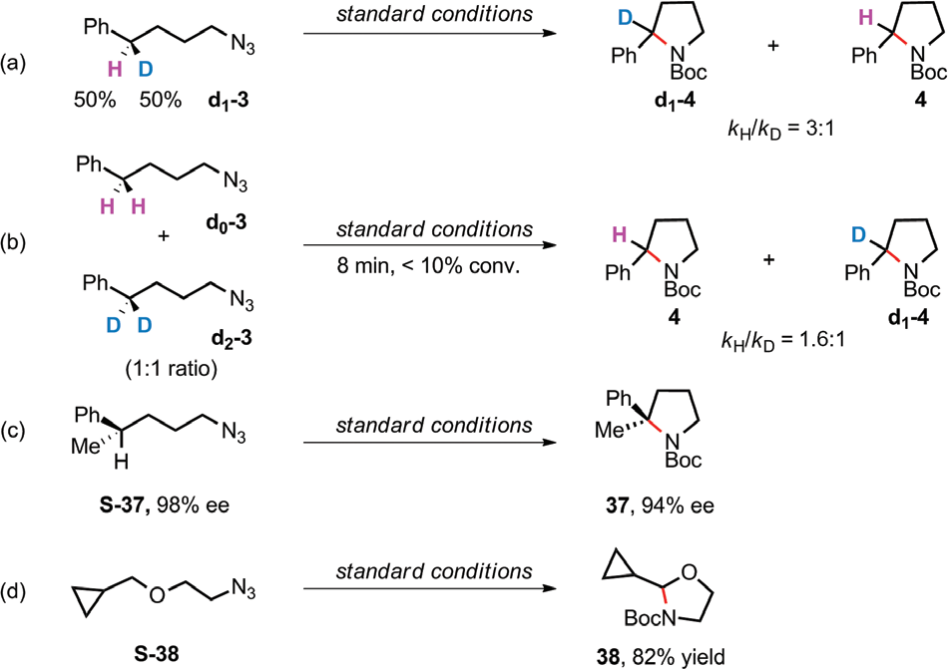
a) Intramolecular KIE; b) intermolecular KIE; c) stereochemical course of the amination of **S‐37** catalyzed by **1**; d) radical clock reaction.

We measured the KIE of intramolecular C(sp^3^)‒H amination of monodeuterated (4‐azidobutyl)benzene substrate (**d_1_‐3**) in the presence of catalyst **1** under standard conditions (**Scheme** **1**a). Analysis of the product by ^1^H nuclear magnetic resonance (NMR) spectroscopy revealed the formation of amination products **d_1_‐4** and **4**, the ratio of which gave a *k*_H_/*k*_D_ value of 3:1 (KIE = 1.6 for intermolecular competitive reaction, Scheme 1b). This value is comparable to that (*k*_H_/*k*_D_ = 3.4:1) reported for other iron‐catalyzed intramolecular C─H amination of alkyl azides.^[^ ^3a,d,f,i^ ^]^ In addition, using (*S*)‐(5‐azidopentan‐2‐yl)benzene (**S‐37**) as the substrate, the corresponding amination product (**37**) with high stereo‐retention level was obtained, and the enantiometric excess (ee) value was slightly reduced (98% to 94%, Scheme 1c). This could be the result of a fast radical rebound after the HAA step. Furthermore, the retention of the cyclopropyl unit in the radical clock experiment (Scheme 1d) also suggests that the radical intermediate following HAA is short‐lived [recombination rate >10^11^ s^–1^] in a stepwise mechanism, although a concerted‐like mechanism cannot be excluded.

**Figure 2:**
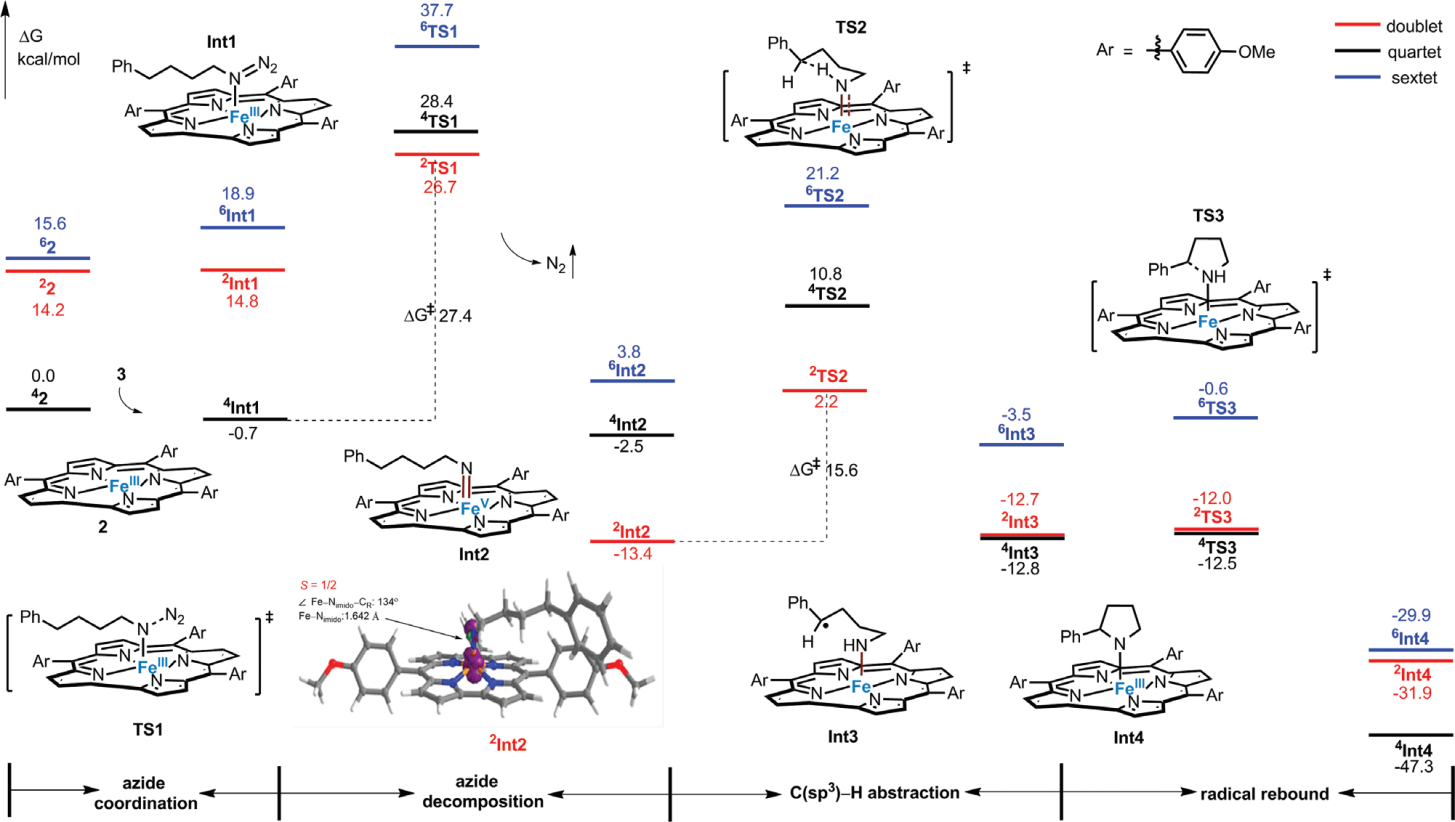
Energy profiles for the intramolecular C(sp^3^)─H amination of alkyl azide **3** catalyzed by **2**.

The mechanism by which alkyl azide N_3_(CH_2_)_4_Ph (**3**) reacts with in situ generated Fe^III^ complex **2** to form the C(sp^3^)─H amination product **4** was studied through DFT calculations (**Figure** **2**). The calculated energy profile is that the initial coordination of **3** to **2** forms the Fe^III^‐azido intermediate [Fe(T*p*‐OMePC)(N_3_(CH_2_)_4_Ph)] (**Int1**), whose quartet state (**^4^Int1**) is 15.5 kcal mol^−1^ lower than the doublet state (**^2^Int1**). UV/Vis spectroscopic titration also supports the formation of a Fe^III^‐azido intermediate upon treatment of **2** with an excess **3** in benzene (binding constant *K*_st_ = 4.62 × 10^2^ M^−1^, at 298 K) (Figure S9, Supporting Information). **^4^Int1** is transformed into the quartet (**^4^Int2**) and doublet (**^2^Int2**) states of Fe^V^‐imido intermediate [Fe(T*p*‐OMePC)(N(CH_2_)_4_Ph)] (**Int2**) via the transition states **^4^TS1** and **^2^TS1**, respectively. The activation energy barriers overcome are 29.1 and 27.4 kcal/mol, respectively, and the formation of **^2^Int2** is more exergonic (10.9 kcal/mol lower energy) than the formation of **^4^Int2**. **^2^Int2** can undergo HAA with an activation barrier of 15.6 kcal/mol (**^2^TS2**) to generate the radical intermediate **Int3** (**^2^Int3** and **^4^Int3** have similar free energies of ΔG = −12.7 and −12.8 kcal/mol, respectively), and then a fast radical rebound process (barrier <1 kcal/mol) occurs to give the ring‐closure intermediate **Int4** (via transition state **TS3**). This is consistent with the high level of stereo‐retention observed in the aforementioned experiments. **^4^Int4** is thermodynamically more stable compared to **^2^Int4**; thus, the formation of the former is more favorable. The DFT‐optimized structure of Fe^V^(N(CH_2_)_4_Ph) intermediate **^2^Int2** has a Fe−N_imido_ distance of 1.642 Å and a Fe−N_imido_‐(CH_2_)_4_Ph angle of 134°. The spin density plot of **^2^Int2** shows that 77% unpaired spins are on Fe, 9% on N_imido_, 11% on Cor and 3% on (CH_2_)_4_Ph (Figure S12, Supporting Information).

### 2.2 Generation, Characterization, and Reactivity of Fe^V^‐alkylimido Species

**Figure 3:**
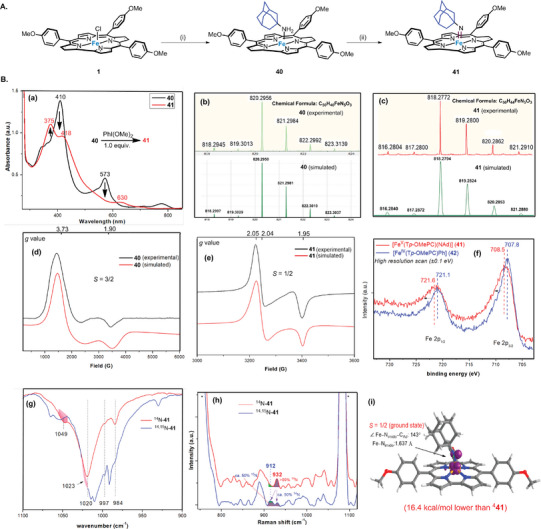
A). Synthesis of Fe^V^‐alkylimido complex **41**. (i) AdNH_2_ (1 equiv.), KC_8_ (1 equiv.), C_6_H_6_, room temperature (RT). (ii) PhI(OMe)_2_ (1 equiv.), C_6_H_6_, RT. B). Spectroscopic characterization of Fe^V^‐alkylimido species. a) UV/Vis spectra of **40** and **41** in C_6_H_6_ under argon. b,c) HR‐ESI‐MS spectra of **40** and **41** (experimental and simulated isotope patterns). d,e) X‐band EPR spectra of **40** and **41** recorded at 100 K (frozen C_6_H_6_; sealed under argon). f) High resolution Fe 2p XP spectra of **41** and [Fe^IV^(*p*‐OMePC)Ph]. g) FTIR spectra (KBr round plates) of ^14^N‐**41** and ^14^N‐**41** +^15^N‐**41** (1:1 ratio). h) rR spectra of ^14^N‐**41** and ^14^N‐**41** +^15^N‐**41** (1:1 ratio) in CH_2_Cl_2_. i) DFT calculations on the structural information of ^**2**^ **41** (*S* = 1/2).

We studied the stoichiometric reaction of in situ generated complex **2** with alkyl azide **3** (1 equiv.) in benzene at 100 °C to afford cyclic imine (**39**) in 70% yield (Figure S10A‐(i), Supporting Information). We did not obtain/detect Fe^V^‐alkylimido species from this reaction, possibly because this Fe^V^ species is unstable at the relatively high temperature at which alkyl azide decomposes. An attempt was then made to generate Fe^V^(NAd) species by decomposing AdN_3_ without β‐H in the presence of **2** (>10^−2^ M in C_6_H_6_) under light irradiation, but the reaction mixture showed incomplete conversion of **2** (Figure S10B‐(h), Supporting Information). With reference to the previous work on the oxidation and deprotonation of Ru^II^(NH_2_Ad) to synthesize Ru^VI^(Ad) species^[^ ^26^ ^]^ and the preparation of [Fe^III^(TPC)(L)] (L = THF, MeCN, Py) from [Fe(TPC)Cl] with KC_8_,^[^ ^27^ ^]^ treatment of **1** with KC_8_/AdNH_2_ in benzene produced [Fe^III^(T*p‐*OMePC)(NH_2_Ad)] (**40**), and 50% ^15^N‐enriched **40** was similarly generated using Ad^15^NH_2_ (**Figure** **3**A‐(i)). Complex **40** can be oxidized by PhI(OMe)_2_ to give [Fe^V^(T*p‐*OMePC)(NAd)] (**41**) (Figure 3A‐(ii)). To our knowledge, Fe^V^(NR) species supported by porphyrin/porphyrinoid ligands have not been previously reported.

The UV/Vis spectrum of **41** in benzene shows bands at λ_max_ = 375, 418, 630 nm, which are different from the bands of **40** (λ_max_ = 410, 573 nm) (Figure 3B‐a). HR‐ESI‐MS analysis of **41** revealed a cluster peak at *m*/*z* 818.2772, consistent with the calculated *m*/*z* value of its formulation [Fe^V^(T*p*‐OMePC)(NAd)] (818.2794, <3 ppm error, Figure 3B‐c). After changing the sample to 50% ^15^N‐enriched **41** (prepared from 50% ^15^N‐enriched **40**), a shift of the cluster peak (*m*/*z* from 818 to 819) was observed. The magnetic susceptibility was measured by Evans method. The effective magnetic moment (*µ*_eff_) values of **41** and **40** are 1.87 µ_B_ and 3.87 µ_B_, resulting from 1 and 3 unpaired electrons, respectively. Consistent with these results, the X‐band EPR spectrum of **40** in C_6_H_6_ at 100 K (Figure 3B‐d) shows a signal at *g* = 3.73, typical of Fe^III^ species with *S* = 3/2.^[^ ^20^ ^]^ Complex **41**, on the other hand, shows a signal with *g* = 2.05, 2.04, 1.95, which can be assigned to Fe^V^ species with *S* = ½ (Figure 3B‐e). The EPR signal *g*_iso_ of **41** is 2.01, which is smaller than low‐spin Fe^III^ and ligand‐centered radical species (typically *g*_iso_ ≥ 2.10),^[^ ^10^, ^20^, ^28^ ^]^ and close to Fe^V^‐imido species (*g*_iso_ ≤ 2.00).^[^ ^10^, ^13^ ^]^ We also measured high‐resolution (down to 0.1 eV) X‐ray photoelectron (XP) spectra of **41** and related Fe^IV^(Cor) species (Figure 3B‐f). The spectrum of **41** shows that Fe 2p_3/2_ and 2p_1/2_ binding energies are 0.7 and 0.5 eV larger than [Fe^IV^(T*p*‐OMePC)Ph] (**42**, prepared by the literature method^[^ ^29^ ^]^), respectively. This change in binding energy is attributed to a change in the oxidation state of the Fe species,^[^ ^28^, ^30^ ^]^ supporting the formulation of **41** as a Fe^V^ species.

Compared with **40**, complex **41** exhibits two new bands at 1049, 984 cm^−1^ in the fourier transform infrared spectroscopy (FTIR) spectrum. The new band at 1049 cm^−1^ can be assigned to *ν*(Fe = NAd), and it was found to disappear after the sample **41** was left standing after 16 h (Figure 3B‐g). By changing **41** to ^15^N‐enriched form, an isotopic shift 26 cm^−1^ is observed, which is close to the prediction of Hooke's law (28 cm^−1^). Likewise, in the rR measurements, the isotopic shift between *ν*(Fe = ^14^NAd) (932 cm^−1^) and *ν*(Fe = ^15^NAd) (912 cm^−1^) is 20 cm^−1^ (Figure 3B‐h), which is consistent with Hooke's law (25 cm^−1^) and DFT calculations (20 cm^−1^; calculated *ν*(Fe = ^14^NAd) and *ν*(Fe = ^15^NAd): 941 and 921 cm^−1^, respectively).

**Figure 4:**
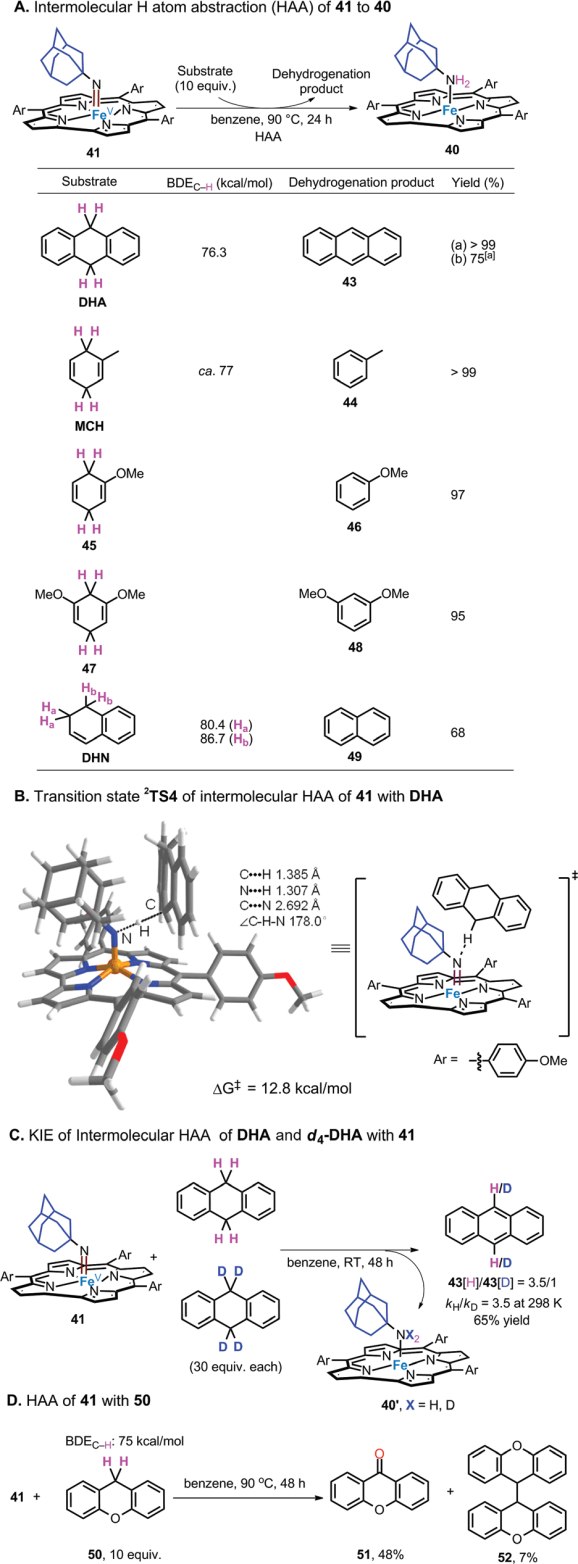
Mechanistic experiments: A) Intermolecular HAA of **41** to give **40**. B) Transition state of intermolecular HAA of **41** with DHA. C) Intermolecular KIE. D) HAA of **41** with **50**. [a] At RT for 48 h.

Complex **41** was found to react with 9,10‐dihydroanthracene (DHA; 10 equiv.) via HAA to form anthracene in >99% yield, along with the formation of **40** (**Figure** **4**A). This HAA reactivity is reminiscent of findings for other Fe‐imido complexes^[^ ^8^, ^11^ ^]^ and [Mn^V^(Cor)(NSO_2_Tol)].^[^ ^31^ ^]^ The HAA reactions of **41** with 1‐methyl‐1,4‐cyclohexadiene (MCH), **45**, **47**, and 1,2‐dihydronaphthalene (DHN) were also studied and the corresponding dehydrogenation products (68–99% yields) were obtained (Figure 4A). ^**2**^ **TS4** is the transition state of the intermolecular HAA between **41** and DHA, its C−N distance is calculated to be 2.692 Å, and the C−H−N angle is 178° (Figure 4B). Intermolecular KIE experiments using **41**, DHA, and *d_4_*‐DHA resulted in a *k*_H_/*k*_D_ value of 3.5:1 (Figure 4C). In addition, the HAA reactivity of **41** was also studied using substrate **50**, which cannot be converted into aromatized products. ^1^H NMR analyses showed that the main organic products originate from radical capture (by trace amount of dioxygen) and radical homo‐coupling reactions (Figure 4D). We also examined the reactivity of **41** toward alkene aziridination reactions. Treatment with styrene (10 equiv.) in benzene at 90 °C for 24 h produced no aziridination products (for details, see the Supporting Information).^[^ ^32^ ^]^ This is different from Betley's Fe‐imidyl species, which reacts with styrene via a radical addition reaction.^[^ ^33^ ^]^ We calculated the structure of [Fe^V^(T*p*‐OMePC)(NAd)] (**41**) through DFT calculations, showing that its doublet state (**^2^41**)  is 16.4 kcal/mol lower in energy than the quartet state (**^4^41**) (Figure 3B‐i). This is similar to the case of the Fe^V^(N(CH_2_)_4_Ph) intermediate **Int2** wherein **^2^Int2** is energetically favored over **^4^Int2**. The Fe−N_imido_ distance and Fe−N_imido_‐Ad angle in the DFT‐optimized structure of **^2^41** are 1.637 Å and 143°, respectively. Its spin density distribution is 73% on Fe, 15% on N_imido_, 8% on Cor, and 4% on Ad (Figure S12, Supporting Information). These values are also comparable to those of **^2^Int2** mentioned above. The molecular orbital of **^2^41** was also calculated (Figure S13, Supporting Information), which shows that this species adopts an electronic configuration of (d_xy_)^2^(d_xz_, d_yz_),^1^ where d_xz_ and d_yz_ have different energies (Δ = 1.1 eV). The short Fe−N_imido_ distance of Fe(NR) complex **41** indicates the covalency of the Fe−N_imido_ bond and the formal oxidation state of the iron center is +5. The similarity between the key structural features of **^2^Int2** and **^2^41**, together with calculated energy profiles (Figure 2), provides support for the participation of Fe^V^‐alkylimido species in Fe(Cor)‐catalyzed alkyl azide intramolecular C(sp^3^)─H amination reaction.

## 3 Conclusion

Fe(Cor) complex [Fe(T*p*‐OMePC)Cl] (**1**) can catalyze a variety of intramolecular C(sp^3^)─H amination reactions of alkyl azides to generate various types of N‐heterocyclic compounds. These catalytic reactions may proceed through high‐valent Fe^V^‐alkylimido intermediates. The complex [Fe^V^(T*p*‐OMePC)(NAd)] was prepared and characterized by spectroscopic methods including UV/Vis, HR‐ESI‐MS, EPR, XPS, rR, and FTIR. The HAA reactivity of Fe^V^(T*p*‐OMePC)(NAd)] toward C(sp^3^)─H bonds was studied. Based on DFT calculations, KIE and stereo‐retention experiments, Fe(Cor)‐catalyzed intramolecular C(sp^3^)─H amination likely proceeds via a HAA step followed by fast radical rebound.

## 4 Experimental Section

### General Procedure for Catalytic C─H Amination

An oven‐dried Schlenk tube was charged with alkyl azide (0.2 mmol, 1.0 equiv.), Boc_2_O (0.4 mmol, 2.0 equiv.), Fe(Cor) catalyst (1 mol%), and dry toluene (2.0 mL) under argon. The mixture was refluxed until full completion was detected by thin‐layer chromatography (usually completed within 2–5 h). The reaction mixture was cooled to room temperature and concentrated, and products were purified via silica chromatography using a mixture of hexane and EtOAc as eluent.

## Conflict of Interest

The authors declare no conflict of interest.

## Supporting information

Supporting Information

## Data Availability

The data that support the findings of this study are available from the corresponding author upon reasonable request.
