# Supplementary material for: Iron Corrole‐Catalyzed Intramolecular Amination Reactions of Alkyl Azides. Spectroscopic Characterization and Reactivity of [FeV(Cor)(NAd)]
Source: Adv Sci (Weinh). 2024 Aug 20;11(39):2401420. doi: 10.1002/advs.202401420 (PMC11497103; doi:10.1002/advs.202401420)
Supplement: Supplementary file 1 — Supporting Information [file ADVS-11-2401420-s001.pdf]

ADVANCED SCIENCE
Open Access

Supporting Information

for *Adv. Sci.*, DOI 10.1002/advs.202401420

Iron Corrole-Catalyzed Intramolecular Amination Reactions of Alkyl Azides. Spectroscopic Characterization and Reactivity of [Fe^{V}(Cor)(NAd)]

*Tingjie You, Ka-Pan Shing, Liangliang Wu, Kai Wu, Hua-Hua Wang, Yungen Liu, Lili Du, Runhui Liang, David Lee Phillips, Xiao-Yong Chang, Jie-Sheng Huang and Chi-Ming Che**

Supporting Information

Iron Corrole-Catalyzed Intramolecular Amination Reactions of Alkyl Azides. Spectroscopic Characterization and Reactivity of [Fe^{V}(Cor)(NAd)]

Tingjie You, Ka-Pan Shing, Liangliang Wu, Kai Wu, Hua-Hua Wang, Yungen Liu, Lili Du, Runhui Liang, David Lee Phillips, Xiao-Yong Chang, Jie-Sheng Huang, and Chi-Ming Che*

Table of Contents

1. Abbreviations ... 2
2. General information ... 2
   2.1 Chemicals, solvents, and synthesis ... 2
   2.2 Instruments ... 3
3. Synthesis of substrates ... 3
   3.1 General procedure A for the synthesis of alkyl azides ... 4
4. Screening of reaction conditions ... 9
5. General procedure for catalytic C–H amination ... 10
6. Characterization data of products ... 11
7. Mechanistic study ... 21
   7.1 Kinetic isotope effect (KIE) ... 24
   7.2 Analysis of stereochemistry ... 25
   7.3 Binding constant ... 26
   7.4 Synthesis and characterization of Fe^{V}-alkylimido complex **41** ... 28
   7.5 Reactivity studies of Fe^{V}-imido complex **41** ... 31
   7.6 KIE of intermolecular HAA of **41** with DHA and d_4-DHA ... 32
   7.7 HAA of **41** with **50** ... 33
   7.8 Stoichiometric or catalytic approach towards intermolecular aziridination ... 33
8. Single crystal X-ray crystallography ... 35
9. Computational details ... 38
10. References ... 59
11. NMR Spectra ... 61

1. Abbreviations

$AdNH_2$: 1-adamantylamine
Boc_2O: di-*tert*-butyl decarbonate
Cor: corrolato (3-)
DCM: dichloromethane
DHA: 9,10-dihydroanthracene
DHN: 1,2-dihydronaphthalene
DIPEA: *N*, *N*-diisopropylethylamine
EDCI: 1-ethyl-3-(3-dimethylaminopropyl)carbodiimide hydrochloride
ESI-MS: electrospray ionization-mass spectrometry
EI-MS: electron impact-mass spectrometry
HAA: hydrogen atom abstraction
KIE: kinetic isotope effect
KC_8: potassium graphite
LAH: lithium aluminium hydride
MCH: 1-methylcyclohexa-1,4-diene
MsCl: methanesulfonyl chloride
TLC: thin-layer chromatography
TPC: 5,10,15-triphenylcorrolato (3-)
T*p*-OMePC: 5,10,15-tris(4-methoxyphenyl)corrolato (3-)
TDCPC: 5,10,15-tris (2,6-dichlorophenyl)corrolato (3-)
F_{15}TPC: 5,10,15-tris(pentafluorophenyl)corrolato (3-)
GC-MS: gas chromatography-mass spectrometry
XPS: X-ray photoelectron spectroscopy
d.r.: diastereomeric ratio
r.r.: regioisomeric ratio
TsCl: *p*-toluenesulfonyl chloride
TON: turnover number
PhIO: iodosylbenzene
rt: room temperature

2. General information

2.1 Chemicals, solvents, and synthesis

All catalytic reactions were performed using the standard Schlenk technique under an argon (Ar) atmosphere. Manipulations of metal complexes synthesis were carried out in the absence of water and dioxygen using standard Schlenk techniques, or in an inert atmosphere glovebox under a dinitrogen atmosphere. Reagents obtained commercially were used without further purification unless indicated otherwise. Anhydrous toluene and DCM were freshly distilled with Na/benzophenone and CaH_2, respectively. Anhydrous benzene was distilled over bluish sodium ketyl for over 2 days and stored over 4 Å molecular sieves prior to use. Anhydrous THF, hexane,

DMF, dioxane, DMSO and MeOH were purchased from J&K and were used directly. C_6D_6 was purchased from J&K and was degassed and stored over 4Å molecular sieves prior to use. [Fe(TPC)Cl],[1] [Fe(T*p*-OMePC)Cl] (**1**),[2] [Fe(TDCPC)Cl],[3] [Fe(F_{15}TPC)Cl],[4] $[Fe(TPC)]_2O$,[1, 4] PhIO[5] and 9,10-dihydroanthracene-d_4 (d_4-DHA)[6] were prepared according to literature procedures. All reactions were monitored by TLC, ^{1}H NMR or UV/Vis spectroscopy. Flash column chromatography was carried out using 300–400 mesh silica gel. Deprotection of the pyrrolidine products **19-24** for 2D NOESY studies was achieved following a previously reported literature procedure.[7] Diastereomeric ratios were determined by GC–MS and ^{1}H NMR.

2.2 Instruments

NMR: ^{1}H and ^{13}C NMR spectra were recorded using a Bruker DPX-600 (600 MHz for ^{1}H and 150 MHz for ^{13}C), Bruker DPX-500 (500 MHz for ^{1}H and 125 MHz for ^{13}C), DPX-400 (400 MHz for ^{1}H and 100 MHz for ^{13}C) spectrometer. Chemical shifts (δ ppm) were determined with tetramethylsilane (TMS) as internal reference or referenced to nondeuterated solvent residual signal (7.26 ppm for ^{1}H in $CDCl_3$, 2.50 ppm for DMSO-d_6, 7.15 ppm for ^{1}H in C_6D_6) and coupling constants (J) were reported in Hertz (Hz). The following abbreviations were used to explain the multiplicities: s = singlet, d = doublet, t = triplet, q = quartet, m = multiplet, br = broad. In the case of products **14**, **20**, **22** and **24**, 2D NOESY spectra were recorded using a Bruker DPX-600 spectrometer to confirm the relative configuration. **MS:** GC-MS analysis was performed by Agilent Technologies 7890B GC system with 5977A MS detector (carrier gas: helium). High-resolution ESI-MS measurements were performed on a Bruker impact II high-resolution LC-QTOF mass spectrometer whereas MALDI-TOF experiment was done using the "ultrafleXtreme™" (Bruker). High-resolution EI mass spectra were recorded on a Thermo Scientific DFS high resolution magnetic sector MS. **UV/Vis:** UV/Vis spectra were recorded on a Cary 8454 UV/Vis spectrophotometer (Agilent Technologies). If necessary, UV/Vis sample could be injected into a UV/Vis cell with side arm connectable to Schlenk line. **EA:** Elemental analysis was performed by the Institute of Chemistry, the Chinese Academy of Sciences. **IR:** Attenuated total-reflectance Fourier-transform infrared (ATR-FTIR) spectroscopy was conducted using the "Spectrum Two™" spectrometer (manufactured by PerkinElmer; Detector type: $LiTaO_3$). FTIR samples were prepared using pairs of round KBr plates (25 x 4 mm) and FTIR spectra were recorded by IRAffinity-1 spectrometer (SHIMADZU). **EPR:** X-band EPR spectra were acquired using a Bruker EMXplus-10/12 instrument (microwave frequency: 9.30 GHz; power: 2-20 mW; power attenuation: 10-20 dB; ModAmp: 3-4 G; ModFreq: 100.00 kHz).

3. Synthesis of substrates

CAUTION: Organic azides are potentially explosive and should be handled with care. While we did not encounter any problems during handling these compounds, proper precautions should be taken during the whole process. Once isolated, these azides were stored in a -20 ºC freezer.

Alkyl azides **3**,[8] **d_1-3**,[8] **d_2-3**,[9] **S-5**,[10] **S-6**,[10] **S-7**,[10] **S-8**,[11] **S-9**,[11] **S-10**,[11] **S-11**,[11] **S-12**,[11] **S-13**,[11] **S-15**,[8] **S-16**,[9] **S-17**,[12] **S-18**,[13] **S-19**,[11] **S-23**,[14] **S-26**,[15] **S-27**,[12], **S-33**,[11] **S-34**,[11] **S-35**,[11] **S-36**,[16] and **S-37**[11] have been previously reported. The synthetic procedures and characterizations for the new azides are described below.

3.1 General procedure A for the synthesis of alkyl azides

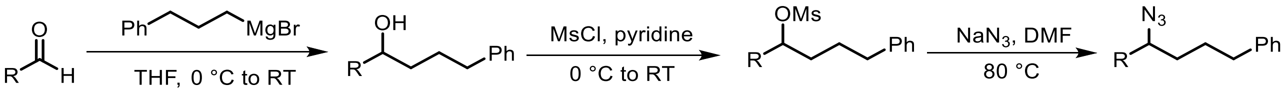

Step 1: (3-Phenylpropyl)magnesium bromide (1.5 equiv.) was added dropwise to aldehyde (1 equiv.) in anhydrous THF (0.25 M) at 0 °C under argon atmosphere, and the reaction mixture was warmed to rt and stirred for 1–3 h. After that, the reaction was quenched with saturated aqueous ammonium chloride, extracted with EtOAc, washed with H_2O, dried with anhydrous $MgSO_4$ and concentrated. The residue was purified by silica gel chromatography to provide the desired secondary alcohol.

Step 2: To a stirring solution of the obtained alcohol (1 equiv.) in anhydrous DCM (0.25 M) at 0 °C, pyridine (3.5 equiv.) and MsCl (3 equiv.) were added. Then the reaction mixture was allowed to warm up to rt and stirred for overnight. The mixture was quenched with 1 M K_2CO_3 once completion. The aqueous phase was extracted three times with DCM. The combined organic phase was washed with saturated aqueous $NaHCO_3$ and brine, dried over Na_2SO_4, filtered, and concentrated *in vacuo*. The residue was purified by silica column chromatography to give the corresponding mesylate.

Step 3: To a stirring solution of mesylate (1 equiv.) in DMF (0.5 M) was added NaN_3 (1.5 equiv.), and the reaction was heated at 80 °C overnight. After completion of the reaction, water was added, and the mixture was extracted with Et_2O three times. The combined organic phase was washed with water and brine, respectively, and dried over Na_2SO_4. After removal of the solvent under reduced pressure, the residue was purified by silica column chromatography to give the desired azide.

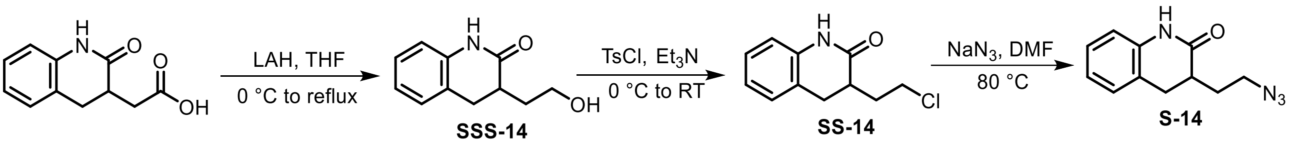

3-(2-Hydroxyethyl)-3,4-dihydroquinolin-2(1*H*)-one (SSS-14):[17] To a solution of 2-(2-oxo-1,2,3,4-tetrahydroquinolin-3-yl)acetic acid[18] (615 mg, 3 mmol, 1 equiv.) in THF (12 mL) was added LAH (225 mg, 6 mmol, 2 equiv.) portionwise at 0 °C under argon atmosphere. Then the solution was refluxed for overnight. After that, a solution of NaOH (10% in water) was added carefully until a white solid precipitated. After filtration over $MgSO_4$ and evaporation of the solvent, the residue was purified by silica column chromatography to obtain the desired product as a white solid (333 mg, 58%). ^{1}H NMR (400 MHz, $CDCl_3$) δ 8.13 (s, 1H), 7.22 – 7.15 (m, 2H), 7.01 (td, J = 7.5, 1.1 Hz, 1H), 6.77 (d, J = 7.9 Hz, 1H), 3.89 – 3.80 (m, 1H), 3.79 – 3.71 (m, 1H), 3.16 (t, J = 5.3 Hz, 1H), 3.03 (dd, J = 14.7, 5.1 Hz, 1H), 2.89 – 2.73 (m, 2H), 2.12 – 2.00 (m, 1H), 1.86 – 1.76 (m, 1H). ^{13}C NMR (125 MHz, $CDCl_3$) δ 174.5, 136.5, 128.2, 127.7, 123.4, 123.4, 115.1, 61.1, 38.6, 33.0, 31.9.

3-(2-Chloroethyl)-3,4-dihydroquinolin-2(1*H*)-one (SS-14): Synthesized following the step 2 in general procedure A while using TsCl (1.5 equiv.) and Et_3N (2 equiv.) instead of MsCl and pyridine and obtained as a white solid (74% yield). ^{1}H NMR (500 MHz, $CDCl_3$) δ 8.18 (s, 1H), 7.22 – 7.16 (m, 2H), 7.01 (td, J = 7.5, 1.2 Hz, 1H), 6.78 (d, J = 7.8 Hz, 1H), 3.81 (dt, J = 11.0, 6.6 Hz, 1H), 3.73 (dt, J = 11.0, 6.6 Hz, 1H), 3.03 (dd, J = 14.4, 4.8 Hz, 1H), 2.88 – 2.75 (m, 2H), 2.43 (dq, J = 13.5, 6.7 Hz, 1H), 1.94 – 1.85 (m, 1H). ^{13}C NMR (125 MHz, $CDCl_3$) δ 172.8, 136.7, 128.2, 127.8, 123.2,

123.1, 115.1, 43.0, 37.2, 32.9, 31.0. HRMS (ESI) m/z: $[M + H]^+$ calcd. for $[C_{11}H_{13}ClNO]^+$: 210.0680, found 210.0679.

3-(2-Azidoethyl)-3,4-dihydroquinolin-2(1*H*)-one (S-14): Synthesized following the step 3 in general procedure A while using the above obtained chloride instead of mesylate, and obtained as a colorless oil (89% yield). ^{1}H NMR (500 MHz, $CDCl_3$) δ 8.56 (s, 1H), 7.22 – 7.15 (m, 2H), 7.00 (td, *J* = 7.5, 0.9 Hz, 1H), 6.80 (d, *J* = 7.8 Hz, 1H), 3.53 (td, *J* = 6.9, 1.6 Hz, 2H), 3.04 (dd, *J* = 15.4, 5.7 Hz, 1H), 2.79 (dd, *J* = 15.4, 10.7 Hz, 1H), 2.74 – 2.66 (m, 1H), 2.25 – 2.15 (m, 1H), 1.78 – 1.69 (m, 1H). ^{13}C NMR (125 MHz, $CDCl_3$) δ 173.0, 136.7, 128.2, 127.8, 123.2, 123.0, 115.2, 49.5, 37.4, 31.2, 29.2. HRMS (ESI) m/z: $[M + H]^+$ calcd. for $[C_{11}H_{13}N_4O]^+$: 217.1084, found 217.1083.

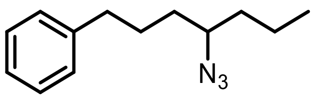

(4-Azidoheptyl)benzene (S-20): Synthesized following the general procedure A and obtained as a colorless oil (46% yield over 3 steps). ^{1}H NMR (400 MHz, $CDCl_3$) δ 7.34 – 7.26 (m, 2H), 7.25 – 7.15 (m, 3H), 3.36 – 3.21 (m, 1H), 2.64 (t, *J* = 7.6 Hz, 2H), 1.86 – 1.75 (m, 1H), 1.74 – 1.64 (m, 1H), 1.59 – 1.31 (m, 6H), 0.92 (t, *J* = 7.1 Hz, 3H). ^{13}C NMR (100 MHz, $CDCl_3$) δ 142.0, 128.4, 125.9, 62.8, 36.6, 35.7, 34.0, 27.9, 19.4, 13.9. HRMS (ESI) m/z: $[M – N_2 + H]^+$ calcd. for $[C_{13}H_{20}N]^+$: 190.1590, found 190.1591.

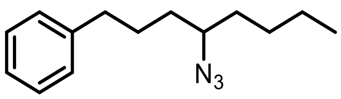

(4-Azidooctyl)benzene (S-21): Synthesized following the general procedure A and obtained as a colorless oil (48% yield over 3 steps). ^{1}H NMR (500 MHz, $CDCl_3$) δ 7.33 – 7.26 (m, 2H), 7.23 – 7.15 (m, 3H), 3.25 (p, *J* = 6.6 Hz, 1H), 2.64 (t, *J* = 7.7 Hz, 2H), 1.85 – 1.74 (m, 1H), 1.73 – 1.63 (m, 1H), 1.55 – 1.45 (m, 4H), 1.44 – 1.26 (m, 4H), 0.91 (t, *J* = 6.4 Hz, 3H). ^{13}C NMR (125 MHz, $CDCl_3$) δ 141.9, 128.3, 125.8, 63.0, 35.6, 34.1, 34.0, 28.2, 27.9, 22.5, 13.9. HRMS (ESI) m/z: $[M – N_2 + H]^+$ calcd. for $[C_{14}H_{22}N]^+$: 204.1747, found 204.1746.

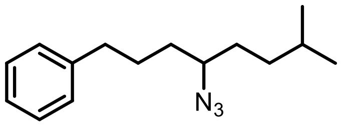

(4-Azido-7-methyloctyl)benzene (S-22): Synthesized following the general procedure A and obtained as a colorless oil (64% yield over 3 steps). ^{1}H NMR (500 MHz, $CDCl_3$) δ 7.30 (t, *J* = 7.6 Hz, 2H), 7.24 – 7.17 (m, 3H), 3.24 (p, *J* = 6.6 Hz, 1H), 2.65 (t, *J* = 7.0 Hz, 2H), 1.87 – 1.76 (m, 1H), 1.75 – 1.65 (m, 1H), 1.61 – 1.48 (m, 5H), 1.38 – 1.18 (m, 2H), 0.90 (dd, *J* = 6.6, 3.2 Hz, 6H). ^{13}C NMR (125 MHz, $CDCl_3$) δ 142.1, 128.5, 126.0, 63.4, 35.8, 35.3, 34.1, 32.4, 28.1, 28.0, 22.8, 22.6. HRMS (ESI) m/z: $[M – N_2 + H]^+$ calcd. for $[C_{15}H_{24}N]^+$: 218.1903, found 218.1902.

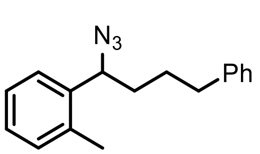

1-(1-Azido-4-phenylbutyl)-2-methylbenzene (S-24): Synthesized following the general procedure

A and obtained as a colorless oil (17% yield over 3 steps). ^{1}H NMR (500 MHz, $CDCl_3$) δ 7.35 – 7.27 (m, 3H), 7.27 – 7.14 (m, 6H), 4.71 – 4.68 (m, 1H), 2.66 (t, *J* = 7.1 Hz, 2H), 2.35 (s, 3H), 1.92 – 1.65 (m, 4H). ^{13}C NMR (125 MHz, $CDCl_3$) δ 141.7, 137.7, 135.4, 130.7, 128.4, 127.9, 126.5, 126.2, 125.9, 62.3, 35.5, 34.8, 28.1, 19.3. HRMS (ESI) m/z: $[M – N_2 + H]^+$ calcd. for $[C_{17}H_{20}N]^+$: 238.1590, found 238.1589.

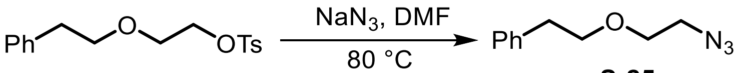

S-25

(2-(2-Azidoethoxy)ethyl)benzene (S-25): Synthesized following the step 3 in general procedure A from 2-phenethoxyethyl 4-methylbenzenesulfonate[19] and obtained as a colorless oil (81% yield). ^{1}H NMR (400 MHz, $CDCl_3$) δ 7.29 (t, *J* = 7.4 Hz, 2H), 7.26 – 7.18 (m, 3H), 3.70 (t, *J* = 7.1 Hz, 2H), 3.62 (t, *J* = 4.9 Hz, 2H), 3.36 (t, *J* = 4.8 Hz, 2H), 2.91 (t, *J* = 7.1 Hz, 2H). ^{13}C NMR (100 MHz, $CDCl_3$) δ 138.7, 128.9, 128.4, 126.3, 72.3, 69.6, 50.8, 36.4. HRMS (EI) m/z: $[M – N_2]^+$ calcd. for $[C_{10}H_{13}NO]^+$: 163.0997, found 163.0995.

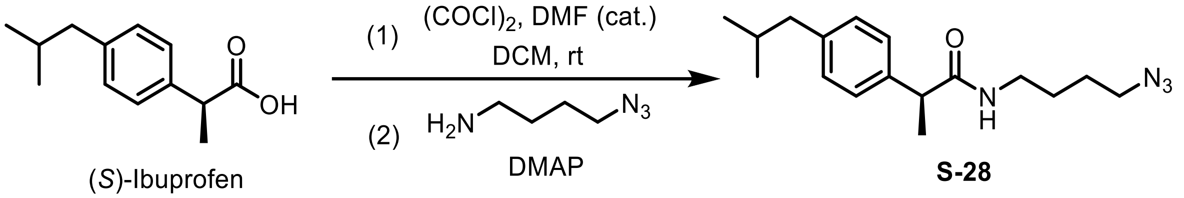

(*S*)-Ibuprofen **S-28**

To a mixture of (*S*)-Ibuprofen (618.8 mg, 3.0 mmol, 1.0 equiv.) in anhydrous DCM (15 mL), DMF (2 drops) and $(COCl)_2$ (716.5 mg, 6 mmol, 2.0 equiv.) were added dropwise at 0 °C. After the addition was completed, the mixture was stirred at rt for 2 h. Then the solution was concentrated *in vacuo* to remove DCM and excess $(COCl)_2$. After drying under high vacuum for 0.5 h, the residue was dissolved in DCM (10 mL), and *N*,*N*-dimethylpyridin-4-amine (550 mg, 4.5 mmol, 1.5 equiv.) was added followed by adding 4-azidobutan-1-amine[20] (285 mg, 2.5 mmol, 0.83 equiv.) to the reaction and left to run at rt overnight. After completion, the reaction was quenched with H_2O (7 mL). The mixture was then extracted with DCM (3 × 7 mL). The DCM layers were collected, washed with saturated Na_2CO_3, then washed with brine, dried with Na_2SO_4, and concentrated *in vacuo*. The residue was purified by flash chromatography to give **S-28** as a colorless oil (35% yield).

(*S*)-*N*-(4-Azidobutyl)-2-(4-isobutylphenyl)propanamide (S-28): ^{1}H NMR (500 MHz, $CDCl_3$) δ 7.18 (d, *J* = 8.2 Hz, 2H), 7.12 (d, *J* = 8.1 Hz, 2H), 5.33 (br s, 1H), 3.52 (q, *J* = 7.2 Hz, 1H), 3.28 – 3.16 (m, 4H), 2.46 (d, *J* = 7.2 Hz, 2H), 1.91 – 1.79 (m, 1H), 1.55 – 1.45 (m, 3H + 4H), 0.90 (d, *J* = 6.6 Hz, 6H). ^{13}C NMR (100 MHz, $CDCl_3$) δ 174.6, 140.8, 138.5, 129.7, 127.3, 51.0, 46.8, 45.0, 38.9, 30.2, 26.8, 26.1, 22.4, 18.4. HRMS (ESI) m/z: $[M + Na]^+$ calcd. for $[C_{17}H_{26}N_4NaO]^+$: 325.1999, found 325.1997. HRMS (ESI) m/z: $[M + H]^+$ calcd. for $[C_{17}H_{27}N_4O]^+$: 303.2179, found 303.2178.

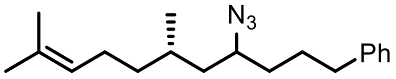

((6*S*)-4-Azido-6,10-dimethylundec-9-en-1-yl)benzene (S-29): Synthesized from (*S*)-(-)-Citronellal following the general procedure A and obtained as a colorless oil containing inseparable diastereomers (53% yield over 3 steps). ^{1}H NMR (500 MHz, $CDCl_3$) δ 7.35 – 7.28 (m, 2H), 7.20 (t, *J* = 7.3 Hz, 3H), 5.10 (t, *J* = 7.0 Hz, 1H), 3.42 – 3.27 (m, 1H), 2.75 – 2.60 (m, 2H), 2.10 – 1.89 (m, 2H), 1.87 – 1.77 (m, 1H), 1.70 (s, 3H), 1.62 (s, 3H), 1.60 – 1.51 (m, 3H), 1.49 – 1.35 (m, 2H), 1.33 – 1.26 (m, 2H), 1.25 – 1.09 (m, 1H), 0.94 – 0.89 (m, 3H). ^{13}C NMR (125 MHz, $CDCl_3$) δ 141.9, 131.5,

131.4, 128.3, 125.9, 124.5, 60.8, 60.6, 41.7, 37.6, 36.4, 35.6, 34.7, 34.2, 31.6, 29.5, 29.4, 27.9, 27.8, 25.7, 25.4, 25.2, 22.6, 19.9, 19.1, 17.6, 14.1. HRMS (ESI) m/z: $[M - N_2 + H]^+$ calcd. for $[C_{19}H_{30}N]^+$: 272.2373, found 272.2365.

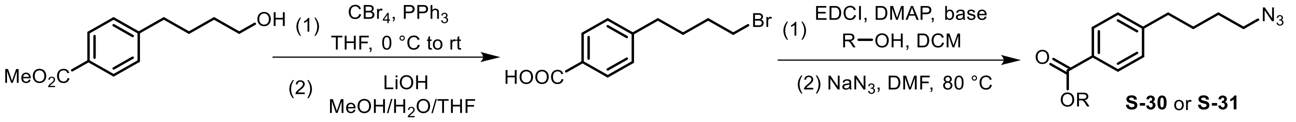

To a stirred solution of methyl 4-(4-hydroxybutyl)benzoate[21] (1.04 g, 5.0 mmol, 1,0 equiv.) and carbon tetrabromide (2.16 g, 6.5 mmol, 1.3 equiv.) in THF (60 mL), triphenylphosphine (1.64 g, 6.25 mmol, 1.25 equiv.) was added portionwise at 0 ºC, then the mixture was stirred at rt overnight. After completion the mixture was concentrated *in vacuo* before copious hexane was added, then the resulting suspension was filtered. After removing the solvent from the collected filtrate under reduced pressure, the residue was purified by silica gel column chromatography. Then the obtained bromide product was dissolved in MeOH/H_2O/THF (1:1:1, v/v/v, 45 mL), and after adding LiOH (10 equiv.) the mixture was heated at 50 ºC for 1 h. Upon completion, the organic solvents were removed *in vacuo*, and the residue was diluted with H_2O and washed with Et_2O. The aqueous solution was acidified to pH 2–3 with 2M HCl. And the resulting solution was extracted with EtOAc for 3 times, the EtOAc phases were collected and evaporated under reduced pressure to give the crude carboxylic acid which was used for next step without purification.

To a stirred, ice cooled solution of the crude carboxylic acid (257 mg, 1.0 mmol, 1.0 equiv.) and estrone (324 mg, 1.2 mmol, 1.2 equiv., for substrate **S-30**) or nopol (200 mg, 1.2 mmol, 1.2 equiv., for substrate **S-31**) in 15 mL DCM was added DMAP (25 mg, 0.2 mmol, 0.2 equiv.), EDCI (384 mg, 2.0 mmol, 2 equiv.) and DIPEA (350 µL, 2.0 mmol, 2 equiv., for substrate **S-30**) or Et_3N (312 µL, 2.25 mmol, 2.25 equiv., for substrate **S-31**) at 0 °C. The mixture was slowly warmed up to room temperature and stirred for 8 h. Upon completion of reaction (monitored by TLC), the reaction mixture was treated with water (10 mL) and DCM was removed under reduced pressure. Then the suspension was extracted with EtOAc (3 × 15 mL). The extract was dried over Na_2SO_4, filtered, and concentrated *in vacuo*. The residue was purified by silica gel column chromatography. Then the obtained ester product was treated with NaN_3 followed by the step 3 in the general procedure A to obtain substrates **S-30** and **S-31**.

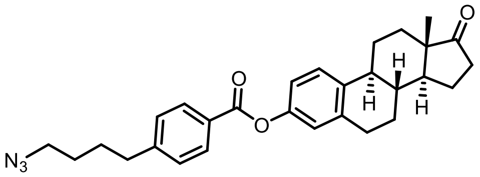

(8*R*,9*S*,13*S*,14*S*)-13-Methyl-17-oxo-7,8,9,11,12,13,14,15,16,17-decahydro-6*H*-cyclopenta[*a*]phenanthren-3-yl 4-(4-azidobutyl)benzoate (S-30): White solid (23% yield over 4 steps). ^{1}H NMR (400 MHz, $CDCl_3$) δ 8.11 (d, *J* = 8.3 Hz, 2H), 7.33 (d, *J* = 8.7 Hz, 2H), 7.30 (s, 1H), 6.98 (dd, *J* = 8.4, 2.6 Hz, 1H), 6.94 (d, *J* = 2.5 Hz, 1H), 3.31 (t, *J* = 6.7 Hz, 2H), 2.99 – 2.90 (m, 2H), 2.74 (t, *J* = 7.5 Hz, 2H), 2.51 (dd, *J* = 18.8, 8.6 Hz, 1H), 2.46 – 2.39 (m, 1H), 2.32 (td, *J* = 11.7, 11.1, 4.1 Hz, 1H), 2.22 – 1.94 (m, 4H), 1.81 – 1.70 (m, 2H), 1.70 – 1.58 (m, 5H), 1.57 – 1.41 (m, 3H), 0.92 (s, 3H). ^{13}C NMR (125 MHz, $CDCl_3$) δ 220.8, 165.4, 148.8, 148.1, 138.0, 137.3, 130.3, 128.6, 127.4, 126.4, 121.7, 118.9, 51.2, 50.4, 47.9, 44.2, 38.0, 35.9, 35.4, 31.6, 29.4, 28.4, 28.1, 26.3, 25.8, 21.6, 13.8. HRMS (ESI) m/z: $[M + H]^+$ calcd. for $[C_{29}H_{34}N_3O_3]^+$: 472.2595, found 472.2615.

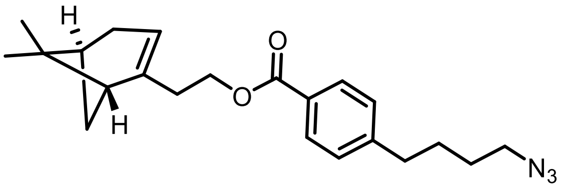

2-((1*R*,5*S*)-6,6-Dimethylbicyclo[3.1.1]hept-2-en-2-yl)ethyl 4-(4-azidobutyl)benzoate (S-31): Colorless oil. (25% yield over 4 steps). ^{1}H NMR (400 MHz, $CDCl_3$) δ 7.95 (d, *J* = 8.3 Hz, 2H), 7.23 (d, *J* = 8.2 Hz, 2H), 5.38 – 5.34 (m, 1H), 4.31 (tt, *J* = 6.8, 3.5 Hz, 2H), 3.29 (t, *J* = 6.7 Hz, 2H), 2.69 (t, *J* = 7.5 Hz, 2H), 2.46 – 2.34 (m, 3H), 2.31 – 2.16 (m, 2H), 2.15 – 2.06 (m, 2H), 1.78 – 1.68 (m, 2H), 1.66 – 1.58 (m, 2H), 1.27 (s, 3H), 1.17 (d, *J* = 8.5 Hz, 1H), 0.84 (s, 3H). ^{13}C NMR (100 MHz, $CDCl_3$) δ 166.6, 147.2, 144.3, 129.7, 128.4, 128.3, 118.9, 63.2, 51.3, 45.8, 40.7, 38.0, 36.1, 35.4, 31.7, 31.4, 28.4, 28.1, 26.3, 21.2. HRMS (ESI) m/z: $[M + Na]^+$ calcd. for $[C_{22}H_{29}N_3NaO_2]^+$: 390.2152, found 390.2149.

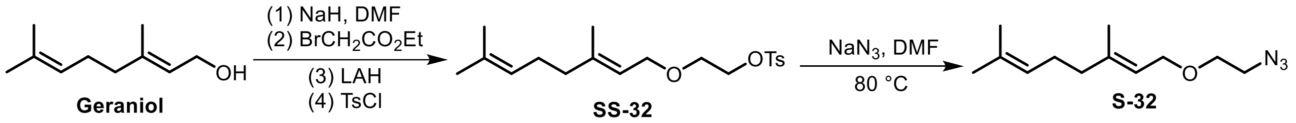

(*E*)-2-((3,7-Dimethylocta-2,6-dien-1-yl)oxy)ethyl 4-methylbenzenesulfonate (SS-32): Synthesized from Geraniol following similar procedure reported previously,[19] and obtained as a colorless oil (21% total yield). ^{1}H NMR (500 MHz, $CDCl_3$) δ 7.80 (d, *J* = 8.3 Hz, 2H), 7.33 (d, *J* = 8.0 Hz, 2H), 5.27 – 5.21 (m, 1H), 5.10 – 5.05 (m, 1H), 4.16 (t, *J* = 5.0 Hz, 2H), 3.96 (d, *J* = 6.8 Hz, 2H), 3.61 (t, *J* = 5.0 Hz, 2H), 2.44 (s, 3H), 2.12 – 2.05 (m, 2H), 2.04 – 1.99 (m, 2H), 1.68 (s, 3H), 1.63 (s, 3H), 1.60 (s, 3H). ^{13}C NMR (125 MHz, $CDCl_3$) δ 144.70, 140.82, 133.04, 131.71, 129.74, 127.96, 123.83, 120.14, 69.29, 67.61, 67.08, 39.54, 26.31, 25.66, 21.60, 17.66, 16.41. HRMS (ESI) m/z: $[M + Na]^+$ calcd. for $[C_{19}H_{28}NaO_4S]^+$: 375.1601, found 375.1600.

(*E*)-1-(2-azidoethoxy)-3,7-dimethylocta-2,6-diene (S-32): Synthesized following the step 3 in general procedure A and obtained as a colorless oil (79% yield). ^{1}H NMR (500 MHz, $CDCl_3$) δ 5.39 – 5.33 (m, 1H), 5.12 – 5.06 (m, 1H), 4.05 (d, *J* = 6.7 Hz, 2H), 3.60 (t, *J* = 5.3 Hz, 2H), 3.40 (t, *J* = 5.1 Hz, 2H), 2.15 – 2.08 (m, 2H), 2.08 – 2.02 (m, 2H), 1.68 (s, 6H), 1.60 (s, 3H). ^{13}C NMR (125 MHz, $CDCl_3$) δ 140.76, 131.67, 123.88, 120.34, 68.30, 67.57, 50.81, 39.55, 26.30, 25.64, 17.64, 16.42. HRMS (ESI) m/z: $[M + H]^+$ calcd. for $[C_{12}H_{22}N_3O]^+$: 224.1757, found 224.1752.

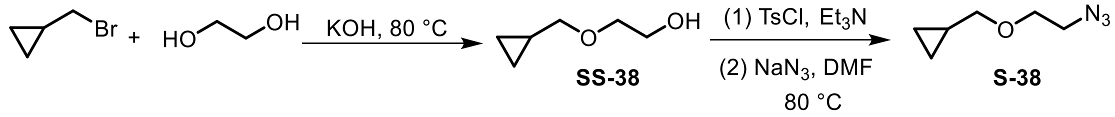

((2-Azidoethoxy)methyl)cyclopropane (S-38): Synthesized following the steps 2 and 3 in general procedure A starting from **SS-38** (prepared following similar procedure reported previously[22]), and obtained as a colorless oil. ^{1}H NMR (500 MHz, $CDCl_3$) δ 3.65 (t, *J* = 5.1 Hz, 2H), 3.40 (t, *J* = 5.1 Hz, 2H), 3.34 (d, *J* = 6.8 Hz, 2H), 1.14 – 1.02 (m, 1H), 0.59 – 0.51 (m, 2H), 0.26 – 0.20 (m, 2H). ^{13}C NMR (100 MHz, $CDCl_3$) δ 75.96, 69.13, 50.78, 10.50, 2.96. GCMS (EI) t_R = 8.7 min; m/z: 55, 72, 112 ($–N_2 – H^+$).

4. Screening of reaction conditions

Table S1. Screening of reaction conditions.[a]

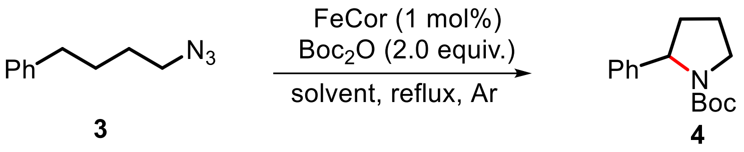

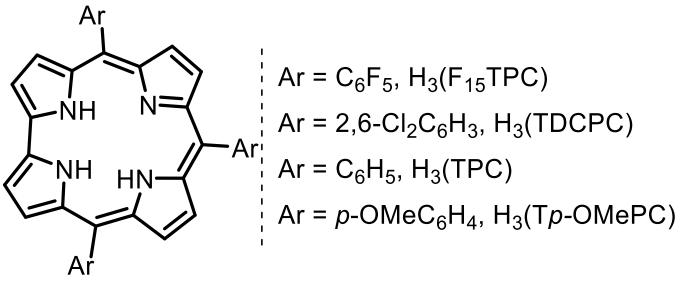

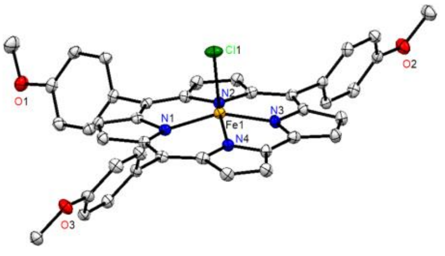

[Fe(T*p*-OMePC)Cl] (**1**)

Entry	Catalyst	Solvent	Time (h)	Conv. (%)[b]	Yield (%)[b]
1	[Fe(TPC)Cl]	toluene	3	>99	68
2	$[Fe(TPC)]_2O$	toluene	3	>99	52
3	$[Fe(F_{15}TPC)Cl]$	toluene	3	77	41
4	[Fe(TDCPC)Cl]	toluene	3	>99	58
5	[Fe(T*p*-OMePC)Cl] (**1**)	toluene	3	>99	85 (94)[c]
6	[Fe(T*p*-OMePC)Cl] (**1**)	PhCl	3	>99	75
7	[Fe(T*p*-OMePC)Cl] (**1**)	DMF	3	70	N.D.[d]
8	[Fe(T*p*-OMePC)Cl] (**1**)	DMSO	3	<10	N.D.[d]
9	[Fe(T*p*-OMePC)Cl] (**1**)	dioxane	3	10	8
10[e]	[Fe(T*p*-OMePC)] (**2**)	toluene	3	>99	70
11[f]	[Fe(T*p*-OMePC)Cl] (**1**)	toluene	16	>99	80
12[g]	[Fe(T*p*-OMePC)Cl] (**1**)	toluene	16	<5	trace
13[h]	[Fe(T*p*-OMePC)Cl] (**1**)	toluene	3	83	N.D.[d]
14	none	toluene	3	0	N.D.[d]

[a]Reaction conditions: Azide **3** (0.2 mmol), FeCor (1 mol%), and Boc_2O (0.4 mmol) in indicated solvent (2.0 mL) were refluxed under argon (Ar) atmosphere. [b]Determined by ^1H NMR using 1,3,5-trimethoxybenzene as an internal standard. [c]0.05 mol% **1** was used for 24 h to achieve a TON of 1880. [d]N.D. = not detected. [e]**2** was *in situ* generated by reacting **1** with 5.0 equiv. Zn for 5 h before adding **3** and Boc_2O. [f]At 100 °C. [g]At 80 °C. [h]Without Boc_2O.

5. General procedure for catalytic C–H amination

An oven-dried Schlenk tube was charged with alkyl azides (0.2 mmol, 1.0 equiv.), Boc_2O (0.4 mmol, 2.0 equiv.), FeCor catalyst (1 mol%), and dry toluene (2.0 mL) under argon. The mixture was refluxed (oil bath: 130 °C) until full completion detected by TLC (usually completed within 2–5 h). The reaction mixture was cooled to rt and concentrated, and products were purified via silica chromatography using mixture of hexane and EtOAc as eluents. Figure S1 and Figure S2 show the reaction progress of a typical catalytic reaction.

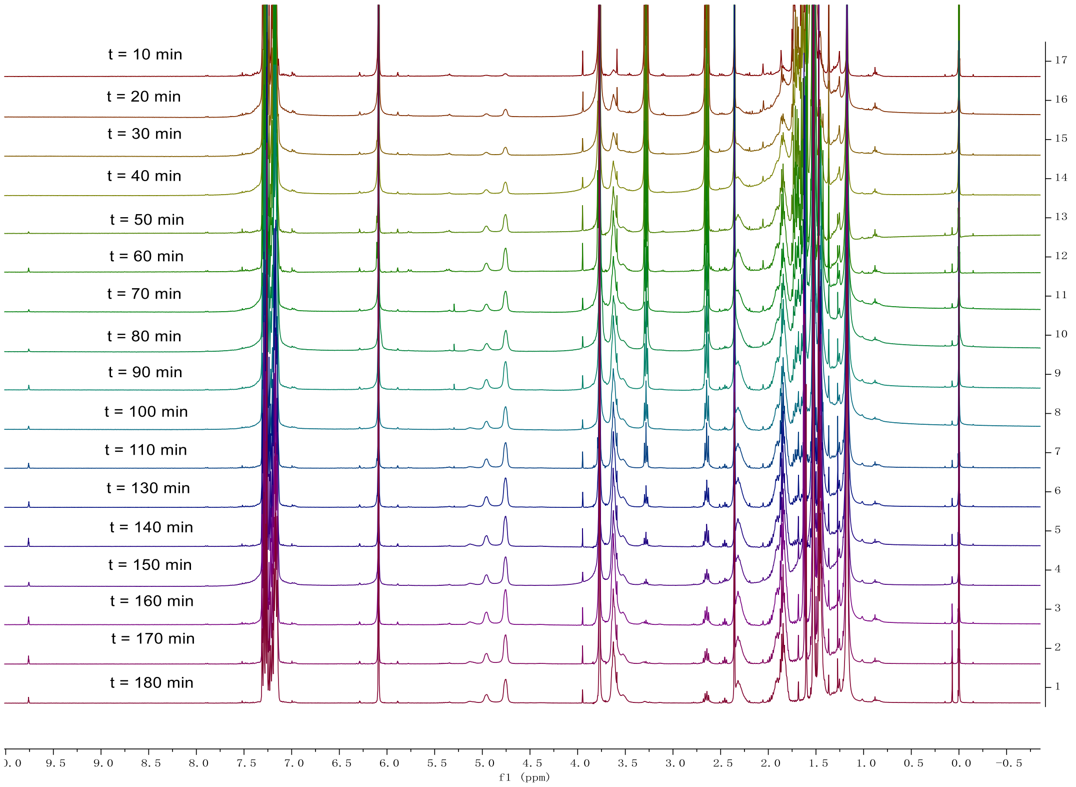

Figure S1. Stacked ^{1}H NMR spectra (in $CDCl_3$) of a typical catalytic reaction. Catalytic conditions: [**1**] = 1.0 mM, [**3**] = 100 mM, [Boc_2O] = 200 mM.

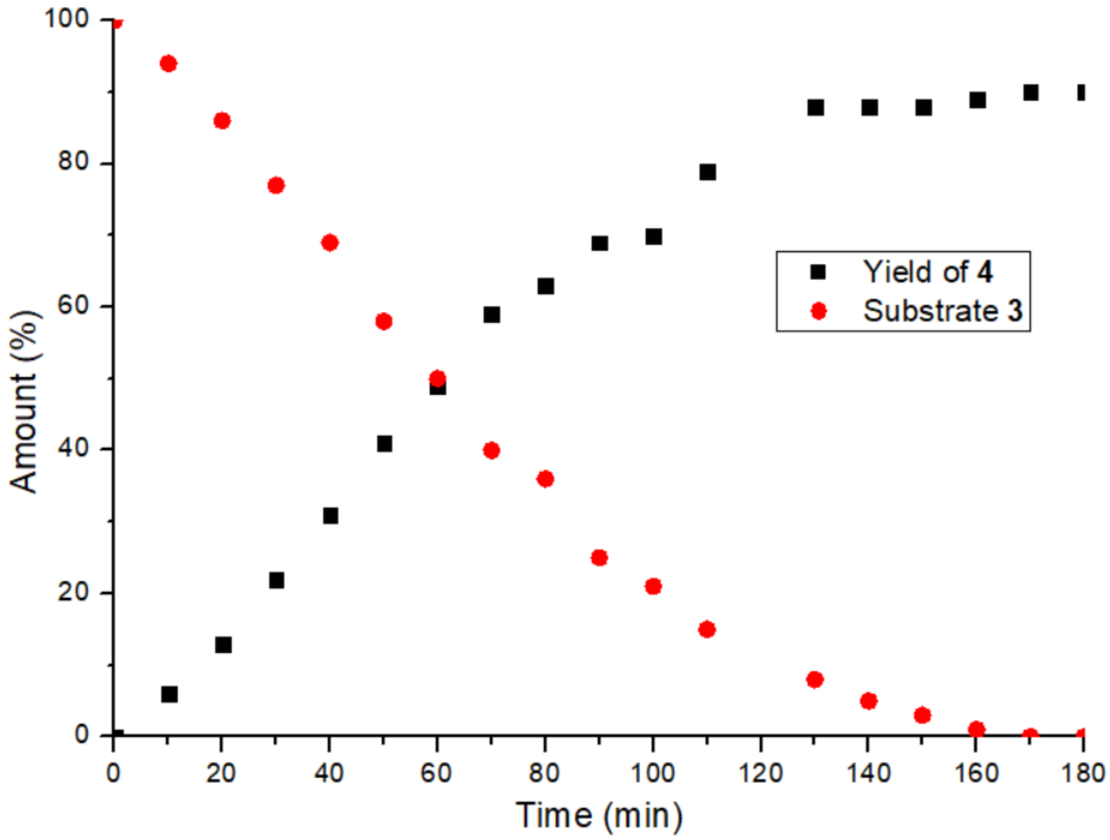

Figure S2. Reaction progress ([**1**] = 1.0 mM, [**3**]$_0$ = 100 mM, [Boc_2O] = 200 mM).

As for the gram scale reaction of **S-19**, the detailed reaction conditions were as follows: **S-19** (1.2 g, 6.34 mmol), Boc_2O (2.77 g, 12.68 mmol), and **1** (9.0 mg, 0.013 mmol) were mixed in dry toluene (24 mL), and refluxed at 130 °C for 48 h. The desired product **19** was obtained in 91% isolated yield (1.51 g) after silica chromatography.

As for reactions with low catalyst loading to afford high TONs (e.g. **18** and **34**), initially a stock catalyst solution was prepared by dissolving 0.7 mg **1** in 1 mL DCM. Then 50 µL (for **S-18**) or 140 µL (for **S-34**) of the prepared stock solution was transferred to a Schlenk tube, after removing DCM under high vacuum, azide (0.2 mmol), Boc_2O (0.4 mmol) and dry toluene (2 mL) was added under Ar. The resulting mixture was refluxed at 130 °C for 24 h and 44 h, respectively. Upon completion, solvent was removed *in vacuo* and the residue was purified by silica chromatography.

6. Characterization data of products

For known products reported previously, usually only ^{1}H NMR spectrum for each compound was given to verify the product's purity, together with the reported literature cited. In addition, most spectra were shown with the existence of amide rotamers.

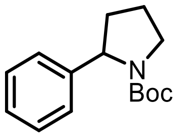

***tert*-Butyl 2-phenylpyrrolidine-1-carboxylate (4):** Colorless oil. ^{1}H NMR (500 MHz, $CDCl_3$) δ

7.28 (t, *J* = 7.5 Hz, 2H), 7.24 – 7.12 (m, 3H), 5.07 – 4.65 (br m, 1H), 3.71 – 3.41 (br m, 2H), 2.38 – 2.20 (br m, 1H), 1.98 – 1.77 (m, 3H), 1.53 – 1.11 (br m, 3H + 6H). For other detailed characterizations, see reference [23].

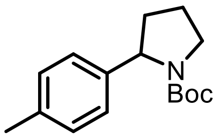

***tert*-Butyl 2-(*p*-tolyl)pyrrolidine-1-carboxylate (5):** Colorless solid. ^{1}H NMR (400 MHz, $CDCl_3$) δ 7.19 – 7.0 (m, 4H), 5.05 – 4.68 (br m, 1H), 3.75 – 3.42 (br m, 2H), 2.42 – 2.17 (s + br m, 3H + 1H), 1.99 – 1.77 (br m, 3H), 1.56 – 1.09 (br m, 3H + 6H). For other detailed characterizations, see reference [10].

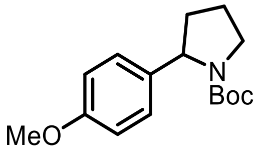

***tert*-Butyl 2-(4-methoxyphenyl)pyrrolidine-1-carboxylate (6):** Colorless oil. ^{1}H NMR (400 MHz, $CDCl_3$) δ 7.08 (d, *J* = 8.1 Hz, 2H), 6.83 (d, *J* = 8.7 Hz, 2H), 5.01 – 4.62 (br m, 1H), 3.79 (s, 3H), 3.69 – 3.43 (br m, 2H), 2.39 – 2.14 (br m, 1H), 1.96 – 1.76 (m, 3H), 1.59 – 1.04 (br m, 3H + 6H). For other detailed characterizations, see reference [10].

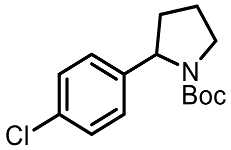

***tert*-Butyl 2-(4-chlorophenyl)pyrrolidine-1-carboxylate (7):** White solid. ^{1}H NMR (500 MHz, $CDCl_3$) δ 7.26 (d, *J* = 8.7 Hz, 2H), 7.10 (d, *J* = 8.0 Hz, 2H), 5.02 – 4.63 (br m, 1H), 3.71 – 3.42 (br m, 2H), 2.40 – 2.21 (br m, 1H), 1.96 – 1.83 (m, 2H), 1.81 – 1.72 (m, 1H), 1.51 – 1.10 (br m, 3H + 6H). For other detailed characterizations, see reference [10].

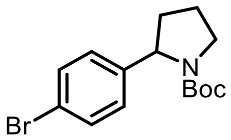

***tert*-Butyl 2-(4-bromophenyl)pyrrolidine-1-carboxylate (8):** Light yellow oil. ^{1}H NMR (400 MHz, $CDCl_3$) δ 7.40 (d, *J* = 8.4 Hz, 2H), 7.04 (d, *J* = 8.1 Hz, 2H), 4.96 – 4.62 (br m, 1H), 3.69 – 3.41 (br m, 2H), 2.37 – 2.22 (br m, 1H), 1.92 – 1.81 (m, 2H), 1.79 – 1.71 (m, 1H), 1.47 – 1.15 (br m, 3H + 6H). For other detailed characterizations, see reference [24].

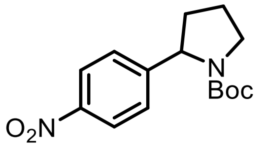

***tert*-Butyl 2-(4-nitrophenyl)pyrrolidine-1-carboxylate (9):** Light yellow oil. ^{1}H NMR (500 MHz, $CDCl_3$) δ 8.18 (d, *J* = 7.9 Hz, 2H), 7.35 (d, *J* = 8.5 Hz, 2H), 5.08 – 4.76 (br m, 1H), 3.74 – 3.52 (br

m, 2H), 2.47 – 2.32 (br m, 1H), 1.96 – 1.88 (m, 2H), 1.85 – 1.77 (m, 1H), 1.48 – 1.15 (br m, 3H + 6H). For other detailed characterizations, see reference [24].

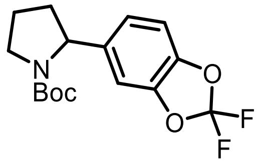

***tert*-Butyl 2-(2,2-difluorobenzo[*d*][1,3]dioxol-5-yl)pyrrolidine-1-carboxylate (10):** Colorless oil. ^{1}H NMR (400 MHz, $CDCl_3$) δ 6.97 (d, J = 8.5 Hz, 1H), 6.93 – 6.83 (m, 2H), 5.04 – 4.66 (br m, 1H), 3.71 – 3.44 (br m, 2H), 2.39 – 2.22 (br m, 1H), 1.94 – 1.83 (br m, 2H), 1.82 – 1.73 (br m, 1H), 1.53 – 1.15 (br m, 4H + 5H). For other detailed characterizations, see reference [11].

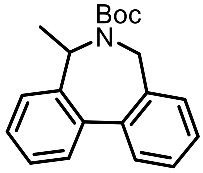

***tert*-Butyl 5-methyl-5,7-dihydro-6*H*-dibenzo[*c*,*e*]azepine-6-carboxylate (11):** Colorless oil. ^{1}H NMR (500 MHz, $CDCl_3$) δ 7.54 – 7.47 (m, 2H), 7.46 – 7.29 (m, 6H), 5.30 – 4.76 (br m, 2H), 3.72 (br s, 1H), 1.53 (s, 9H), 0.86 (d, J = 6.9 Hz, 3H). For other detailed characterizations, see reference [25].

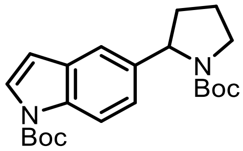

***tert*-Butyl 5-(1-(*tert*-butoxycarbonyl)pyrrolidin-2-yl)-1H-indole-1-carboxylate (12):** Colorless oil. ^{1}H NMR (400 MHz, $CDCl_3$) δ 8.05 (d, J = 7.9 Hz, 1H), 7.57 (s, 1H), 7.33 (s, 1H), 7.12 (d, J = 8.2 Hz, 1H), 6.51 (d, J = 3.7 Hz, 1H), 5.10 – 4.81 (br m, 1H), 3.76 – 3.47 (br m, 2H), 2.43 – 2.24 (br m, 1H), 1.99 – 1.90 (m, 1H), 1.90 – 1.80 (m, 2H), 1.66 (s, 9H), 1.51 – 1.09 (br m, 3H + 6H). For other detailed characterizations, see reference [26].

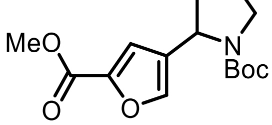

***tert*-Butyl 2-(5-(methoxycarbonyl)furan-3-yl)pyrrolidine-1-carboxylate (13):** Colorless oil. ^{1}H NMR (500 MHz, $CDCl_3$) δ 7.46 – 7.31 (m, 1H), 7.05 (s, 1H), 4.97 – 4.64 (br m, 1H), 3.86 (s, 3H), 3.56 – 3.30 (br m, 2H), 2.26 – 2.07 (br m, 1H), 1.92 – 1.82 (m, 3H), 1.50 – 1.27 (br m, 4H + 5H). For other detailed characterizations, see reference [11].

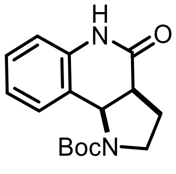

***tert*-Butyl 4-oxo-2,3,3a,4,5,9b-hexahydro-1*H*-pyrrolo[3,2-*c*]quinoline-1-carboxylate (14):** White solid. ^{1}H NMR (600 MHz, $CDCl_3$) δ 9.45 (br s, 1H), 7.47 (br s, 1H), 7.21 (t, *J* = 7.5 Hz, 1H), 7.06 (t, *J* = 7.5 Hz, 1H), 6.85 (d, *J* = 7.8 Hz, 1H), 5.34 (br s, 1H), 3.54 (br s, 1H), 3.42 – 3.28 (m, 1H), 3.07 (dt, *J* = 11.8, 7.3 Hz, 1H), 2.46 – 2.29 (m, 1H), 2.06 (br s, 1H), 1.54 (s, 9H). ^{13}C NMR (150 MHz, $CDCl_3$) δ 171.2, 155.2, 134.6, 128.7, 123.8, 122.5, 115.6, 80.3, 56.7, 44.4, 43.9, 29.7, 28.5. HRMS (ESI) m/z: $[M + H]^+$ calcd. for $[C_{16}H_{21}N_2O_3]^+$: 289.1547, found 289.1545.

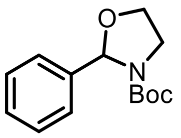

***tert*-Butyl 2-phenyloxazolidine-3-carboxylate (15):** Light yellow oil. ^{1}H NMR (400 MHz, $CDCl_3$) δ 7.50 – 7.29 (m, 5H), 6.20 – 5.84 (br m, 1H), 4.16 – 4.05 (br m, 1H), 4.05 – 3.97 (m, 1H), 3.82 (br s, 1H), 3.61 – 3.48 (br m, 1H), 1.30 (br s, 9H). For other detailed characterizations, see reference [27].

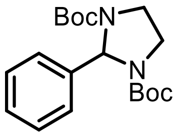

Di-*tert*-butyl 2-phenylimidazolidine-1,3-dicarboxylate (16): Light yellow oil. ^{1}H NMR (500 MHz, $CDCl_3$) δ 7.43 – 7.26 (m, 5H), 6.41 – 5.73 (br m, 1H), 3.91 – 3.55 (br m, 4H), 1.47 – 1.28 (br m, 18H). For other detailed characterizations, see reference [9].

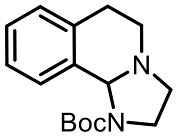

***tert*-Butyl 2,3,6,10b-tetrahydroimidazo[2,1-*a*]isoquinoline-1(5*H*)-carboxylate (17):** Light yellow oil. ^{1}H NMR (400 MHz, $CDCl_3$) δ 7.58 (s, 1H), 7.24 – 7.13 (m, 2H), 7.05 (d, *J* = 7.0 Hz, 1H), 5.78 (s, 1H), 3.66 (q, *J* = 9.2 Hz, 1H), 3.41 (td, *J* = 13.1, 12.5, 4.6 Hz, 1H), 3.32 (t, *J* = 8.7 Hz, 1H), 3.23 – 3.13 (m, 1H), 3.10 – 2.93 (m, 3H), 2.53 (d, *J* = 16.6 Hz, 1H), 1.52 (s, 9H). For other detailed characterizations, see reference [12].

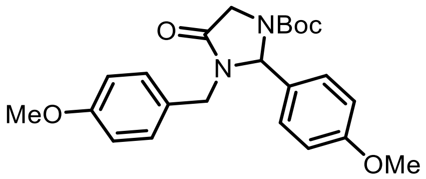

***tert*-Butyl 3-(4-methoxybenzyl)-2-(4-methoxyphenyl)-4-oxoimidazolidine-1-carboxylate (18):** Colorless oil. ^{1}H NMR (500 MHz, $CDCl_3$) δ 7.20 (d, *J* = 7.4 Hz, 1H), 7.12 (d, *J* = 7.9 Hz, 1H), 7.07 (d, *J* = 7.8 Hz, 2H), 6.91 (d, *J* = 7.9 Hz, 2H), 6.89 – 6.82 (m, 2H), 5.73 – 5.41 (m, 1H), 5.02 (d, *J* = 14.8 Hz, 1H), 4.28 – 4.10 (m, 2H), 3.84 (s, 3H), 3.81 (s, 3H), 3.38 – 3.22 (m, 1H), 1.44 – 1.11 (s+s, 4H + 5H). For other detailed characterizations, see reference [13].

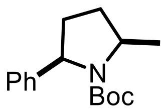

***tert*-Butyl 2-methyl-5-phenylpyrrolidine-1-carboxylate (19):** Colorless solid (*d.r.*>99:1). ^{1}H NMR (400 MHz, $CDCl_3$) δ 7.38 – 7.29 (m, 2H), 7.29 – 7.17 (m, 3H), 5.07 – 4.59 (br m, 1H), 4.36 – 3.89 (br m, 1H), 2.33 – 2.20 (m, 1H), 2.10 – 1.99 (m, 1H), 1.97 – 1.84 (m, 1H), 1.66 – 1.57 (m, 1H), 1.55 – 1.11 (m, 3H + 3H + 6H). For other detailed characterizations, see reference [23].

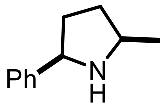

***cis*-2-Methyl-5-phenylpyrrolidine (19'):** (the deprotection product of **19**; *d.r.*>99:1). Colorless oil. ^{1}H NMR (400 MHz, $CDCl_3$) δ 7.37 (d, *J* = 7.6 Hz, 2H), 7.31 (t, *J* = 7.5 Hz, 2H), 7.22 (t, *J* = 7.2 Hz, 1H), 4.15 (t, *J* = 7.9 Hz, 1H), 3.30 (h, *J* = 6.6 Hz, 1H), 2.23 – 2.11 (m, 1H), 2.03 – 1.92 (m + br s, 1H + 1H), 1.81 – 1.69 (m, 1H), 1.51 – 1.39 (m, 1H), 1.24 (d, *J* = 6.3 Hz, 3H). ^{13}C NMR (100 MHz, $CDCl_3$) δ 144.7, 128.3, 126.8, 126.6, 63.0, 54.8, 34.1, 33.6, 21.6. For other detailed characterizations, see reference [28].

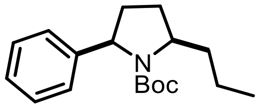

***tert*-Butyl 2-phenyl-5-propylpyrrolidine-1-carboxylate (20):** Colorless oil (*d.r.*>99:1). ^{1}H NMR (400 MHz, $CDCl_3$) δ 7.34 – 7.25 (m, 2H), 7.25 – 7.16 (m, 3H), 4.99 – 4.55 (br m, 1H), 3.95 (br s, 1H), 2.31 – 2.18 (m, 1H), 2.11 – 1.91 (m, 2H), 1.90 – 1.79 (m, 1H), 1.76 – 1.61 (br m, 1H), 1.56 – 1.29 (m, 6H), 1.28 – 1.06 (br m, 6H), 0.99 (t, *J* = 7.2 Hz, 3H). ^{13}C NMR (100 MHz, $CDCl_3$) δ 155.0, 145.2, 128.2, 126.4, 125.6, 79.1, 62.9, 59.1, 37.5, 34.8, 29.3, 28.2, 20.2, 14.2. HRMS (ESI) m/z: $[M + H]^+$ calcd. for $[C_{18}H_{28}NO_2]^+$: 290.2115, found 290.2119; HRMS (ESI) m/z: $[M + Na]^+$ calcd. for $[C_{18}H_{27}NNaO_2]^+$: 312.1934, found 312.1939.

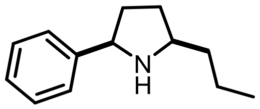

***cis*-2-Phenyl-5-propylpyrrolidine (20'):** (the deprotection product of **20**; *d.r.*>99:1). Colorless oil. ^{1}H NMR (500 MHz, $CDCl_3$) δ 7.38 (d, *J* = 7.7 Hz, 2H), 7.31 (t, *J* = 7.5 Hz, 2H), 7.22 (t, *J* = 7.3 Hz, 1H), 4.14 (t, *J* = 8.0 Hz, 1H), 3.18 (p, *J* = 7.0 Hz, 1H), 2.20 – 2.10 (m, 1H), 2.02 – 1.94 (m, 1H), 1.90 (br s, 1H), 1.77 – 1.67 (m, 1H), 1.62 – 1.54 (m, 1H), 1.53 – 1.34 (m, 4H), 0.94 (t, *J* = 7.1 Hz, 3H). ^{13}C NMR (125 MHz, $CDCl_3$) δ 144.5, 128.3, 126.8, 126.7, 62.6, 59.3, 38.9, 33.7, 31.6, 20.5, 14.3. HRMS (ESI) m/z: $[M + H]^+$ calcd. for $[C_{13}H_{20}N]^+$: 190.1590, found 190.1590.

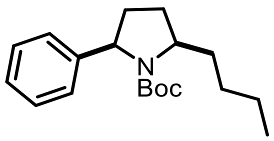

***tert*-Butyl 2-butyl-5-phenylpyrrolidine-1-carboxylate (21):** Colorless oil (*d.r.*>99:1). ^{1}H NMR (500 MHz, $CDCl_3$) δ 7.32 – 7.27 (m, 2H), 7.25 – 7.17 (m, 3H), 4.97 – 4.57 (br m, 1H), 3.93 (br s, 1H), 2.30 – 2.20 (m, 1H), 2.11 – 1.91 (br m, 2H), 1.90 – 1.77 (m, 1H), 1.73 – 1.61 (br m, 1H), 1.50 –

1.29 (m, 8H), 1.27 – 1.09 (br m, 6H), 0.94 (t, *J* = 6.8 Hz, 3H). ^{13}C NMR (125 MHz, $CDCl_3$) δ 155.0, 145.2, 128.1, 126.3, 125.5, 79.0, 62.8, 59.2, 35.0, 34.8, 29.2, 28.2, 27.4, 22.7, 14.2. HRMS (ESI) m/z: $[M + H]^+$ calcd. for $[C_{19}H_{30}NO_2]^+$: 304.2271, found 304.2270; HRMS (ESI) m/z: $[M + Na]^+$ calcd. for $[C_{19}H_{29}NNaO_2]^+$: 326.2091, found 326.2090.

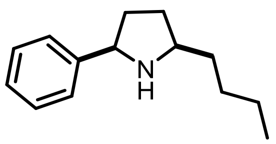

***cis*-2-Butyl-5-phenylpyrrolidine (21'):** (the deprotection product of **21**; *d.r.*>99:1). Colorless oil. ^{1}H NMR (400 MHz, $CDCl_3$) δ 7.38 (d, *J* = 7.6 Hz, 2H), 7.31 (t, *J* = 7.5 Hz, 2H), 7.22 (t, *J* = 7.3 Hz, 1H), 4.12 (t, *J* = 7.9 Hz, 1H), 3.14 (p, *J* = 7.0 Hz, 1H), 2.21 – 2.08 (m, 1H), 2.03 – 1.89 (m, 1H), 1.82 (br s, 1H) 1.75 – 1.63 (m, 1H), 1.63 – 1.54 (m, 1H), 1.52 – 1.43 (m, 2H), 1.42 – 1.28 (m, 4H), 0.92 (t, *J* = 6.8 Hz, 3H). ^{13}C NMR (100 MHz, $CDCl_3$) δ 144.9, 128.3, 126.8, 126.7, 62.7, 59.6, 36.6, 33.9, 31.7, 29.6, 23.0, 14.1. For other detailed characterizations, see reference [28a].

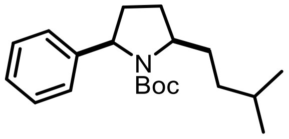

***tert*-Butyl 2-isopentyl-5-phenylpyrrolidine-1-carboxylate (22):** Colorless oil (*d.r.*>99:1). ^{1}H NMR (500 MHz, $CDCl_3$) δ 7.30 (t, *J* = 7.5 Hz, 2H), 7.25 – 7.16 (m, 3H), 4.99 – 4.58 (br m, 1H), 3.90 (br s, 1H), 2.30 – 2.19 (m, 1H), 2.10 – 1.91 (br m, 2H), 1.90 – 1.81 (br m, 1H), 1.71 – 1.55 (br m, 3H), 1.52 – 1.38 (br m, 3H), 1.33 – 1.07 (br m, 8H), 0.92 (dd, *J* = 6.6, 3.0 Hz, 6H). ^{13}C NMR (100 MHz, $CDCl_3$) δ 155.0, 145.1, 128.2, 126.4, 125.6, 79.1, 62.9, 59.5, 36.2, 34.7, 33.1, 28.2, 28.1, 22.9, 22.7. HRMS (EI) m/z: $[M]^+$ calcd. for $[C_{20}H_{31}NO_2]^+$: 317.2355, found 317.2352.

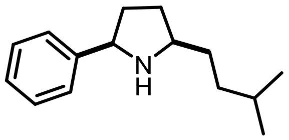

***cis*-2-Isopentyl-5-phenylpyrrolidine (22'):[29]** (the deprotection product of **22**; *d.r.*>99:1). Colorless oil. ^{1}H NMR (400 MHz, MeOD) δ 7.39 (d, *J* = 7.5 Hz, 2H), 7.33 (t, *J* = 7.5 Hz, 2H), 7.25 (t, *J* = 7.5 Hz, 1H), 4.07 (t, *J* = 8.2 Hz, 1H), 3.09 (p, *J* = 7.4 Hz, 1H), 2.27 – 2.15 (m, 1H), 2.13 – 2.00 (m, 1H), 1.85 – 1.73 (m, 1H), 1.72 – 1.64 (m, 1H), 1.63 – 1.46 (m, 3H), 1.41 – 1.24 (m, 2H), 0.95 (d, *J* = 6.6 Hz, 6H). ^{13}C NMR (100 MHz, MeOD) δ 142.9, 128.0, 126.7, 126.4, 62.8, 60.1, 36.4, 33.2, 32.8, 31.3, 28.0, 21.7, 21.6. HRMS (ESI) m/z: $[M + H]^+$ calcd. for $[C_{15}H_{24}N]^+$: 218.1903, found 218.1900.

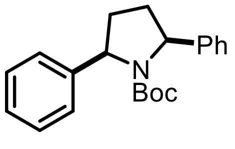

***tert*-Butyl 2,5-diphenylpyrrolidine-1-carboxylate (23):** Colorless oil (*d.r.*>99:1). ^{1}H NMR (400 MHz, $CDCl_3$) δ 7.48 – 7.28 (m, 8H), 7.27 – 7.20 (m, 2H), 5.24 – 4.75 (br m, 2H), 2.41 – 2.25 (m, 2H), 2.16 – 1.97 (br m, 2H), 1.21 (s, 9H). For other detailed characterizations, see reference [14].

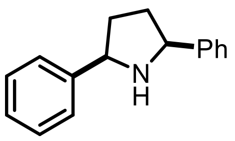

***cis*-2,5-Diphenylpyrrolidine (23'):** (the deprotection product of **23**; *d.r.*>99:1). Colorless oil. ^{1}H NMR (400 MHz, $CDCl_3$) δ 7.51 (d, *J* = 7.6 Hz, 4H), 7.34 (t, *J* = 7.5 Hz, 4H), 7.24 (t, *J* = 6.9 Hz, 2H), 4.33 (t, *J* = 5.6 Hz, 2H), 2.32 – 2.20 (m, 2H), 2.11 (br s, 1H), 1.87 – 1.77 (m, 2H). For other detailed characterizations, see reference [28a].

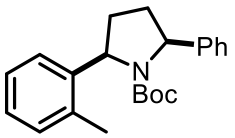

***tert*-Butyl 2-phenyl-5-(*o*-tolyl)pyrrolidine-1-carboxylate (24):** Colorless oil (*d.r.*>99:1). ^{1}H NMR (400 MHz, $CDCl_3$) δ 7.56 (d, *J* = 7.2 Hz, 2H), 7.50 – 7.39 (m, 3H), 7.36 – 7.27 (m, 1H), 7.24 – 7.13 (m, 3H), 5.33 – 4.94 (br m, 2H), 2.51 – 2.31 (m, 3H (s) + 2H), 2.24 – 2.09 (m, 1H), 1.95 – 1.83 (m, 1H), 1.23 (s, 9H). ^{13}C NMR (100 MHz, $CDCl_3$) δ 155.8, 143.6, 142.6, 134.2, 130.3, 128.3, 126.8, 126.7, 126.3, 126.0, 124.8, 79.7, 62.7, 60.4, 33.1, 28.1, 19.5. HRMS (ESI) m/z: $[M + H]^+$ calcd. for $[C_{22}H_{28}NO_2]^+$: 338.2115, found 338.2113; HRMS (ESI) m/z: $[M + Na]^+$ calcd. for $[C_{22}H_{27}NNaO_2]^+$: 360.1934, found 360.1932.

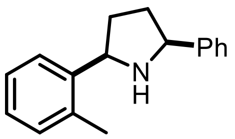

***cis*-2-Phenyl-5-(*o*-tolyl)pyrrolidine (24'):** (the deprotection product of **24**; *d.r.*>99:1). Colorless oil. ^{1}H NMR (500 MHz, $CDCl_3$) δ 7.88 (d, *J* = 7.6 Hz, 1H), 7.57 – 7.52 (m, 2H), 7.35 (t, *J* = 7.6 Hz, 2H), 7.28 – 7.20 (m, 2H), 7.17 – 7.12 (m, 2H), 4.53 (t, *J* = 7.7 Hz, 1H), 4.32 (t, *J* = 7.8 Hz, 1H), 2.38 (s, 3H), 2.32 (dtd, *J* = 12.0, 8.1, 6.5 Hz, 1H), 2.23 (dddd, *J* = 12.0, 8.4, 7.2, 4.8 Hz, 1H), 1.99 (br s, 1H), 1.80 (dddd, *J* = 12.0, 10.1, 8.5, 6.5 Hz, 1H), 1.69 (dddd, *J* = 12.1, 9.8, 7.4, 4.8 Hz, 1H). ^{13}C NMR (125 MHz, $CDCl_3$) δ 145.2, 143.5, 135.1, 130.1, 128.3, 126.8, 126.7, 126.3, 126.0, 125.6, 62.2, 58.4, 34.5, 32.8, 19.4. HRMS (ESI) m/z: $[M + H]^+$ calcd. for $[C_{17}H_{20}N]^+$: 238.1590, found 238.1588.

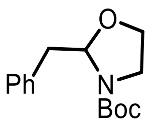

***tert*-Butyl 2-benzyloxazolidine-3-carboxylate (25):** Colorless oil. ^{1}H NMR (500 MHz, $CDCl_3$) δ 7.31 – 7.26 (m, 2H), 7.25 – 7.19 (m, 3H), 5.29 (br s, 1H), 3.90 (br s, 1H), 3.81 (q, *J* = 7.4 Hz, 1H), 3.72 – 3.39 (br m, 1H), 3.21 – 2.81 (br m, 3H), 1.50 (s, 9H). ^{13}C NMR (125 MHz, $CDCl_3$) (minor rotamer was shown in the parentheses) δ 152.8, 136.6, 129.9, 128.2, 126.5, 89.2, 80.2 (80.4), 65.4, 44.4 (44.8), 40.6 (39.6), 28.4. HRMS (ESI) m/z: $[M + Na]^+$ calcd. for $[C_{15}H_{21}NNaO_3]^+$: 286.1414, found 286.1429.

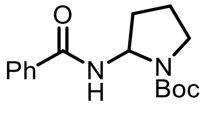

***tert*-Butyl 2-benzamidopyrrolidine-1-carboxylate (26):** Light yellow solid. ^1H NMR (500 MHz, $CDCl_3$) δ 7.60 – 7.31 (m, 5H), 5.72 – 5.05 (br, m, 2H), 3.81 – 3.25 (br, m, 2H), 2.45 – 1.67 (br, m, 4H), 1.55 – 1.27 (br m, 9H). For other detailed characterizations, see reference [11].

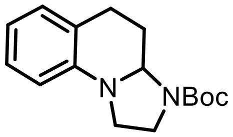

***tert*-Butyl 1,2,4,5-tetrahydroimidazo[1,2-*a*]quinoline-3(3a*H*)-carboxylate (27):** Light yellow oil. ^1H NMR (400 MHz, $CDCl_3$) δ 7.09 (t, J = 7.6 Hz, 1H), 7.02 (d, J = 7.4 Hz, 1H), 6.69 (t, J = 7.4 Hz, 1H), 6.57 (d, J = 8.0 Hz, 1H), 4.68 (d, J = 9.2 Hz, 1H), 3.75 (br s, 1H), 3.55 – 3.41 (m, 3H), 2.90 (ddd, J = 18.0, 13.1, 5.5 Hz, 1H), 2.79 (ddd, J = 16.7, 5.1, 2.2 Hz, 1H), 2.72 – 2.44 (br m, 1H), 1.55 – 1.44 (s+m, 9H + 1H). For other detailed characterizations, see reference [12].

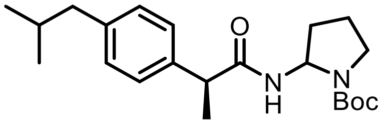

***tert*-Butyl 2-((*S*)-2-(4-isobutylphenyl)propanamido)pyrrolidine-1-carboxylate (28):** Colorless oil. ^1H NMR (400 MHz, $CDCl_3$) (mixture of rotamers and diastereomers) δ 7.41 – 6.98 (m, 4H), 5.84 – 5.29 (m, 1H), 5.26 – 4.63 (m, 1H), 4.08 – 3.65 (m, 1H), 3.64 – 3.08 (m, 2H), 2.43 (d + d, J = 7.2 Hz, 2H), 2.22 – 1.68 (m, 5H), 1.49 (s, 3H), 1.48 – 1.36 (br m, 9H), 0.89 (d, J = 6.4 Hz, 6H). ^{13}C NMR (100 MHz, $CDCl_3$) (mixture of rotamers and diastereomers) δ 174.5 (174.0, 173.1, 173.1), 154.8 (154.7, 154.3, 154.0), 140.3 (140.2, 140.0), 138.8, (139.1,138.5, 138.1), 129.4 (129.6, 129.5, 129.1), 127.4 (127.3, 127.2, 127.1), 80.1 (80.0, 79.5), 65.9 (65.8, 65.3, 64.3), 46.1 (46.7), 45.1 (45.5, 44.9, 44.7), 43.2 (43.1), 34.9 (34.3), 30.2 (31.6, 30.2, 29.7), 28.4 (28.3), 22.4 (23.4, 22.5, 22.4), 21.2 (21.1, 21.0), 20.7, (20.4, 20.3, 20.0). HRMS (ESI) m/z: $[M + Na]^+$ calcd. for $[C_{22}H_{34}N_2NaO_3]^+$: 397.2462, found 397.2457. The *d.r.* (1.8:1) was determined with HPLC analysis: CHIRALPAK ADH, 10% IPA in hexane, 1.0 mL/min, 220 nm, tr_{major} = 4.95 min, tr_{minor} = 6.05 min.

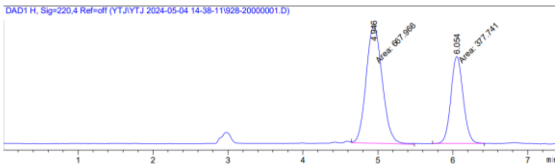

Peak #	RetTime [min]	Type	Width [min]	Area [mAU*s]	Height [mAU]	Area %
1	4.946	MM	0.2484	667.96649	44.81458	63.8770
2	6.054	MM	0.1891	377.74078	33.29792	36.1230

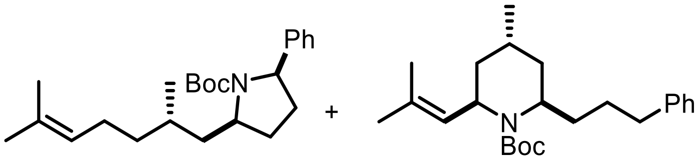

29a 29b

***tert*-Butyl 2-((*S*)-2,6-dimethylhept-5-en-1-yl)-5-phenylpyrrolidine-1-carboxylate (29a) and *tert*-butyl (4*R*)-4-methyl-2-(2-methylprop-1-en-1-yl)-6-(3-phenylpropyl)piperidine-1-carboxylate (29b):** Obtained as a mixture of isomers (colorless oil, with a 2:1 *r.r.*).^1H NMR (400 MHz, $CDCl_3$) δ 7.42 – 7.11 (m, 5H), 5.46 – 5.08 (br m, 1H), 5.05 – 4.62 (br m, 1H), 4.37 – 3.89 (br m, 1H), 2.73 – 1.82 (br m, 5H), 1.78 – 1.64 (br m, 6H), 1.60 – 1.53 (br m, 2H), 1.50 – 1.41 (br m, 4H), 1.38 – 1.10 (br m, 8H), 1.08 – 0.96 (br m, 2H), 0.95 – 0.82 (br m, 2H). ^{13}C NMR (100 MHz, $CDCl_3$) δ 155.3, 155.0, 154.9,

142.7, 131.1, 128.4, 128.4, 128.2, 128.2, 126.5, 126.4, 125.6, 125.5, 124.9, 124.8, 79.0, 62.8, 57.3, 57.3, 39.5, 38.2, 36.6, 36.0, 35.0, 30.4, 30.3, 29.7, 28.9, 28.6, 28.5, 28.3, 26.1, 25.8, 25.6, 22.8, 22.5, 20.6, 19.2, 18.0, 17.7. HRMS (ESI) m/z: $[M + Na]^+$ calcd. for $[C_{24}H_{37}NNaO_2]^+$: 394.2717, found 394.2719.

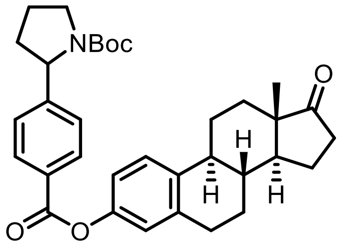

***tert*-Butyl 2-(4-((((8*R*,9*S*,13*S*,14*S*)-13-methyl-17-oxo-7,8,9,11,12,13,14,15,16,17-decahydro-6H-cyclopenta[*a*]phenanthren-3-yl)oxy)carbonyl)phenyl)pyrrolidine-1-carboxylate (30):** Colorless oil. ^{1}H NMR (500 MHz, $CDCl_3$) δ 8.12 (d, *J* = 8.3 Hz, 2H), 7.33 (d, *J* = 9.0 Hz, 1H), 7.30 (d, *J* = 8.2 Hz, 2H), 7.01 – 6.91 (m, 2H), 5.09 – 4.74 (br m, 1H), 3.72 – 3.49 (br m, 2H), 2.98 – 2.89 (m, 2H), 2.51 (dd, *J* = 19.0, 8.7 Hz, 1H), 2.46 – 2.29 (m, 3H), 2.15 (dt, *J* = 18.6, 8.9 Hz, 1H), 2.10 – 2.00 (m, 2H), 1.97 (dd, *J* = 12.5, 3.3 Hz, 1H), 1.90 (dt, *J* = 12.5, 6.2 Hz, 2H), 1.83 (tt, *J* = 11.8, 5.9 Hz, 1H), 1.67 – 1.58 (m, 3H), 1.57 – 1.49 (m, 3H), 1.46 (br s, 3H), 1.20 (br s, 6H), 0.92 (s, 3H). ^{13}C NMR (125 MHz, $CDCl_3$) (only the major rotamer was given) δ 220.7, 165.3, 154.4, 148.8, 138.0, 137.4, 130.2, 128.0, 126.4, 125.6, 121.7, 118.9, 79.6, 61.2, 50.4, 47.9, 47.1, 44.2, 38.0, 35.8, 31.6, 29.4, 28.4, 28.2, 26.3, 25.8, 23.2, 21.6, 13.8. HRMS (ESI) m/z: $[M + Na]^+$ calcd. for $[C_{34}H_{41}NNaO_5]^+$: 566.2877, found 566.2860. The *d.r.* was determined by HPLC analysis: CHIRALPAK IA, 20% IPA in hexane, 1.0 mL/min, 240 nm, tr_1 = 12.42 min, tr_2 = 13.33 min.

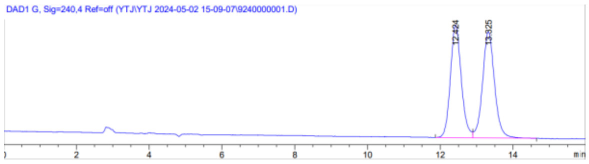

Peak #	RetTime [min]	Type	Width [min]	Area [mAU*s]	Height [mAU]	Area %
1	12.424	BV	0.3185	810.23004	38.98484	49.0120
2	13.325	VB	0.3481	842.89508	36.68518	50.9880

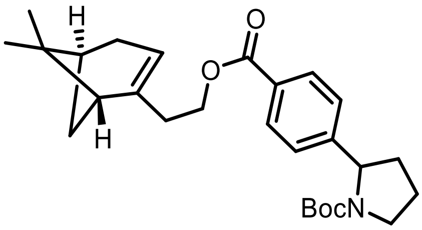

***tert*-Butyl 2-(4-((2-((1*R*,5*S*)-6,6-dimethylbicyclo[3.1.1]hept-2-en-2-yl)ethoxy)carbonyl)phenyl)pyrrolidine-1-carboxylate (31):** Colorless oil. ^{1}H NMR (500 MHz, $CDCl_3$) δ 7.95 (d, *J* = 8.4 Hz, 2H), 7.22 (d, *J* = 8.1 Hz, 2H), 5.35 (s, 1H), 5.06 – 4.68 (br m, 1H), 4.36 – 4.24 (m, 2H), 3.69 – 3.47 (br m, 2H), 2.45 – 2.31 (m, 4H), 2.29 – 2.16 (m, 2H), 2.11 (t, *J* = 5.5 Hz, 1H), 2.10 – 2.05 (m, 1H), 1.96 – 1.83 (m, 2H), 1.83 – 1.76 (m, 1H), 1.44 (br s, 3H), 1.26 (s, 3H), 1.15 (br s 6H + 1H), 0.82 (s, 3H). ^{13}C NMR

(125 MHz, $CDCl_3$) (only the major rotamer was given) δ 166.4, 154.4, 150.4, 144.3, 129.6, 128.8, 125.4, 118.9, 79.4, 63.2, 61.2, 47.1, 45.7, 40.7, 38.0, 36.0, 34.7, 31.6, 31.3, 28.1, 26.2, 23.3, 21.1. HRMS (ESI) m/z: $[M + Na]^+$ calcd. for $[C_{27}H_{37}NNaO_4]^+$: 462.2615, found 462.2609. The *d.r.* (1:1) was determined by HPLC analysis: CHIRALPAK IE-3, 10% IPA in hexane, 1.0 mL/min, 220 nm, tr_1 = 13.99 min, tr_2 = 15.05 min.

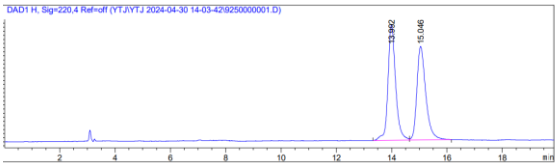

Peak #	RetTime [min]	Type	Width [min]	Area [mAU*s]	Height [mAU]	Area %
1	13.992	BV	0.2899	1079.83557	56.26939	51.4201
2	15.046	VB	0.3365	1020.19250	45.68761	48.5799

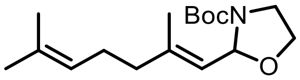

***tert*-Butyl (*E*)-2-(2,6-dimethylhepta-1,5-dien-1-yl)oxazolidine-3-carboxylate (32):** Colorless oil. ^1H NMR (500 MHz, $CDCl_3$) δ 5.71 (br s, 1H), 5.19 – 5.13 (m, 1H), 5.13 – 5.08 (m, 1H), 4.07 (ddd, J = 8.4, 6.7, 4.7 Hz, 1H), 3.92 – 3.84 (m, 1H), 3.72 – 3.63 (br m, 1H), 3.42 (dt, J = 9.9, 7.0 Hz, 1H), 2.15 – 2.08 (m, 2H), 2.07 – 2.00 (m, 2H), 1.80 (s, 3H), 1.68 (s, 3H), 1.60 (s, 3H), 1.44 (s, 9H). ^{13}C NMR (125 MHz, $CDCl_3$) δ 153.0, 141.7, 131.8, 123.8, 122.1, 84.6, 78.0, 65.4, 44.5, 39.6, 28.4, 26.2, 25.6, 17.7, 16.5. HRMS (ESI) m/z: $[M + Na]^+$ calcd. for $[C_{17}H_{29}NNaO_3]^+$: 318.2040, found 318.2040.

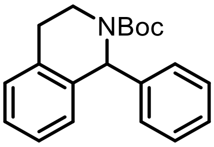

***tert*-Butyl 1-phenyl-3,4-dihydroisoquinoline-2(1*H*)-carboxylate (33):** White solid. ^1H NMR (400 MHz, $CDCl_3$) δ 7.37 – 7.22 (m, 3H), 7.25 – 7.12 (m, 5H), 7.09 – 7.01 (m, 1H), 6.55 – 6.09 (br m, 1H), 4.28 – 3.76 (br m, 1H), 3.21 (ddd, J = 13.3, 10.7, 4.4 Hz, 1H), 3.06 – 2.89 (br m, 1H), 2.84 – 2.66 (br m, 1H), 1.49 (s, 9H). For other detailed characterizations, see reference [30].

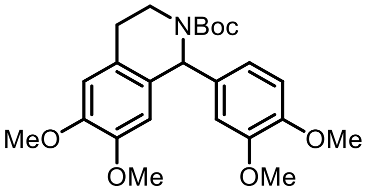

***tert*-Butyl 1-(3,4-dimethoxyphenyl)-6,7-dimethoxy-3,4-dihydroisoquinoline-2(1*H*)-carboxylate (34):** Colorless oil. ^1H NMR (400 MHz, $CDCl_3$) δ 6.94 (s, 1H), 6.71 (d, J = 8.2 Hz, 1H), 6.65 (s, 1H), 6.57 (d, J = 6.5 Hz, 1H), 6.49 (s, 1H), 6.42 – 6.04 (br m, 1H), 4.24 – 3.93 (br m, 1H), 3.87 (s, 3H), 3.83 (s, 3H), 3.81 (s, 3H), 3.74 (s, 3H), 3.03 (td, J = 12.6, 3.5 Hz, 1H), 2.97 – 2.83 (br m, 1H), 2.71 – 2.56 (br m, 1H), 1.50 (br s, 9H). For other detailed characterizations, see reference [11].

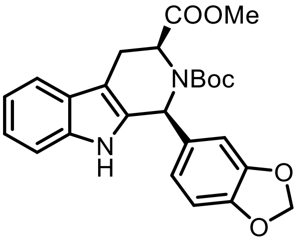

2-(*tert*-Butyl) 3-methyl (1*S*,3*S*)-1-(benzo[*d*][1,3]dioxol-5-yl)-1,3,4,9-tetrahydro-2*H*-pyrido[3,4-*b*]indole-2,3-dicarboxylate (35): Light yellow solid. ^{1}H NMR (400 MHz, $CDCl_3$) δ 7.73 (br s, 1H), 7.58 (d, *J* = 8.0 Hz, 1H), 7.24 (d, *J* = 6.9 Hz, 1H), 7.20 – 7.09 (m, 2H), 6.91 (s, 1H), 6.66 (s, 2H), 6.33 (br s, 1H), 5.90 (s, 2H), 5.36 (br s, 1H), 3.57 (d, *J* = 15.7 Hz, 1H), 3.22 (s, 3H), 3.05 (ddd, *J* = 15.7, 7.2, 1.8 Hz, 1H), 1.52 (s, 9H). The *d.r.* (> 99:1) was determined by a higher resolution of ^{1}H NMR spectrum collecting at high temperature: ^{1}H NMR (500 MHz, DMSO-d_6, 70 °C) δ 10.66 (br s, 1H), 7.51 (d, *J* = 7.8 Hz, 1H), 7.30 (d, *J* = 8.0 Hz, 1H), 7.09 (t, *J* = 7.5 Hz, 1H), 7.02 (t, *J* = 7.5 Hz, 1H), 6.80 (d, *J* = 8.0 Hz, 1H), 6.78 (d, *J* = 1.7 Hz, 1H), 6.67 (dd, *J* = 8.1, 1.7 Hz, 1H), 6.34 (s, 1H), 5.96 (s, 2H), 5.19 (d, *J* = 4.1 Hz, 1H), 3.32 (dd, *J* = 15.7, 2.8 Hz, 1H), 3.22 (s, 3H), 3.02 (dd, *J* = 15.7, 7.0 Hz, 1H), 1.49 (s, 9H). For other detailed characterizations, see reference [31].

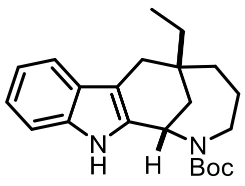

***tert*-Butyl 6-ethyl-3,4,5,6,7,12-hexahydro-1,6-methanoazonino[3,4-*b*]indole-2(1*H*)-carboxylate (36):** Light yellow solid (*d.r.* > 99:1). ^{1}H NMR (500 MHz, $CDCl_3$) δ 8.86 – 7.93 (br m, 1H), 7.48 (d, *J* = 7.7 Hz, 1H), 7.31 (d, *J* = 8.1 Hz, 1H), 7.15 (t, *J* = 7.4 Hz, 1H), 7.07 (t, *J* = 7.3 Hz, 1H), 5.48 – 5.32 (m, 1H), 3.67 – 3.55 (m, 1H), 3.40 – 3.20 (m, 1H), 2.69 – 2.51 (m, 2H), 2.14 – 1.99 (m, 2H), 1.77 – 1.68 (m, 1H), 1.61 – 1.54 (m, 3H), 1.50 (s, 9H), 1.48 – 1.44 (m, 2H), 0.99 (t, *J* = 7.5 Hz, 3H). ^{13}C NMR (125 MHz, $CDCl_3$) (only the major rotamer was given) δ 156.7, 136.2, 135.2, 126.7, 121.8, 118.9, 118.4, 111.1, 111.0, 79.9, 47.5, 46.6, 41.6, 38.7, 36.9, 35.0, 32.7, 28.6, 24.8, 8.2. HRMS (ESI) m/z: $[M + H]^+$ calcd. for $[C_{22}H_{31}N_2O_2]^+$: 355.2380, found 355.2379.

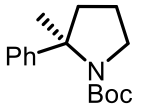

***tert*-Butyl (*R*)-2-methyl-2-phenylpyrrolidine-1-carboxylate (37):** Colorless oil. ^{1}H NMR (400 MHz, $CDCl_3$) δ 7.33 – 7.14 (m, 5H), 3.76 – 3.55 (m, 2H), 2.13 – 1.98 (m, 2H), 1.92 – 1.80 (m, 2H), 1.75 (s, 3H), 1.45 and 1.12 (2s, 2H + 7H). For other detailed characterizations, see reference [8].

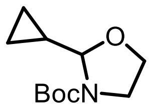

***tert*-Butyl 2-cyclopropyloxazolidine-3-carboxylate (38):** Colorless oil. ^{1}H NMR (500 MHz, $CDCl_3$) δ 4.81 (s, 1H), 4.07 (ddd, *J* = 8.2, 6.7, 5.3 Hz, 1H), 3.91 – 3.84 (m, 1H), 3.70 (br s, 1H), 3.36 (dt, *J* = 10.0, 6.8 Hz, 1H), 1.48 (s, 9H), 1.17 – 1.06 (m, 1H), 0.59 – 0.44 (m, 3H), 0.39 (dt, *J* = 10.3, 4.9 Hz, 1H). ^{13}C NMR (125 MHz, $CDCl_3$) δ 153.43, 90.93, 80.18, 65.39, 44.78, 28.42, 14.67, 2.38, 0.97. HRMS (ESI) m/z: $[M + Na]^+$ calcd. for $[C_{11}H_{19}NNaO_3]^+$: 236.1257, found 236.1252.

7. Mechanistic study

A model catalytic reaction, using complex **1** and **3**, carried out at 100 °C was monitored by UV/Vis

spectroscopy (Figure S3A), together with the time course experiments by ^{1}H NMR spectroscopy (Figure S4A). The cyclic voltammogram of **1** was also measured (Figure S5). The reaction of **1** with **3** or 4-phenylbutan-1-amine (**II**, a by-product derived from **3**) can produce Fe^{III}(Cor) species, as shown by UV/Vis spectroscopic analysis (Figure S6).

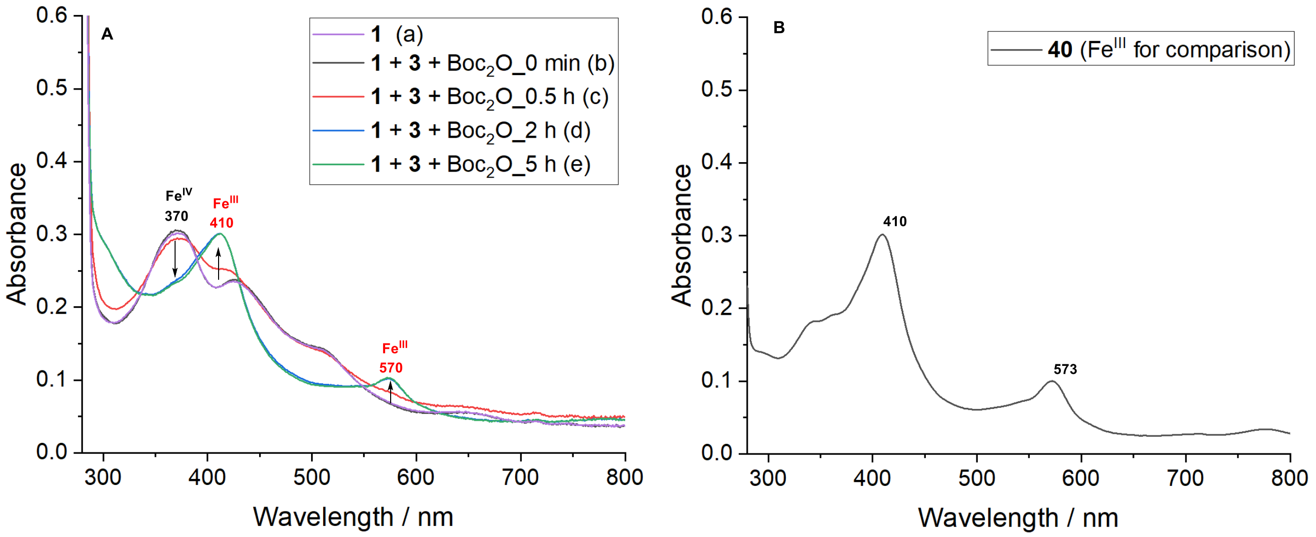

Figure S3. (A) UV/Vis spectra for detecting Fe species in the catalytic reaction (in toluene; under argon). (B) UV/Vis spectrum of Fe^{III} complex **40**.

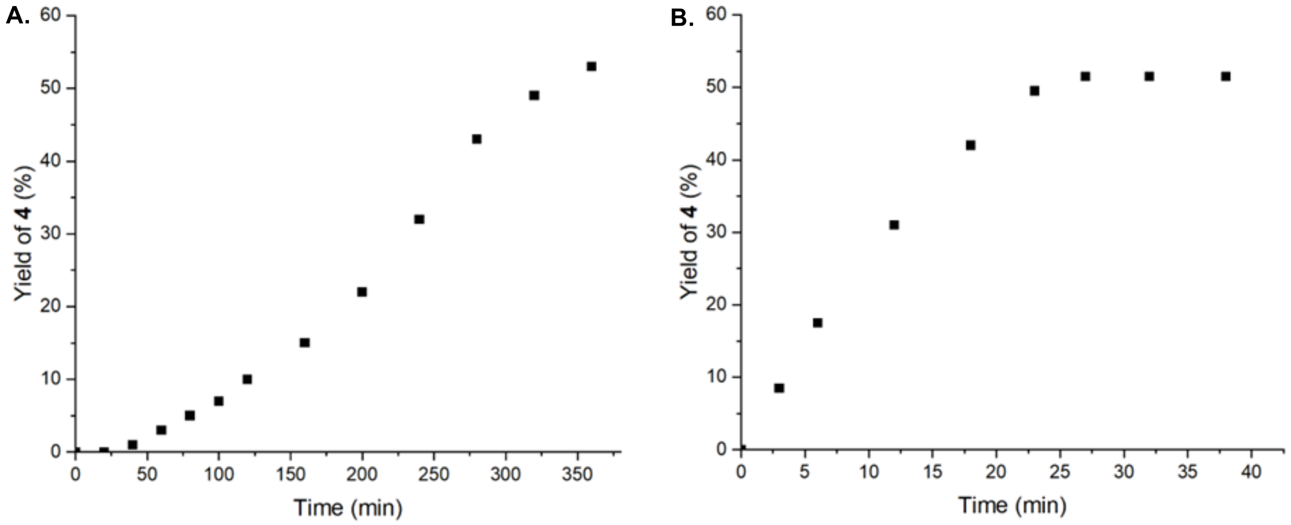

Figure S4. Time-course experiments catalyzed by (A) **1** or (B) **2** ([**cat.**] = 1.0 mM, [**3**]$_0$ = 100 mM, [Boc_2O] = 200 mM, 100 °C).

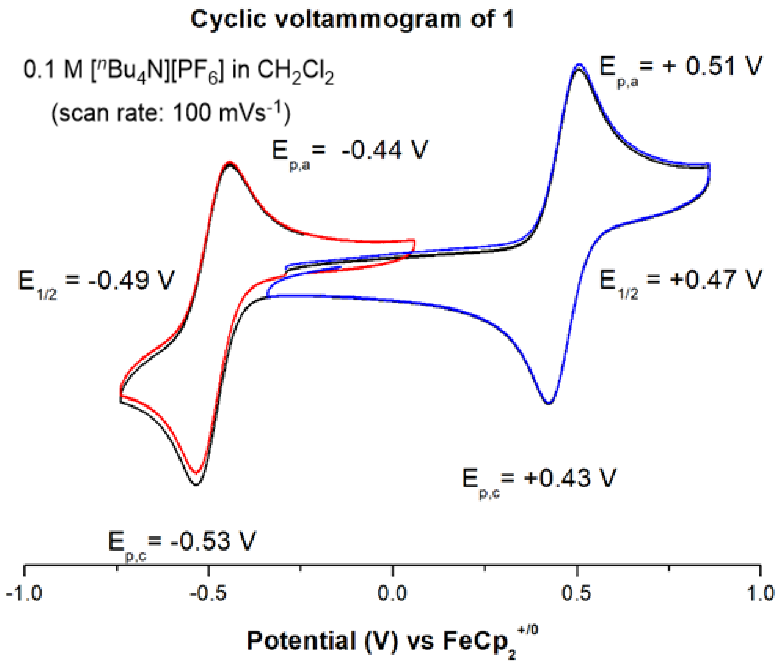

Figure S5. Cyclic voltammogram (CV) of **1**.

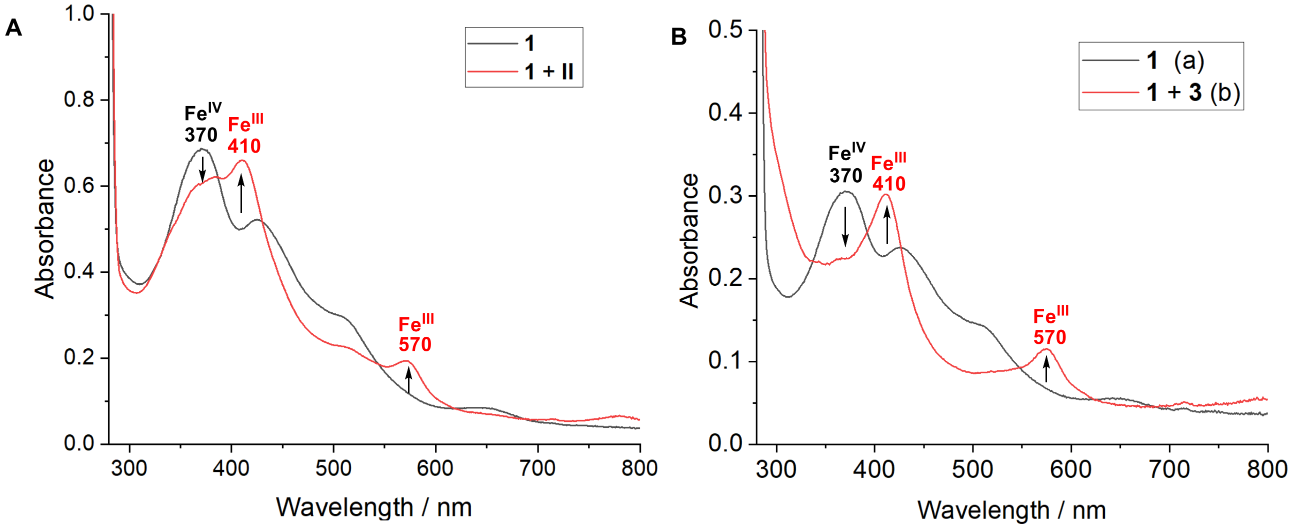

Figure S6. UV/Vis analysis for (A) reaction of **1** with 4-phenylbutan-1-amine (**II**) at room temperature and (B) reaction of **1** with **3** after heating.

7.1 Kinetic isotope effect (KIE)

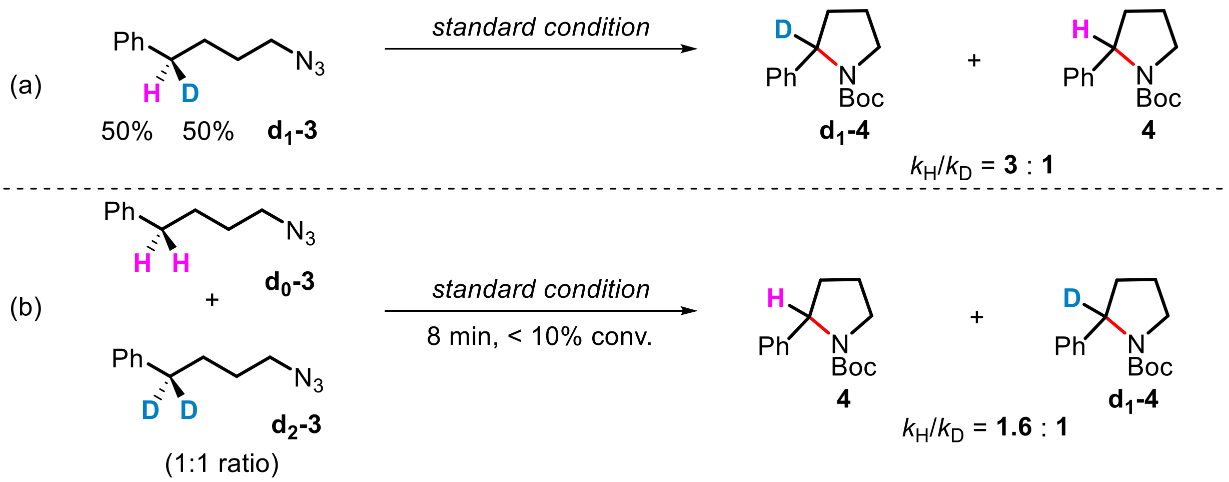

Scheme S1. (a) Intramolecular KIE; (b) intermolecular KIE.

(a): Monodeuterated azide **d₁-3** was treated under the standard conditions. The ratio of k_H/k_D was determined via the yield ratio of (**d₁-4**)/**4** by ^{1}H NMR integration of the purified product. Figure S7 shows the ^{1}H NMR analysis results. (b): A 1:1 ratio of **d₀-3** and **d₂-3** were treated under the standard conditions for 8 min (<10% conversion of the starting azides). The ratio of k_H/k_D was determined via the yield ratio of **4**/(**d₁-4**) by ^{1}H NMR integration of the crude spectrum. Figure S8 shows the ^{1}H NMR analysis results.

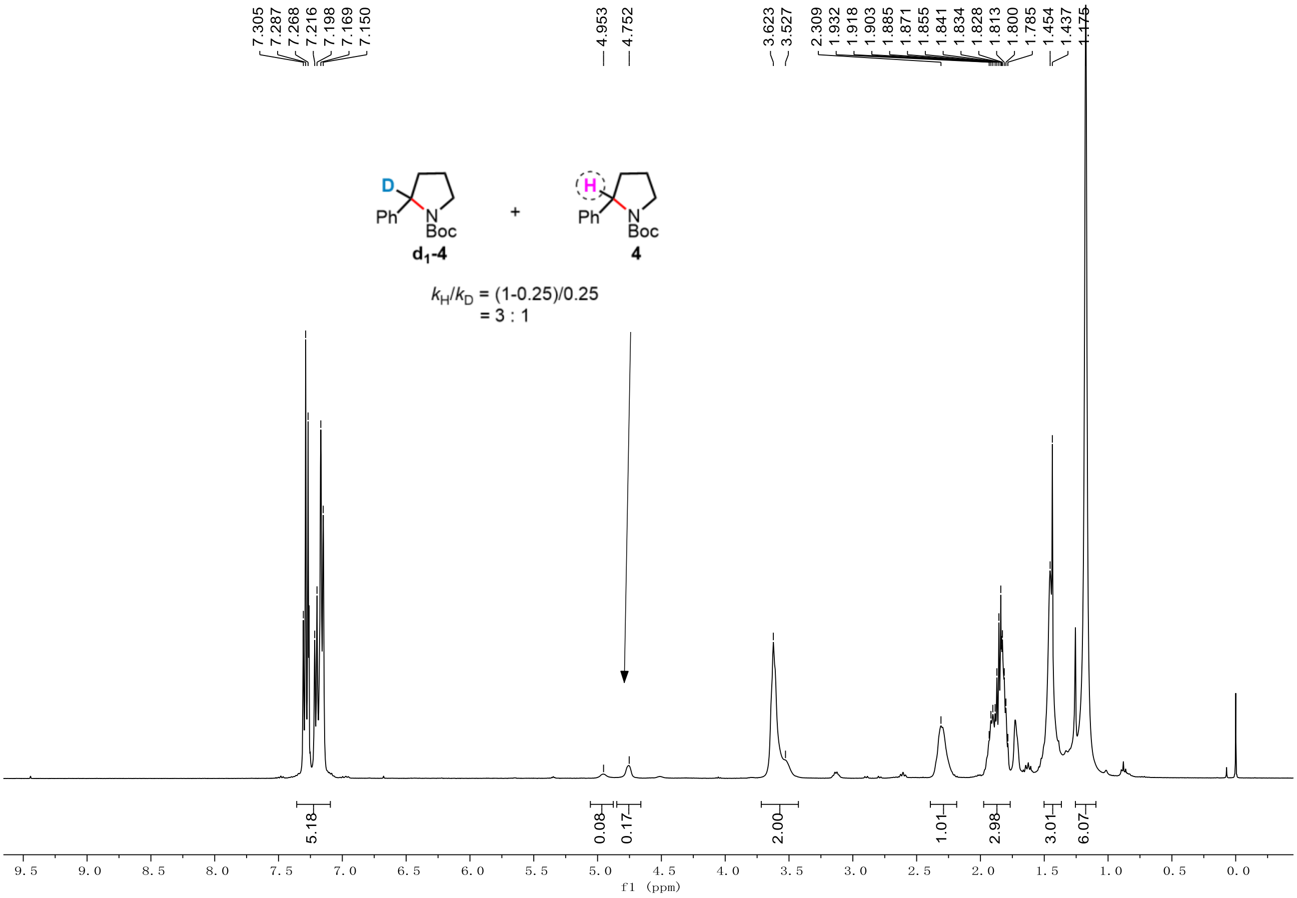

Figure S7. ^{1}H NMR spectrum for intramolecular KIE.

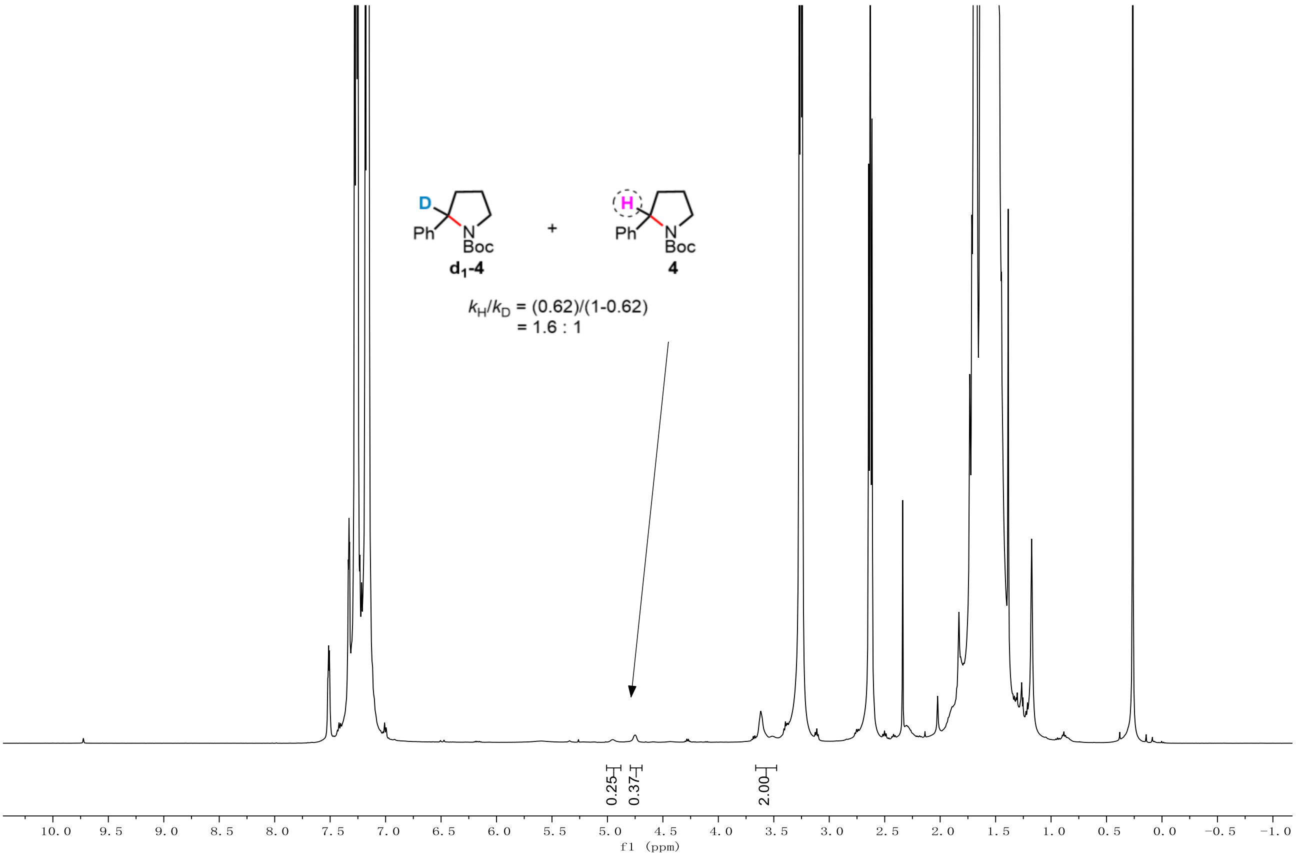

Figure S8. ^{1}H NMR spectrum for intermolecular KIE.

7.2 Analysis of stereochemistry

(*S*)-(5-Azidopentan-2-yl)benzene (**S-37**) was treated under the standard catalytic conditions, and the product was determined to be 94% ee by chiral HPLC analysis (CHIRALCEL OJ-H, 1% IPA in hexane, 0.5 mL/min, 220 nm, tr (minor) = 10.28 min, tr (major) = 11.83 min).

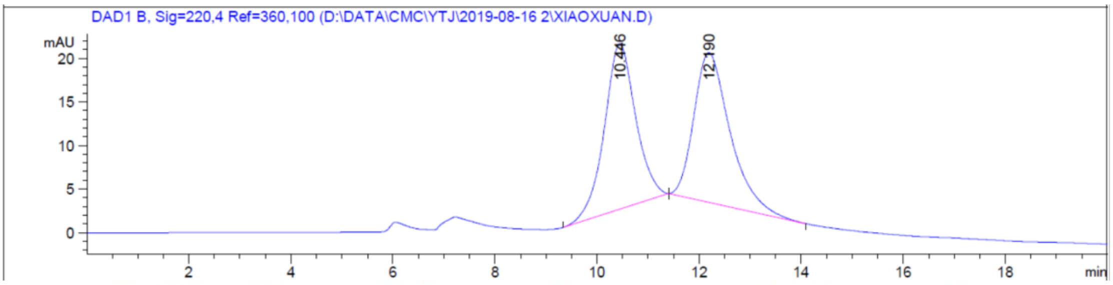

Peak #	RetTime [min]	Type	Width [min]	Area [mAU*s]	Height [mAU]	Area %
1	10.446	BB	0.6341	811.26819	19.00231	48.8866
2	12.190	BBA	0.7400	848.22205	17.17933	51.1134

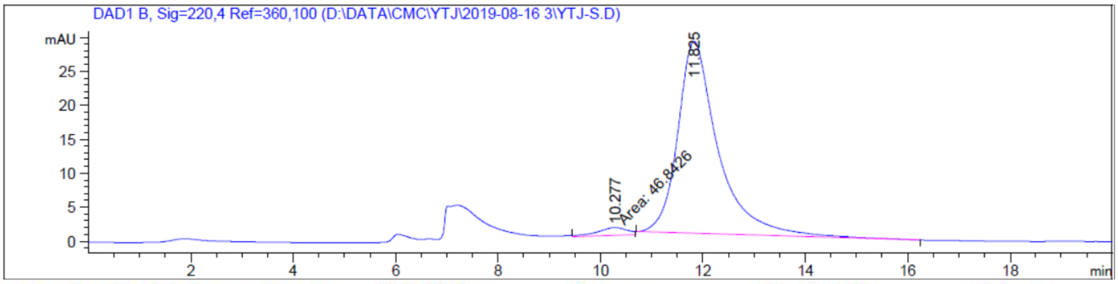

Peak #	RetTime [min]	Type	Width [min]	Area [mAU*s]	Height [mAU]	Area %
1	10.277	MM	0.6853	46.84262	1.13928	2.9238
2	11.825	BB	0.7976	1555.25403	28.27812	97.0762

7.3 Binding constant

The binding constants (K_{st}) of **2** with $Ph(CH_2)_4N_3$ (**3**) were examined as follows: $\{[Fe(T\textit{p}\text{-OMePC})]\}_o$ = 13.02 µM, $[Ph(CH_2)_4N_3]_o$ = 0.23 mM, 0.38 mM, 0.53 mM or 0.69 mM.

Given that $[RN_3]_o >> [Fe(Cor)]_o$, hence $[RN_3] \approx [RN_3]_o$. Based on Benesi–Hildebrand plot, the slope can be used to calculate K_{st}. Wavelength at 336 nm was used to monitor the spectral change, so as to minimize the errors originated from the wavelength close to isosbestic point.

Consider the following equilibrium:

$[Fe(T\textit{p}\text{-OMePC})] + RN_3 \rightleftharpoons [Fe(T\textit{p}\text{-OMePC})(RN_3)]$, where R = $(CH_2)_4Ph$

$$\frac{1}{\Delta A_{336}} = \left(\frac{1}{b\Delta\varepsilon[Fe(Tp-OMePC)]_o K_{st}}\right)\frac{1}{[RN_3]_o} + \frac{1}{b\Delta\varepsilon[Fe(Tp-OMePC)]_o}\text{, where b} = 1\text{ cm}$$

R = $(CH_2)_4$Ph:

$$\frac{1}{b\Delta\varepsilon[Fe(Tp-OMePC)]_o} = 5.86325 \Rightarrow \Delta\varepsilon = 13097\ M^{-1}\ cm^{-1}$$

$$\frac{1}{b\Delta\varepsilon[Fe(Tp-OMePC)]_o K_{st}} = 0.01268$$

$$K_{st}((CH_2)_4Ph) = 4.62 \times 10^2\ (\pm 0.47 \times 10^2)\ M^{-1}\ \text{at 298 K}$$

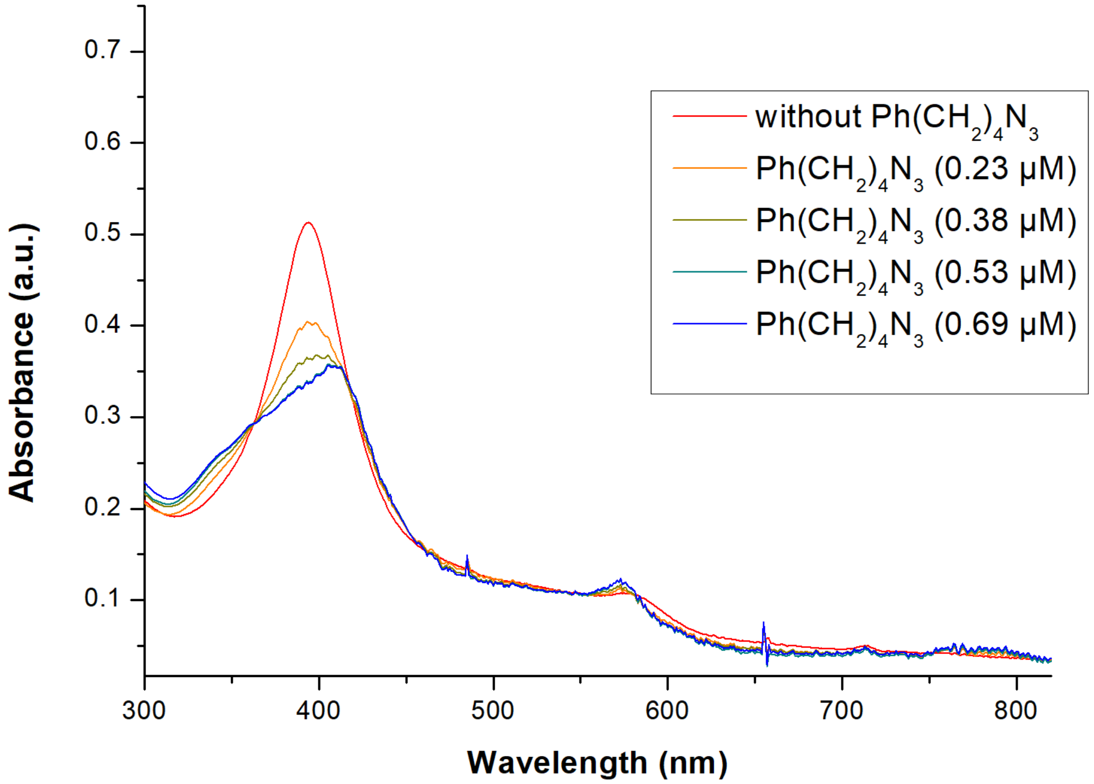

Figure S9. UV/Vis spectra of “**2** + **3**” for binding constant measurement.

7.4 Synthesis and characterization of Fe^V-alkylimido complex 41

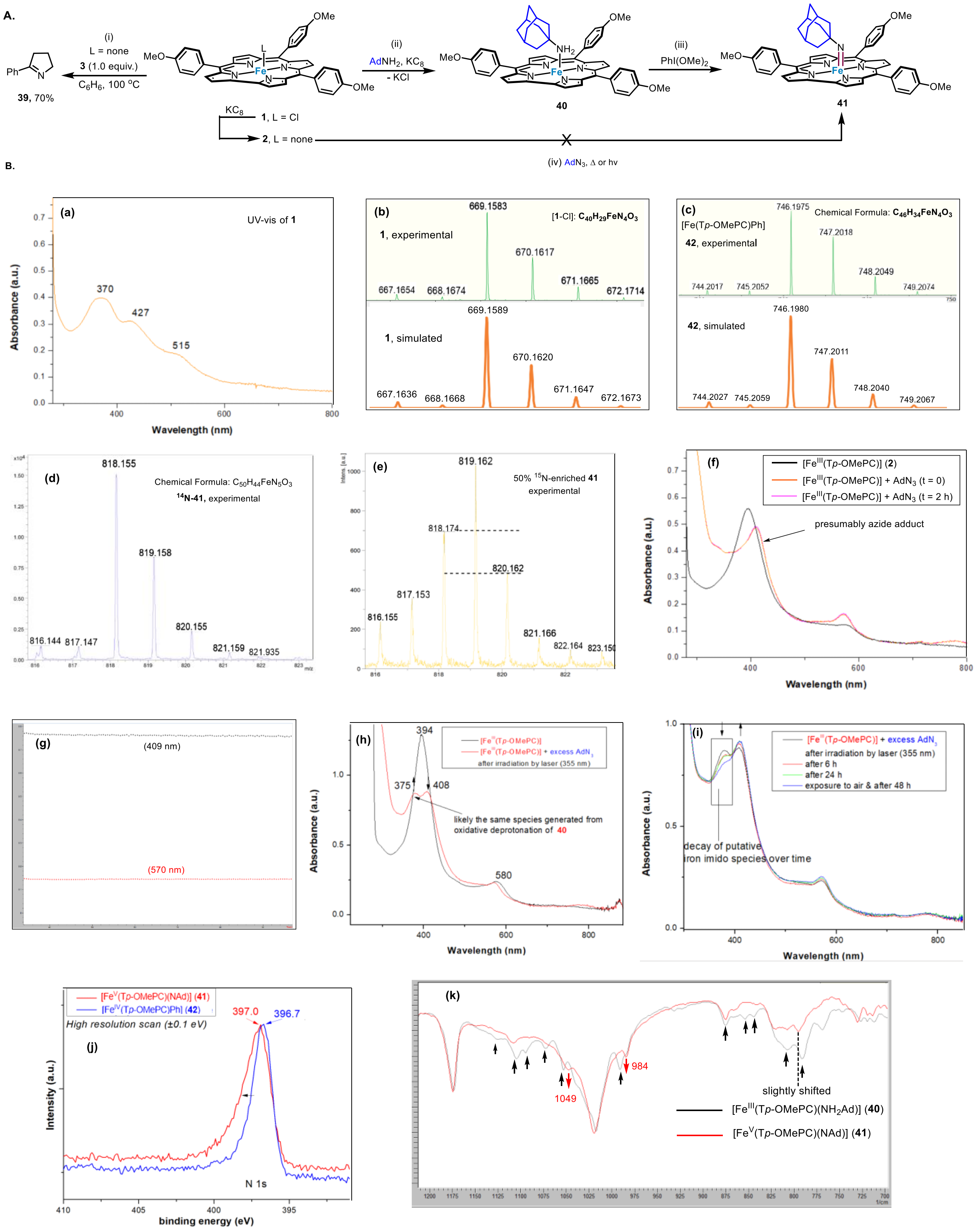

Figure S10. A. Synthesis of Fe^V-alkylimido complex **41**. B. Spectroscopic characterization of related species (others see the main text). (a) UV/Vis spectrum of **1** in benzene. (b)&(c) HR

ESI-MS$^+$ spectra of **1** and **42** (experimental and simulated isotope patterns). (d)&(e) MALDI-TOF MS of ^{14}N-**41** and ^{14}N-**41** +^{15}N-**41** (1:1 ratio). (f) UV/Vis spectra of “**2** + AdN_3” in benzene. (g) Stability test for “**2** + AdN_3” in benzene. (h)&(i) UV/Vis spectra of “**2** + AdN_3” with irradiation in benzene. (j) High resolution N 1s XP spectra of **41** and **42** (innocent corrole ligand). (k) FTIR spectra (KBr round plates) of **40** and **41**.

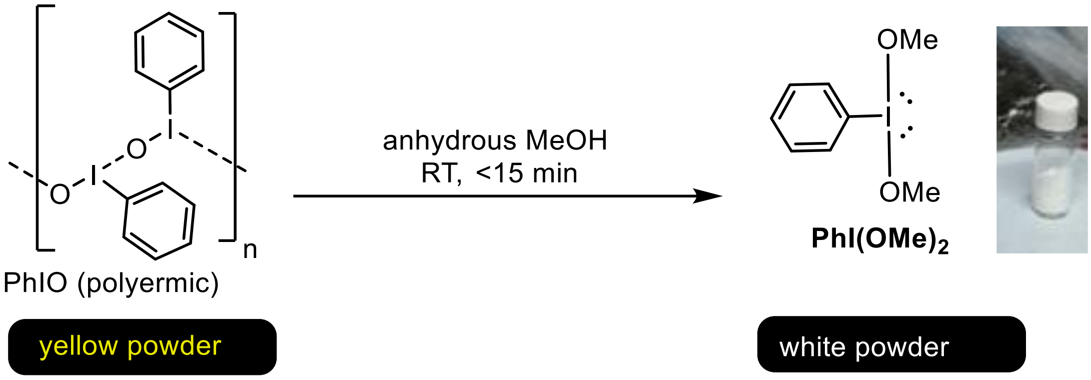

The reported procedure was used with some modifications.[32] In a moisture- and oxygen-free (<1 ppm) glovebox, iodosylbenzene (2.00 g, 9.09 mmol; PhIO) was added to a round-bottom flask containing 20 mL of anhydrous MeOH for no more than 15 min. When the reaction mixture became clear, it was then evaporated to dryness and extracted with dry benzene (10 mL × 4 times). A white solid was obtained after removal of benzene solvent (yield: 40–60%).

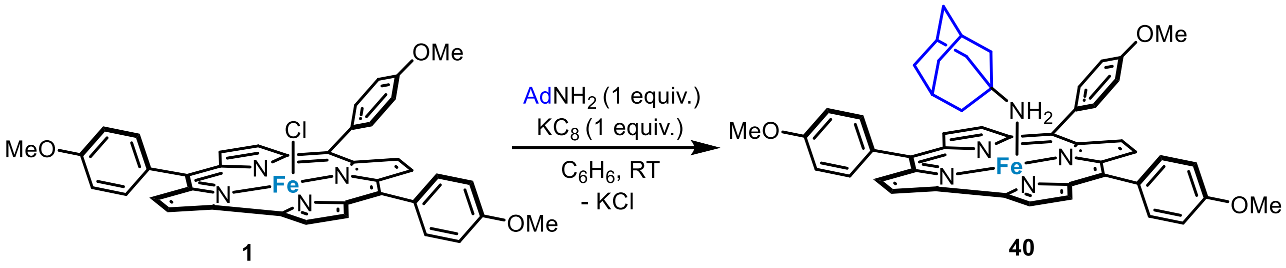

To an oven-dried seal tube, complex **1** (20 mg, 0.0284 mmol), $AdNH_2$ (4.30 mg, 0.0284 mmol) and KC_8 (3.8 mg, 0.0284 mmol; Pyrex® spinbar was used) were dissolved with anhydrous benzene (4 mL) and stirred for 24 h. The reaction progress was monitored using UV/Vis spectroscopy until the absorption bands of **1** disappeared. After 24 h, the reaction mixture was filtered using a plastic syringe equipped with PTFE filter (0.45 µm). The filtrate was evaporated to dryness. 50% ^{15}N-enriched **40** was prepared similarly from ^{15}N-enriched $AdNH_2$ which was synthesized from 1-adamantanecarboxylic acid (AdCOOH) and $Na^{15}N_3$ according to reported procedures.[33]

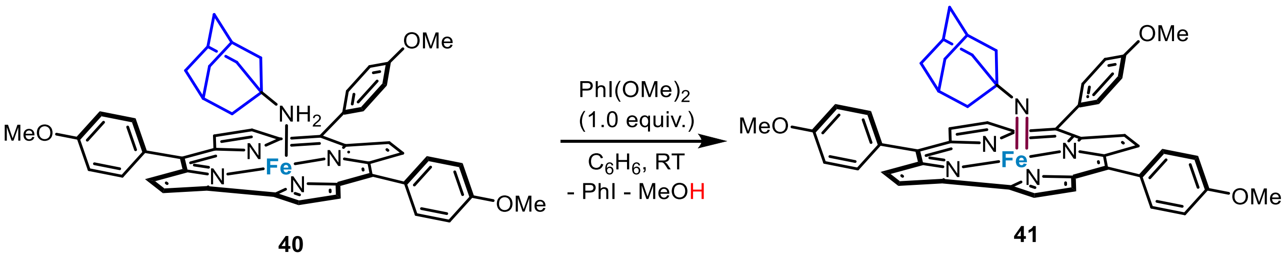

To an oven-dried sealed tube, complex **40** (10 mg, 0.0122 mmol) was dissolved with anhydrous

benzene (2 mL) and mixed with a benzene solution (2 mL) of $PhI(OMe)_2$ (1 equiv.) upon rapid stirring. The reaction mixture became yellowish immediately. Reaction progress was monitored using UV/Vis spectroscopy until no further change was observed. Typically, the reaction mixture was stirred for no more than 5 min and evaporated to dryness. The reaction mixture was washed with anhydrous hexane to remove any residual $PhI(OMe)_2$ (which is soluble in non-polar solvent).

[Fe(T*p*-OMePC)(NH_2Ad)] (**40**): Yield: 92%. UV/Vis (Benzene): λ_{max} (ε x 10^{-4}): 410 (5.56), 570 (1.48) nm; HRMS (ESI) ($C_{50}H_{46}FeN_5O_3$): m/z calc 820.2950, found: 820.2956; X-band EPR (frozen benzene, 100 K): g = 3.73, 1.90; effective magnetic moment (Evans method, ^{1}H NMR in C_6D_6 at 298 K): 3.87 μ_B; elemental analysis for $C_{50}H_{46}FeN_5O_3$•$0.5H_2O$, calc (found): C, 72.37 (72.49); H, 5.71 (5.71); N, 8.44 (7.95).

[Fe(T*p*-OMePC)(NAd)](**41**): Yield: 94%. HRMS (ESI) ($C_{50}H_{44}FeN_5O_3$): m/z calc 818.2794, found: 818.2772; X-band EPR (frozen benzene, 100 K): g = 2.05, 2.04, 1.95; effective magnetic moment (Evans method, ^{1}H NMR in C_6D_6 at 298 K): 1.85-1.88 μ_B; XPS (±0.1 eV): 708.5 (Fe $2p_{3/2}$), 721.6 (Fe $2p_{1/2}$) eV; UV/Vis (benzene): λ_{max} (ε x 10^{-4}): 375 (5.14), 418 (4.50), 630 (0.53) nm; Raman (solvent: DCM): 912 (Fe=^{15}NAd), 932 (Fe=^{14}NAd) cm^{-1}.

Complex [Fe(T*p*-OMePC)Ph](**42**), used in the XPS study, was prepared by the reported method.[34]

X-band EPR

In a glovebox, 1 mg of **40** was dissolved in 0.2 mL of dry and deaerated benzene. The solution was transferred to an oven-dried quartz EPR tube. The EPR tube was sealed with a plastic cap and then the cap was wrapped with parafilm. Once the tube was taken out from the antechamber, the solution was immediately frozen by immersing into a liquid nitrogen bath. The frost on the tube was gently removed using a tissue paper. After that, the tube was placed into the sample holder of the EPR instrument (pre-equilibrated at 100 K). The scan width was 200 to 6200 Gauss. In a similar way, 1.5 mg of freshly prepared **41** was dissolved in 0.2 mL of benzene and used to record the EPR spectrum of **41** at the same temperature. The EPR spectral simulation was done using the EasySpin toolbox.[35]

XPS

X-ray photoelectron spectroscopy was performed on a PHI 5000 VersaProbe III Photoelectron Spectrometer (manufactured by ULVAC-PHI Inc.) with a monochromatic AI Kα X-ray source. The peak energy position was checked by using polished fine grain Au and Cu as reference. The air-sensitive powder samples were mounted on a 25 mm diameter sample holder by placing them on pieces of carbon tapes, followed by pressing with spatula. The sample holder was then loaded to a transfer vessel in a glovebox filled with high purity argon. The sample holder was transferred to the analysis chamber without exposing to ambient air. The survey scans were recorded at 280 eV pass energy by 1 eV step with X-ray source setting of 100 micrometer, 25 W and 15 kV. For the high-resolution scans for each element, they were recorded at 112 eV pass energy by 0.1 eV step with X-ray source setting of 200 micrometer, 50 W and 15 kV.

Resonance Raman (RR) spectroscopy

The experimental setup of RR spectroscopy was described in detail previously.[36] The 416 nm laser pulse with an intensity at around 2 mW was produced from the first Stokes hydrogen Raman-shifted laser line from the third harmonic (355 nm) of a Nd:YAG nanosecond pulsed laser system. The Raman scattered signal was collected using a backscattering geometry and observed by a liquid-nitrogen-cooled charge-coupled device (CCD) detector. The Raman bands of MeCN were used to calibrate the Raman shifts with an estimated uncertainty of 5 cm^{-1}. The Raman signal was acquired for 30 s by the CCD before reading out to the computer, and 20 scans of the signal were accumulated to produce each resonance Raman spectrum. The sample solutions were prepared in DCM to have an absorbance of ~1.5 at 416 nm in a 2 mm path-length cuvette.

7.5 Reactivity studies of Fe^{V}-imido complex 41

Intermolecular H atom transfer (HAA) of 41 to 40

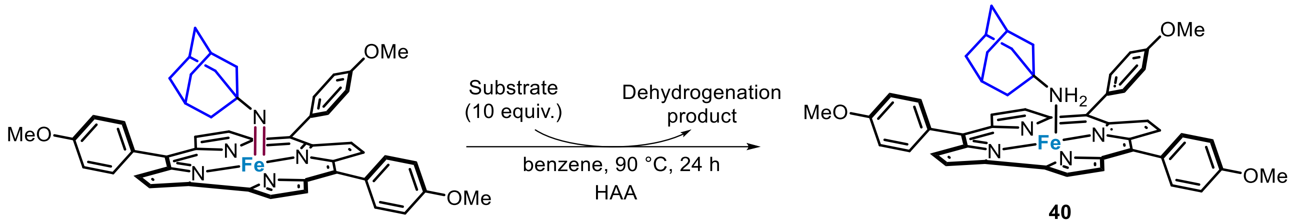

Stoichiometric reactions of **41**-mediated hydrogen atom abstraction were studied as follows: To an oven-dried seal tube, **41** (0.01222 mmol) and substrate (e.g. DHA, MCH, DHN, **45**, **47** or **50**; 0.1222 mmol) were dissolved in 2 mL of dry benzene (or benzene-d_6 for substrates MCH, **45** and **47**). The sealed tube was placed into a pre-equilibrated oil bath (90 ºC) if heating was needed and stirred for 24 h (48 h for non-heating reaction). After that, the reaction mixture was evaporated to dryness and extracted with hexane (for reactions performed in benzene-d_6, internal standard was added and recorded ^{1}H NMR spectra directly). The extract was evaporated to dryness and re-dissolved with suitable deuterated solvent ($CDCl_3$: xanthene, fluorene, DHA or benzene-d_6: MCH, DHN, **45**, **47**) for ^{1}H NMR spectroscopy (internal standard: 1,3,5-trimethoxybenzene for % yield determination).

7.6 KIE of intermolecular HAA of 41 with DHA and d_4-DHA

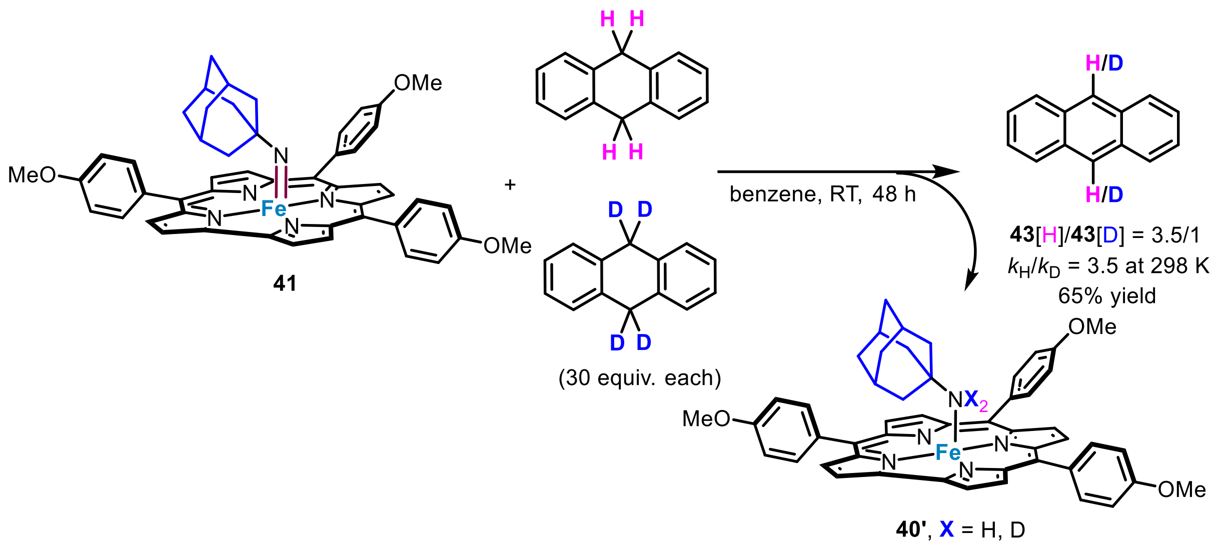

The KIE for **41**-mediated C–H abstraction reaction was studied as follows: To an oven-dried seal tube, **41**, DHA and d_4-DHA in a mole ratio of 1: 30: 30 (scale: 0.01222 mmol of **41**) were dissolved with 2 mL of dry benzene. After stirring for 48 h, the reaction mixture was evaporated to dryness and extracted with hexane. The hexane solution was again evaporated to dryness and re-dissolved with benzene-d_6. The product ratio (KIE = [anthracene]/[anthracene-d_2]) was analyzed using ^{1}H NMR spectroscopy. Below are shown the ^{1}H NMR analysis results.

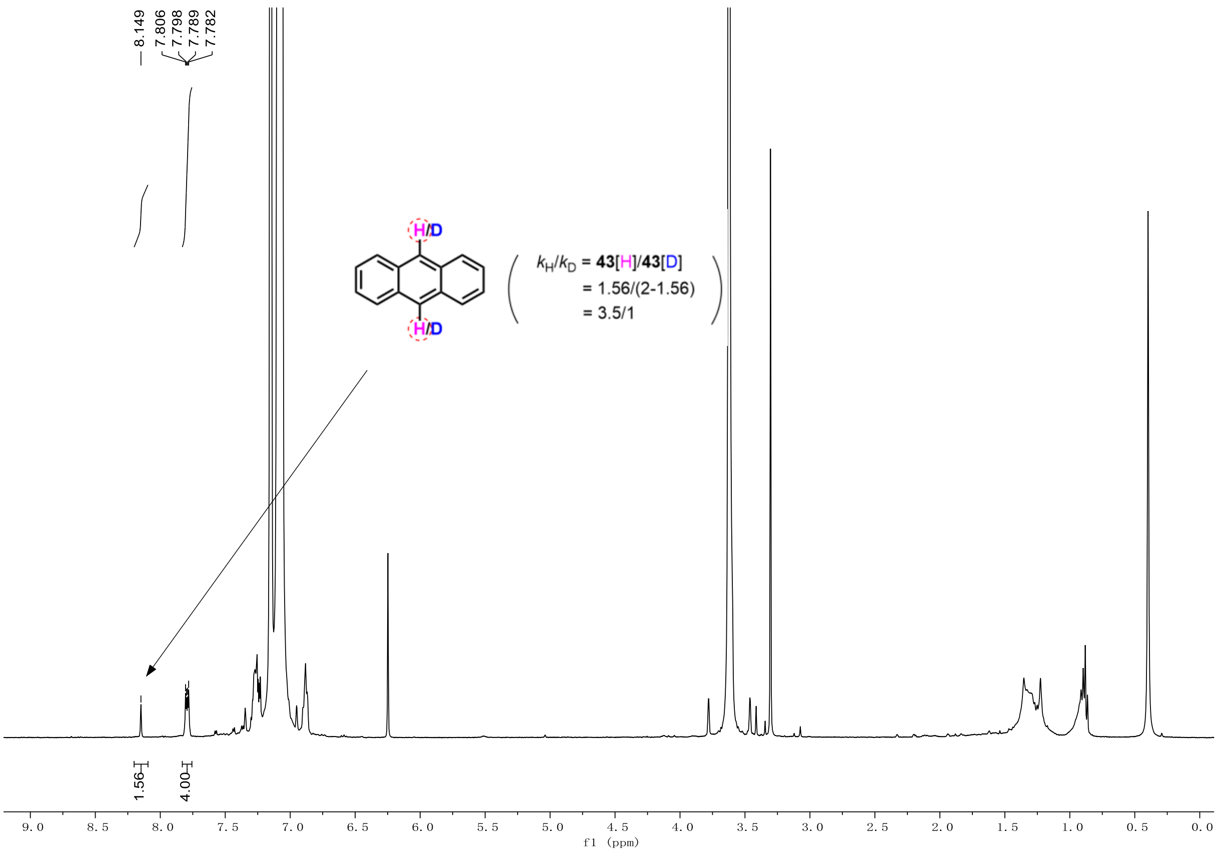

Figure S11. ^{1}H NMR spectrum for KIE of intermolecular HAA.

7.7 HAA of 41 with 50

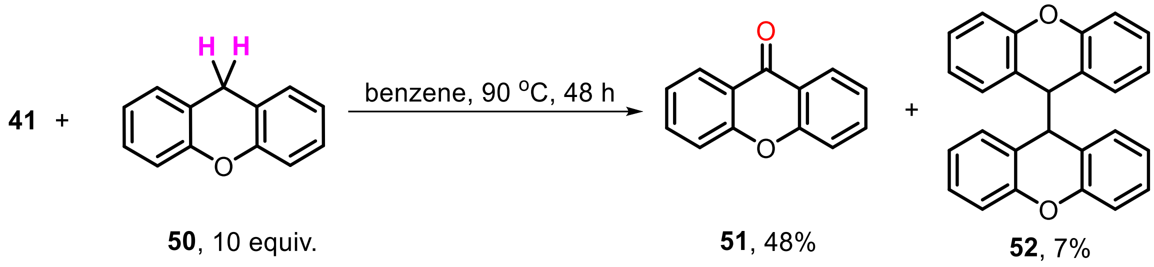

For detailed procedures, see the above mentioned stochiometric HAA reactions. The yield presented here is the ^{1}H NMR yield. The ^{1}H NMR spectrum of isolated **51** is consistent with that of a commercial sample, and the NMR data of **52** are as follows:

***9H*,9'*H*-9,9'-bixanthene (52):** White solid. ^{1}H NMR (400 MHz, $CDCl_3$) δ 7.20 (t, J = 7.7 Hz, 4H), 6.93 (t, J = 7.4 Hz, 4H), 6.86 (d, J = 8.1 Hz, 4H), 6.66 (d, J = 7.5 Hz, 4H), 4.20 (s, 2H). ^{13}C NMR (100 MHz, $CDCl_3$) δ 153.0, 129.2, 128.1, 122.6, 121.9, 115.9, 49.5. For other characterizations, see reference [37].

7.8 Stoichiometric or catalytic approach towards intermolecular aziridination

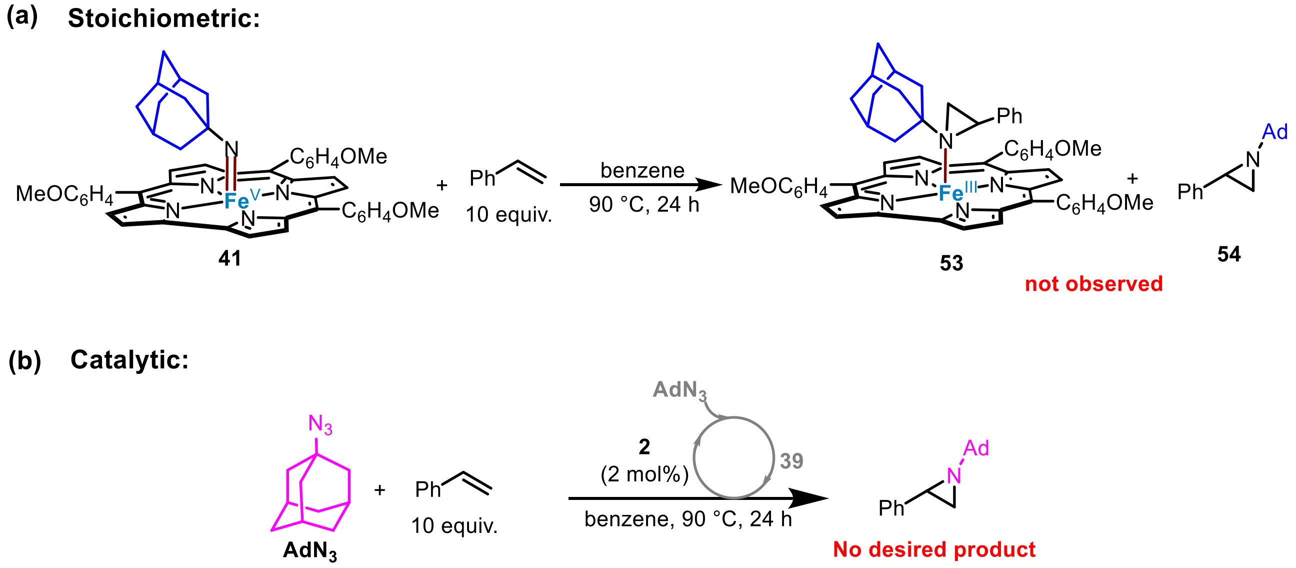

Scheme S2. Attempts on intermolecular aziridination.

The stochiometric reaction of **41** towards aziridination was attempted under the following conditions: To an oven-dried seal tube, **41** (0.01222 mmol) and styrene (0.1222 mmol) were dissolved in 2 mL of dry benzene and placed into a hot oil bath pre-equilibrated at 90 ºC. After stirring for 24 h, the reaction mixture was evaporated to dryness and extracted with dry hexane. The hexane solution was analyzed using ^{1}H NMR spectroscopy after evaporation to dryness and re-dissolution with $CDCl_3$. The portion that did not dissolve in hexane (mostly Fe corrole species) was analyzed using ESI-MS. In both portions, neither Fe-aziridine complex nor free aziridine was observed. Similarly, the **2**-catalyzed aziridination reaction of AdN_3 and styrene was attempted except that **2**, AdN_3 and

styrene were added in a 1: 50: 500 ratio (scale: 0.004 mmol of **2**). The crude reaction mixture was directly analyzed using ^{1}H NMR spectroscopy (solvent: $CDCl_3$). Like the stoichiometric scenario, no aziridine product was found.

8. Single crystal X-ray crystallography

Crystallographic data have been deposited in the Cambridge Crystallographic Data Centre database (CCDC) and the deposition numbers are 2246211 (for **1**) and 2246212 (for **36**). Copies of the data can be obtained free of charge from the CCDC at www.ccdc.cam.ac.uk.

Table S2. Crystallographic data and structure refinement parameters.

Compound	**1**	**36**
Empirical formula	$C_{41}H_{31}Cl_3FeN_4O_3$ (**1**•DCM)	$C_{22}H_{30}N_2O_2$
Formula weight	789.90 (**1**•DCM)	354.48
Temperature/K	100.0	
Crystal system	monoclinic	triclinic
Space group	Cc	P-1
a/Å	17.6759(7)	10.6572(7)
b/Å	16.1433(6)	10.9033(7)
c/Å	15.1661(9)	17.8299(12)
α/°	90	79.794(2)
β/°	124.7700(10)	81.063(2)
γ/°	90	88.298(2)
Volume/Å^3	3554.9(3)	2014.3(2)
Z	4	4
ρ_{calc}g/cm^3	1.476	1.169
μ/mm^{-1}	0.697	0.075
F(000)	1624.0	768.0
Crystal size/mm^3	0.35 × 0.31 × 0.29	0.20 × 0.15 × 0.15
Radiation	MoKα (λ = 0.71073)	
Reflections collected	26187	77289
Data/restraints/parameters	8831/2/474	9281/0/477
Goodness-of-fit on F^2	1.045	1.034
Final R indexes [I>=2σ (I)]	R_1 = 0.0338, wR_2 = 0.0739	R_1 = 0.0470, wR_2 = 0.1104
Final R indexes [all data]	R_1 = 0.0394, wR_2 = 0.0766	R_1 = 0.0684, wR_2 = 0.1216
Largest diff. peak/hole / e Å^{-3}	0.66/-0.71	0.34/-0.31

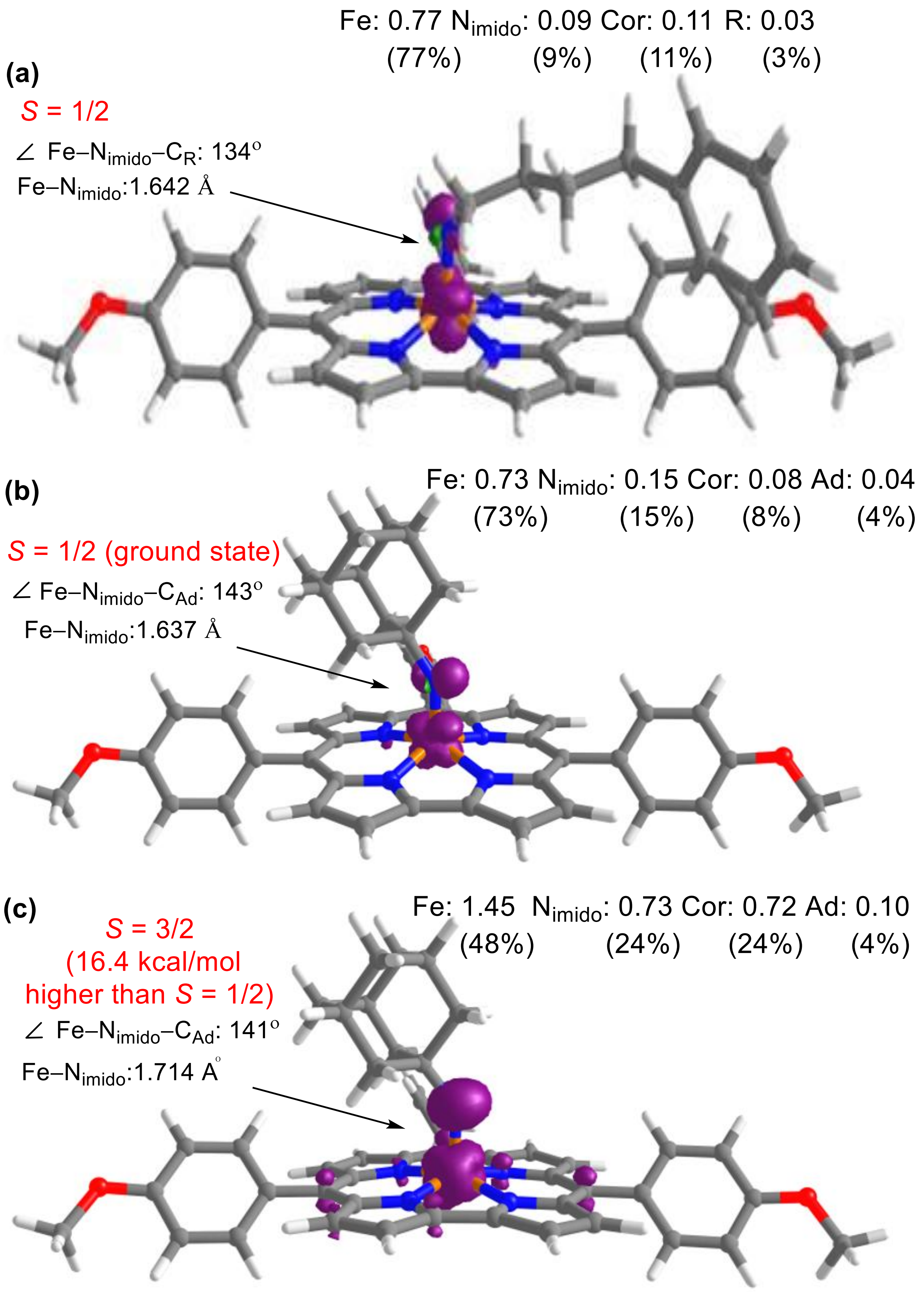

Figure S12. DFT-calculations on the structural information and spin density distribution (contour value: 0.01) of ^{2}**Int2** (a), ^{2}**41** (S = 1/2) (b) and ^{4}**41** (S = 3/2) (c).

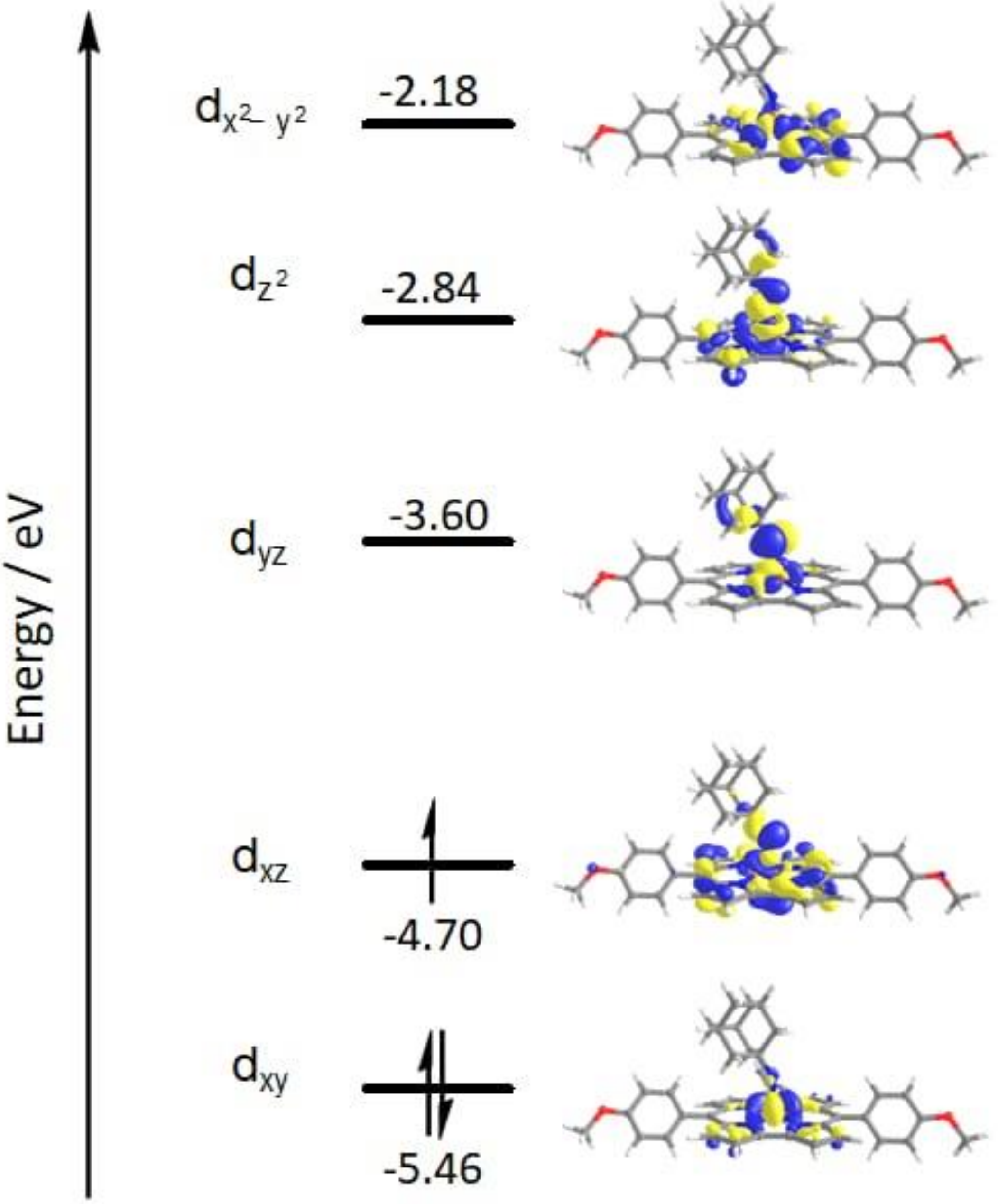

Figure S13. DFT-calculated molecular orbitals of **^{2}41** (*S* = 1/2).

9. Computational details

In this work, all DFT calculations were performed using GAUSSIAN09.[38] Geometries were optimized using the BP86 functional[39] in combination with the def2-SVP basis set. Dispersion correction with Grimme's D3 version was employed.[40] Solvent effect was considered through the polarizable continuum model (PCM).[41] Frequency calculations were carried out for optimized structures to verify the stationary points or transition states at the same level. Single-point energies were refined with the triple-ζ basis set def2-TZVP using the M06L functional.[42] Spin density plots were generated using Multiwfn.[43]

Cartesian Coordinates for DFT-calculated structures

Structure of ^{2}41

Fe	-0.300803000	0.403664000	-0.287044000
N	0.497873000	0.438971000	1.477529000
N	-0.859108000	-1.416929000	0.117477000
C	-1.021317000	-2.227535000	3.794305000
C	-1.132193000	-2.059477000	1.301641000
C	-0.686213000	-1.522247000	2.529327000
C	0.095122000	-0.335806000	2.572961000
C	-0.644464000	-3.572203000	3.994887000
C	1.356207000	1.379836000	3.402583000
H	1.895170000	2.075284000	4.054861000
C	-1.362968000	-2.157270000	-0.929365000
C	0.655077000	0.247315000	3.769149000
H	0.542566000	-0.170926000	4.775475000
C	-1.738012000	-1.580186000	4.831576000
C	-0.959884000	-4.256968000	5.180460000
H	-0.636080000	-5.300201000	5.299309000
C	-1.866158000	-3.264823000	0.988934000
H	-2.232930000	-3.991164000	1.723432000
C	-1.999134000	-3.327569000	-0.401113000
H	-2.488577000	-4.116680000	-0.984834000
C	-1.671596000	-3.593983000	6.203036000
C	-2.059310000	-2.247413000	6.015879000
H	-2.623473000	-1.750044000	6.819018000
C	1.259154000	1.500699000	1.968665000
N	-0.385262000	-0.359349000	-2.042669000
N	0.919249000	1.715074000	-0.995747000
C	0.619691000	2.024690000	-4.804486000
C	1.051879000	2.100144000	-2.336133000
C	3.775014000	3.321403000	2.581441000
C	0.488934000	1.427201000	-3.451832000
C	-0.207933000	0.209594000	-3.293938000
C	2.603829000	3.601429000	1.848907000
C	0.162974000	3.342008000	-5.061417000
C	-1.393885000	-1.734469000	-3.567123000
H	-1.940049000	-2.584178000	-3.994368000
C	1.689360000	2.589065000	-0.229576000
C	2.179944000	4.950720000	1.766830000
C	-0.848822000	-0.646988000	-4.258535000
H	-0.889369000	-0.460396000	-5.337723000
C	1.178546000	1.301604000	-5.879316000
C	1.826717000	2.522875000	1.175439000
C	4.504967000	4.338983000	3.219077000
H	5.417564000	4.078082000	3.772405000
C	0.256537000	3.907736000	-6.334309000
H	-0.107178000	4.926095000	-6.537255000
C	1.931729000	3.241595000	-2.397659000
H	2.255019000	3.727113000	-3.325154000
C	4.064009000	5.676969000	3.130488000
C	2.328593000	3.538694000	-1.106285000
H	3.025498000	4.318904000	-0.781722000
C	2.892782000	5.973809000	2.396315000
H	2.561536000	7.021670000	2.342517000
C	0.818710000	3.169709000	-7.401252000
C	1.282990000	1.857541000	-7.165345000
H	1.736936000	1.263837000	-7.970796000
C	-1.106150000	-1.536425000	-2.182585000
H	4.132100000	2.281719000	2.642102000
H	1.264768000	5.189623000	1.203205000
H	-0.070675000	-4.089410000	3.210503000
H	-2.059693000	-0.537146000	4.688989000
H	1.560279000	0.285048000	-5.698718000
H	-0.289127000	3.920167000	-4.241087000
N	-1.660990000	1.302760000	-0.140819000
O	-2.026481000	-4.161434000	7.392098000
C	-1.661794000	-5.511289000	7.625359000
H	-2.118648000	-6.199932000	6.878220000
H	-0.556654000	-5.651295000	7.609378000
H	-2.042261000	-5.768966000	8.631761000
O	4.693252000	6.739536000	3.710178000
C	5.872985000	6.494725000	4.457473000
H	5.683153000	5.821247000	5.324386000
H	6.677147000	6.048289000	3.828977000
H	6.214465000	7.477486000	4.833696000
O	0.869701000	3.803880000	-8.607484000
C	1.425170000	3.105860000	-9.709663000
H	2.491588000	2.835999000	-9.534304000
H	0.855691000	2.177077000	-9.942089000
H	1.365372000	3.791087000	-10.576127000
C	-3.582266000	2.688033000	0.306900000
C	-2.267095000	2.539136000	-0.519345000
C	-2.627334000	2.480100000	-2.037882000
C	-3.369913000	3.770851000	-2.436164000
C	-2.463898000	4.991823000	-2.169719000
C	-2.122315000	5.061748000	-0.666376000
C	-3.419677000	5.196820000	0.155606000
C	-4.323134000	3.974757000	-0.111709000
C	-4.669265000	3.903553000	-1.614652000
C	-1.366222000	3.780821000	-0.263592000
H	-4.211784000	1.790099000	0.136250000
H	-3.322955000	2.711025000	1.386236000
H	-1.693735000	2.364185000	-2.620163000
H	-3.250811000	1.582056000	-2.228147000
H	-3.611181000	3.712202000	-3.519904000
H	-1.527764000	4.910519000	-2.764246000
H	-2.974666000	5.924956000	-2.492669000
H	-1.460858000	5.934471000	-0.474174000
H	-3.179922000	5.264719000	1.239324000
H	-3.951953000	6.133785000	-0.118551000
H	-5.257248000	4.058725000	0.485145000
H	-5.224589000	4.816532000	-1.921980000
H	-5.336098000	3.036162000	-1.812471000
H	-1.075133000	3.800935000	0.807562000

H	-0.441018000	3.692175000	-0.860479000

Structure of ^{4}41

Fe	-0.227393000	0.420788000	-0.227660000
N	0.526380000	0.486185000	1.574066000
N	-0.946103000	-1.328868000	0.182075000
C	-0.914048000	-2.250482000	3.832425000
C	-1.163918000	-1.992667000	1.365694000
C	-0.622134000	-1.506613000	2.582803000
C	0.186363000	-0.326062000	2.647327000
C	-0.609493000	-3.625468000	3.938992000
C	1.422137000	1.404000000	3.485836000
H	1.956200000	2.098401000	4.143094000
C	-1.604553000	-1.972832000	-0.834615000
C	0.776989000	0.234060000	3.844637000
H	0.717218000	-0.210127000	4.844272000
C	-1.518102000	-1.617720000	4.949183000
C	-0.879189000	-4.350854000	5.110347000
H	-0.608312000	-5.414678000	5.156566000
C	-2.018208000	-3.125861000	1.078432000
H	-2.381889000	-3.852692000	1.813798000
C	-2.285099000	-3.113184000	-0.295287000
H	-2.893665000	-3.832036000	-0.857107000
C	-1.477938000	-3.702176000	6.212181000
C	-1.799088000	-2.327895000	6.117400000
H	-2.280635000	-1.843806000	6.980124000
C	1.275186000	1.547113000	2.053950000
N	-0.554810000	-0.236030000	-1.996840000
N	0.957176000	1.745814000	-0.951924000
C	0.659147000	2.017178000	-4.760659000
C	1.120096000	2.086179000	-2.292228000
C	3.755446000	3.375046000	2.665697000
C	0.491964000	1.439188000	-3.406806000
C	-0.320858000	0.291694000	-3.246248000
C	2.613668000	3.651006000	1.883854000
C	0.349723000	3.378530000	-5.014756000
C	-1.704919000	-1.526086000	-3.488016000
H	-2.334075000	-2.324246000	-3.899869000
C	1.709865000	2.623769000	-0.185574000
C	2.232804000	5.007372000	1.720348000
C	-1.058320000	-0.509610000	-4.200403000
H	-1.089038000	-0.341881000	-5.282988000
C	1.096703000	1.228275000	-5.847101000
C	1.829848000	2.568873000	1.232742000
C	4.498523000	4.402467000	3.268907000
H	5.390713000	4.144929000	3.855995000
C	0.464021000	3.921028000	-6.295105000
H	0.207653000	4.971767000	-6.496609000
C	2.042482000	3.193962000	-2.366559000
H	2.409368000	3.644238000	-3.295466000
C	4.098891000	5.746586000	3.101668000
C	2.401080000	3.530709000	-1.070692000
H	3.106953000	4.306685000	-0.755971000
C	2.956104000	6.038909000	2.321294000
H	2.655207000	7.091204000	2.209139000
C	0.903407000	3.117235000	-7.372898000
C	1.223353000	1.761777000	-7.139745000
H	1.582433000	1.116490000	-7.953220000
C	-1.375841000	-1.336323000	-2.107017000
H	4.085066000	2.331469000	2.782875000
H	1.339307000	5.241524000	1.121582000
H	-0.123766000	-4.131829000	3.090952000
H	-1.791763000	-0.554155000	4.878664000
H	1.368979000	0.176885000	-5.668674000
H	-0.012818000	4.006760000	-4.187116000
N	-1.580816000	1.438698000	0.035762000
O	-1.781001000	-4.308071000	7.394525000
C	-1.478966000	-5.686331000	7.540309000
H	-2.027843000	-6.311490000	6.799722000
H	-0.387817000	-5.884025000	7.435740000
H	-1.800313000	-5.970419000	8.559966000
O	4.743274000	6.817599000	3.644043000
C	5.896679000	6.579968000	4.435009000
H	5.665008000	5.956829000	5.328647000
H	6.703506000	6.081471000	3.851019000
H	6.254942000	7.571387000	4.770256000
O	0.986915000	3.734169000	-8.584777000
C	1.417142000	2.971078000	-9.700635000
H	2.449832000	2.578346000	-9.559883000
H	0.733967000	2.115563000	-9.905299000
H	1.407576000	3.655739000	-10.569420000
C	-3.536641000	2.753979000	0.339204000
C	-2.242169000	2.570826000	-0.522924000
C	-2.672083000	2.367533000	-2.003284000
C	-3.491650000	3.575775000	-2.498438000
C	-2.633631000	4.854445000	-2.390938000
C	-2.229230000	5.086147000	-0.919725000
C	-3.497920000	5.242810000	-0.055944000
C	-4.351930000	3.962490000	-0.164150000
C	-4.760080000	3.734149000	-1.634616000
C	-1.404968000	3.880814000	-0.424583000
H	-4.130687000	1.818123000	0.279221000
H	-3.236838000	2.887938000	1.399837000
H	-1.764172000	2.245356000	-2.623282000
H	-3.252887000	1.424563000	-2.085891000
H	-3.778205000	3.407091000	-3.559678000
H	-1.723349000	4.751440000	-3.021711000
H	-3.198514000	5.731109000	-2.777858000
H	-1.608158000	6.006004000	-0.843873000
H	-3.216426000	5.424922000	1.004469000
H	-4.086255000	6.124843000	-0.392774000
H	-5.262486000	4.065005000	0.466449000
H	-5.370521000	4.589730000	-1.999240000
H	-5.393453000	2.823895000	-1.717370000
H	-1.081678000	4.017855000	0.629382000
H	-0.490804000	3.773035000	-1.039845000

Structure of ^{2}2

Fe	-0.005855000	0.210484000	-0.312829000
N	0.491056000	0.477308000	1.509086000
N	-0.727757000	-1.476978000	0.112591000
C	-0.964194000	-2.244071000	3.792897000
C	-1.028231000	-2.101552000	1.301625000
C	-0.616823000	-1.538412000	2.537090000
C	0.121409000	-0.312589000	2.600021000
C	-0.637270000	-3.607590000	3.970796000
C	1.311389000	1.447232000	3.433882000
H	1.813002000	2.163321000	4.093713000
C	-1.302674000	-2.180515000	-0.924601000
C	0.649762000	0.287001000	3.801490000
H	0.545898000	-0.122817000	4.812011000
C	-1.651779000	-1.586715000	4.846329000
C	-0.966303000	-4.296771000	5.148431000
H	-0.676042000	-5.351286000	5.251709000
C	-1.825480000	-3.267060000	0.998738000
H	-2.241882000	-3.964711000	1.734736000
C	-1.987827000	-3.316873000	-0.393050000
H	-2.544568000	-4.067520000	-0.967026000
C	-1.649601000	-3.624125000	6.185787000
C	-1.992555000	-2.261113000	6.019679000
H	-2.539291000	-1.756290000	6.830216000
C	1.220247000	1.553226000	1.997777000
N	-0.344063000	-0.385360000	-2.069094000
N	0.905302000	1.746202000	-0.987070000

C	0.603153000	2.031521000	-4.800107000
C	1.028997000	2.124869000	-2.324934000
C	3.699510000	3.345216000	2.634684000
C	0.462797000	1.440923000	-3.449572000
C	-0.219240000	0.205246000	-3.306153000
C	2.561792000	3.654354000	1.857208000
C	0.219451000	3.373175000	-5.061843000
C	-1.440369000	-1.721178000	-3.566704000
H	-2.029550000	-2.547292000	-3.982961000
C	1.635871000	2.648371000	-0.226462000
C	2.200907000	5.019632000	1.726348000
C	-0.911261000	-0.625683000	-4.263645000
H	-1.016145000	-0.415311000	-5.334398000
C	1.108362000	1.269742000	-5.878625000
C	1.773955000	2.585301000	1.189075000
C	4.457535000	4.346582000	3.262003000
H	5.344660000	4.061713000	3.843959000
C	0.332147000	3.921942000	-6.339804000
H	0.019366000	4.956358000	-6.546807000
C	1.887665000	3.281960000	-2.397986000
H	2.205836000	3.775105000	-3.323095000
C	4.078239000	5.700493000	3.125356000
C	2.265525000	3.604510000	-1.104516000
H	2.940068000	4.408478000	-0.790293000
C	2.939587000	6.026381000	2.351163000
H	2.651795000	7.084764000	2.263002000
C	0.844653000	3.146957000	-7.407585000
C	1.235897000	1.810962000	-7.167191000
H	1.651235000	1.188826000	-7.971736000
C	-1.082183000	-1.543100000	-2.194349000
H	4.013130000	2.294479000	2.730754000
H	1.309603000	5.284970000	1.137422000
H	-0.085953000	-4.133772000	3.176576000
H	-1.944816000	-0.533331000	4.721383000
H	1.437714000	0.235684000	-5.695455000
H	-0.199757000	3.980434000	-4.245300000
O	-2.016165000	-4.194784000	7.365488000
C	-1.699634000	-5.564513000	7.580893000
H	-2.186564000	-6.222785000	6.827072000
H	-0.600985000	-5.741019000	7.557324000
H	-2.085128000	-5.818528000	8.585683000
O	4.736916000	6.748056000	3.692508000
C	5.886887000	6.475300000	4.483226000
H	5.641041000	5.836410000	5.360899000
H	6.686437000	5.978860000	3.889063000
H	6.257643000	7.453739000	4.841066000
O	0.922975000	3.768774000	-8.615211000
C	1.429543000	3.034029000	-9.722698000
H	2.478501000	2.703959000	-9.551351000
H	0.802667000	2.141858000	-9.945775000
H	1.403465000	3.720294000	-10.589339000

Structure of ^{4}2

Fe	-0.067921000	0.245972000	-0.304972000
N	0.466502000	0.483039000	1.505397000
N	-0.806957000	-1.425735000	0.122860000
C	-0.974090000	-2.248263000	3.801059000
C	-1.078211000	-2.069858000	1.308813000
C	-0.641699000	-1.527743000	2.545979000
C	0.099216000	-0.314921000	2.602497000
C	-0.616531000	-3.603483000	3.974447000
C	1.299116000	1.444301000	3.427680000
H	1.809096000	2.158084000	4.083912000
C	-1.387991000	-2.133234000	-0.924214000
C	0.637167000	0.285666000	3.797413000
H	0.535191000	-0.125508000	4.807685000
C	-1.675427000	-1.610967000	4.856388000
C	-0.930785000	-4.304991000	5.149882000
H	-0.620326000	-5.354347000	5.247537000
C	-1.865260000	-3.237622000	1.001769000
H	-2.256799000	-3.949999000	1.737339000
C	-2.050290000	-3.276397000	-0.389195000
H	-2.603847000	-4.031289000	-0.960604000
C	-1.625268000	-3.650919000	6.191153000
C	-1.996997000	-2.295526000	6.030343000
H	-2.550559000	-1.804173000	6.844615000
C	1.200967000	1.560051000	1.993641000
N	-0.413855000	-0.338522000	-2.056203000
N	0.887280000	1.746485000	-0.982471000
C	0.607252000	2.035658000	-4.806309000
C	1.010574000	2.130843000	-2.328042000
C	3.714751000	3.351026000	2.595931000
C	0.449182000	1.457508000	-3.448772000
C	-0.255170000	0.234401000	-3.298285000
C	2.548234000	3.659823000	1.864329000
C	0.205357000	3.365826000	-5.092197000
C	-1.495256000	-1.683492000	-3.558024000
H	-2.081866000	-2.508440000	-3.980091000
C	1.614218000	2.652221000	-0.216474000
C	2.162721000	5.020892000	1.779658000
C	-0.937054000	-0.600691000	-4.255071000
H	-1.013346000	-0.401856000	-5.330470000
C	1.148076000	1.271601000	-5.864249000
C	1.754480000	2.592017000	1.193874000
C	4.476590000	4.348969000	3.226182000
H	5.383688000	4.063753000	3.776495000
C	0.339045000	3.904132000	-6.373590000
H	0.016053000	4.931859000	-6.597848000
C	1.861399000	3.292391000	-2.389700000
H	2.173488000	3.792725000	-3.313111000
C	4.074468000	5.699448000	3.132731000
C	2.234276000	3.612930000	-1.094888000
H	2.897356000	4.424095000	-0.774360000
C	2.908014000	6.025460000	2.402124000
H	2.603816000	7.081478000	2.345277000
C	0.885689000	3.126382000	-7.421351000
C	1.292026000	1.799822000	-7.157459000
H	1.731459000	1.174430000	-7.946645000
C	-1.164997000	-1.501917000	-2.183217000
H	4.045131000	2.302647000	2.658981000
H	1.250107000	5.286565000	1.223924000
H	-0.056725000	-4.116212000	3.177033000
H	-1.989793000	-0.562992000	4.735954000
H	1.488015000	0.243903000	-5.663843000
H	-0.240141000	3.975452000	-4.291182000
O	-1.977575000	-4.234306000	7.370683000
C	-1.629121000	-5.596575000	7.578954000
H	-2.103163000	-6.263426000	6.824128000
H	-0.526740000	-5.748590000	7.550735000
H	-2.004966000	-5.864235000	8.583984000
O	4.736491000	6.744673000	3.704017000
C	5.915374000	6.469277000	4.448483000
H	5.708656000	5.805966000	5.318367000
H	6.701364000	5.998012000	3.816522000
H	6.285701000	7.443727000	4.817827000
O	0.978842000	3.736143000	-8.635707000
C	1.518203000	2.995312000	-9.722492000
H	2.568642000	2.683136000	-9.526964000
H	0.910020000	2.090204000	-9.946358000
H	1.499081000	3.669279000	-10.599049000

Structure of ^{6}2

Fe	-0.475396000	0.544667000	-0.224841000
N	0.296959000	0.582093000	1.588463000

N	-0.993466000	-1.347762000	0.149823000
C	-1.017253000	-2.237838000	3.815712000
C	-1.199515000	-2.022619000	1.329663000
C	-0.754598000	-1.475718000	2.568746000
C	-0.032687000	-0.237458000	2.662959000
C	-0.613028000	-3.585260000	3.944137000
C	1.211291000	1.504355000	3.477724000
H	1.753034000	2.195476000	4.133010000
C	-1.477533000	-2.081431000	-0.905465000
C	0.550325000	0.343444000	3.853030000
H	0.488279000	-0.080328000	4.861770000
C	-1.688404000	-1.643001000	4.915066000
C	-0.857520000	-4.320436000	5.115241000
H	-0.511448000	-5.361358000	5.176958000
C	-1.888499000	-3.254828000	1.003948000
H	-2.226010000	-4.011795000	1.722279000
C	-2.050369000	-3.291262000	-0.388689000
H	-2.522793000	-4.090421000	-0.973700000
C	-1.527792000	-3.710053000	6.198144000
C	-1.942929000	-2.362402000	6.084375000
H	-2.475111000	-1.905221000	6.932085000
C	1.068047000	1.643668000	2.044231000
N	-0.585752000	-0.244810000	-2.063037000
N	0.696248000	1.928223000	-0.994810000
C	0.561566000	2.069038000	-4.813210000
C	0.880756000	2.253201000	-2.334508000
C	3.623493000	3.339268000	2.605075000
C	0.328183000	1.534577000	-3.449707000
C	-0.384267000	0.307700000	-3.305835000
C	2.473917000	3.695903000	1.867370000
C	0.240687000	3.412446000	-5.140647000
C	-1.515177000	-1.676014000	-3.571426000
H	-2.034775000	-2.543274000	-3.997946000
C	1.485198000	2.760730000	-0.212278000
C	2.160886000	5.074325000	1.758004000
C	-0.987294000	-0.584904000	-4.275468000
H	-1.027934000	-0.425402000	-5.359750000
C	1.107853000	1.258254000	-5.833871000
C	1.626118000	2.662734000	1.209453000
C	4.435821000	4.306619000	3.218819000
H	5.328400000	3.984033000	3.772253000
C	0.448613000	3.915348000	-6.426050000
H	0.185604000	4.952440000	-6.683132000
C	1.819043000	3.351689000	-2.395641000
H	2.196262000	3.811065000	-3.316463000
C	4.102749000	5.674470000	3.104873000
C	2.193325000	3.661364000	-1.095287000
H	2.921581000	4.417152000	-0.780030000
C	2.955275000	6.048478000	2.366861000
H	2.705666000	7.117636000	2.292870000
C	0.995388000	3.089206000	-7.436359000
C	1.327841000	1.751085000	-7.129678000
H	1.770084000	1.089396000	-7.887020000
C	-1.251674000	-1.439838000	-2.180395000
H	3.902136000	2.277080000	2.681905000
H	1.264976000	5.377914000	1.194707000
H	-0.068799000	-4.062057000	3.114648000
H	-2.033940000	-0.601118000	4.833174000
H	1.395688000	0.222449000	-5.598536000
H	-0.202364000	4.060893000	-4.369352000
O	-1.815180000	-4.329011000	7.376812000
C	-1.420431000	-5.685135000	7.539346000
H	-1.905511000	-6.348252000	6.788358000
H	-0.316293000	-5.804336000	7.464083000
H	-1.747984000	-5.985706000	8.551926000
O	4.816149000	6.692793000	3.661042000
C	5.977855000	6.370011000	4.413996000
H	5.735432000	5.729605000	5.291641000
H	6.741756000	5.852518000	3.791353000
H	6.394100000	7.330152000	4.771577000
O	1.163528000	3.666995000	-8.657558000
C	1.698239000	2.875820000	-9.711144000
H	2.724832000	2.515650000	-9.475922000
H	1.051580000	1.997979000	-9.935179000
H	1.740609000	3.530465000	-10.601381000

Structure of 3

C	-0.135455000	0.047008000	-5.227830000
C	-1.333504000	-0.365862000	-4.605431000
C	0.970481000	0.362183000	-4.409142000
C	-1.425318000	-0.462061000	-3.206735000
C	0.884128000	0.266946000	-3.009885000
C	-0.315723000	-0.144749000	-2.403403000
H	-2.206117000	-0.619856000	-5.230159000
H	1.915285000	0.682508000	-4.879105000
H	-2.368096000	-0.790338000	-2.740851000
H	1.760410000	0.512353000	-2.388932000
H	-0.384618000	-0.222123000	-1.306926000
C	-0.060103000	0.209499000	-6.730626000
H	0.979536000	0.024076000	-7.077779000
H	-0.699577000	-0.554418000	-7.224293000
C	-0.503730000	1.612391000	-7.192558000
H	-1.541830000	1.799095000	-6.837112000
H	0.130270000	2.376195000	-6.689094000
C	-0.433191000	1.794802000	-8.713206000
H	0.607982000	1.612049000	-9.063387000
H	-1.070874000	1.037473000	-9.219583000
C	-0.878082000	3.194617000	-9.154928000
H	-1.923765000	3.384009000	-8.842042000
H	-0.246180000	3.974312000	-8.669515000
N	-0.867642000	3.378629000	-10.620569000
N	0.213712000	3.339694000	-11.210126000
N	1.148478000	3.331170000	-11.890093000

Structure of ^{2}Int1

Fe	-0.136428000	0.123976000	-0.817953000
N	0.305517000	0.224498000	1.054827000
N	-0.876364000	-1.606305000	-0.563321000
C	-1.458619000	-2.552141000	3.037436000
C	-1.248446000	-2.309514000	0.564455000
C	-0.939305000	-1.815791000	1.858086000
C	-0.185930000	-0.612839000	2.059663000
C	-1.265218000	-3.944349000	3.179462000
C	0.934226000	1.090646000	3.097758000
H	1.379174000	1.762284000	3.839844000
C	-1.324363000	-2.278418000	-1.690870000
C	0.232509000	-0.079158000	3.336219000
H	0.034908000	-0.542306000	4.309225000
C	-2.224539000	-1.892183000	4.034638000
C	-1.823379000	-4.663303000	4.249115000
H	-1.642237000	-5.744468000	4.320105000
C	-1.979768000	-3.481722000	0.124740000
H	-2.436952000	-4.234318000	0.775691000
C	-2.016715000	-3.461391000	-1.272797000
H	-2.489547000	-4.202160000	-1.928852000
C	-2.600122000	-3.990039000	5.218005000
C	-2.785471000	-2.592271000	5.104313000
H	-3.399675000	-2.081843000	5.860849000
C	0.980283000	1.277799000	1.667828000
N	-0.285457000	-0.412459000	-2.625012000
N	0.958889000	1.621757000	-1.323287000
C	0.941494000	2.139034000	-5.120880000
C	1.166394000	2.089294000	-2.621895000
C	3.329823000	3.142214000	2.614419000
C	0.687494000	1.470939000	-3.819726000

C	-0.015533000	0.239089000	-3.811173000
C	2.241842000	3.430166000	1.764022000
C	0.542721000	3.481730000	-5.345588000
C	-1.181922000	-1.679591000	-4.308125000
H	-1.699763000	-2.491002000	-4.834072000
C	1.594570000	2.494727000	-0.449414000
C	1.800804000	4.774284000	1.679597000
C	-0.584119000	-0.551507000	-4.881433000
H	-0.553716000	-0.295081000	-5.946935000
C	1.575314000	1.455599000	-6.181831000
C	1.577504000	2.362502000	0.967690000
C	3.967210000	4.148637000	3.359039000
H	4.819253000	3.883969000	4.000075000
C	0.764186000	4.107699000	-6.573882000
H	0.443195000	5.144922000	-6.752712000
C	2.004903000	3.266285000	-2.550808000
H	2.379296000	3.823784000	-3.416722000
C	3.512863000	5.482393000	3.264638000
C	2.269538000	3.516801000	-1.215710000
H	2.889711000	4.316351000	-0.795908000
C	2.420969000	5.784947000	2.417490000
H	2.072792000	6.827453000	2.364021000
C	1.401325000	3.409194000	-7.626324000
C	1.809522000	2.072910000	-7.421355000
H	2.319555000	1.508207000	-8.213857000
C	-0.991091000	-1.576739000	-2.892061000
H	3.700450000	2.107694000	2.680634000
H	0.941361000	5.012671000	1.034855000
H	-0.649637000	-4.477363000	2.439524000
H	-2.402527000	-0.809983000	3.945220000
H	1.916458000	0.421022000	-6.024198000
H	0.030344000	4.030594000	-4.540642000
O	-3.201798000	-4.591248000	6.279664000
C	-3.106324000	-6.005200000	6.395013000
H	-3.542598000	-6.516823000	5.508005000
H	-2.052245000	-6.338922000	6.522594000
H	-3.684735000	-6.284157000	7.295158000
O	4.055268000	6.532653000	3.942467000
C	5.153750000	6.280469000	4.808480000
H	4.881080000	5.575638000	5.625846000
H	6.028170000	5.868704000	4.256180000
H	5.432424000	7.255182000	5.250466000
O	1.575451000	4.101082000	-8.786696000
C	2.195810000	3.437336000	-9.880110000
H	3.234211000	3.120221000	-9.634729000
H	1.614266000	2.544770000	-10.202973000
H	2.228194000	4.167450000	-10.710092000
C	-5.755158000	-2.864546000	1.884584000
C	-4.957026000	-3.942405000	2.325621000
C	-7.056733000	-3.141557000	1.415131000
C	-5.442495000	-5.259832000	2.297673000
C	-7.546008000	-4.458958000	1.383308000
C	-6.739595000	-5.524073000	1.823495000
H	-3.943688000	-3.739514000	2.701954000
H	-7.697247000	-2.311662000	1.072592000
H	-4.801592000	-6.083489000	2.651482000
H	-8.565996000	-4.655653000	1.015956000
H	-7.124274000	-6.556003000	1.800876000
C	-5.188586000	-1.462735000	1.871283000
H	-5.953896000	-0.730110000	2.209606000
H	-4.355516000	-1.415067000	2.602764000
C	-4.649184000	-1.049270000	0.486323000
H	-4.075640000	-1.898687000	0.056705000
H	-5.497003000	-0.859336000	-0.209478000
C	-3.718328000	0.164648000	0.565018000
H	-4.217385000	1.017589000	1.076306000
H	-2.842397000	-0.111514000	1.189210000
C	-3.191427000	0.606797000	-0.807781000
H	-3.043648000	-0.266988000	-1.471108000
H	-3.878346000	1.310046000	-1.325367000
N	-1.844570000	1.217571000	-0.713421000
N	-1.724058000	2.330149000	-0.143880000
N	-1.381197000	3.321100000	0.338602000

Structure of ^{4}Int1

Fe	-0.069516000	0.077242000	-0.843681000
N	0.292920000	0.202034000	1.027930000
N	-0.789696000	-1.647207000	-0.584274000
C	-1.436498000	-2.607485000	3.006566000
C	-1.199910000	-2.343687000	0.529739000
C	-0.910955000	-1.859589000	1.837695000
C	-0.173893000	-0.660524000	2.034610000
C	-1.260583000	-4.004517000	3.125696000
C	0.925241000	1.054197000	3.073019000
H	1.368726000	1.727361000	3.814954000
C	-1.229938000	-2.307777000	-1.729770000
C	0.243954000	-0.126845000	3.309202000
H	0.060255000	-0.600532000	4.279764000
C	-2.187961000	-1.956079000	4.020554000
C	-1.822114000	-4.734515000	4.186295000
H	-1.655474000	-5.819187000	4.237313000
C	-1.945647000	-3.489341000	0.072532000
H	-2.436704000	-4.230605000	0.712276000
C	-1.954105000	-3.466820000	-1.332883000
H	-2.435210000	-4.192673000	-1.999301000
C	-2.582375000	-4.067775000	5.172398000
C	-2.748687000	-2.666144000	5.083803000
H	-3.350623000	-2.160208000	5.853148000
C	0.961058000	1.255833000	1.644842000
N	-0.162687000	-0.447341000	-2.653882000
N	0.967279000	1.601985000	-1.332031000
C	1.007587000	2.143930000	-5.136396000
C	1.198980000	2.077443000	-2.634733000
C	3.320306000	3.155940000	2.595923000
C	0.763634000	1.465814000	-3.838505000
C	0.087834000	0.213971000	-3.833059000
C	2.204341000	3.419422000	1.775505000
C	0.577569000	3.476911000	-5.360754000
C	-1.078084000	-1.704506000	-4.329102000
H	-1.605973000	-2.508688000	-4.855974000
C	1.573962000	2.481506000	-0.444104000
C	1.707369000	4.745677000	1.737068000
C	-0.485420000	-0.566160000	-4.900212000
H	-0.468256000	-0.297773000	-5.963050000
C	1.657599000	1.477081000	-6.198078000
C	1.560460000	2.345386000	0.966510000
C	3.929750000	4.167615000	3.357964000
H	4.802701000	3.921153000	3.977861000
C	0.788804000	4.110757000	-6.587144000
H	0.445306000	5.141050000	-6.764424000
C	2.000589000	3.273569000	-2.538650000
H	2.373529000	3.847370000	-3.394441000
C	3.420989000	5.483680000	3.306830000
C	2.232404000	3.522051000	-1.197071000
H	2.819296000	4.337966000	-0.760582000
C	2.302327000	5.763165000	2.487802000
H	1.913496000	6.792448000	2.465775000
C	1.442032000	3.428507000	-7.640052000
C	1.878407000	2.100907000	-7.437028000
H	2.399969000	1.548092000	-8.230436000
C	-0.875891000	-1.610074000	-2.921267000
H	3.732390000	2.135324000	2.628674000
H	0.827992000	4.967124000	1.112562000
H	-0.655284000	-4.533627000	2.374455000
H	-2.354373000	-0.870669000	3.949920000
H	2.020420000	0.449377000	-6.042761000
H	0.049332000	4.011924000	-4.556628000
O	-3.185676000	-4.679080000	6.227934000
C	-3.110223000	-6.095771000	6.317036000
H	-3.559261000	-6.584985000	5.423740000
H	-2.060435000	-6.447452000	6.431921000

H	-3.687189000	-6.382903000	7.215592000
O	3.934932000	6.538476000	4.002233000
C	5.060042000	6.308057000	4.838522000
H	4.833488000	5.571976000	5.642674000
H	5.938580000	5.945584000	4.258558000
H	5.309974000	7.281248000	5.300844000
O	1.601741000	4.126075000	-8.799742000
C	2.235915000	3.476986000	-9.893668000
H	3.281456000	3.182929000	-9.649551000
H	1.674452000	2.571642000	-10.216791000
H	2.251465000	4.207907000	-10.723485000
C	-5.765135000	-2.777856000	2.021571000
C	-4.974681000	-3.896615000	2.364732000
C	-7.120939000	-2.989249000	1.693392000
C	-5.521681000	-5.189846000	2.378245000
C	-7.672089000	-4.282501000	1.703606000
C	-6.873529000	-5.388578000	2.045078000
H	-3.918893000	-3.745429000	2.633930000
H	-7.754622000	-2.126122000	1.429259000
H	-4.886178000	-6.046449000	2.655237000
H	-8.733720000	-4.427572000	1.446925000
H	-7.306008000	-6.401593000	2.056224000
C	-5.139382000	-1.402942000	1.957657000
H	-5.861913000	-0.630483000	2.300396000
H	-4.280855000	-1.373760000	2.659515000
C	-4.629442000	-1.047918000	0.546420000
H	-4.085697000	-1.923919000	0.131074000
H	-5.493595000	-0.866842000	-0.131500000
C	-3.674149000	0.150153000	0.550458000
H	-4.140339000	1.031464000	1.044696000
H	-2.778005000	-0.114859000	1.150132000
C	-3.205384000	0.524381000	-0.860749000
H	-3.016405000	-0.388410000	-1.457971000
H	-3.955484000	1.133069000	-1.409508000
N	-1.905468000	1.233430000	-0.855438000
N	-1.846816000	2.424237000	-0.506146000
N	-1.592312000	3.511539000	-0.219098000

Structure of ^{6}Int1

Fe	0.048202000	0.016303000	-0.875774000
N	0.295528000	0.205917000	1.021492000
N	-0.686649000	-1.697486000	-0.615011000
C	-1.434383000	-2.614644000	2.971664000
C	-1.123551000	-2.392530000	0.494994000
C	-0.892432000	-1.880689000	1.814287000
C	-0.170549000	-0.648016000	2.021886000
C	-1.304571000	-4.021925000	3.081881000
C	0.896522000	1.085942000	3.059068000
H	1.308077000	1.771000000	3.808107000
C	-1.096926000	-2.374897000	-1.767947000
C	0.219624000	-0.112067000	3.294258000
H	0.034367000	-0.583357000	4.264947000
C	-2.163101000	-1.951772000	4.000391000
C	-1.882523000	-4.742571000	4.138001000
H	-1.745538000	-5.831847000	4.180959000
C	-1.845746000	-3.538701000	0.029515000
H	-2.359066000	-4.274471000	0.657602000
C	-1.816651000	-3.523551000	-1.393428000
H	-2.274169000	-4.256477000	-2.068214000
C	-2.623409000	-4.062622000	5.130296000
C	-2.746886000	-2.654841000	5.053598000
H	-3.336812000	-2.138702000	5.825574000
C	0.954118000	1.272013000	1.643030000
N	-0.053590000	-0.478986000	-2.692441000
N	0.961735000	1.638814000	-1.356651000
C	1.028311000	2.181027000	-5.150794000
C	1.214467000	2.109378000	-2.644798000
C	3.249656000	3.149054000	2.653782000
C	0.797931000	1.485140000	-3.869905000
C	0.172665000	0.198222000	-3.873164000
C	2.189731000	3.435186000	1.766101000
C	0.644089000	3.537897000	-5.334521000
C	-0.937988000	-1.759856000	-4.363511000
H	-1.445263000	-2.575904000	-4.891614000
C	1.568673000	2.516678000	-0.454864000
C	1.751760000	4.780337000	1.663539000
C	-0.377750000	-0.587402000	-4.936989000
H	-0.388134000	-0.309017000	-5.996939000
C	1.621882000	1.519008000	-6.252582000
C	1.550082000	2.368199000	0.954834000
C	3.863280000	4.157328000	3.413694000
H	4.697297000	3.895646000	4.079210000
C	0.841486000	4.192020000	-6.550782000
H	0.527694000	5.236957000	-6.694629000
C	2.020110000	3.297511000	-2.546356000
H	2.414624000	3.863978000	-3.396625000
C	3.410239000	5.491132000	3.303047000
C	2.241104000	3.545559000	-1.195939000
H	2.840666000	4.351598000	-0.758603000
C	2.345345000	5.791777000	2.421116000
H	1.997241000	6.833546000	2.356100000
C	1.439487000	3.513099000	-7.639365000
C	1.831521000	2.165206000	-7.480293000
H	2.312281000	1.614431000	-8.300585000
C	-0.734668000	-1.661112000	-2.971134000
H	3.619487000	2.115372000	2.732213000
H	0.914260000	5.019288000	0.990654000
H	-0.713790000	-4.563225000	2.328318000
H	-2.300101000	-0.861749000	3.942136000
H	1.955453000	0.477128000	-6.132718000
H	0.154805000	4.072628000	-4.506161000
O	-3.245393000	-4.664061000	6.180228000
C	-3.212177000	-6.083037000	6.258538000
H	-3.674064000	-6.551438000	5.360868000
H	-2.173324000	-6.466239000	6.371863000
H	-3.798792000	-6.359796000	7.154062000
O	3.929718000	6.541571000	3.995145000
C	4.997890000	6.292385000	4.900462000
H	4.696288000	5.588551000	5.708078000
H	5.891438000	5.881496000	4.379441000
H	5.258759000	7.268387000	5.350046000
O	1.592793000	4.233454000	-8.785156000
C	2.174580000	3.591229000	-9.912096000
H	3.214413000	3.253789000	-9.702718000
H	1.572156000	2.715602000	-10.242774000
H	2.195437000	4.342966000	-10.722912000
C	-5.791540000	-2.749154000	2.127104000
C	-5.003314000	-3.888610000	2.401304000
C	-7.179782000	-2.920954000	1.946960000
C	-5.585568000	-5.162411000	2.495481000
C	-7.767109000	-4.195401000	2.038791000
C	-6.971431000	-5.321662000	2.313337000
H	-3.920207000	-3.769267000	2.552777000
H	-7.810169000	-2.040913000	1.736155000
H	-4.950631000	-6.035672000	2.716147000
H	-8.854063000	-4.309033000	1.898773000
H	-7.431162000	-6.319831000	2.388795000
C	-5.132154000	-1.395979000	1.986855000
H	-5.839286000	-0.588882000	2.276973000
H	-4.277836000	-1.345385000	2.693365000
C	-4.600497000	-1.135760000	0.563642000
H	-4.053114000	-2.037777000	0.212498000
H	-5.454740000	-0.996508000	-0.136288000
C	-3.642137000	0.058329000	0.504479000
H	-4.114012000	0.970484000	0.933295000
H	-2.754866000	-0.167979000	1.132116000
C	-3.155352000	0.338641000	-0.921446000
H	-2.930571000	-0.610388000	-1.443994000
H	-3.911259000	0.883545000	-1.526719000
N	-1.876289000	1.084365000	-0.946786000
N	-1.861209000	2.299688000	-0.697738000

N	-1.661497000	3.417232000	-0.500042000

Structure of ^{2}TS1

Fe	-0.354463000	0.267662000	-0.827033000
N	0.181115000	0.307948000	1.065736000
N	-0.981677000	-1.529469000	-0.561817000
C	-1.509018000	-2.506517000	3.050532000
C	-1.329306000	-2.242207000	0.575140000
C	-1.023862000	-1.749972000	1.869097000
C	-0.295386000	-0.533217000	2.071625000
C	-1.270564000	-3.891812000	3.188239000
C	0.811726000	1.183937000	3.099904000
H	1.252386000	1.863124000	3.837670000
C	-1.368765000	-2.245681000	-1.689246000
C	0.121274000	0.008993000	3.345386000
H	-0.066978000	-0.453328000	4.320420000
C	-2.282092000	-1.870517000	4.057362000
C	-1.794122000	-4.628569000	4.263374000
H	-1.578174000	-5.703493000	4.331412000
C	-2.001408000	-3.445968000	0.138416000
H	-2.436729000	-4.208440000	0.792684000
C	-2.011628000	-3.449431000	-1.261202000
H	-2.434253000	-4.224007000	-1.912412000
C	-2.579796000	-3.979963000	5.241900000
C	-2.809068000	-2.588394000	5.132292000
H	-3.429409000	-2.097577000	5.896698000
C	0.856492000	1.364648000	1.668916000
N	-0.357871000	-0.370276000	-2.631541000
N	0.854364000	1.687343000	-1.334398000
C	0.964397000	2.126742000	-5.141323000
C	1.106230000	2.125743000	-2.638584000
C	3.239114000	3.181928000	2.615096000
C	0.658086000	1.487177000	-3.836741000
C	-0.042541000	0.255185000	-3.824373000
C	2.168902000	3.488205000	1.748165000
C	0.575721000	3.464771000	-5.406791000
C	-1.129541000	-1.709759000	-4.316329000
H	-1.593750000	-2.552921000	-4.842413000
C	1.508988000	2.547880000	-0.457642000
C	1.769880000	4.843590000	1.633926000
C	-0.539202000	-0.580606000	-4.894381000
H	-0.458501000	-0.357141000	-5.964376000
C	1.643558000	1.423481000	-6.159986000
C	1.481285000	2.429094000	0.961721000
C	3.900379000	4.181939000	3.346970000
H	4.738620000	3.903284000	4.000184000
C	0.850081000	4.067563000	-6.635934000
H	0.537658000	5.101168000	-6.847679000
C	1.979180000	3.273271000	-2.564686000
H	2.389446000	3.805609000	-3.430149000
C	3.487792000	5.527138000	3.223823000
C	2.230978000	3.530354000	-1.226731000
H	2.872677000	4.312933000	-0.807636000
C	2.413376000	5.847372000	2.360646000
H	2.097793000	6.898858000	2.285428000
C	1.531893000	3.349821000	-7.646688000
C	1.931061000	2.017765000	-7.399472000
H	2.474971000	1.438809000	-8.158432000
C	-1.013457000	-1.564127000	-2.896017000
H	3.578442000	2.138262000	2.702365000
H	0.923769000	5.094604000	0.976971000
H	-0.645335000	-4.404589000	2.442169000
H	-2.493641000	-0.794205000	3.971632000
H	1.978801000	0.392763000	-5.968192000
H	0.030028000	4.027913000	-4.634268000
O	-3.148826000	-4.599173000	6.310761000
C	-2.997312000	-6.007724000	6.433380000
H	-3.423843000	-6.542453000	5.555377000
H	-1.929652000	-6.299383000	6.550614000
H	-3.553831000	-6.302665000	7.342168000
O	4.055817000	6.572312000	3.887106000
C	5.138017000	6.303609000	4.768901000
H	4.836541000	5.621384000	5.595147000
H	6.005357000	5.857307000	4.232602000
H	5.440437000	7.277472000	5.196797000
O	1.756341000	4.020237000	-8.810564000
C	2.431335000	3.340596000	-9.861133000
H	3.457739000	3.032728000	-9.559951000
H	1.869522000	2.440125000	-10.196668000
H	2.501169000	4.056641000	-10.700975000
C	-5.757642000	-2.834885000	1.875526000
C	-4.967746000	-3.909741000	2.338394000
C	-7.047026000	-3.117547000	1.375686000
C	-5.449363000	-5.228672000	2.302047000
C	-7.532323000	-4.435954000	1.335646000
C	-6.734029000	-5.497988000	1.797892000
H	-3.964770000	-3.704120000	2.739966000
H	-7.680943000	-2.290191000	1.015213000
H	-4.815261000	-6.049798000	2.673648000
H	-8.542734000	-4.636049000	0.944338000
H	-7.115313000	-6.531042000	1.769048000
C	-5.198312000	-1.429970000	1.868883000
H	-5.965505000	-0.704109000	2.217732000
H	-4.360917000	-1.381484000	2.594633000
C	-4.671581000	-1.002229000	0.483626000
H	-4.097521000	-1.846502000	0.044079000
H	-5.526254000	-0.816255000	-0.205345000
C	-3.751349000	0.219825000	0.554087000
H	-4.254930000	1.073263000	1.059024000
H	-2.871334000	-0.036862000	1.178563000
C	-3.237346000	0.669178000	-0.826109000
H	-3.165606000	-0.203757000	-1.508776000
H	-3.930536000	1.397355000	-1.303654000
N	-1.882674000	1.231717000	-0.835520000
N	-1.940052000	2.566191000	-0.024020000
N	-1.223251000	3.448744000	0.158805000

Structure of ^{4}TS1

Fe	-0.100842000	0.121757000	-0.854827000
N	0.280559000	0.242854000	1.041763000
N	-0.614459000	-1.724386000	-0.607125000
C	-1.489644000	-2.551750000	2.969487000
C	-1.088908000	-2.382421000	0.508193000
C	-0.910289000	-1.836964000	1.810628000
C	-0.208975000	-0.605075000	2.033030000
C	-1.325646000	-3.946277000	3.140750000
C	0.878429000	1.105549000	3.091033000
H	1.311204000	1.776229000	3.840921000
C	-0.942355000	-2.445223000	-1.743078000
C	0.179443000	-0.067652000	3.318097000
H	-0.024727000	-0.536175000	4.287016000
C	-2.287788000	-1.863780000	3.923447000
C	-1.950653000	-4.640267000	4.188058000
H	-1.789844000	-5.722536000	4.285176000
C	-1.759346000	-3.577668000	0.049061000
H	-2.277902000	-4.307126000	0.680780000
C	-1.650972000	-3.619836000	-1.349722000
H	-2.047602000	-4.394254000	-2.017072000
C	-2.770024000	-3.940957000	5.102627000
C	-2.918521000	-2.540331000	4.966936000
H	-3.565190000	-2.010632000	5.681763000
C	0.947620000	1.294522000	1.660906000
N	0.011879000	-0.531540000	-2.678320000
N	0.953937000	1.643010000	-1.334357000

C	0.989402000	2.166330000	-5.124995000
C	1.182015000	2.112487000	-2.632586000
C	3.257098000	3.177271000	2.667074000
C	0.775586000	1.463682000	-3.840028000
C	0.200563000	0.161108000	-3.852144000
C	2.204257000	3.448977000	1.767639000
C	0.530861000	3.497481000	-5.309988000
C	-0.762724000	-1.860151000	-4.363816000
H	-1.201404000	-2.713577000	-4.895053000
C	1.591191000	2.506286000	-0.449189000
C	1.765029000	4.791451000	1.639884000
C	-0.286667000	-0.668174000	-4.929489000
H	-0.290012000	-0.399802000	-5.992276000
C	1.645091000	1.537770000	-6.207959000
C	1.564390000	2.372240000	0.967605000
C	3.863100000	4.198136000	3.417257000
H	4.690742000	3.947492000	4.094880000
C	0.714006000	4.162462000	-6.522598000
H	0.344202000	5.187699000	-6.673402000
C	2.016608000	3.289011000	-2.545071000
H	2.403881000	3.848879000	-3.403627000
C	3.409975000	5.529253000	3.281213000
C	2.278036000	3.524255000	-1.207251000
H	2.901999000	4.316444000	-0.779597000
C	2.352024000	5.815059000	2.385727000
H	2.004617000	6.855641000	2.300609000
C	1.375301000	3.520037000	-7.596779000
C	1.844928000	2.197769000	-7.429917000
H	2.376992000	1.679201000	-8.239152000
C	-0.582914000	-1.745162000	-2.949204000
H	3.627728000	2.145301000	2.765184000
H	0.933016000	5.016229000	0.955144000
H	-0.673996000	-4.497879000	2.447093000
H	-2.440701000	-0.780220000	3.811192000
H	2.037293000	0.517760000	-6.078279000
H	-0.000842000	3.999233000	-4.487224000
O	-3.444532000	-4.517629000	6.130517000
C	-3.391571000	-5.932941000	6.264740000
H	-3.786143000	-6.441974000	5.357569000
H	-2.355912000	-6.289924000	6.460354000
H	-4.030952000	-6.187011000	7.130139000
O	3.923128000	6.591768000	3.960195000
C	4.986164000	6.358955000	4.875316000
H	4.681273000	5.667872000	5.692764000
H	5.883369000	5.940856000	4.366253000
H	5.243294000	7.342467000	5.310554000
O	1.511413000	4.248465000	-8.737233000
C	2.161051000	3.647671000	-9.851107000
H	3.213775000	3.374888000	-9.614727000
H	1.622119000	2.738476000	-10.199512000
H	2.155967000	4.403181000	-10.658449000
C	-5.833989000	-2.681935000	2.056538000
C	-4.991264000	-3.784026000	2.325084000
C	-7.221534000	-2.911006000	1.954547000
C	-5.518539000	-5.073295000	2.493230000
C	-7.755044000	-4.202178000	2.119667000
C	-6.905358000	-5.288659000	2.390666000
H	-3.907064000	-3.621228000	2.413975000
H	-7.894251000	-2.062175000	1.747294000
H	-4.840289000	-5.914559000	2.709568000
H	-8.842612000	-4.359384000	2.039107000
H	-7.322832000	-6.299270000	2.524132000
C	-5.230319000	-1.310055000	1.855372000
H	-5.998774000	-0.523326000	2.014126000
H	-4.449719000	-1.153135000	2.629652000
C	-4.576074000	-1.134055000	0.470164000
H	-3.987229000	-2.047468000	0.231942000
H	-5.366888000	-1.068519000	-0.310766000
C	-3.634309000	0.071702000	0.398966000
H	-4.150712000	1.006250000	0.711880000
H	-2.803073000	-0.079735000	1.119533000
C	-3.016052000	0.265980000	-0.998953000
H	-2.845696000	-0.724417000	-1.472048000
H	-3.712535000	0.811602000	-1.673192000
N	-1.721950000	0.937814000	-0.994843000
N	-2.007992000	2.540626000	-0.831840000
N	-1.253978000	3.405104000	-0.730824000

Structure of ^{6}TS1

Fe	-0.203518000	0.217115000	-0.748783000
N	0.203205000	0.360483000	1.125575000
N	-0.788073000	-1.577849000	-0.452976000
C	-1.587615000	-2.361514000	3.146361000
C	-1.233608000	-2.228677000	0.677084000
C	-1.038968000	-1.664464000	1.962463000
C	-0.315969000	-0.437610000	2.142315000
C	-1.363563000	-3.740513000	3.361310000
C	0.872253000	1.234020000	3.141213000
H	1.347852000	1.900199000	3.868831000
C	-1.128616000	-2.314567000	-1.567852000
C	0.100795000	0.111580000	3.407321000
H	-0.132109000	-0.313568000	4.389543000
C	-2.407247000	-1.672816000	4.080427000
C	-1.940291000	-4.420844000	4.444996000
H	-1.731816000	-5.491022000	4.578338000
C	-1.898099000	-3.438739000	0.248279000
H	-2.404338000	-4.157677000	0.901341000
C	-1.813810000	-3.497375000	-1.148842000
H	-2.221940000	-4.277540000	-1.802100000
C	-2.771553000	-3.722137000	5.349525000
C	-2.991268000	-2.336900000	5.159431000
H	-3.649135000	-1.809282000	5.865655000
C	0.944002000	1.376002000	1.708354000
N	-0.180308000	-0.428145000	-2.550402000
N	0.923076000	1.685976000	-1.284387000
C	0.879075000	2.163069000	-5.077427000
C	1.134548000	2.128174000	-2.586439000
C	3.388883000	3.109973000	2.657252000
C	0.654534000	1.496575000	-3.776516000
C	0.006727000	0.231965000	-3.744020000
C	2.345471000	3.439782000	1.765332000
C	0.502197000	3.518268000	-5.274918000
C	-1.013167000	-1.775512000	-4.192788000
H	-1.490560000	-2.625693000	-4.694722000
C	1.633923000	2.504600000	-0.429346000
C	1.992617000	4.806928000	1.627553000
C	-0.529425000	-0.602537000	-4.791157000
H	-0.555712000	-0.351620000	-5.857767000
C	1.466314000	1.477090000	-6.165459000
C	1.626658000	2.397480000	0.990394000
C	4.069267000	4.097049000	3.388250000
H	4.887004000	3.800289000	4.059230000
C	0.697340000	4.150830000	-6.502772000
H	0.389635000	5.195038000	-6.662530000
C	2.032866000	3.258493000	-2.540440000
H	2.418725000	3.793109000	-3.415353000
C	3.702581000	5.453495000	3.241037000
C	2.352418000	3.482473000	-1.211341000
H	3.034745000	4.238288000	-0.807255000
C	2.654511000	5.797756000	2.354362000
H	2.372807000	6.857528000	2.263247000
C	1.289344000	3.450626000	-7.581261000
C	1.677487000	2.103501000	-7.402794000
H	2.155740000	1.539341000	-8.215133000
C	-0.793739000	-1.637805000	-2.787058000
H	3.692400000	2.057019000	2.763196000
H	1.162929000	5.076006000	0.956799000
H	-0.703737000	-4.289747000	2.673281000
H	-2.611380000	-0.602397000	3.927808000
H	1.797833000	0.436883000	-6.027528000
H	0.022604000	4.066380000	-4.449894000

O	-3.394808000	-4.286084000	6.416844000
C	-3.262980000	-5.689035000	6.613231000
H	-3.651510000	-6.260481000	5.740986000
H	-2.205203000	-5.981799000	6.796756000
H	-3.866328000	-5.935726000	7.506216000
O	4.291667000	6.487646000	3.901634000
C	5.345923000	6.196611000	4.810287000
H	5.004774000	5.535531000	5.638146000
H	6.209595000	5.716126000	4.298343000
H	5.669855000	7.166159000	5.232031000
O	1.443619000	4.150944000	-8.736532000
C	2.028302000	3.493630000	-9.854630000
H	3.067204000	3.159223000	-9.637179000
H	1.425674000	2.614894000	-10.175588000
H	2.051326000	4.235633000	-10.674064000
C	-5.709384000	-2.991984000	1.647259000
C	-4.953881000	-3.997186000	2.288521000
C	-6.914057000	-3.364277000	1.011760000
C	-5.382912000	-5.335036000	2.292923000
C	-7.347026000	-4.701128000	1.012561000
C	-6.581533000	-5.693664000	1.651828000
H	-4.018761000	-3.720954000	2.797367000
H	-7.522493000	-2.593478000	0.510079000
H	-4.775198000	-6.100133000	2.802829000
H	-8.291123000	-4.971034000	0.512562000
H	-6.921962000	-6.741279000	1.652966000
C	-5.200361000	-1.568811000	1.605748000
H	-5.996567000	-0.861783000	1.928615000
H	-4.375640000	-1.471200000	2.341646000
C	-4.671520000	-1.150718000	0.217524000
H	-4.074979000	-1.987746000	-0.205190000
H	-5.522038000	-0.990839000	-0.482910000
C	-3.781535000	0.091887000	0.289570000
H	-4.316141000	0.941511000	0.767851000
H	-2.914828000	-0.132562000	0.945036000
C	-3.227712000	0.550262000	-1.081403000
H	-3.070339000	-0.343404000	-1.731073000
H	-3.985336000	1.188228000	-1.600418000
N	-1.953050000	1.209473000	-1.019185000
N	-2.191633000	2.607395000	-0.090716000
N	-1.325482000	3.293182000	0.247328000

Structure of ^{2}Int2

Fe	-0.342114000	0.271983000	-0.843594000
N	0.235480000	0.301853000	1.013124000
N	-0.826050000	-1.562875000	-0.576049000
C	-1.535218000	-2.450391000	3.025825000
C	-1.231630000	-2.256676000	0.551199000
C	-0.987026000	-1.737224000	1.844574000
C	-0.253193000	-0.532427000	2.028053000
C	-1.320924000	-3.832027000	3.224403000
C	0.871485000	1.171397000	3.052843000
H	1.326639000	1.845021000	3.786853000
C	-1.141530000	-2.303034000	-1.706197000
C	0.162636000	0.010987000	3.299233000
H	-0.040795000	-0.449602000	4.272278000
C	-2.350867000	-1.770791000	3.968190000
C	-1.912173000	-4.524910000	4.293984000
H	-1.716191000	-5.599576000	4.409420000
C	-1.855672000	-3.479173000	0.103690000
H	-2.299062000	-4.242622000	0.751353000
C	-1.786260000	-3.509655000	-1.293893000
H	-2.148240000	-4.306363000	-1.954752000
C	-2.741872000	-3.834378000	5.204758000
C	-2.943467000	-2.444466000	5.037861000
H	-3.595692000	-1.919890000	5.751637000
C	0.915949000	1.357507000	1.622613000
N	-0.145671000	-0.411702000	-2.624498000
N	0.881686000	1.718530000	-1.346890000
C	0.983907000	2.178774000	-5.147040000
C	1.133237000	2.166456000	-2.643376000
C	3.353504000	3.114183000	2.556462000
C	0.727556000	1.516344000	-3.843372000
C	0.108483000	0.246297000	-3.818915000
C	2.252569000	3.461564000	1.745939000
C	0.518865000	3.495315000	-5.396095000
C	-0.886016000	-1.764724000	-4.314595000
H	-1.319296000	-2.624543000	-4.839887000
C	1.525572000	2.578783000	-0.467298000
C	1.858095000	4.821759000	1.711165000
C	-0.357082000	-0.603762000	-4.887571000
H	-0.299742000	-0.362767000	-5.955191000
C	1.684887000	1.522111000	-6.181808000
C	1.534565000	2.432814000	0.941575000
C	4.043980000	4.075821000	3.312739000
H	4.903148000	3.763426000	3.921968000
C	0.738944000	4.121243000	-6.624784000
H	0.367653000	5.137979000	-6.822823000
C	1.971573000	3.340694000	-2.566085000
H	2.366583000	3.884605000	-3.431568000
C	3.633866000	5.426396000	3.268958000
C	2.220800000	3.590214000	-1.228793000
H	2.848016000	4.379658000	-0.800193000
C	2.532628000	5.790072000	2.459383000
H	2.221510000	6.845434000	2.441551000
C	1.439031000	3.448026000	-7.653229000
C	1.916073000	2.139024000	-7.422346000
H	2.477331000	1.595805000	-8.194971000
C	-0.762778000	-1.623614000	-2.897281000
H	3.689209000	2.065962000	2.583712000
H	0.995899000	5.114660000	1.092326000
H	-0.664027000	-4.378516000	2.531283000
H	-2.539539000	-0.694657000	3.835579000
H	2.080979000	0.510638000	-6.003619000
H	-0.043285000	4.022427000	-4.609967000
O	-3.380333000	-4.411718000	6.258325000
C	-3.267795000	-5.819247000	6.423254000
H	-3.656426000	-6.365949000	5.535038000
H	-2.215285000	-6.130954000	6.607559000
H	-3.881133000	-6.079679000	7.305665000
O	4.229717000	6.437547000	3.961661000
C	5.340598000	6.122615000	4.790462000
H	5.065543000	5.401284000	5.592531000
H	6.186843000	5.700575000	4.203079000
H	5.662503000	7.073278000	5.254906000
O	1.602750000	4.136192000	-8.817461000
C	2.283091000	3.495725000	-9.888750000
H	3.332973000	3.242822000	-9.618674000
H	1.761120000	2.566722000	-10.210637000
H	2.290307000	4.215753000	-10.728113000
C	-5.821495000	-2.859378000	1.862024000
C	-4.951514000	-3.879594000	2.305702000
C	-7.142994000	-3.208952000	1.513936000
C	-5.389209000	-5.209736000	2.402531000
C	-7.585086000	-4.540624000	1.607185000
C	-6.709169000	-5.546770000	2.051916000
H	-3.920557000	-3.621517000	2.587489000
H	-7.836686000	-2.424029000	1.169131000
H	-4.692889000	-5.986142000	2.758668000
H	-8.622159000	-4.793476000	1.334219000
H	-7.056330000	-6.589341000	2.128668000
C	-5.309242000	-1.443340000	1.721035000
H	-6.127114000	-0.713732000	1.905834000
H	-4.538026000	-1.266144000	2.499905000
C	-4.675060000	-1.180855000	0.339212000
H	-4.022441000	-2.041138000	0.075486000
H	-5.467445000	-1.140071000	-0.441028000
C	-3.823599000	0.091859000	0.319531000
H	-4.414256000	0.986838000	0.607696000
H	-3.017266000	-0.011560000	1.074902000
C	-3.159789000	0.325862000	-1.071679000

H	-3.094203000	-0.643437000	-1.625159000
H	-3.769360000	1.025090000	-1.690330000
N	-1.859971000	0.895424000	-0.896790000

Structure of ^{4}Int2

Fe	-0.171437000	0.195798000	-0.843686000
N	0.196296000	0.347602000	1.051341000
N	-0.651728000	-1.641531000	-0.579915000
C	-1.562120000	-2.429576000	2.998833000
C	-1.135485000	-2.290296000	0.539796000
C	-0.988586000	-1.722107000	1.833604000
C	-0.300291000	-0.479715000	2.049124000
C	-1.360864000	-3.814489000	3.204044000
C	0.850778000	1.198528000	3.086087000
H	1.317594000	1.856361000	3.826910000
C	-0.936582000	-2.389203000	-1.704538000
C	0.111558000	0.053024000	3.328830000
H	-0.098976000	-0.403998000	4.301937000
C	-2.386563000	-1.740202000	3.929008000
C	-1.972393000	-4.499253000	4.265142000
H	-1.782198000	-5.573816000	4.390038000
C	-1.757040000	-3.515495000	0.091318000
H	-2.263143000	-4.249388000	0.727485000
C	-1.614084000	-3.581026000	-1.301746000
H	-1.969226000	-4.380246000	-1.963232000
C	-2.816882000	-3.800578000	5.157256000
C	-3.006024000	-2.408757000	4.984456000
H	-3.671193000	-1.879909000	5.682728000
C	0.909696000	1.375280000	1.651954000
N	-0.049989000	-0.460578000	-2.661717000
N	0.918634000	1.715856000	-1.352584000
C	0.961158000	2.191491000	-5.143413000
C	1.137147000	2.172580000	-2.644027000
C	3.325238000	3.113404000	2.613406000
C	0.724754000	1.509903000	-3.850785000
C	0.151152000	0.212273000	-3.845977000
C	2.270133000	3.463646000	1.742967000
C	0.508981000	3.520465000	-5.357331000
C	-0.724483000	-1.853083000	-4.336750000
H	-1.116200000	-2.736004000	-4.856408000
C	1.570504000	2.566097000	-0.472041000
C	1.916457000	4.833528000	1.646450000
C	-0.282948000	-0.659027000	-4.918205000
H	-0.263524000	-0.417600000	-5.987125000
C	1.632043000	1.544800000	-6.206420000
C	1.556890000	2.430402000	0.945827000
C	4.011468000	4.081097000	3.364083000
H	4.837627000	3.767227000	4.016618000
C	0.707184000	4.163502000	-6.579466000
H	0.340823000	5.186400000	-6.752963000
C	1.978022000	3.347831000	-2.573517000
H	2.359884000	3.902332000	-3.437971000
C	3.640965000	5.440354000	3.259629000
C	2.256986000	3.582589000	-1.237521000
H	2.898843000	4.365571000	-0.818758000
C	2.583835000	5.806344000	2.393235000
H	2.300819000	6.867841000	2.331355000
C	1.378771000	3.500797000	-7.634755000
C	1.846953000	2.182332000	-7.437753000
H	2.389429000	1.648925000	-8.230262000
C	-0.582942000	-1.697701000	-2.917770000
H	3.632168000	2.058847000	2.687338000
H	1.086663000	5.127837000	0.985925000
H	-0.690634000	-4.364728000	2.527302000
H	-2.566957000	-0.664017000	3.789366000
H	2.023469000	0.527845000	-6.053148000
H	-0.031689000	4.038585000	-4.550762000
O	-3.478602000	-4.370180000	6.197282000
C	-3.381113000	-5.778953000	6.370548000
H	-3.755190000	-6.324675000	5.476132000
H	-2.335352000	-6.096861000	6.579343000
H	-4.015869000	-6.029594000	7.240321000
O	4.234835000	6.456497000	3.943097000
C	5.299941000	6.141627000	4.831382000
H	4.969489000	5.455437000	5.642899000
H	6.158991000	5.678994000	4.295878000
H	5.625939000	7.099256000	5.277965000
O	1.526571000	4.207476000	-8.787343000
C	2.176164000	3.580786000	-9.886938000
H	3.228473000	3.311837000	-9.644355000
H	1.635787000	2.664819000	-10.214623000
H	2.172685000	4.317930000	-10.711095000
C	-5.856074000	-2.843964000	1.882688000
C	-4.972050000	-3.872923000	2.277226000
C	-7.203464000	-3.178704000	1.634702000
C	-5.419923000	-5.194442000	2.424813000
C	-7.657005000	-4.502438000	1.778800000
C	-6.766855000	-5.515923000	2.175037000
H	-3.919329000	-3.626615000	2.478504000
H	-7.908072000	-2.387862000	1.327854000
H	-4.711179000	-5.977327000	2.740046000
H	-8.714334000	-4.743041000	1.583164000
H	-7.122662000	-6.551929000	2.291391000
C	-5.328929000	-1.439553000	1.693325000
H	-6.146920000	-0.697838000	1.819202000
H	-4.584775000	-1.232404000	2.491480000
C	-4.639383000	-1.238526000	0.327444000
H	-4.012628000	-2.131102000	0.108269000
H	-5.406719000	-1.199777000	-0.478370000
C	-3.737503000	-0.001992000	0.286172000
H	-4.302035000	0.916003000	0.560708000
H	-2.941430000	-0.115285000	1.051508000
C	-3.044402000	0.195395000	-1.089630000
H	-2.963347000	-0.797686000	-1.602641000
H	-3.693404000	0.813323000	-1.759844000
N	-1.763529000	0.818845000	-1.008407000

Structure of ^{6}Int2

Fe	-0.464982000	0.493234000	-1.006368000
N	0.057534000	0.424784000	0.912268000
N	-0.626369000	-1.607197000	-0.780882000
C	-1.538294000	-2.506725000	2.758162000
C	-1.135511000	-2.281333000	0.302020000
C	-0.994139000	-1.748028000	1.615406000
C	-0.354497000	-0.476443000	1.873443000
C	-1.348193000	-3.905826000	2.873390000
C	0.733999000	1.217123000	2.955995000
H	1.179512000	1.866636000	3.717025000
C	-1.026902000	-2.224750000	-1.924379000
C	0.072957000	0.015313000	3.164898000
H	-0.065166000	-0.501406000	4.121160000
C	-2.305338000	-1.865253000	3.770044000
C	-1.910929000	-4.643917000	3.924135000
H	-1.729512000	-5.725980000	3.977561000
C	-1.859291000	-3.441625000	-0.195245000
H	-2.391240000	-4.187093000	0.407518000
C	-1.778494000	-3.407703000	-1.590969000
H	-2.224890000	-4.120593000	-2.295425000
C	-2.699729000	-3.988940000	4.897412000
C	-2.878355000	-2.587339000	4.815051000
H	-3.505204000	-2.095200000	5.572989000
C	0.747020000	1.455084000	1.528581000
N	-0.203897000	-0.232319000	-2.851790000
N	0.827577000	1.907354000	-1.499560000

C	1.178403000	2.305082000	-5.275798000
C	1.193435000	2.306379000	-2.770163000
C	3.144920000	3.168627000	2.565746000
C	0.794957000	1.660971000	-4.000077000
C	0.125992000	0.409099000	-4.022751000
C	2.117107000	3.539127000	1.670279000
C	0.870611000	3.669533000	-5.523594000
C	-0.770496000	-1.639198000	-4.548660000
H	-1.126799000	-2.529375000	-5.081671000
C	1.477892000	2.700568000	-0.574699000
C	1.799668000	4.917607000	1.559202000
C	-0.259862000	-0.466774000	-5.115940000
H	-0.156116000	-0.247359000	-6.185086000
C	1.862258000	1.591004000	-6.286684000
C	1.401516000	2.530906000	0.845649000
C	3.833515000	4.123260000	3.330075000
H	4.639246000	3.793839000	4.000250000
C	1.215104000	4.281319000	-6.728949000
H	0.959572000	5.332596000	-6.929257000
C	2.123084000	3.406508000	-2.646275000
H	2.624035000	3.911226000	-3.480034000
C	3.493906000	5.490048000	3.215653000
C	2.301963000	3.649256000	-1.290523000
H	2.973980000	4.383001000	-0.831376000
C	2.467732000	5.877234000	2.321369000
H	2.210264000	6.944545000	2.249550000
C	1.895453000	3.550456000	-7.732372000
C	2.223290000	2.195900000	-7.499908000
H	2.771673000	1.609303000	-8.249590000
C	-0.716450000	-1.462912000	-3.122379000
H	3.432820000	2.109317000	2.644095000
H	0.996348000	5.228438000	0.874034000
H	-0.718900000	-4.421393000	2.133024000
H	-2.481762000	-0.781813000	3.700918000
H	2.150640000	0.545420000	-6.101934000
H	0.325230000	4.243583000	-4.759242000
O	-3.319865000	-4.609563000	5.932010000
C	-3.244933000	-6.028166000	6.015921000
H	-3.678050000	-6.509445000	5.111727000
H	-2.197174000	-6.377505000	6.150144000
H	-3.840035000	-6.318255000	6.901214000
O	4.091795000	6.493955000	3.912288000
C	5.127634000	6.158634000	4.827883000
H	4.762617000	5.479611000	5.630420000
H	5.990626000	5.679051000	4.314288000
H	5.460651000	7.110024000	5.282430000
O	2.191331000	4.231094000	-8.871708000
C	2.857957000	3.538426000	-9.920733000
H	3.858663000	3.171900000	-9.600308000
H	2.258208000	2.676162000	-10.288275000
H	2.985958000	4.267419000	-10.742161000
C	-5.995328000	-2.889689000	2.422527000
C	-5.099374000	-3.980503000	2.491822000
C	-7.364997000	-3.136965000	2.647322000
C	-5.556442000	-5.274537000	2.782246000
C	-7.828865000	-4.432837000	2.938944000
C	-6.925820000	-5.507296000	3.008522000
H	-4.025544000	-3.806565000	2.325717000
H	-8.078489000	-2.297788000	2.596891000
H	-4.837266000	-6.108374000	2.830259000
H	-8.903440000	-4.603158000	3.112863000
H	-7.287361000	-6.522293000	3.237482000
C	-5.468547000	-1.505218000	2.114675000
H	-6.276521000	-0.752831000	2.238685000
H	-4.689258000	-1.256072000	2.867243000
C	-4.838023000	-1.382862000	0.714316000
H	-4.207896000	-2.278189000	0.519027000
H	-5.638963000	-1.404327000	-0.058849000
C	-3.955329000	-0.143817000	0.547805000
H	-4.511292000	0.786260000	0.798963000
H	-3.107237000	-0.202917000	1.261826000
C	-3.359509000	-0.032420000	-0.877844000
H	-3.187364000	-1.066659000	-1.278608000
H	-4.121672000	0.408344000	-1.572039000
N	-2.167204000	0.723783000	-0.963479000

Structure of ^{2}TS2

Fe	-0.489105000	0.433098000	-0.241261000
N	0.219422000	0.526077000	1.558317000
N	-1.077559000	-1.355283000	0.105069000
C	-1.063578000	-2.366479000	3.747138000
C	-1.309252000	-2.037677000	1.286517000
C	-0.827830000	-1.547540000	2.527787000
C	-0.105287000	-0.325307000	2.626004000
C	-0.662937000	-3.720269000	3.790945000
C	1.081013000	1.424036000	3.494514000
H	1.570702000	2.128130000	4.173888000
C	-1.634812000	-2.063347000	-0.954277000
C	0.455823000	0.237722000	3.830481000
H	0.388352000	-0.212253000	4.826359000
C	-1.700264000	-1.836404000	4.896632000
C	-0.885165000	-4.522028000	4.922670000
H	-0.545557000	-5.566920000	4.914090000
C	-2.067764000	-3.220160000	0.958789000
H	-2.424747000	-3.963680000	1.680732000
C	-2.262409000	-3.234648000	-0.430187000
H	-2.787563000	-4.000905000	-1.013583000
C	-1.525597000	-3.973740000	6.055425000
C	-1.930326000	-2.619162000	6.030350000
H	-2.434188000	-2.205658000	6.916948000
C	0.954651000	1.595741000	2.067714000
N	-0.701207000	-0.223682000	-2.033969000
N	0.700979000	1.813788000	-0.921140000
C	0.440909000	2.162401000	-4.733971000
C	0.823396000	2.219341000	-2.254778000
C	3.286486000	3.553441000	2.836355000
C	0.248865000	1.569355000	-3.383933000
C	-0.488091000	0.363474000	-3.266383000
C	2.180276000	3.770516000	1.988659000
C	0.032017000	3.490017000	-5.017400000
C	-1.675524000	-1.576660000	-3.598149000
H	-2.210794000	-2.418117000	-4.055195000
C	1.399478000	2.718900000	-0.129065000
C	1.723245000	5.103402000	1.833346000
C	-1.112604000	-0.477677000	-4.260127000
H	-1.130108000	-0.279338000	-5.338111000
C	1.027124000	1.418125000	-5.780244000
C	1.491147000	2.652509000	1.286126000
C	3.908964000	4.608946000	3.524070000
H	4.772737000	4.393757000	4.168066000
C	0.202067000	4.045527000	-6.287845000
H	-0.123961000	5.072653000	-6.510842000
C	1.677191000	3.385635000	-2.291746000
H	2.001420000	3.895328000	-3.206119000
C	3.428662000	5.927388000	3.367164000
C	2.029253000	3.694441000	-0.990021000
H	2.686326000	4.503218000	-0.651247000
C	2.330996000	6.165339000	2.507579000
H	1.968842000	7.198134000	2.392843000
C	0.792149000	3.286711000	-7.325285000
C	1.206357000	1.962631000	-7.062957000
H	1.678542000	1.350973000	-7.844043000
C	-1.409879000	-1.406094000	-2.202761000
H	3.682869000	2.532950000	2.949931000
H	0.860725000	5.299129000	1.178043000
H	-0.144570000	-4.152325000	2.921369000
H	-2.028051000	-0.787899000	4.889132000
H	1.372009000	0.392199000	-5.579312000
H	-0.443633000	4.085120000	-4.222719000
O	-1.792144000	-4.660207000	7.202331000
C	-1.408488000	-6.026935000	7.272587000

H	-1.914958000	-6.637935000	6.491912000
H	-0.307306000	-6.152206000	7.167914000
H	-1.719154000	-6.387217000	8.270992000
O	3.953420000	7.021236000	3.988762000
C	5.057454000	6.832502000	4.863463000
H	4.799837000	6.161688000	5.713840000
H	5.939573000	6.412384000	4.330005000
H	5.317277000	7.832412000	5.258203000
O	0.917716000	3.912186000	-8.530217000
C	1.501943000	3.189636000	-9.605253000
H	2.549452000	2.888037000	-9.378896000
H	0.913503000	2.279115000	-9.858615000
H	1.503379000	3.872038000	-10.475625000
C	-2.196136000	2.573481000	3.266610000
C	-2.189269000	1.942773000	4.539465000
C	-1.424591000	3.759563000	3.112848000
C	-1.476208000	2.490251000	5.614848000
C	-0.689976000	4.288288000	4.179184000
C	-0.719996000	3.661708000	5.440847000
H	-2.799682000	1.041839000	4.695947000
H	-1.392654000	4.249936000	2.126749000
H	-1.507465000	1.992339000	6.596792000
H	-0.078868000	5.190321000	4.024950000
H	-0.148370000	4.084516000	6.281523000
C	-2.977783000	2.053011000	2.145112000
H	-3.513006000	2.842488000	1.577892000
H	-2.090670000	1.846536000	1.009913000
C	-3.665612000	0.709723000	2.200528000
H	-2.999486000	0.000369000	2.732720000
H	-4.596108000	0.781546000	2.810836000
C	-4.015653000	0.120797000	0.803093000
H	-5.109893000	0.143760000	0.619099000
H	-3.708801000	-0.943412000	0.771098000
C	-3.330557000	0.892862000	-0.352290000
H	-3.321983000	0.276420000	-1.276573000
H	-3.913067000	1.818262000	-0.575138000
N	-1.972090000	1.310127000	-0.051977000

Structure of ^{4}TS2

Fe	-0.192666000	0.133816000	-0.147505000
N	0.374718000	0.267162000	1.686418000
N	-0.571336000	-1.740768000	0.118721000
C	-1.081577000	-2.593057000	3.763560000
C	-0.948049000	-2.397402000	1.272075000
C	-0.662913000	-1.848097000	2.554056000
C	0.024303000	-0.607674000	2.717132000
C	-0.758049000	-3.957234000	3.940166000
C	1.257918000	1.072581000	3.654732000
H	1.789061000	1.727754000	4.352691000
C	-1.000673000	-2.458343000	-0.980989000
C	0.582384000	-0.097844000	3.950536000
H	0.499579000	-0.587522000	4.927297000
C	-1.839962000	-1.957165000	4.781423000
C	-1.165764000	-4.669525000	5.078721000
H	-0.877043000	-5.724370000	5.183313000
C	-1.633663000	-3.603457000	0.879816000
H	-2.066409000	-4.343181000	1.563278000
C	-1.652419000	-3.644500000	-0.524135000
H	-2.091860000	-4.426870000	-1.154952000
C	-1.925339000	-4.019724000	6.077340000
C	-2.259190000	-2.653985000	5.915327000
H	-2.860906000	-2.165229000	6.696148000
C	1.127922000	1.301273000	2.232835000
N	-0.261190000	-0.484290000	-1.980617000
N	0.732428000	1.715290000	-0.727415000
C	0.284497000	2.286914000	-4.483703000
C	0.763260000	2.220540000	-2.026342000
C	3.506454000	3.199675000	2.977528000
C	0.254393000	1.563699000	-3.190970000
C	-0.242625000	0.232736000	-3.155658000
C	2.338743000	3.480812000	2.236787000
C	-0.275854000	3.585417000	-4.605984000
C	-1.138711000	-1.837413000	-3.594079000
H	-1.581254000	-2.710411000	-4.089216000
C	1.430907000	2.590504000	0.096949000
C	1.867915000	4.816783000	2.221418000
C	-0.798361000	-0.613122000	-4.187346000
H	-0.934370000	-0.332711000	-5.238416000
C	0.862122000	1.708696000	-5.636561000
C	1.613156000	2.415091000	1.495668000
C	4.188217000	4.203127000	3.685561000
H	5.100600000	3.943812000	4.239855000
C	-0.265225000	4.266687000	-5.823871000
H	-0.713357000	5.266735000	-5.923223000
C	1.523937000	3.450962000	-2.012866000
H	1.760876000	4.052984000	-2.897255000
C	3.699852000	5.528323000	3.663720000
C	1.943356000	3.672719000	-0.713382000
H	2.573148000	4.490975000	-0.347551000
C	2.532302000	5.824634000	2.922376000
H	2.157421000	6.859124000	2.925634000
C	0.317162000	3.673204000	-6.969076000
C	0.886337000	2.384356000	-6.866681000
H	1.361262000	1.904101000	-7.733180000
C	-0.810438000	-1.727230000	-2.205760000
H	3.903581000	2.172940000	2.985276000
H	0.944074000	5.051465000	1.673483000
H	-0.146089000	-4.465091000	3.179408000
H	-2.123055000	-0.901064000	4.657614000
H	1.331297000	0.716129000	-5.559977000
H	-0.747923000	4.049443000	-3.726641000
O	-2.374616000	-4.615272000	7.215353000
C	-2.075086000	-5.990095000	7.422695000
H	-2.505109000	-6.629973000	6.620025000
H	-0.977848000	-6.168369000	7.476768000
H	-2.534032000	-6.265936000	8.390171000
O	4.277987000	6.575244000	4.316914000
C	5.452864000	6.328798000	5.077970000
H	5.267572000	5.598585000	5.897556000
H	6.284424000	5.950785000	4.441795000
H	5.748125000	7.299148000	5.518786000
O	0.283022000	4.413841000	-8.110541000
C	0.847069000	3.859834000	-9.292558000
H	1.935008000	3.657274000	-9.173587000
H	0.335454000	2.917038000	-9.589437000
H	0.705182000	4.614458000	-10.088314000
C	-2.318410000	3.202349000	2.429443000
C	-1.519238000	2.895311000	3.562494000
C	-2.392952000	4.563978000	2.024772000
C	-0.846942000	3.902713000	4.266329000
C	-1.726713000	5.570670000	2.734864000
C	-0.952374000	5.246815000	3.866226000
H	-1.414202000	1.851719000	3.890295000
H	-2.997149000	4.823227000	1.139810000
H	-0.225952000	3.634130000	5.135193000
H	-1.810701000	6.618786000	2.405659000
H	-0.421254000	6.034570000	4.421901000
C	-3.025580000	2.166665000	1.661162000
H	-3.835161000	2.581755000	1.025417000
H	-2.193995000	1.710039000	0.759487000
C	-3.360408000	0.819747000	2.285340000
H	-2.449106000	0.440675000	2.791124000
H	-4.139024000	0.923691000	3.074909000
C	-3.801393000	-0.226646000	1.235828000
H	-4.904102000	-0.211003000	1.097079000
H	-3.538193000	-1.239574000	1.602118000
C	-3.130848000	0.022266000	-0.150427000
H	-3.006611000	-0.936984000	-0.697929000
H	-3.807469000	0.647020000	-0.784333000
N	-1.864942000	0.700682000	-0.032008000

Structure of ^{6}TS2

Fe	-0.173066000	0.121898000	-0.139151000	H	2.164730000	6.859630000	2.941975000
N	0.361065000	0.278536000	1.704284000	C	0.299954000	3.660157000	-6.954684000
N	-0.562270000	-1.727751000	0.156456000	C	0.867683000	2.370287000	-6.853606000
C	-1.074564000	-2.586843000	3.796251000	H	1.334044000	1.886746000	-7.722926000
C	-0.927416000	-2.397890000	1.301843000	C	-0.762068000	-1.736150000	-2.169779000
C	-0.660728000	-1.849382000	2.582180000	H	3.913232000	2.174099000	2.993677000
C	0.025390000	-0.601789000	2.732844000	H	0.950304000	5.053184000	1.689545000
C	-0.751249000	-3.950000000	3.981835000	H	-0.138283000	-4.462871000	3.225205000
C	1.279300000	1.065094000	3.656626000	H	-2.113947000	-0.887917000	4.680389000
H	1.825072000	1.712225000	4.350709000	H	1.320461000	0.704822000	-5.546998000
C	-0.962688000	-2.453121000	-0.946180000	H	-0.739869000	4.045864000	-3.705143000
C	0.595941000	-0.104298000	3.958777000	O	-2.362880000	-4.584521000	7.264220000
H	0.519236000	-0.596076000	4.934701000	C	-2.066576000	-5.958842000	7.479103000
C	-1.830348000	-1.943070000	4.811638000	H	-2.499498000	-6.602275000	6.680826000
C	-1.158582000	-4.654522000	5.125397000	H	-0.969709000	-6.139684000	7.532555000
H	-0.870333000	-5.708806000	5.236776000	H	-2.524699000	-6.227875000	8.448883000
C	-1.589244000	-3.617671000	0.900252000	O	4.285384000	6.574004000	4.332925000
H	-2.017949000	-4.363284000	1.579879000	C	5.459782000	6.326630000	5.094465000
C	-1.593839000	-3.656853000	-0.500811000	H	5.273907000	5.595362000	5.912968000
H	-2.019154000	-4.440580000	-1.139228000	H	6.291770000	5.949509000	4.458331000
C	-1.915176000	-3.997165000	6.121300000	H	5.754681000	7.296434000	5.536702000
C	-2.247013000	-2.631821000	5.951280000	O	0.257324000	4.397355000	-8.097887000
H	-2.846298000	-2.137205000	6.730288000	C	0.811118000	3.839527000	-9.283042000
C	1.127578000	1.301558000	2.242997000	H	1.899655000	3.635724000	-9.172161000
N	-0.213217000	-0.488567000	-1.952520000	H	0.295726000	2.896785000	-9.573412000
N	0.729193000	1.723429000	-0.707067000	H	0.663935000	4.592389000	-10.079487000
C	0.287245000	2.281205000	-4.464811000	C	-2.308901000	3.179531000	2.400222000
C	0.761920000	2.223198000	-2.006650000	C	-1.508619000	2.825674000	3.515238000
C	3.513935000	3.200004000	2.989645000	C	-2.386399000	4.555682000	2.054641000
C	0.266827000	1.562860000	-3.170967000	C	-0.831636000	3.802281000	4.258797000
C	-0.220065000	0.226669000	-3.129000000	C	-1.714507000	5.532275000	2.801576000
C	2.345251000	3.481244000	2.249594000	C	-0.934789000	5.161191000	3.914324000
C	-0.273614000	3.580010000	-4.586532000	H	-1.408027000	1.769816000	3.802258000
C	-1.113859000	-1.847187000	-3.549504000	H	-2.995591000	4.852422000	1.184869000
H	-1.576039000	-2.715980000	-4.033815000	H	-0.210379000	3.496668000	5.115176000
C	1.428778000	2.592993000	0.111077000	H	-1.798677000	6.593135000	2.515955000
C	1.875084000	4.818237000	2.235753000	H	-0.398845000	5.924555000	4.498774000
C	-0.788498000	-0.619468000	-4.147945000	C	-3.025049000	2.174307000	1.585396000
H	-0.946110000	-0.338701000	-5.195815000	H	-3.898806000	2.617076000	1.060338000
C	0.852661000	1.698191000	-5.621785000	H	-2.316001000	1.850165000	0.602422000
C	1.617739000	2.418610000	1.508986000	C	-3.309841000	0.788510000	2.152617000
C	4.195436000	4.202637000	3.698923000	H	-2.373613000	0.404593000	2.604764000
H	5.108729000	3.943057000	4.251617000	H	-4.055786000	0.840426000	2.977429000
C	-0.272311000	4.257555000	-5.806394000	C	-3.778686000	-0.219434000	1.074258000
H	-0.721048000	5.257384000	-5.905150000	H	-4.886790000	-0.296808000	1.051787000
C	1.517215000	3.456826000	-1.996351000	H	-3.392746000	-1.226846000	1.330040000
H	1.749577000	4.059542000	-2.881312000	C	-3.294258000	0.175057000	-0.362066000
C	3.707089000	5.527930000	3.678741000	H	-3.242280000	-0.743123000	-0.997038000
C	1.938276000	3.678628000	-0.697769000	H	-4.074232000	0.831272000	-0.830302000
H	2.564824000	4.499028000	-0.331143000	N	-2.035936000	0.838638000	-0.360349000
C	2.539757000	5.825187000	2.937338000				

Structure of ^{2}Int3

Fe	-0.360305000	0.469940000	-0.893495000	H	-2.929649000	-5.351516000	3.905904000
N	-0.195729000	0.452003000	1.038500000	C	-2.492274000	-3.062178000	-0.369117000
N	-1.189096000	-1.247246000	-0.809427000	H	-3.096134000	-3.794453000	0.179033000
C	-2.488760000	-2.191849000	2.609217000	C	-2.254060000	-3.042442000	-1.749867000
C	-1.808303000	-1.932990000	0.216967000	H	-2.616916000	-3.764719000	-2.491026000
C	-1.724447000	-1.474100000	1.556966000	C	-4.020284000	-3.571063000	4.571814000
C	-0.939106000	-0.343236000	1.918399000	C	-4.180410000	-2.177880000	4.385094000
C	-2.338102000	-3.580607000	2.809790000	H	-4.916386000	-1.647186000	5.007696000
C	0.123972000	1.237002000	3.182559000	C	0.453913000	1.429221000	1.789197000
H	0.489646000	1.857410000	4.008106000	N	-0.089994000	-0.124494000	-2.693180000
C	-1.433811000	-1.903465000	-2.016118000	N	1.016105000	1.789740000	-1.145064000
C	-0.720374000	0.144650000	3.261698000	C	1.801817000	2.318500000	-4.864470000
H	-1.145808000	-0.300666000	4.167909000	C	1.521136000	2.242323000	-2.372900000
C	-3.426628000	-1.504371000	3.421940000	C	2.810956000	2.978000000	3.188359000
C	-3.087751000	-4.272078000	3.776209000	C	1.255274000	1.665330000	-3.647827000

C	0.482819000	0.485704000	-3.791502000
C	1.919436000	3.410420000	2.184555000
C	1.520101000	3.679238000	-5.147131000
C	-0.684614000	-1.351830000	-4.531373000
H	-1.129778000	-2.133730000	-5.158523000
C	1.557555000	2.589215000	-0.138887000
C	1.647503000	4.797803000	2.092583000
C	0.103381000	-0.276648000	-4.959427000
H	0.385634000	-0.037576000	-5.991165000
C	2.596895000	1.605275000	-5.787194000
C	1.285692000	2.441357000	1.245728000
C	3.416465000	3.883661000	4.075592000
H	4.112288000	3.505102000	4.836776000
C	2.010563000	4.296233000	-6.299908000
H	1.783465000	5.349064000	-6.525414000
C	2.425208000	3.337382000	-2.111277000
H	3.007052000	3.864217000	-2.875976000
C	3.132046000	5.262749000	3.969909000
C	2.453734000	3.545953000	-0.743193000
H	3.053948000	4.279486000	-0.193553000
C	2.239909000	5.711327000	2.968280000
H	2.022466000	6.788154000	2.904475000
C	2.806973000	3.568240000	-7.214769000
C	3.099583000	2.212239000	-6.949934000
H	3.725380000	1.623708000	-7.634746000
C	-0.801931000	-1.245373000	-3.110364000
H	3.049844000	1.906149000	3.268118000
H	0.944969000	5.156336000	1.324406000
H	-1.599318000	-4.132208000	2.208395000
H	-3.581333000	-0.426119000	3.266939000
H	2.847774000	0.553976000	-5.578480000
H	0.886765000	4.250549000	-4.451129000
O	-4.799922000	-4.140758000	5.532683000
C	-4.680800000	-5.539495000	5.755785000
H	-4.940186000	-6.125574000	4.845612000
H	-3.654163000	-5.818965000	6.083019000
H	-5.396229000	-5.788572000	6.561470000
O	3.661959000	6.225029000	4.776729000
C	4.563958000	5.824294000	5.799230000
H	4.082376000	5.132454000	6.526502000
H	5.469562000	5.330849000	5.380166000
H	4.867732000	6.747734000	6.326598000
O	3.241137000	4.253187000	-8.309310000
C	4.045095000	3.566761000	-9.259648000
H	4.997200000	3.205926000	-8.809691000
H	3.507325000	2.700228000	-9.705904000
H	4.276981000	4.297149000	-10.056992000
C	-5.271352000	0.861646000	-1.211413000
C	-4.746015000	-0.370098000	-0.688531000
C	-6.244899000	1.547892000	-0.407102000
C	-5.162691000	-0.861232000	0.550000000
C	-6.647458000	1.049108000	0.832209000
C	-6.108951000	-0.160015000	1.325908000
H	-4.018146000	-0.951920000	-1.273078000
H	-6.666819000	2.493553000	-0.784843000
H	-4.740420000	-1.805433000	0.924047000
H	-7.389214000	1.604138000	1.428649000
H	-6.421956000	-0.555037000	2.304565000
C	-4.859002000	1.413135000	-2.455641000
H	-5.354488000	2.348185000	-2.775272000
H	-2.687385000	1.106642000	-0.726582000
C	-3.753394000	0.904164000	-3.322610000
H	-3.331775000	-0.037179000	-2.917568000
H	-4.142235000	0.654680000	-4.337370000
C	-2.606293000	1.927254000	-3.496509000
H	-2.952870000	2.788676000	-4.110279000
H	-1.782064000	1.446200000	-4.060977000
C	-2.077873000	2.496059000	-2.159448000
H	-1.148200000	3.073678000	-2.352292000
H	-2.830594000	3.218930000	-1.761041000
N	-1.827261000	1.537552000	-1.104104000

Structure of ^{4}Int3

Fe	-0.357964000	0.460622000	-0.884703000
N	-0.188204000	0.442321000	1.047856000
N	-1.183838000	-1.256857000	-0.799362000
C	-2.479887000	-2.201235000	2.620900000
C	-1.804622000	-1.940511000	0.227786000
C	-1.715852000	-1.483571000	1.568194000
C	-0.928400000	-0.354128000	1.928938000
C	-2.330416000	-3.590446000	2.819625000
C	0.135240000	1.226923000	3.191448000
H	0.501829000	1.847685000	4.016313000
C	-1.434164000	-1.910975000	-2.006430000
C	-0.706854000	0.133049000	3.272162000
H	-1.129439000	-0.313490000	4.179116000
C	-3.416672000	-1.513905000	3.435129000
C	-3.079893000	-4.282377000	3.785806000
H	-2.922712000	-5.362127000	3.914011000
C	-2.495744000	-3.065094000	-0.358084000
H	-3.103077000	-3.794061000	0.190694000
C	-2.259881000	-3.045510000	-1.739464000
H	-2.628619000	-3.764898000	-2.480515000
C	-4.011333000	-3.581551000	4.582857000
C	-4.170468000	-2.188024000	4.397916000
H	-4.905500000	-1.657507000	5.021763000
C	0.460803000	1.420729000	1.797097000
N	-0.089688000	-0.133463000	-2.685429000
N	1.011902000	1.785255000	-1.139073000
C	1.792082000	2.313938000	-4.860087000
C	1.513789000	2.238809000	-2.368228000
C	2.816745000	2.976376000	3.189720000
C	1.249728000	1.659330000	-3.642393000
C	0.480966000	0.477151000	-3.784651000
C	1.920706000	3.406725000	2.189022000
C	1.503990000	3.673240000	-5.143278000
C	-0.688868000	-1.359277000	-4.523044000
H	-1.135752000	-2.140665000	-5.149611000
C	1.553569000	2.586246000	-0.133908000
C	1.644028000	4.793305000	2.099067000
C	0.099415000	-0.284740000	-4.952098000
H	0.380173000	-0.045580000	-5.984232000
C	2.588814000	1.603450000	-5.783489000
C	1.287129000	2.436250000	1.251584000
C	3.421988000	3.883238000	4.075896000
H	4.121383000	3.506301000	4.834604000
C	1.990366000	4.291633000	-6.297026000
H	1.758401000	5.343310000	-6.522954000
C	2.414403000	3.336909000	-2.108389000
H	2.993166000	3.865541000	-2.874192000
C	3.132719000	5.261473000	3.972315000
C	2.444968000	3.545862000	-0.740301000
H	3.044005000	4.281429000	-0.192100000
C	2.236122000	5.707998000	2.973748000
H	2.014954000	6.784161000	2.911561000
C	2.788828000	3.566512000	-7.212385000
C	3.087422000	2.211881000	-6.947221000
H	3.714703000	1.625600000	-7.632601000
C	-0.803305000	-1.253338000	-3.101665000
H	3.059429000	1.905261000	3.267818000
H	0.937933000	5.150253000	1.333422000
H	-1.592843000	-4.141980000	2.216665000
H	-3.570024000	-0.435079000	3.282342000
H	2.844181000	0.553261000	-5.574573000
H	0.868849000	4.242061000	-4.446877000
O	-4.790825000	-4.151750000	5.543470000
C	-4.673409000	-5.551032000	5.764190000
H	-4.933876000	-6.135206000	4.853113000
H	-3.647018000	-5.832355000	6.090601000
H	-5.388870000	-5.800528000	6.569712000
O	3.661861000	6.224766000	4.778418000

C	4.568045000	5.826082000	5.798026000
H	4.090791000	5.131995000	6.526011000
H	5.474092000	5.336005000	5.375982000
H	4.870249000	6.750043000	6.325381000
O	3.218774000	4.252703000	-8.307804000
C	4.024708000	3.569298000	-9.258646000
H	4.978758000	3.212645000	-8.809467000
H	3.490177000	2.700334000	-9.704070000
H	4.252756000	4.300348000	-10.056491000
C	-5.274558000	0.873825000	-1.264847000
C	-4.721494000	-0.333337000	-0.712842000
C	-6.267835000	1.553532000	-0.478839000
C	-5.129831000	-0.806177000	0.536114000
C	-6.661003000	1.073739000	0.770615000
C	-6.093803000	-0.110055000	1.293756000
H	-3.983851000	-0.914822000	-1.285399000
H	-6.712215000	2.479276000	-0.879452000
H	-4.688368000	-1.732951000	0.930693000
H	-7.417953000	1.623722000	1.352470000
H	-6.400073000	-0.489938000	2.280536000
C	-4.869608000	1.407101000	-2.518741000
H	-5.384909000	2.322548000	-2.862571000
H	-2.689808000	1.070429000	-0.714566000
C	-3.733950000	0.913907000	-3.355777000
H	-3.318409000	-0.029084000	-2.948123000
H	-4.087661000	0.674033000	-4.385092000
C	-2.588515000	1.946726000	-3.480338000
H	-2.926042000	2.817730000	-4.085502000
H	-1.748197000	1.481271000	-4.033631000
C	-2.093023000	2.494259000	-2.121952000
H	-1.174397000	3.097626000	-2.287627000
H	-2.867630000	3.190269000	-1.717968000
N	-1.834152000	1.517784000	-1.084709000

Structure of ^{6}Int3

Fe	-0.330877000	0.386519000	-0.923235000
N	-0.210915000	0.390999000	1.014249000
N	-1.193232000	-1.314554000	-0.832231000
C	-2.399161000	-2.334339000	2.587160000
C	-1.793933000	-2.013596000	0.184322000
C	-1.660525000	-1.592283000	1.538454000
C	-0.882965000	-0.452962000	1.897447000
C	-2.275749000	-3.736201000	2.713560000
C	0.083141000	1.198425000	3.146647000
H	0.410009000	1.846871000	3.966617000
C	-1.528938000	-1.881044000	-2.047710000
C	-0.674025000	0.044486000	3.239662000
H	-1.053193000	-0.427756000	4.152578000
C	-3.293783000	-1.668376000	3.466400000
C	-3.004422000	-4.457399000	3.672747000
H	-2.865561000	-5.544740000	3.745630000
C	-2.560428000	-3.078759000	-0.418780000
H	-3.168575000	-3.813368000	0.121408000
C	-2.392129000	-2.992871000	-1.810342000
H	-2.833074000	-3.647845000	-2.571015000
C	-3.892292000	-3.776683000	4.535153000
C	-4.029149000	-2.372829000	4.420582000
H	-4.734804000	-1.858861000	5.090380000
C	0.384879000	1.403506000	1.747132000
N	-0.102850000	-0.160387000	-2.738145000
N	1.003771000	1.755559000	-1.193130000
C	1.881896000	2.207802000	-4.893826000
C	1.571799000	2.154771000	-2.401920000
C	2.641662000	3.053272000	3.174346000
C	1.303623000	1.583309000	-3.681966000
C	0.472196000	0.438881000	-3.831137000
C	1.782377000	3.440610000	2.123423000
C	1.712690000	3.591534000	-5.161741000
C	-0.854759000	-1.286013000	-4.576600000
H	-1.384138000	-2.011650000	-5.205428000
C	1.493600000	2.574528000	-0.189506000
C	1.509638000	4.823475000	1.968086000
C	0.000832000	-0.259048000	-5.004868000
H	0.266061000	-0.014603000	-6.040025000
C	2.601247000	1.438747000	-5.836453000
C	1.188694000	2.440931000	1.195062000
C	3.211447000	3.995446000	4.046104000
H	3.884789000	3.650620000	4.842779000
C	2.237714000	4.175030000	-6.315774000
H	2.094550000	5.244590000	-6.530979000
C	2.482006000	3.245107000	-2.131802000
H	3.111293000	3.738726000	-2.880765000
C	2.922708000	5.367958000	3.878831000
C	2.431696000	3.507259000	-0.773735000
H	3.011013000	4.252304000	-0.217598000
C	2.064678000	5.772464000	2.829335000
H	1.843281000	6.844545000	2.717621000
C	2.960077000	3.391630000	-7.247026000
C	3.140245000	2.012624000	-6.998414000
H	3.708312000	1.381018000	-7.695098000
C	-0.905856000	-1.205407000	-3.151880000
H	2.889080000	1.987915000	3.299540000
H	0.833966000	5.148365000	1.161937000
H	-1.572109000	-4.272651000	2.058824000
H	-3.435965000	-0.582265000	3.365120000
H	2.766981000	0.368735000	-5.638888000
H	1.133411000	4.206405000	-4.456343000
O	-4.648949000	-4.376104000	5.494467000
C	-4.560330000	-5.787902000	5.640680000
H	-4.869200000	-6.317345000	4.711720000
H	-3.530323000	-6.109552000	5.913458000
H	-5.252266000	-6.059993000	6.459180000
O	3.417384000	6.363199000	4.666247000
C	4.280926000	6.008777000	5.738321000
H	3.774658000	5.341216000	6.471234000
H	5.205500000	5.507239000	5.374096000
H	4.557303000	6.954002000	6.241444000
O	3.438509000	4.047857000	-8.339222000
C	4.166865000	3.304368000	-9.308365000
H	5.088153000	2.855358000	-8.874357000
H	3.547961000	2.494915000	-9.756114000
H	4.452720000	4.021297000	-10.100228000
C	-5.238639000	1.059761000	-1.096127000
C	-4.651053000	-0.142445000	-0.569894000
C	-6.255139000	1.689629000	-0.298405000
C	-5.054779000	-0.660861000	0.661607000
C	-6.641973000	1.166492000	0.936069000
C	-6.045301000	-0.014595000	1.430894000
H	-3.879552000	-0.672672000	-1.145703000
H	-6.725531000	2.611373000	-0.678433000
H	-4.587921000	-1.584224000	1.034216000
H	-7.418442000	1.679019000	1.526561000
H	-6.348864000	-0.430946000	2.403906000
C	-4.851891000	1.631060000	-2.339062000
H	-5.389218000	2.543216000	-2.657538000
H	-2.614659000	1.469825000	-0.342435000
C	-3.718226000	1.174498000	-3.198660000
H	-3.294878000	0.224409000	-2.817990000
H	-4.078469000	0.964790000	-4.232640000
C	-2.575361000	2.213646000	-3.295285000
H	-2.908705000	3.102249000	-3.876928000
H	-1.734362000	1.759020000	-3.858061000
C	-2.074125000	2.707416000	-1.912150000
H	-1.133694000	3.283127000	-2.082282000
H	-2.823822000	3.421268000	-1.497432000
N	-1.816629000	1.647596000	-0.969922000

Structure of ^{2}TS3

Fe	-0.246838000	0.381517000	-0.209356000
N	0.444560000	0.409846000	1.603923000
N	-0.880413000	-1.388390000	0.095456000
C	-1.195317000	-2.309649000	3.749674000
C	-1.182465000	-2.076729000	1.258141000
C	-0.763815000	-1.592639000	2.522810000
C	0.044033000	-0.428355000	2.651517000
C	-0.933695000	-3.683906000	3.933785000
C	1.367360000	1.194632000	3.567043000
H	1.941327000	1.822744000	4.256847000
C	-1.392856000	-2.087071000	-0.997454000
C	0.633031000	0.064387000	3.876303000
H	0.520456000	-0.406533000	4.859158000
C	-1.910029000	-1.628683000	4.768093000
C	-1.362819000	-4.367261000	5.083901000
H	-1.126536000	-5.434649000	5.193154000
C	-1.946652000	-3.239353000	0.884502000
H	-2.360042000	-3.976736000	1.582242000
C	-2.064244000	-3.248224000	-0.514856000
H	-2.573691000	-4.001745000	-1.127642000
C	-2.076409000	-3.673159000	6.085599000
C	-2.345336000	-2.294728000	5.915642000
H	-2.909466000	-1.769785000	6.701128000
C	1.246764000	1.414506000	2.143817000
N	-0.384126000	-0.257378000	-2.024720000
N	0.902542000	1.794073000	-0.819688000
C	0.600552000	2.312704000	-4.613574000
C	1.016670000	2.260179000	-2.140789000
C	3.748855000	3.194536000	2.857674000
C	0.452026000	1.645414000	-3.295150000
C	-0.236570000	0.407437000	-3.225626000
C	2.585088000	3.520744000	2.131377000
C	0.180211000	3.652835000	-4.810244000
C	-1.438360000	-1.509996000	-3.620737000
H	-1.998365000	-2.322545000	-4.099600000
C	1.649735000	2.640582000	-0.001517000
C	2.164096000	4.874139000	2.125887000
C	-0.895901000	-0.380379000	-4.242491000
H	-0.956855000	-0.120396000	-5.305638000
C	1.157296000	1.629697000	-5.716849000
C	1.804062000	2.484095000	1.399952000
C	4.473513000	4.169561000	3.564127000
H	5.380111000	3.873838000	4.109701000
C	0.310497000	4.278342000	-6.051947000
H	-0.025450000	5.314506000	-6.207950000
C	1.878398000	3.417115000	-2.134283000
H	2.189690000	3.970153000	-3.027534000
C	4.034435000	5.511685000	3.554049000
C	2.269467000	3.648794000	-0.826360000
H	2.945008000	4.430586000	-0.461851000
C	2.871921000	5.854771000	2.824848000
H	2.539770000	6.903870000	2.830707000
C	0.872254000	3.580794000	-7.147005000
C	1.297774000	2.245686000	-6.971117000
H	1.749234000	1.681045000	-7.798423000
C	-1.125131000	-1.410483000	-2.225881000
H	4.106207000	2.153144000	2.859369000
H	1.249361000	5.147606000	1.578806000
H	-0.359858000	-4.228214000	3.168100000
H	-2.139471000	-0.559939000	4.638815000
H	1.512346000	0.596301000	-5.583520000
H	-0.273554000	4.201643000	-3.970693000
O	-2.540201000	-4.237437000	7.235789000
C	-2.303057000	-5.622349000	7.450063000
H	-2.772589000	-6.248214000	6.658139000
H	-1.214774000	-5.852810000	7.491779000
H	-2.761819000	-5.869941000	8.425400000
O	4.657144000	6.533435000	4.206380000
C	5.826667000	6.238549000	4.958268000
H	5.619133000	5.511118000	5.775015000
H	6.639581000	5.833330000	4.314681000
H	6.161042000	7.194513000	5.402580000
O	0.960778000	4.272470000	-8.317104000
C	1.516875000	3.615016000	-9.448214000
H	2.571185000	3.306586000	-9.268268000
H	0.924037000	2.717924000	-9.736153000
H	1.491264000	4.346218000	-10.277465000
C	-3.000669000	3.126026000	2.409059000
C	-1.700545000	2.864627000	2.956002000
C	-3.422242000	4.497612000	2.350121000
C	-0.889030000	3.904756000	3.407977000
C	-2.596978000	5.531815000	2.795729000
C	-1.321286000	5.246320000	3.329586000
H	-1.336561000	1.830597000	3.018294000
H	-4.418007000	4.725790000	1.935408000
H	0.104361000	3.671966000	3.819469000
H	-2.945244000	6.575263000	2.731136000
H	-0.667792000	6.059376000	3.681955000
C	-3.860300000	2.097296000	1.937278000
H	-4.854707000	2.413352000	1.569722000
H	-1.627897000	2.244708000	-0.780374000
C	-3.550947000	0.639413000	1.871055000
H	-2.496730000	0.453726000	2.151505000
H	-4.177373000	0.075928000	2.603839000
C	-3.800163000	0.030127000	0.472774000
H	-4.890270000	-0.007635000	0.254294000
H	-3.449117000	-1.021024000	0.475658000
C	-3.126435000	0.836139000	-0.651915000
H	-3.092735000	0.209860000	-1.579664000
H	-3.758779000	1.718717000	-0.899326000
N	-1.814231000	1.353051000	-0.297924000

Structure of ^{4}TS3

Fe	-0.303898000	0.359139000	-0.234709000
N	0.374769000	0.395983000	1.585192000
N	-0.914945000	-1.420610000	0.071099000
C	-1.219090000	-2.359942000	3.720396000
C	-1.219801000	-2.111973000	1.228957000
C	-0.803214000	-1.630737000	2.495504000
C	-0.001262000	-0.462663000	2.625010000
C	-0.947593000	-3.734259000	3.891030000
C	1.322883000	1.156284000	3.544419000
H	1.902181000	1.779268000	4.233924000
C	-1.412184000	-2.121208000	-1.025360000
C	0.605903000	0.013176000	3.847370000
H	0.516591000	-0.477379000	4.823078000
C	-1.926141000	-1.692131000	4.752753000
C	-1.359162000	-4.429198000	5.040561000
H	-1.115376000	-5.496013000	5.138469000
C	-1.974430000	-3.279445000	0.849047000
H	-2.386494000	-4.021241000	1.542961000
C	-2.081780000	-3.287129000	-0.550794000
H	-2.581452000	-4.042746000	-1.169029000
C	-2.064491000	-3.747327000	6.056413000
C	-2.343679000	-2.369363000	5.900433000
H	-2.901679000	-1.854055000	6.696626000
C	1.170081000	1.401562000	2.128906000
N	-0.409336000	-0.281634000	-2.044733000
N	0.824354000	1.793912000	-0.833197000
C	0.567860000	2.293767000	-4.632195000
C	0.948746000	2.258907000	-2.152566000
C	3.615299000	3.252225000	2.864895000
C	0.413231000	1.630286000	-3.312609000

C	-0.256338000	0.381534000	-3.245916000
C	2.446392000	3.548383000	2.133530000
C	0.128260000	3.625749000	-4.841851000
C	-1.435934000	-1.548885000	-3.647603000
H	-1.985289000	-2.366644000	-4.129873000
C	1.540501000	2.655908000	-0.005838000
C	1.992914000	4.890808000	2.123706000
C	-0.897764000	-0.414895000	-4.266462000
H	-0.952731000	-0.156387000	-5.330329000
C	1.149596000	1.614406000	-5.724841000
C	1.694393000	2.495860000	1.395853000
C	4.312386000	4.245588000	3.573449000
H	5.224116000	3.973278000	4.122663000
C	0.264655000	4.247036000	-6.085103000
H	-0.085821000	5.276861000	-6.250893000
C	1.783265000	3.435746000	-2.136067000
H	2.095758000	3.994018000	-3.025641000
C	3.841018000	5.576797000	3.558196000
C	2.147794000	3.678713000	-0.822668000
H	2.800740000	4.475436000	-0.449447000
C	2.675021000	5.889994000	2.822118000
H	2.317150000	6.930658000	2.824086000
C	0.851615000	3.553146000	-7.169127000
C	1.296058000	2.225992000	-6.980628000
H	1.767040000	1.664370000	-7.799070000
C	-1.137093000	-1.443586000	-2.251315000
H	4.000683000	2.220898000	2.867127000
H	1.073995000	5.140672000	1.573340000
H	-0.379149000	-4.268998000	3.114674000
H	-2.164745000	-0.624101000	4.634365000
H	1.519344000	0.587507000	-5.581483000
H	-0.345315000	4.171444000	-4.011256000
O	-2.510656000	-4.323417000	7.207906000
C	-2.262486000	-5.708461000	7.408044000
H	-2.737946000	-6.330938000	6.616974000
H	-1.172382000	-5.932558000	7.434386000
H	-2.707578000	-5.966734000	8.386950000
O	4.436385000	6.615026000	4.211470000
C	5.610309000	6.349824000	4.966970000
H	5.418636000	5.618796000	5.784520000
H	6.434861000	5.963218000	4.326636000
H	5.920394000	7.314254000	5.410743000
O	0.944167000	4.240272000	-8.341860000
C	1.525329000	3.585901000	-9.461981000
H	2.582116000	3.295594000	-9.266909000
H	0.950986000	2.677656000	-9.752547000
H	1.498585000	4.312054000	-10.295646000
C	-2.642083000	3.268701000	2.354373000
C	-1.581054000	2.890862000	3.239413000
C	-2.835402000	4.671068000	2.121218000
C	-0.784168000	3.854905000	3.857078000
C	-2.031090000	5.627681000	2.743059000
C	-1.000484000	5.228893000	3.621733000
H	-1.381899000	1.826211000	3.426214000
H	-3.639920000	4.986382000	1.436572000
H	0.032312000	3.534650000	4.521811000
H	-2.202667000	6.698157000	2.546549000
H	-0.360530000	5.980270000	4.108528000
C	-3.483897000	2.319508000	1.712849000
H	-4.283825000	2.731743000	1.070948000
H	-1.762123000	2.135705000	-0.960639000
C	-3.449355000	0.838117000	1.891501000
H	-2.434077000	0.525203000	2.207917000
H	-4.132350000	0.528220000	2.720275000
C	-3.836850000	0.066403000	0.612843000
H	-4.940093000	0.050521000	0.475948000
H	-3.522441000	-0.990585000	0.726599000
C	-3.202915000	0.698506000	-0.645269000
H	-3.125246000	-0.073795000	-1.451073000
H	-3.874733000	1.484597000	-1.055933000
N	-1.918260000	1.317622000	-0.351514000

Structure of ^{6}TS3

Fe	-0.272911000	0.405935000	-0.211821000
N	0.249751000	0.569461000	1.656082000
N	-0.840809000	-1.376052000	0.129119000
C	-1.254633000	-2.226260000	3.785163000
C	-1.203210000	-2.031139000	1.287154000
C	-0.860875000	-1.497522000	2.562029000
C	-0.103499000	-0.285358000	2.689992000
C	-1.002603000	-3.610015000	3.936848000
C	1.241154000	1.321893000	3.588953000
H	1.816499000	1.952556000	4.273558000
C	-1.240300000	-2.123532000	-0.961937000
C	0.522165000	0.180852000	3.904271000
H	0.443638000	-0.305427000	4.882648000
C	-1.916864000	-1.558807000	4.850622000
C	-1.388173000	-4.308253000	5.090868000
H	-1.153931000	-5.378457000	5.173389000
C	-1.898228000	-3.231841000	0.906241000
H	-2.346906000	-3.956346000	1.595340000
C	-1.904992000	-3.294241000	-0.501579000
H	-2.345751000	-4.080107000	-1.126293000
C	-2.053300000	-3.625918000	6.134497000
C	-2.313781000	-2.241132000	6.000607000
H	-2.842587000	-1.725572000	6.816139000
C	1.073476000	1.556250000	2.174944000
N	-0.278449000	-0.276256000	-2.019844000
N	0.740099000	1.931890000	-0.806286000
C	0.524871000	2.342681000	-4.609767000
C	0.886576000	2.360521000	-2.123942000
C	3.541324000	3.324420000	2.907988000
C	0.396480000	1.680508000	-3.295596000
C	-0.187319000	0.389772000	-3.222699000
C	2.406384000	3.666539000	2.139930000
C	0.098979000	3.684702000	-4.802928000
C	-1.274330000	-1.598529000	-3.594197000
H	-1.805508000	-2.440118000	-4.055116000
C	1.455506000	2.791364000	0.010797000
C	2.047635000	5.037044000	2.064905000
C	-0.810852000	-0.439241000	-4.231291000
H	-0.923029000	-0.181769000	-5.290810000
C	1.062028000	1.658769000	-5.725894000
C	1.611362000	2.638099000	1.425095000
C	4.288753000	4.296928000	3.591400000
H	5.173574000	3.988105000	4.164684000
C	0.198553000	4.304495000	-6.048411000
H	-0.147870000	5.337529000	-6.201897000
C	1.721944000	3.531292000	-2.128658000
H	2.052683000	4.069537000	-3.023547000
C	3.904777000	5.654394000	3.517753000
C	2.082997000	3.791259000	-0.810229000
H	2.754984000	4.577967000	-0.450277000
C	2.776675000	6.014489000	2.742812000
H	2.487082000	7.075226000	2.700347000
C	0.740379000	3.605112000	-7.153676000
C	1.176266000	2.271890000	-6.982342000
H	1.617649000	1.708601000	-7.815963000
C	-0.949446000	-1.460202000	-2.207420000
H	3.863573000	2.272973000	2.950575000
H	1.158980000	5.321372000	1.484075000
H	-0.458984000	-4.144573000	3.143485000
H	-2.145128000	-0.486968000	4.752645000
H	1.429500000	0.629715000	-5.595719000
H	-0.343207000	4.233697000	-3.957815000
O	-2.473805000	-4.205831000	7.291105000
C	-2.244757000	-5.597955000	7.471981000
H	-2.753768000	-6.203168000	6.688903000
H	-1.158472000	-5.839936000	7.465443000
H	-2.666760000	-5.856640000	8.460755000
O	4.552034000	6.674975000	4.144461000

C	5.688679000	6.366043000	4.941099000
H	5.429586000	5.680623000	5.778857000
H	6.501698000	5.904846000	4.336890000
H	6.047717000	7.325818000	5.356758000
O	0.801485000	4.292891000	-8.325549000
C	1.330857000	3.634401000	-9.470022000
H	2.390265000	3.330577000	-9.315645000
H	0.733075000	2.735255000	-9.739572000
H	1.281206000	4.364132000	-10.299277000
C	-2.505619000	2.982626000	2.247341000
C	-1.943515000	2.492232000	3.468496000
C	-2.188568000	4.334032000	1.880586000
C	-1.118654000	3.295304000	4.258432000
C	-1.356224000	5.124920000	2.671339000
C	-0.804523000	4.612729000	3.867959000
H	-2.162305000	1.463129000	3.785599000
H	-2.607492000	4.737410000	0.943869000
H	-0.702808000	2.887627000	5.192487000
H	-1.126326000	6.156785000	2.359749000
H	-0.138012000	5.234621000	4.484207000
C	-3.327448000	2.188984000	1.400317000
H	-3.879704000	2.731265000	0.613918000
H	-1.873188000	2.203761000	-0.947288000
C	-3.679738000	0.752046000	1.625587000
H	-2.848572000	0.267712000	2.176861000
H	-4.573831000	0.664282000	2.289515000
C	-3.942331000	-0.026056000	0.308729000
H	-5.029504000	-0.096995000	0.091020000
H	-3.583896000	-1.067799000	0.432172000
C	-3.235007000	0.656089000	-0.888621000
H	-3.048236000	-0.104896000	-1.688555000
H	-3.926991000	1.401270000	-1.345328000
N	-2.019394000	1.311487000	-0.457376000

Structure of ^{2}Int4

Fe	-0.900348000	0.190395000	0.274167000
N	-0.227364000	0.314802000	2.063653000
N	-1.724693000	-1.451171000	0.690615000
C	-1.160545000	-2.752539000	4.183591000
C	-1.814292000	-2.205891000	1.842752000
C	-1.184924000	-1.796278000	3.042940000
C	-0.483586000	-0.557373000	3.130873000
C	-0.712833000	-4.077054000	3.970415000
C	0.700695000	1.206163000	3.981923000
H	1.233930000	1.895770000	4.645074000
C	-2.323242000	-2.152551000	-0.353693000
C	0.129840000	-0.008751000	4.316883000
H	0.136025000	-0.486333000	5.301791000
C	-1.542280000	-2.403712000	5.502543000
C	-0.654056000	-5.020336000	5.009188000
H	-0.288108000	-6.033334000	4.791941000
C	-2.580307000	-3.395239000	1.531545000
H	-2.854100000	-4.176627000	2.249925000
C	-2.896176000	-3.356777000	0.170720000
H	-3.448402000	-4.112461000	-0.401472000
C	-1.046829000	-4.650274000	6.313498000
C	-1.490559000	-3.328869000	6.547954000
H	-1.799938000	-3.053450000	7.567665000
C	0.512782000	1.387966000	2.563097000
N	-1.250884000	-0.418089000	-1.475566000
N	0.326763000	1.503232000	-0.429176000
C	0.201257000	1.676942000	-4.261788000
C	0.516073000	1.842033000	-1.771505000
C	2.937767000	3.344944000	3.194230000
C	-0.076859000	1.179538000	-2.888497000
C	-0.935784000	0.063600000	-2.730683000
C	1.768386000	3.550649000	2.433479000
C	-0.089524000	3.013415000	-4.635559000
C	-2.303639000	-1.764421000	-3.004089000
H	-2.905535000	-2.574411000	-3.434066000
C	1.013855000	2.434180000	0.342615000
C	1.286082000	4.876966000	2.310752000
C	-1.614750000	-0.766259000	-3.702230000
H	-1.581001000	-0.626450000	-4.789070000
C	0.743773000	0.824074000	-5.246927000
C	1.057396000	2.424114000	1.761142000
C	3.602849000	4.409640000	3.826442000
H	4.514907000	4.205226000	4.403903000
C	0.152027000	3.473546000	-5.932316000
H	-0.083102000	4.508026000	-6.224948000
C	1.407204000	2.980630000	-1.832137000
H	1.785424000	3.432205000	-2.756213000
C	3.099519000	5.722903000	3.701766000
C	1.702176000	3.355510000	-0.533412000
H	2.360780000	4.166640000	-0.203110000
C	1.934285000	5.947337000	2.932763000
H	1.554680000	6.976473000	2.844432000
C	0.697523000	2.606145000	-6.907125000
C	0.994022000	1.271313000	-6.555139000
H	1.428505000	0.575279000	-7.285869000
C	-2.058970000	-1.543346000	-1.610549000
H	3.348616000	2.327400000	3.281762000
H	0.377761000	5.063150000	1.716482000
H	-0.377937000	-4.370292000	2.963936000
H	-1.911166000	-1.389438000	5.698188000
H	0.994516000	-0.213111000	-4.976425000
H	-0.530677000	3.694520000	-3.891520000
O	-1.030001000	-5.481730000	7.394274000
C	-0.587289000	-6.818830000	7.206474000
H	-1.229087000	-7.368545000	6.481562000
H	0.467616000	-6.858944000	6.852826000
H	-0.655011000	-7.311815000	8.194259000
O	3.661778000	6.824792000	4.275417000
C	4.832291000	6.650079000	5.061916000
H	4.647940000	5.981968000	5.933232000
H	5.673886000	6.234498000	4.463296000
H	5.113336000	7.654333000	5.430345000
O	0.897697000	3.143442000	-8.144085000
C	1.434458000	2.308721000	-9.161124000
H	2.449598000	1.935786000	-8.896826000
H	0.775041000	1.436136000	-9.369175000
H	1.505670000	2.932623000	-10.071570000
C	-3.548476000	0.896085000	2.445940000
C	-4.201394000	-0.308985000	2.765359000
C	-2.962561000	1.651911000	3.484123000
C	-4.311299000	-0.724788000	4.101972000
C	-3.059670000	1.233708000	4.817516000
C	-3.762075000	0.056184000	5.131323000
H	-4.635960000	-0.927866000	1.967418000
H	-2.432734000	2.590093000	3.249865000
H	-4.828928000	-1.666762000	4.339425000
H	-2.598354000	1.835417000	5.615342000
H	-3.876001000	-0.255895000	6.181180000
C	-3.585990000	1.477791000	1.054465000
H	-4.054034000	2.488152000	1.163386000
H	-1.706278000	2.421648000	0.955068000
C	-4.339801000	0.745148000	-0.059695000
H	-4.041435000	-0.321174000	-0.074919000
H	-5.434748000	0.794660000	0.095005000
C	-3.865387000	1.462971000	-1.351899000
H	-4.656363000	2.107514000	-1.783654000
H	-3.570049000	0.734875000	-2.130541000
C	-2.660374000	2.321078000	-0.906329000
H	-1.816454000	2.319877000	-1.615840000
H	-2.963779000	3.376903000	-0.733351000
N	-2.253601000	1.737089000	0.408039000

Structure of ^{4}Int4

Fe	-0.854537000	0.215637000	0.351605000
N	-0.168917000	0.359498000	2.138781000
N	-1.596986000	-1.460954000	0.798981000
C	-1.100580000	-2.660444000	4.340748000
C	-1.700212000	-2.187943000	1.961102000
C	-1.081216000	-1.751281000	3.164955000
C	-0.381942000	-0.516094000	3.217994000
C	-0.726131000	-4.015749000	4.187290000
C	0.815174000	1.261356000	4.016434000
H	1.365031000	1.960306000	4.656247000
C	-2.234299000	-2.145432000	-0.231264000
C	0.260568000	0.046485000	4.380399000
H	0.303335000	-0.424028000	5.367880000
C	-1.467852000	-2.237103000	5.643664000
C	-0.726033000	-4.917184000	5.264153000
H	-0.414803000	-5.956755000	5.091645000
C	-2.491126000	-3.357737000	1.670462000
H	-2.788395000	-4.118337000	2.401292000
C	-2.823209000	-3.326954000	0.307106000
H	-3.406020000	-4.072184000	-0.247840000
C	-1.104193000	-4.472461000	6.549253000
C	-1.471139000	-3.118890000	6.726735000
H	-1.766882000	-2.783949000	7.732511000
C	0.584529000	1.433236000	2.602730000
N	-1.203010000	-0.406060000	-1.396370000
N	0.307144000	1.557248000	-0.382880000
C	0.124972000	1.718025000	-4.220049000
C	0.489697000	1.881762000	-1.737561000
C	3.071896000	3.357638000	3.127143000
C	-0.111546000	1.221602000	-2.839951000
C	-0.950346000	0.088280000	-2.654892000
C	1.861893000	3.583147000	2.438858000
C	-0.169693000	3.055678000	-4.587440000
C	-2.332833000	-1.736410000	-2.871626000
H	-2.961982000	-2.537171000	-3.279062000
C	1.017035000	2.479371000	0.374871000
C	1.384921000	4.914843000	2.364717000
C	-1.674434000	-0.727492000	-3.595945000
H	-1.698269000	-0.574485000	-4.681222000
C	0.633269000	0.861200000	-5.220632000
C	1.108848000	2.470176000	1.788866000
C	3.782376000	4.407228000	3.735176000
H	4.724271000	4.186679000	4.255968000
C	0.039143000	3.513715000	-5.890731000
H	-0.198192000	4.549346000	-6.177470000
C	1.376790000	3.018963000	-1.810863000
H	1.739481000	3.471138000	-2.740846000
C	3.285525000	5.726378000	3.657065000
C	1.689963000	3.395525000	-0.516234000
H	2.349765000	4.210455000	-0.197324000
C	2.079233000	5.971274000	2.961239000
H	1.704922000	7.004876000	2.909075000
C	0.552013000	2.642667000	-6.879850000
C	0.848847000	1.305783000	-6.535608000
H	1.257925000	0.606606000	-7.277919000
C	-2.017538000	-1.529348000	-1.497405000
H	3.477304000	2.335134000	3.176525000
H	0.443748000	5.116653000	1.829385000
H	-0.399816000	-4.369234000	3.197569000
H	-1.777405000	-1.196355000	5.799627000
H	0.886186000	-0.177211000	-4.956757000
H	-0.588691000	3.738944000	-3.832680000
O	-1.140994000	-5.258381000	7.662984000
C	-0.778733000	-6.625986000	7.532094000
H	-1.448467000	-7.164741000	6.824461000
H	0.273808000	-6.743446000	7.188837000
H	-0.881580000	-7.073955000	8.538098000
O	3.891827000	6.815829000	4.210805000
C	5.105403000	6.619413000	4.922989000
H	4.967118000	5.941989000	5.795757000
H	5.905157000	6.203002000	4.269857000
H	5.419083000	7.615976000	5.286085000
O	0.721934000	3.177924000	-8.122280000
C	1.227215000	2.339401000	-9.152242000
H	2.246528000	1.960434000	-8.913771000
H	0.557481000	1.470653000	-9.342671000
H	1.278792000	2.962282000	-10.064702000
C	-3.911343000	0.526885000	2.187216000
C	-4.576698000	-0.712780000	2.165632000
C	-3.415442000	1.009154000	3.417860000
C	-4.749869000	-1.452502000	3.347159000
C	-3.592438000	0.275538000	4.597834000
C	-4.269167000	-0.957478000	4.567740000
H	-4.957195000	-1.116424000	1.216768000
H	-2.893683000	1.979514000	3.459041000
H	-5.264006000	-2.425130000	3.309432000
H	-3.205584000	0.674620000	5.548684000
H	-4.408968000	-1.536708000	5.492827000
C	-3.817442000	1.403627000	0.959567000
H	-4.358977000	2.352367000	1.207593000
H	-1.969346000	2.357076000	1.284655000
C	-4.390967000	0.879357000	-0.364767000
H	-4.082341000	-0.173545000	-0.513859000
H	-5.497603000	0.913124000	-0.368491000
C	-3.741361000	1.789543000	-1.441022000
H	-4.473059000	2.483859000	-1.899215000
H	-3.302955000	1.186192000	-2.258067000
C	-2.649341000	2.578162000	-0.681382000
H	-1.706998000	2.690582000	-1.244628000
H	-3.017339000	3.594341000	-0.412280000
N	-2.430313000	1.788446000	0.558789000

Structure of ^{6}Int4

Fe	-0.811339000	0.192569000	0.337364000
N	-0.189267000	0.381364000	2.157670000
N	-1.617319000	-1.442561000	0.799908000
C	-1.094820000	-2.658156000	4.331260000
C	-1.725911000	-2.171804000	1.964304000
C	-1.101780000	-1.736609000	3.176822000
C	-0.415226000	-0.469708000	3.237908000
C	-0.773900000	-4.026397000	4.135352000
C	0.719086000	1.346650000	4.040006000
H	1.221657000	2.069878000	4.691205000
C	-2.286976000	-2.111533000	-0.223497000
C	0.174526000	0.120650000	4.409510000
H	0.197041000	-0.327295000	5.406832000
C	-1.387993000	-2.258779000	5.664647000
C	-0.749530000	-4.950857000	5.189633000
H	-0.476135000	-5.994243000	4.979479000
C	-2.546959000	-3.311451000	1.682647000
H	-2.868016000	-4.060536000	2.414969000
C	-2.895653000	-3.267323000	0.305158000
H	-3.496303000	-3.999256000	-0.247783000
C	-1.053198000	-4.525985000	6.501849000
C	-1.371158000	-3.166052000	6.724643000
H	-1.616207000	-2.847110000	7.749117000
C	0.537158000	1.484647000	2.624799000
N	-1.191206000	-0.403698000	-1.410379000
N	0.308562000	1.588311000	-0.392019000
C	0.176339000	1.711929000	-4.225514000
C	0.538468000	1.882955000	-1.735647000
C	2.979086000	3.373482000	3.244285000
C	-0.072108000	1.218068000	-2.856016000

C	-0.934528000	0.090488000	-2.672310000
C	1.841421000	3.608686000	2.442429000
C	-0.002409000	3.079245000	-4.570940000
C	-2.396647000	-1.682015000	-2.865036000
H	-3.061514000	-2.455440000	-3.267488000
C	1.033849000	2.499802000	0.374998000
C	1.437049000	4.953380000	2.245053000
C	-1.698679000	-0.684684000	-3.602959000
H	-1.742697000	-0.520379000	-4.685618000
C	0.577636000	0.829453000	-5.256986000
C	1.092391000	2.496546000	1.797789000
C	3.688698000	4.428032000	3.841622000
H	4.577973000	4.201495000	4.445593000
C	0.208315000	3.535384000	-5.872947000
H	0.053405000	4.591682000	-6.140241000
C	1.466599000	2.978476000	-1.808558000
H	1.876851000	3.399086000	-2.732873000
C	3.260886000	5.759959000	3.646367000
C	1.761768000	3.367674000	-0.502691000
H	2.449254000	4.159425000	-0.184602000
C	2.126442000	6.012329000	2.839489000
H	1.804421000	7.055153000	2.699567000
C	0.613335000	2.637259000	-6.888785000
C	0.798592000	1.273774000	-6.569741000
H	1.126583000	0.553139000	-7.331676000
C	-2.055904000	-1.490737000	-1.511298000
H	3.333803000	2.340922000	3.382374000
H	0.553744000	5.160446000	1.621491000
H	-0.503695000	-4.367251000	3.124893000
H	-1.670919000	-1.218304000	5.864207000
H	0.746495000	-0.230725000	-5.014629000
H	-0.341586000	3.786533000	-3.798915000
O	-1.062309000	-5.335331000	7.598471000
C	-0.749031000	-6.710314000	7.422240000
H	-1.466438000	-7.211954000	6.734496000
H	0.282844000	-6.851150000	7.028962000
H	-0.820483000	-7.177377000	8.422213000
O	3.869984000	6.853180000	4.182639000
C	5.013785000	6.653884000	5.003701000
H	4.774840000	6.038190000	5.899671000
H	5.843090000	6.167086000	4.443264000
H	5.340262000	7.658240000	5.331669000
O	0.795853000	3.174762000	-8.128024000
C	1.195013000	2.311254000	-9.183922000
H	2.183116000	1.840566000	-8.980539000
H	0.445666000	1.508217000	-9.365339000
H	1.275457000	2.941688000	-10.089043000
C	-3.873435000	0.532259000	2.222537000
C	-4.568258000	-0.690431000	2.264885000
C	-3.317104000	1.038555000	3.417152000
C	-4.712982000	-1.389304000	3.474859000
C	-3.463715000	0.344939000	4.625125000
C	-4.171187000	-0.870404000	4.659390000
H	-4.990738000	-1.115174000	1.343522000
H	-2.766031000	1.993393000	3.407785000
H	-5.250189000	-2.350024000	3.487228000
H	-3.023252000	0.759429000	5.545447000
H	-4.287625000	-1.418204000	5.606590000
C	-3.807778000	1.365508000	0.963605000
H	-4.341274000	2.323599000	1.193360000
H	-1.962749000	2.339566000	1.210775000
C	-4.419861000	0.800986000	-0.326551000
H	-4.131086000	-0.261498000	-0.445653000
H	-5.525691000	0.850762000	-0.302840000
C	-3.787205000	1.662224000	-1.451592000
H	-4.525483000	2.335068000	-1.930695000
H	-3.359956000	1.022193000	-2.246579000
C	-2.684979000	2.483535000	-0.745523000
H	-1.756941000	2.585346000	-1.333362000
H	-3.054882000	3.505515000	-0.501528000
N	-2.431379000	1.739060000	0.516527000

Structure of ^{2}TS4

Fe	-0.305927000	0.794626000	-0.082576000
N	0.593970000	0.696792000	1.631214000
N	-0.951853000	-0.980088000	0.257494000
C	-1.204215000	-1.779771000	3.938431000
C	-1.240914000	-1.647324000	1.433154000
C	-0.747767000	-1.164232000	2.666812000
C	0.171818000	-0.083002000	2.716657000
C	-1.033786000	-3.151357000	4.217089000
C	1.790927000	1.323899000	3.502668000
H	2.504890000	1.864945000	4.132813000
C	-1.450945000	-1.710957000	-0.811557000
C	0.924288000	0.314564000	3.882588000
H	0.821135000	-0.140469000	4.874171000
C	-1.864084000	-0.980548000	4.906305000
C	-1.498509000	-3.719912000	5.415551000
H	-1.334752000	-4.790544000	5.600235000
C	-2.005613000	-2.822524000	1.094916000
H	-2.394755000	-3.554404000	1.811977000
C	-2.114510000	-2.869905000	-0.301163000
H	-2.605960000	-3.647004000	-0.899136000
C	-2.155043000	-2.909372000	6.367682000
C	-2.333252000	-1.531598000	6.100592000
H	-2.855374000	-0.915375000	6.848079000
C	1.585157000	1.565392000	2.093832000
N	-0.529590000	0.140575000	-1.868450000
N	1.007238000	2.055261000	-0.793812000
C	0.318445000	2.740252000	-4.488945000
C	1.046524000	2.520030000	-2.108040000
C	4.417736000	2.911942000	2.557402000
C	0.284168000	2.010494000	-3.197668000
C	-0.450878000	0.808735000	-3.079068000
C	3.251437000	3.424050000	1.953160000
C	-0.042100000	4.111222000	-4.537729000
C	-1.625281000	-1.136188000	-3.417665000
H	-2.190398000	-1.959822000	-3.870774000
C	1.992334000	2.723700000	-0.076839000
C	3.048204000	4.826485000	1.982113000
C	-1.160744000	0.019631000	-4.056766000
H	-1.292845000	0.286638000	-5.111640000
C	0.699820000	2.114447000	-5.694767000
C	2.250929000	2.532674000	1.302444000
C	5.356819000	3.755182000	3.175306000
H	6.257890000	3.316476000	3.625582000
C	-0.026431000	4.821111000	-5.739924000
H	-0.317999000	5.881420000	-5.781748000
C	2.100171000	3.503857000	-2.214511000
H	2.388338000	4.011652000	-3.141976000
C	5.135893000	5.149852000	3.198486000
C	2.693635000	3.618715000	-0.969217000
H	3.545570000	4.247065000	-0.687200000
C	3.970878000	5.677750000	2.594688000
H	3.809808000	6.765758000	2.627170000
C	0.356987000	4.179958000	-6.941374000
C	0.723547000	2.816351000	-6.911610000
H	1.040622000	2.295040000	-7.825292000
C	-1.234235000	-1.040263000	-2.046409000
H	4.603834000	1.827074000	2.530349000
H	2.140231000	5.248473000	1.523728000
H	-0.504633000	-3.786408000	3.490031000
H	-2.022512000	0.088391000	4.696729000
H	1.012990000	1.059352000	-5.677288000
H	-0.359282000	4.611471000	-3.609820000
O	-2.645563000	-3.357965000	7.557677000
C	-2.499445000	-4.736812000	7.869258000
H	-3.015396000	-5.384452000	7.125166000
H	-1.428631000	-5.036291000	7.924329000
H	-2.966305000	-4.883683000	8.861018000
O	5.978245000	6.055932000	3.770787000

C	7.160880000	5.574817000	4.394600000
H	6.931243000	4.882662000	5.235800000
H	7.825689000	5.051689000	3.670888000
H	7.687688000	6.462656000	4.791310000
O	0.343196000	4.952101000	-8.064205000
C	0.713675000	4.352853000	-9.298561000
H	1.762066000	3.979107000	-9.277493000
H	0.037491000	3.510265000	-9.567128000
H	0.628115000	5.143511000	-10.067097000
N	-1.635111000	1.838234000	0.187029000
C	-3.647662000	0.685298000	-0.703865000
H	-3.184219000	-0.306885000	-0.585411000
H	-3.387017000	1.061025000	-1.715496000
C	-3.076931000	1.649942000	0.364699000
C	-5.175096000	0.548099000	-0.532933000
H	-5.547900000	-0.156666000	-1.307695000
C	-5.477855000	-0.016109000	0.871931000
H	-5.003078000	-1.014599000	0.989596000
H	-6.573786000	-0.154212000	1.000908000
C	-3.406301000	1.087543000	1.777097000
H	-2.989168000	1.757935000	2.553239000
H	-2.915189000	0.102145000	1.887457000
C	-4.932880000	0.953641000	1.940970000
H	-5.141647000	0.552977000	2.956647000
C	-5.596707000	2.336954000	1.777997000
H	-6.694727000	2.256546000	1.934289000
H	-5.204370000	3.036202000	2.548524000
C	-3.776227000	3.025310000	0.189547000
H	-3.518675000	3.441455000	-0.806605000
H	-3.391144000	3.721758000	0.948712000
C	-5.855427000	1.920132000	-0.700742000
H	-5.659535000	2.322875000	-1.718461000
H	-6.958009000	1.819928000	-0.594550000
C	-5.300571000	2.883986000	0.365082000
H	-5.762827000	3.888769000	0.246043000
C	-0.354472000	5.322796000	-0.369994000
C	-1.191544000	5.129672000	0.757292000
C	-0.965813000	3.989915000	1.659244000
C	-2.269580000	6.032385000	0.976844000
C	-3.202221000	5.863814000	2.156055000
C	-2.790001000	4.825237000	3.175904000
C	-1.681198000	3.965900000	2.942895000
C	-0.568168000	6.381439000	-1.257926000
C	-2.473370000	7.092529000	0.072493000
H	-4.223188000	5.617340000	1.777093000
C	-3.512425000	4.705394000	4.380337000
C	-1.303477000	3.045370000	3.953079000
C	-2.023376000	2.944996000	5.146567000
C	-3.147251000	3.769383000	5.359152000
H	-4.376083000	5.368865000	4.553025000
H	-0.434733000	2.395421000	3.780432000
H	-1.708049000	2.224450000	5.917440000
H	-3.727046000	3.691835000	6.292060000
C	-1.637847000	7.272231000	-1.040903000
H	0.478348000	4.629517000	-0.539488000
H	-1.329251000	2.894307000	0.893296000
H	0.106277000	6.516585000	-2.118005000
H	-3.306440000	7.793006000	0.249574000
H	-1.816043000	8.108854000	-1.734476000
H	-3.331236000	6.844950000	2.666555000
H	0.080294000	3.635735000	1.707746000

10. References

[1] E. Steene, T. Wondimagegn, A. Ghosh, *J. Phys. Chem. B* **2001**, *105*, 11406-11413.

[2] a) S. Ganguly, H. Vazquez-Lima, A. Ghosh, *Chem. – Eur. J.* **2016**, *22*, 10336-10340; b) S. Cai, S. Licoccia, C. D'Ottavi, R. Paolesse, S. Nardis, V. Bulach, B. Zimmer, T. K. Shokhireva, F. A. Walker, *Inorg. Chim. Acta* **2002**, *339*, 171-178.

[3] L. Simkhovich, I. Goldberg, Z. Gross, *Inorg. Chem.* **2002**, *41*, 5433-5439.

[4] K. Nakano, K. Kobayashi, T. Ohkawara, H. Imoto, K. Nozaki, *J. Am. Chem. Soc.* **2013**, *135*, 8456-8459.

[5] P. Dauban, L. Sanière, A. Tarrade, R. H. Dodd, *J. Am. Chem. Soc.* **2001**, *123*, 7707-7708.

[6] a) H. Gao, J. T. Groves, *J. Am. Chem. Soc.* **2017**, *139*, 3938-3941; b) C. R. Goldsmith, R. T. Jonas, T. D. P. Stack, *J. Am. Chem. Soc.* **2002**, *124*, 83-96.

[7] B. M. Trost, S. M. Silverman, J. P. Stambuli, *J. Am. Chem. Soc.* **2011**, *133*, 19483-19497.

[8] E. T. Hennessy, T. A. Betley, *Science* **2013**, *340*, 591-595.

[9] P. F. Kuijpers, M. J. Tiekink, W. B. Breukelaar, D. L. J. Broere, N. P. van Leest, J. I. van der Vlugt, J. N. H. Reek, B. de Bruin, *Chem. – Eur. J.* **2017**, *23*, 7945-7952.

[10] J. Qin, Z. Zhou, T. Cui, M. Hemming, E. Meggers, *Chem. Sci.* **2019**, *10*, 3202-3207.

[11] T. You, S.-H. Zeng, J. Fan, L. Wu, F. Kang, Y. Liu, C.-M. Che, *Chem. Commun.* **2021**, *57*, 10711-10714.

[12] Y.-D. Du, C.-Y. Zhou, W.-P. To, H.-X. Wang, C.-M. Che, *Chem. Sci.* **2020**, *11*, 4680-4686.

[13] Z. Zhou, S. Chen, J. Qin, X. Nie, X. Zheng, K. Harms, R. Riedel, K. N. Houk, E. Meggers, *Angew. Chem. Int. Ed.* **2019**, *58*, 1088-1093.

[14] D. A. Iovan, M. J. T. Wilding, Y. Baek, E. T. Hennessy, T. A. Betley, *Angew. Chem. Int. Ed.* **2017**, *56*, 15599-15602.

[15] Y. Xia, L. Wang, A. Studer, *Angew. Chem. Int. Ed.* **2018**, *57*, 12940-12944.

[16] J. Fan, Y. Wang, X. Hu, Y. Liu, C.-M. Che, *Org. Chem. Front.* **2023**, *10*, 1368-1374.

[17] G. McCort, C. Hoornaert, M. Aletru, C. Denys, O. Duclos, C. Cadilhac, E. Guilpain, G. Dellac, P. Janiak, A.-M. Galzin, M. Delahaye, F. Guilbert, S. O'Connor, *Bioorg. Med. Chem.* **2001**, *9*, 2129-2137.

[18] V. Molteni, X. He, Y. He, A. Kreusch, J. Nabakka, K. Yang, PCT Int. Appl., 2005011611, 10 Feb **2005**.

[19] D. N. A. M. Eriyagama, Y. Yin, S. Fang, *Tetrahedron* **2022**, *119*, 132861.

[20] R. Srinivasan, L. P. Tan, H. Wu, P.-Y. Yang, K. A. Kalesh, S. Q. Yao, *Org. Biomol. Chem.* **2009**, *7*, 1821-1828.

[21] E. C. Taylor, B. Liu, *J. Org. Chem.* **2003**, *68*, 9938-9947.

[22] A. J. Zaitouna, J. Joyce, R. L. Cerny, P. H. Dussault, R. Y. Lai, *Anal. Chem.* **2015**, *87*, 6966-6973.

[23] K.-P. Shing, Y. Liu, B. Cao, X.-Y. Chang, T. You, C.-M. Che, *Angew. Chem. Int. Ed.* **2018**, *57*, 11947-11951.

[24] Y. Wang, X. Wen, X. Cui, X. P. Zhang, *J. Am. Chem. Soc.* **2018**, *140*, 4792-4796.

[25] S. L. Pira, T. W. Wallace, J. P. Graham, *Org. Lett.* **2009**, *11*, 1663-1666.

[26] K. R. Campos, A. Klapars, J. H. Waldman, P. G. Dormer, C.-y. Chen, *J. Am. Chem. Soc.* **2006**, *128*, 3538-3539.

[27] S. Liang, X. Zhao, T. Yang, W. Yu, *Org. Lett.* **2020**, *22*, 1961-1965.

[28] a) M. Jäkel, J. Qu, T. Schnitzer, G. Helmchen, *Chem. – Eur. J.* **2013**, *19*, 16746-16755; b) W. Chen, L.

Ma, A. Paul, D. Seidel, *Nat. Chem.* **2018**, *10*, 165-169.
[29] S. Munnuri, A. M. Adebesin, M. P. Paudyal, M. Yousufuddin, A. Dalipe, J. R. Falck, *J. Am. Chem. Soc.* **2017**, *139*, 18288-18294.
[30] X. Li, I. Coldham, *J. Am. Chem. Soc.* **2014**, *136*, 5551-5554.
[31] S. Xiao, X.-X. Shi, J. Xing, J.-J. Yan, S.-L. Liu, W.-D. Lu, *Tetrahedron: Asymmetry* **2009**, *20*, 2090-2096.
[32] B. C. Schardt, C. L. Hill, *Inorg. Chem.* **1983**, *22*, 1563-1565.
[33] a) C. Ernst, J. Sindlinger, D. Schwarzer, P. Koch, F. M. Boeckler, *ACS Omega* **2018**, *3*, 12361-12368; b) T. Sasaki, S. Eguchi, T. Okano, *Synthesis* **1980**, *1980*, 472-475.
[34] S. Ganguly, L. J. Giles, K. E. Thomas, R. Sarangi, A. Ghosh, *Chem. – Eur. J.* **2017**, *23*, 15098-15106.
[35] S. Stoll, A. Schweiger, *J. Magn. Reson.* **2006**, *178*, 42-55.
[36] L. Du, Y. Qiu, X. Lan, R. Zhu, D. L. Phillips, M.-D. Li, A. S. Dutton, A. H. Winter, *J. Am. Chem. Soc.* **2017**, *139*, 15054-15059.
[37] P.-C. Li, T.-S. Wang, G.-H. Lee, Y.-H. Liu, Y. Wang, C.-T. Chen, I. Chao, *J. Org. Chem.* **2002**, *67*, 8002-8009.
[38] M. J. Frisch, G. W. Trucks, H. B. Schlegel, G. E. Scuseria, M. A. Robb, J. R. Cheeseman, G. Scalmani, V. Barone, B. Mennucci, G. A. Petersson, H. Nakatsuji, M. Caricato, X. Li, H. P. Hratchian, A. F. Izmaylov, J. Bloino, G. Zheng, J. L. Sonnenberg, M. Hada, M. Ehara, K. Toyota, R. Fukuda, J. Hasegawa, M. Ishida, T. Nakajima, Y. Honda, O. Kitao, H. Nakai, T. Vreven, J. A. Montgomery, Jr., J. E. Peralta, F. Ogliaro, M. Bearpark, J. J. Heyd, E. Brothers, K. N. Kudin, V. N. Staroverov, R. Kobayashi, J. Normand, K. Raghavachari, A. Rendell, J. C. Burant, S. S. Iyengar, J. Tomasi, M. Cossi, N. Rega, J. M. Millam, M. Klene, J. E. Knox, J. B. Cross, V. Bakken, C. Adamo, J. Jaramillo, R. Gomperts, R. E. Stratmann, O. Yazyev, A. J. Austin, R. Cammi, C. Pomelli, J. W. Ochterski, R. L. Martin, K. Morokuma, V. G. Zakrzewski, G. A. Voth, P. Salvador, J. J. Dannenberg, S. Dapprich, A. D. Daniels, Ö. Farkas, J. B. Foresman, J. V. Ortiz, J. Cioslowski and D. J. Fox, Gaussian 09 Revision D.01,Gaussian Inc., Wallingford CT, (**2013**).
[39] a) A. D. Becke, *Phys. Rev. A* **1988**, *38*, 3098-3100; b) J. P. Perdew, *Phys. Rev. B* **1986**, *33*, 8822-8824.
[40] a) S. Grimme, J. Antony, S. Ehrlich, H. Krieg, *J. Chem. Phys.* **2010**, *132*, 154104; b) S. Grimme, S. Ehrlich, L. Goerigk, *J. Comput. Chem.* **2011**, *32*, 1456-1465.
[41] M. Cossi, G. Scalmani, N. Rega, V. Barone, *J. Chem. Phys.* **2002**, *117*, 43-54.
[42] Y. Zhao, D. G. Truhlar, *J. Chem. Phys.* **2006**, *125*, 194101.
[43] T. Lu, F. Chen, *J. Comput. Chem.* **2012**, *33*, 580-592.

11. NMR Spectra

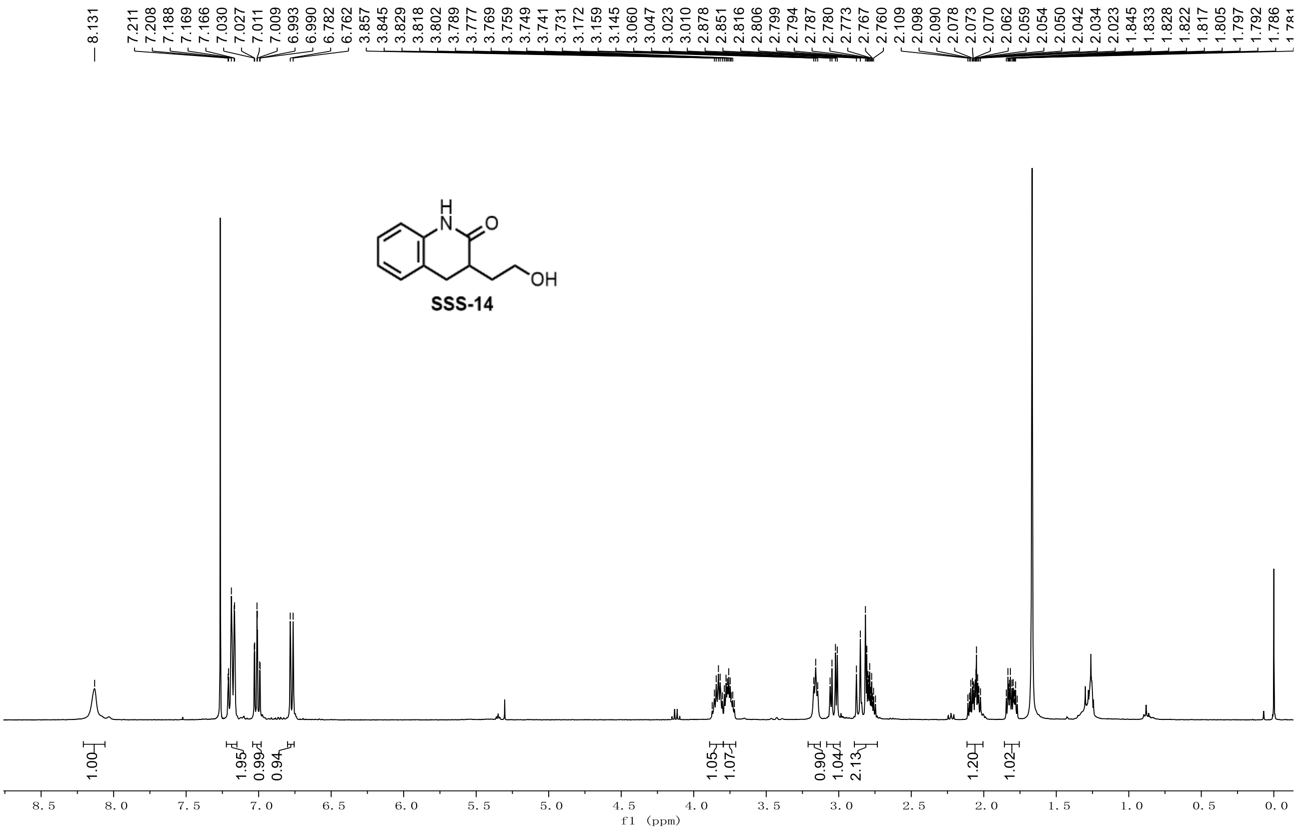

^{1}H NMR spectrum of **SSS-14** (400 MHz, $CDCl_3$)

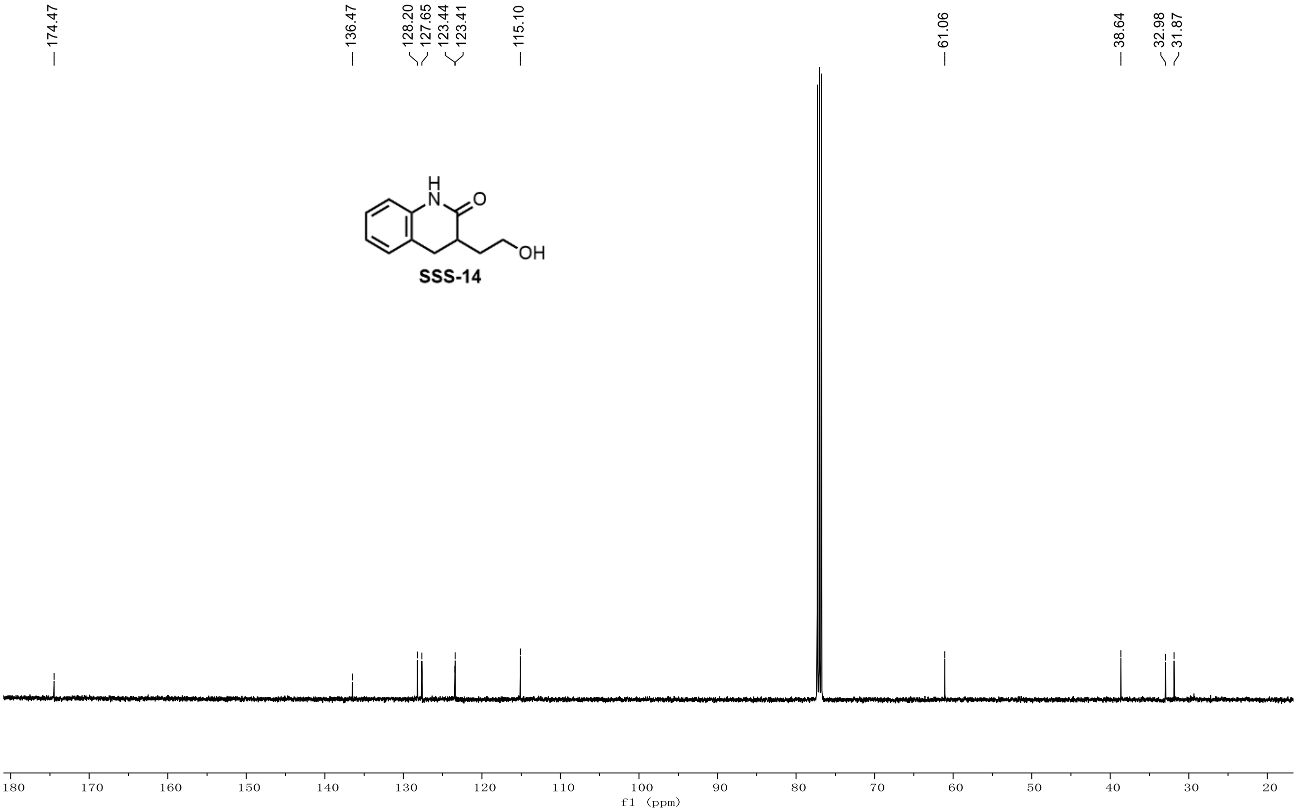

^{13}C NMR spectrum of **SSS-14** (125 MHz, $CDCl_3$)

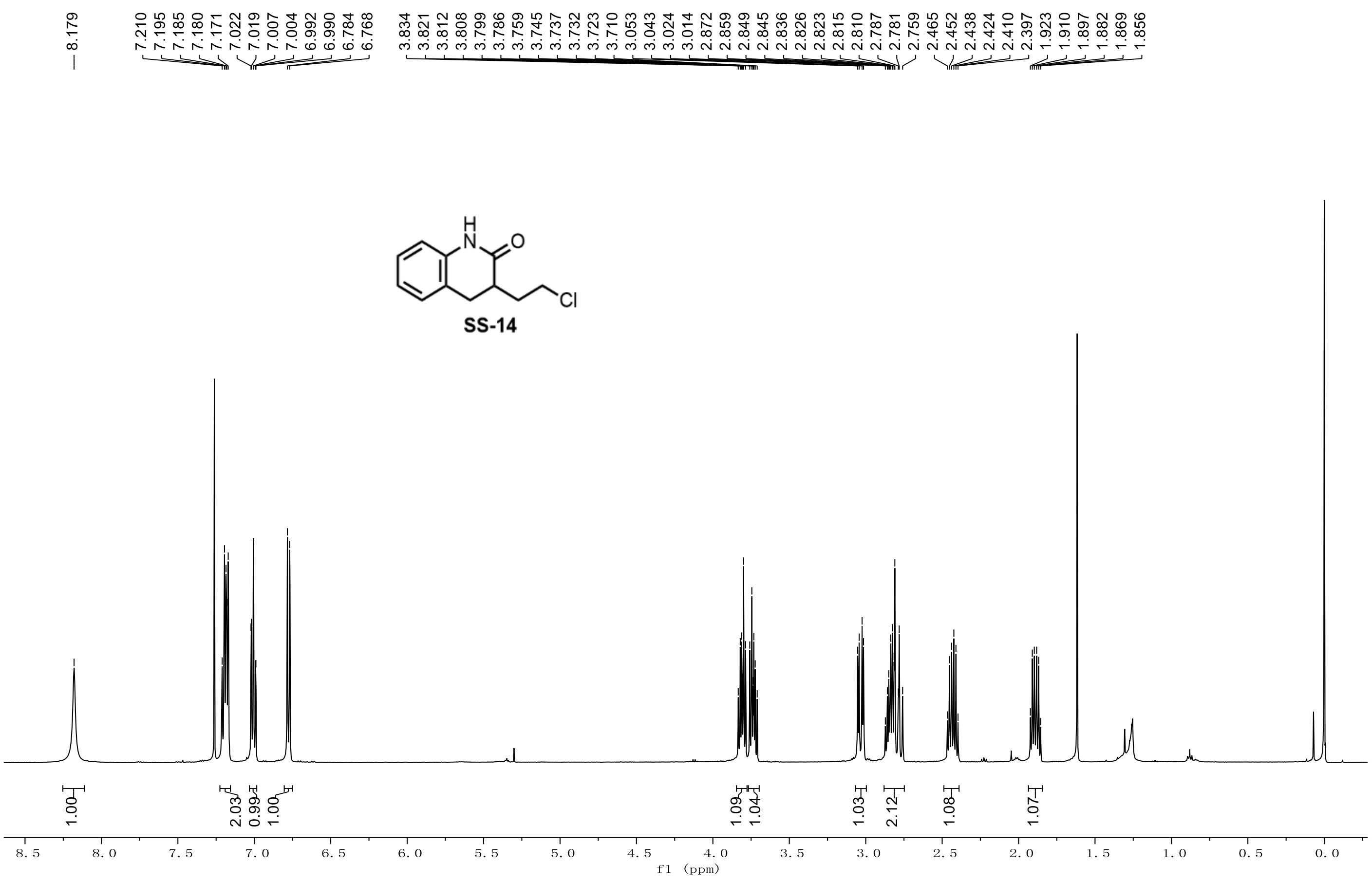

^{1}H NMR spectrum of **SS-14** (500 MHz, $CDCl_3$)

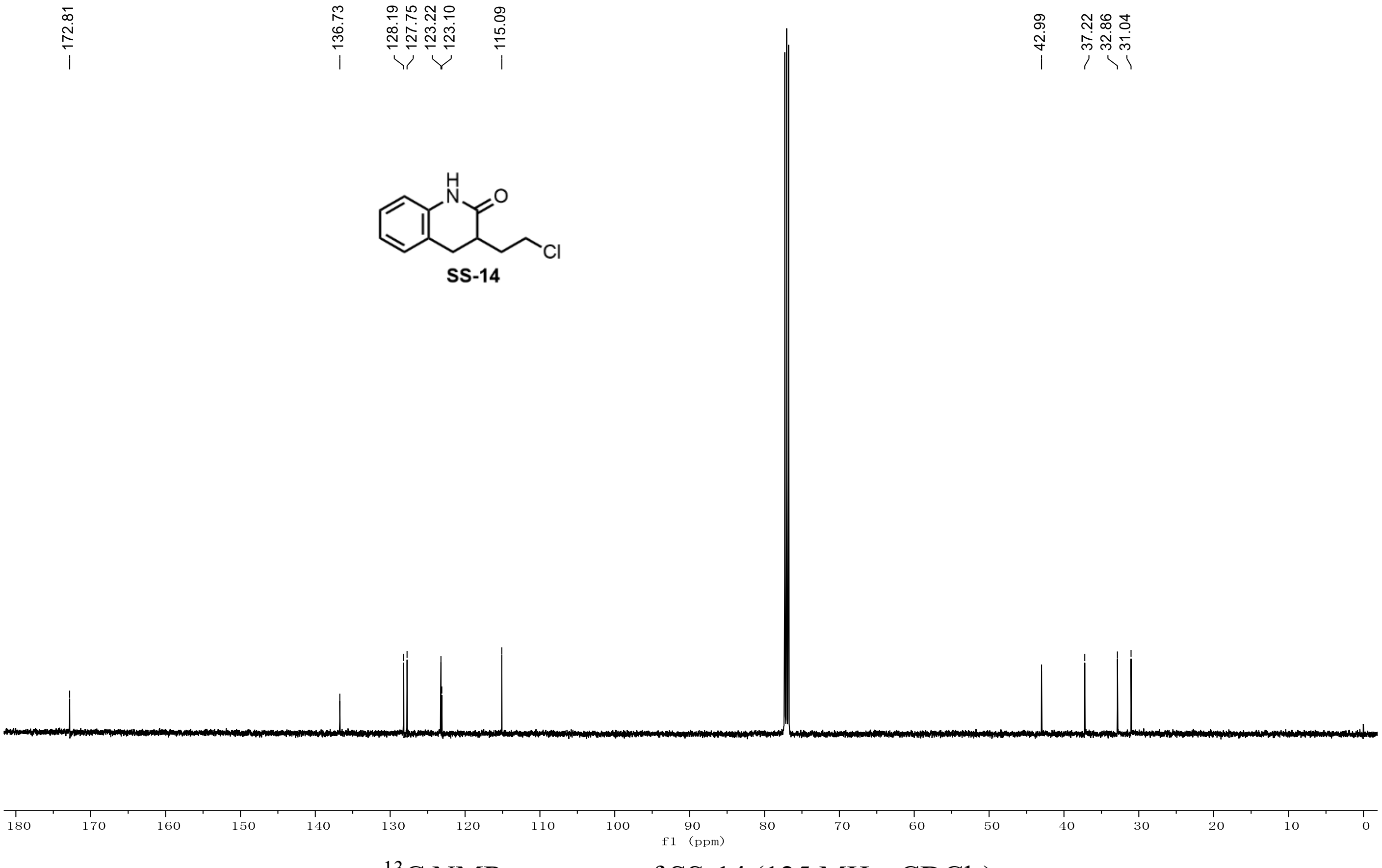

^{13}C NMR spectrum of **SS-14** (125 MHz, $CDCl_3$)

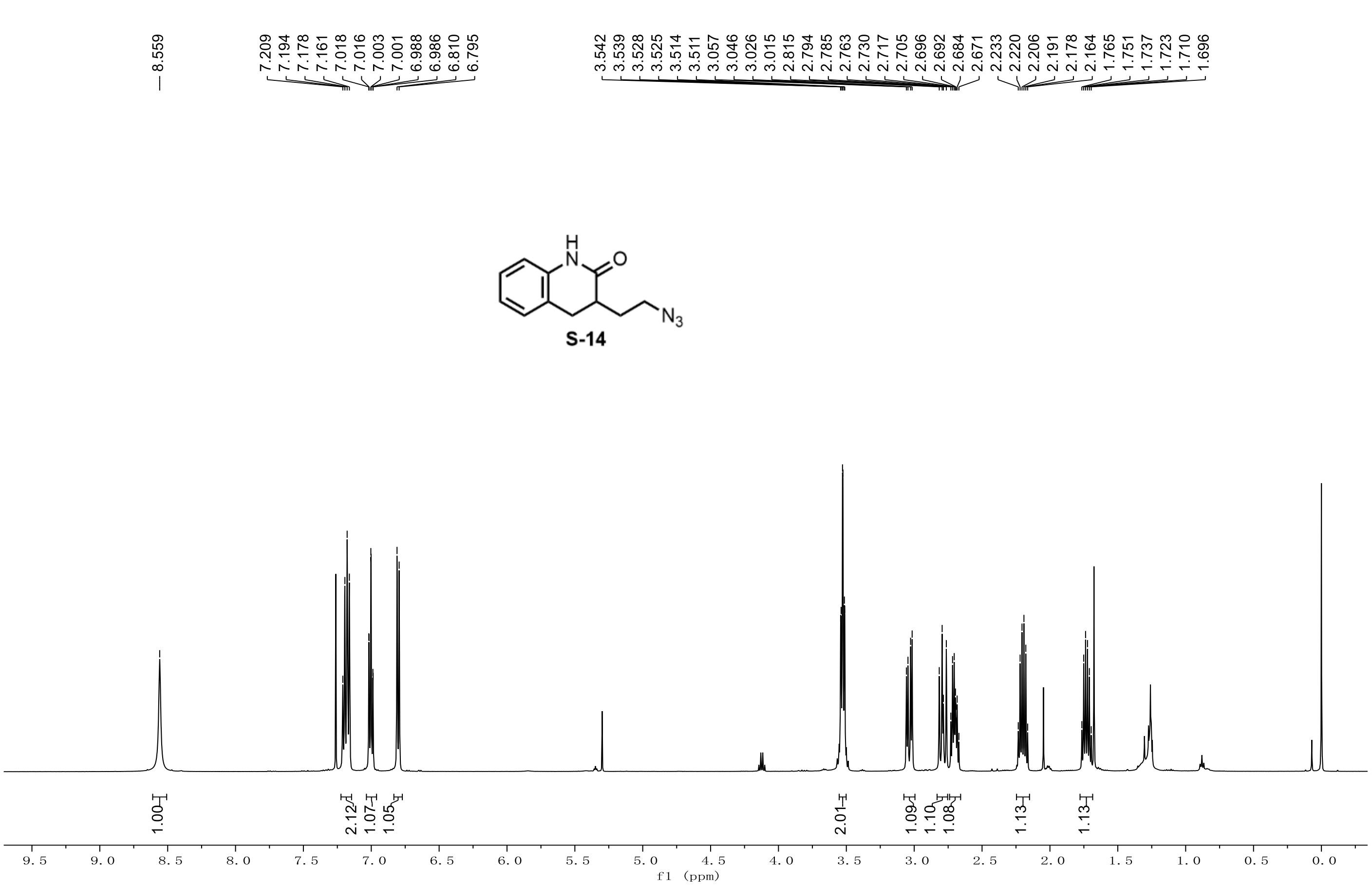

^{1}H NMR spectrum of **S-14** (500 MHz, $CDCl_3$)

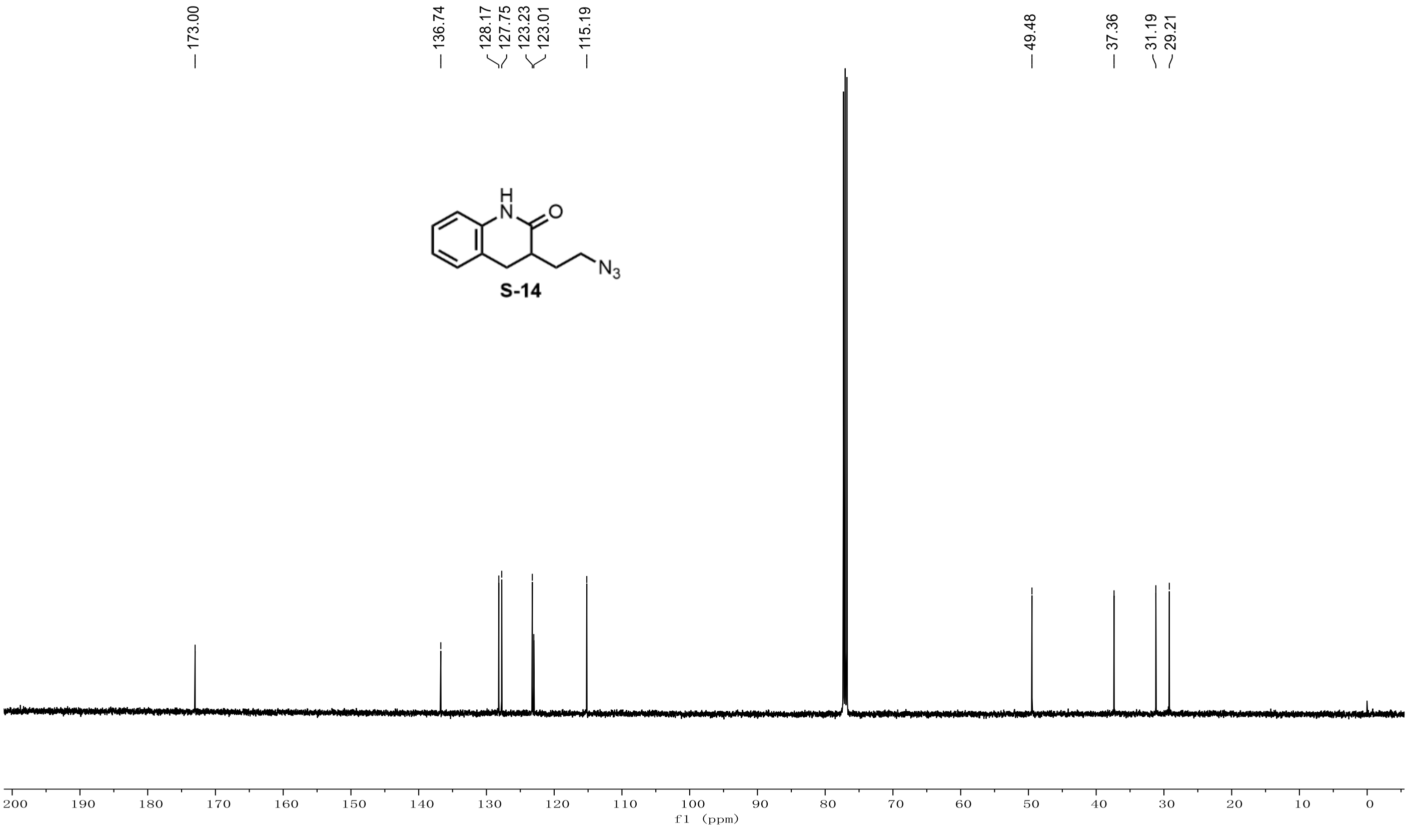

^{13}C NMR spectrum of **S-14** (125 MHz, $CDCl_3$)

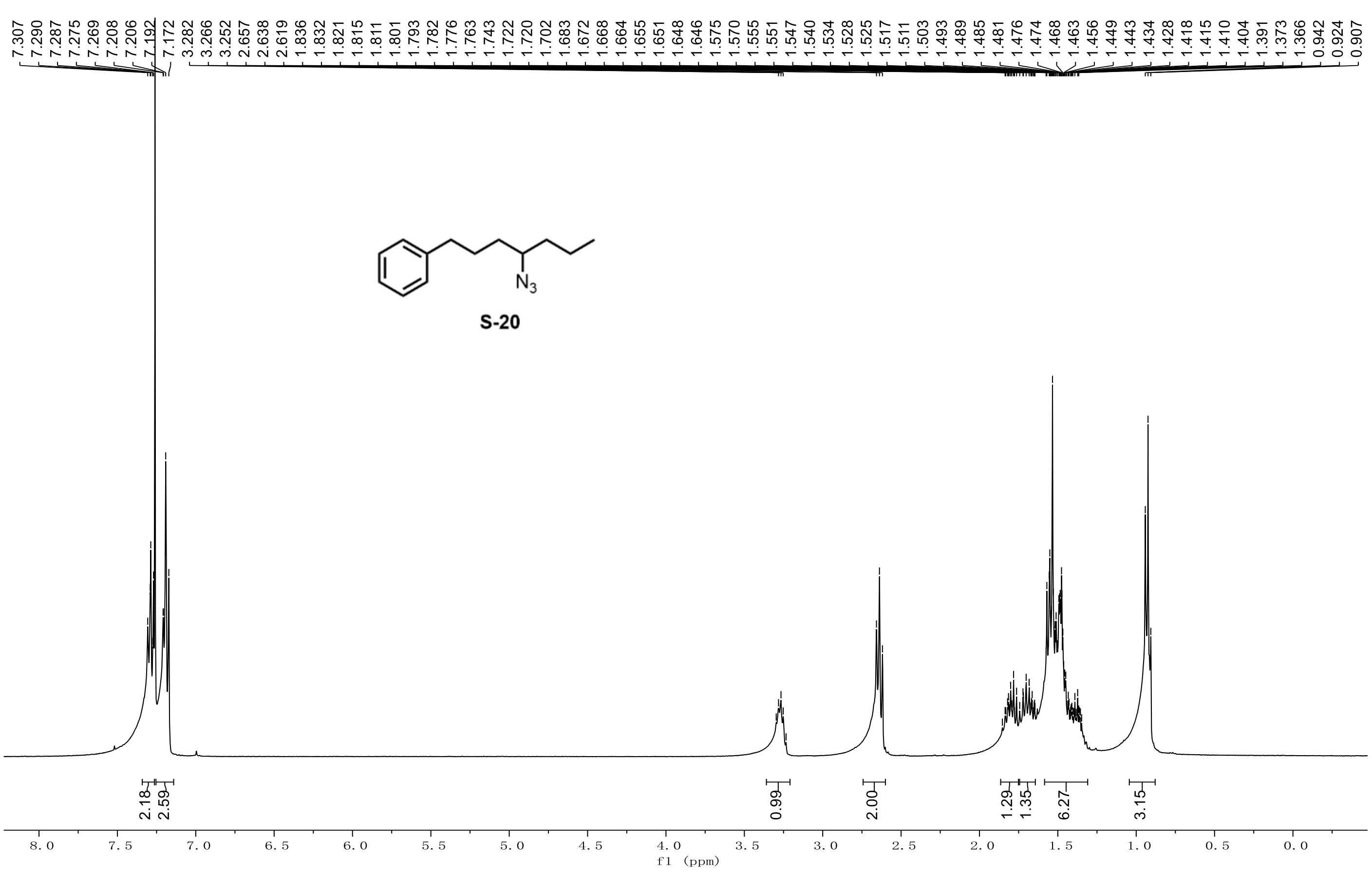

^{1}H NMR spectrum of **S-20** (400 MHz, $CDCl_3$)

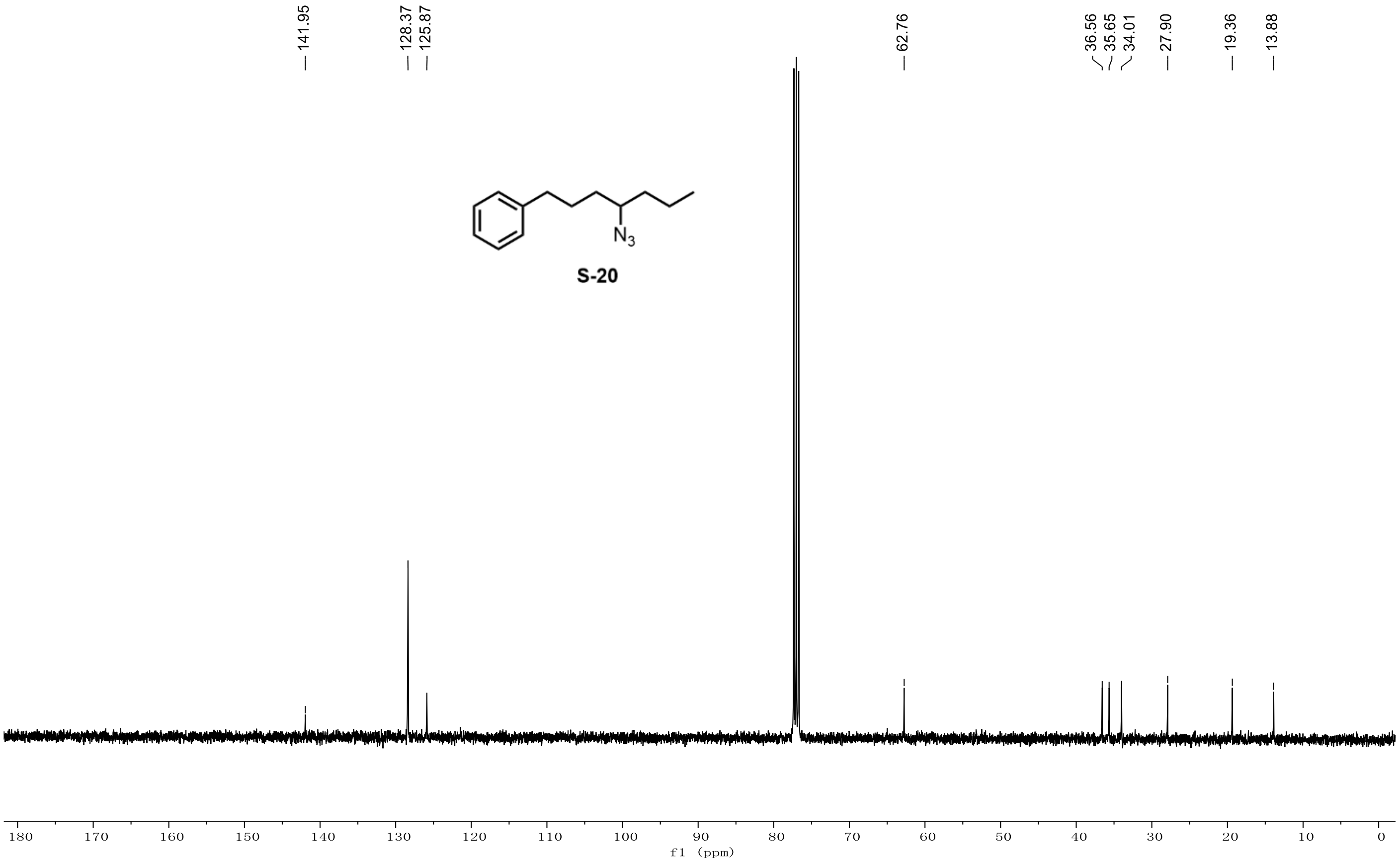

^{13}C NMR spectrum of **S-20** (100 MHz, $CDCl_3$)

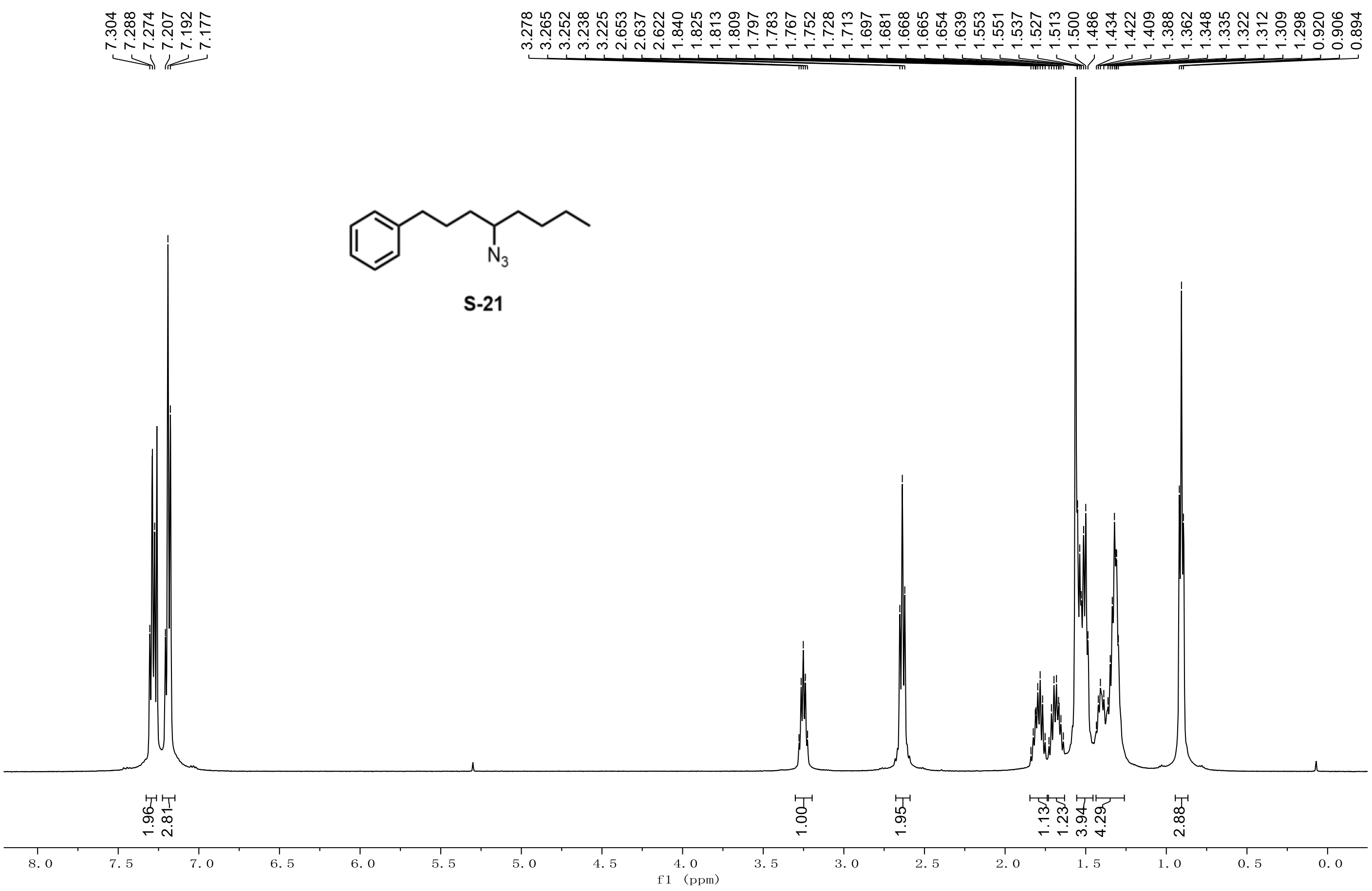

^{1}H NMR spectrum of **S-21** (500 MHz, $CDCl_3$)

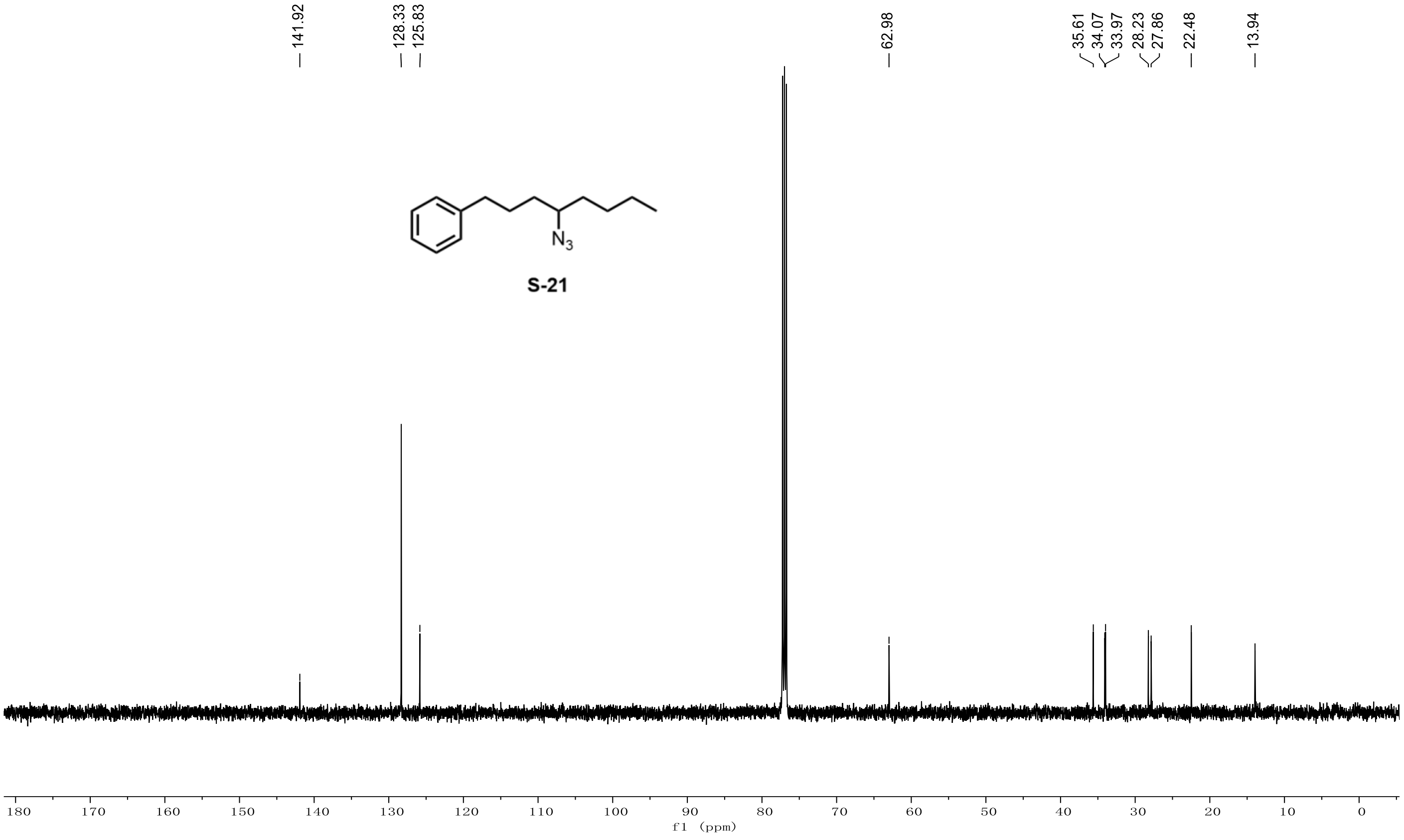

^{13}C NMR spectrum of **S-21** (125 MHz, $CDCl_3$)

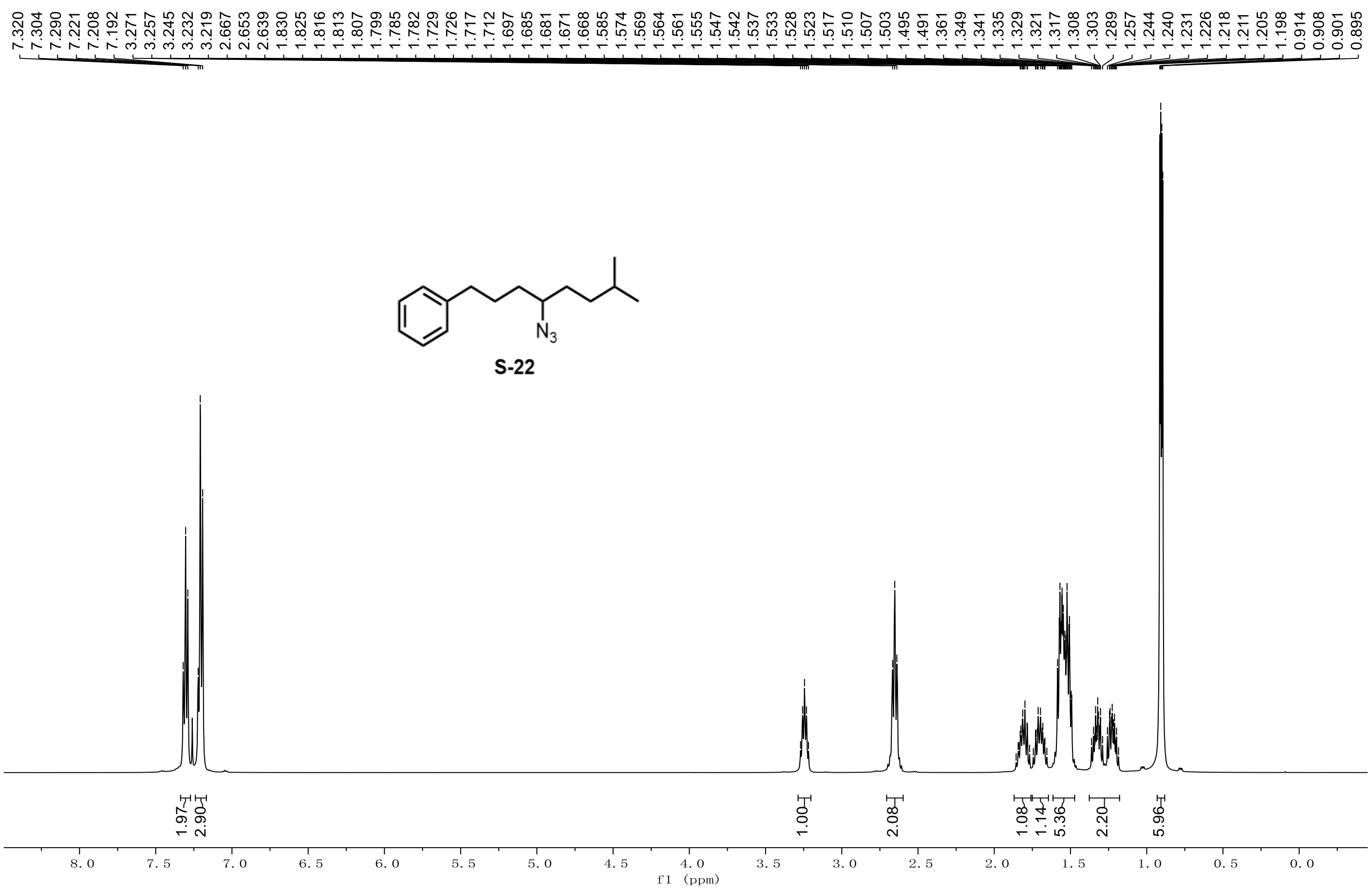

^{1}H NMR spectrum of **S-22** (500 MHz, $CDCl_3$)

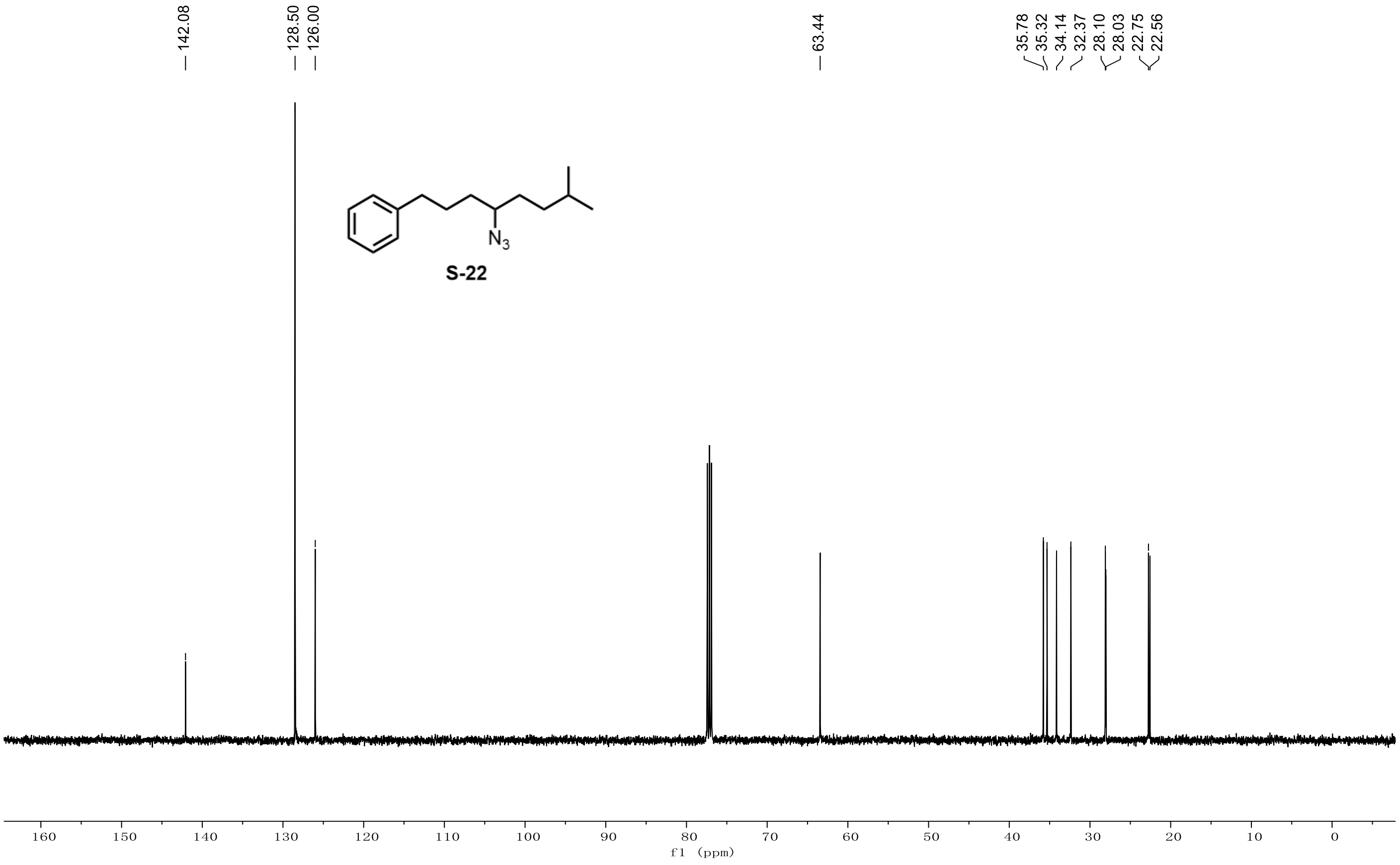

^{13}C NMR spectrum of **S-22** (125 MHz, $CDCl_3$)

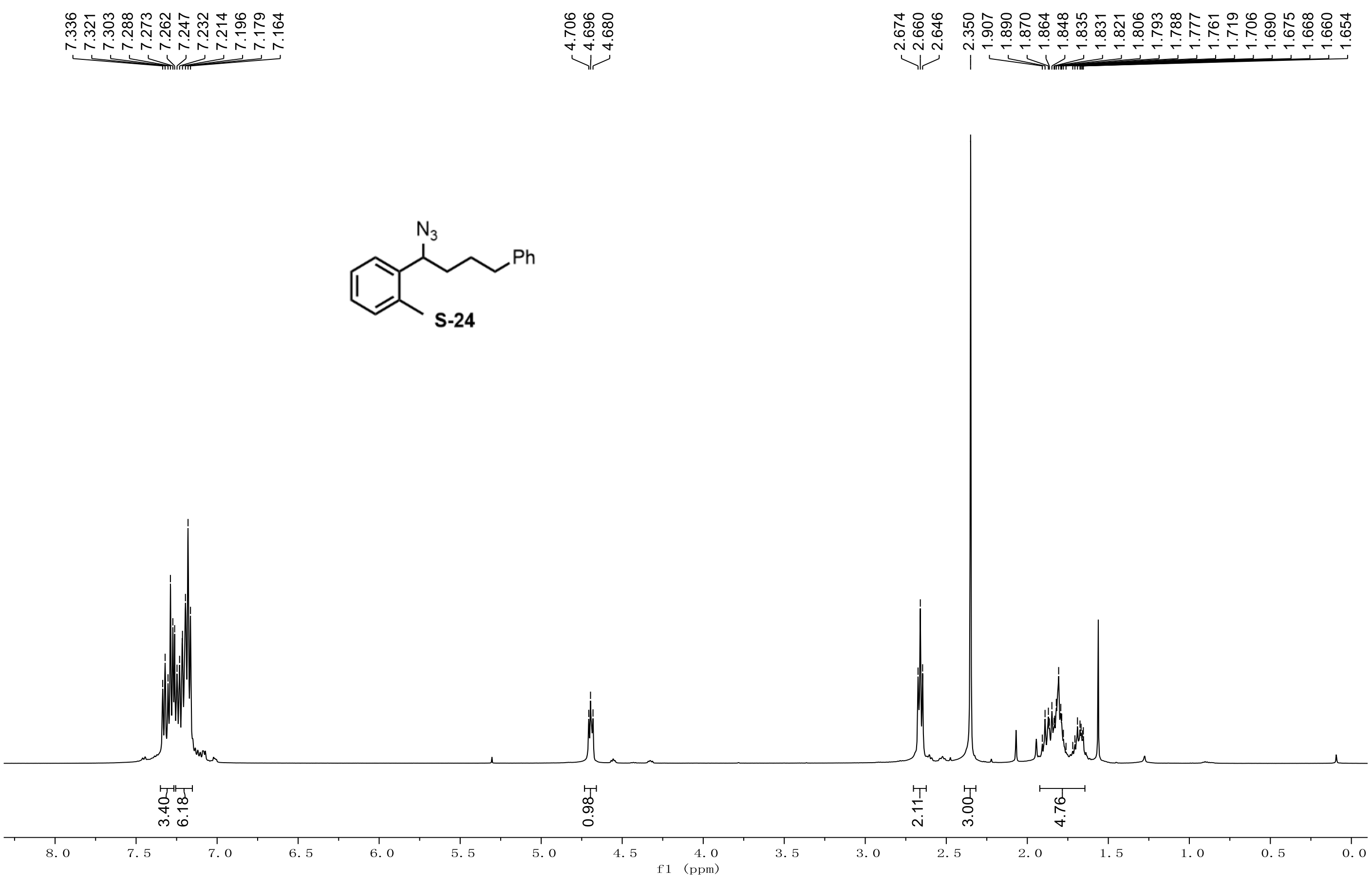

^{1}H NMR spectrum of **S-24** (500 MHz, $CDCl_3$)

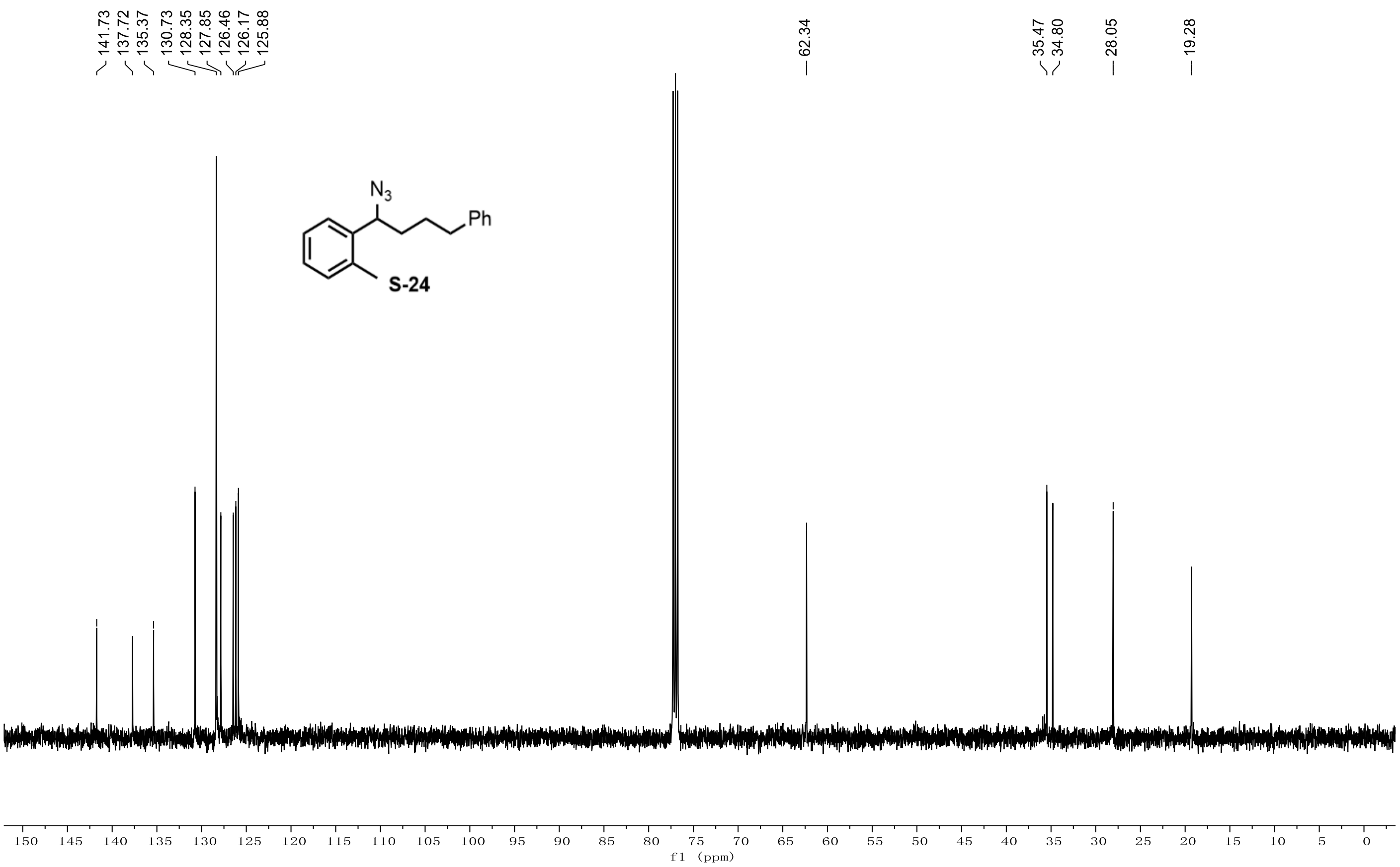

^{13}C NMR spectrum of **S-24** (125 MHz, $CDCl_3$)

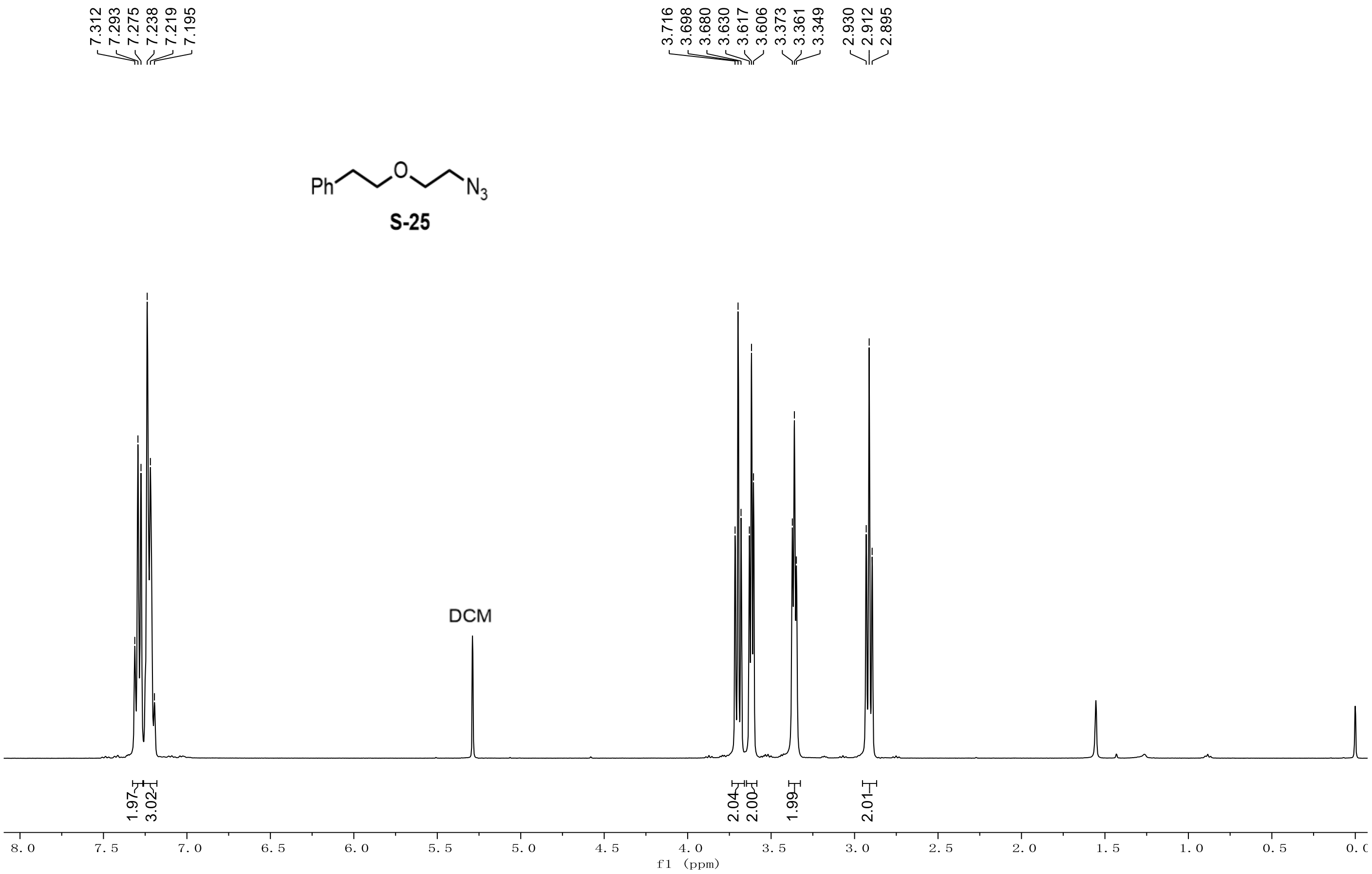

^{1}H NMR spectrum of **S-25** (400 MHz, $CDCl_3$)

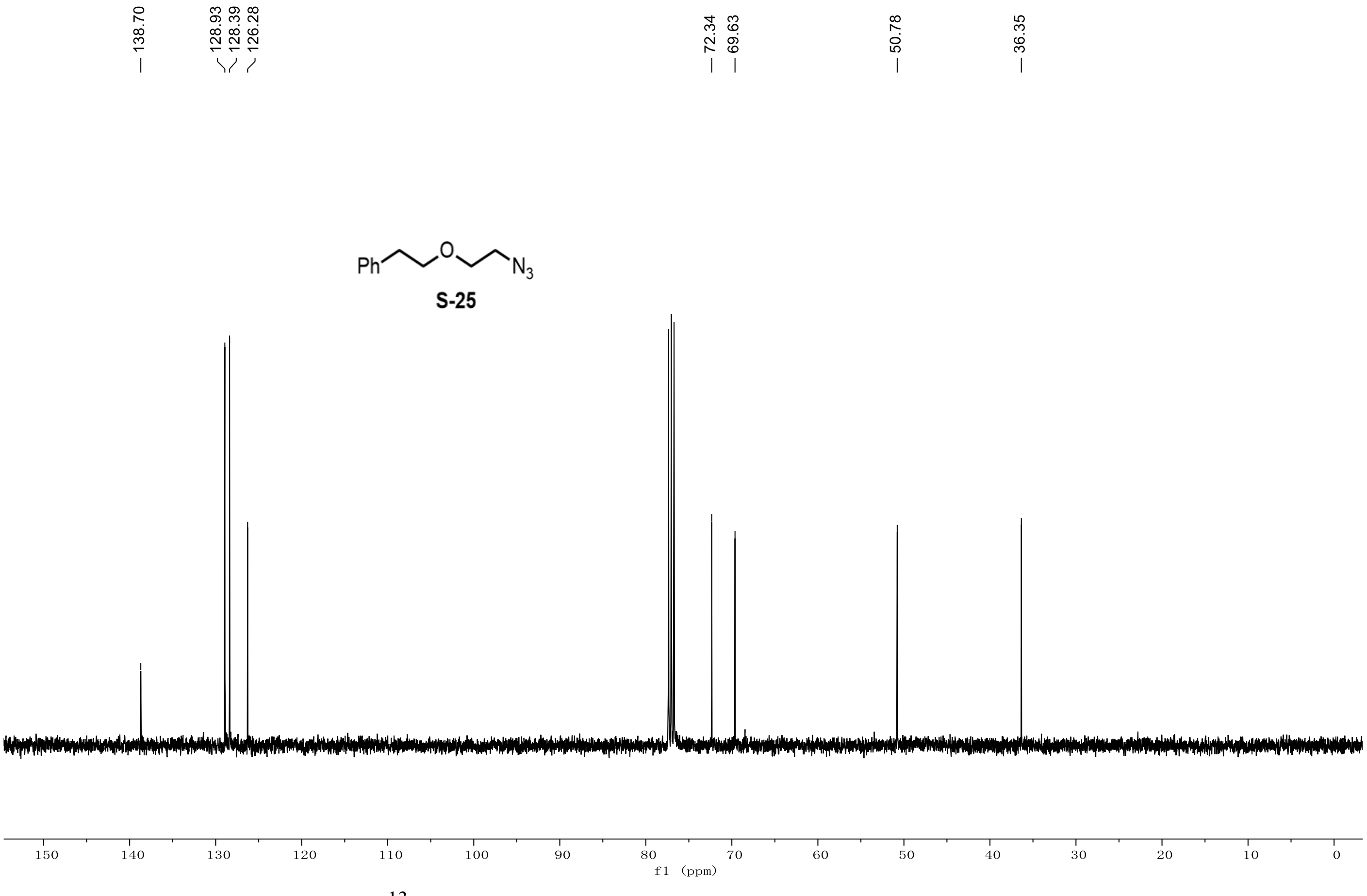

^{13}C NMR spectrum of **S-25** (100 MHz, $CDCl_3$)

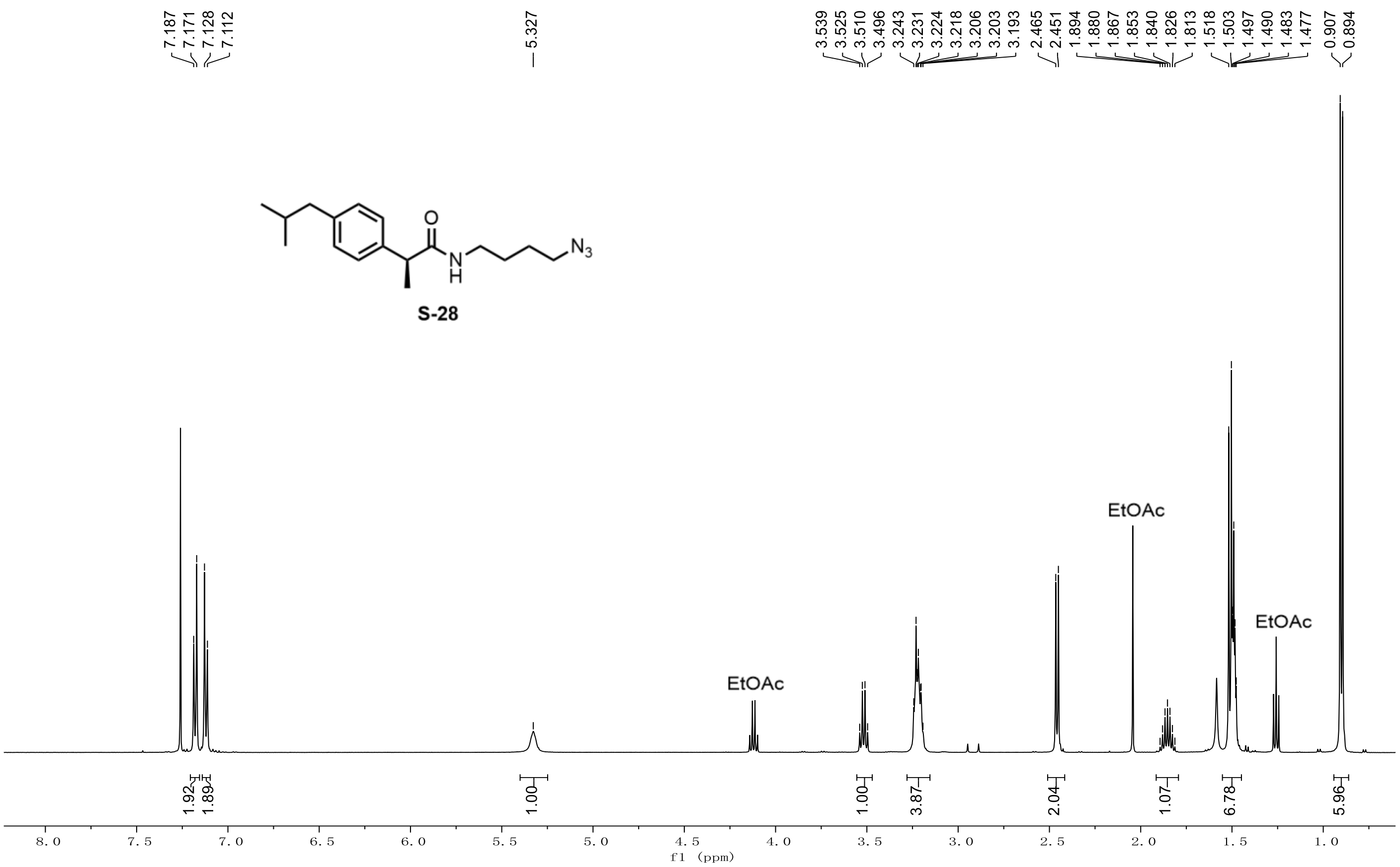

^{1}H NMR spectrum of **S-28** (500 MHz, $CDCl_3$)

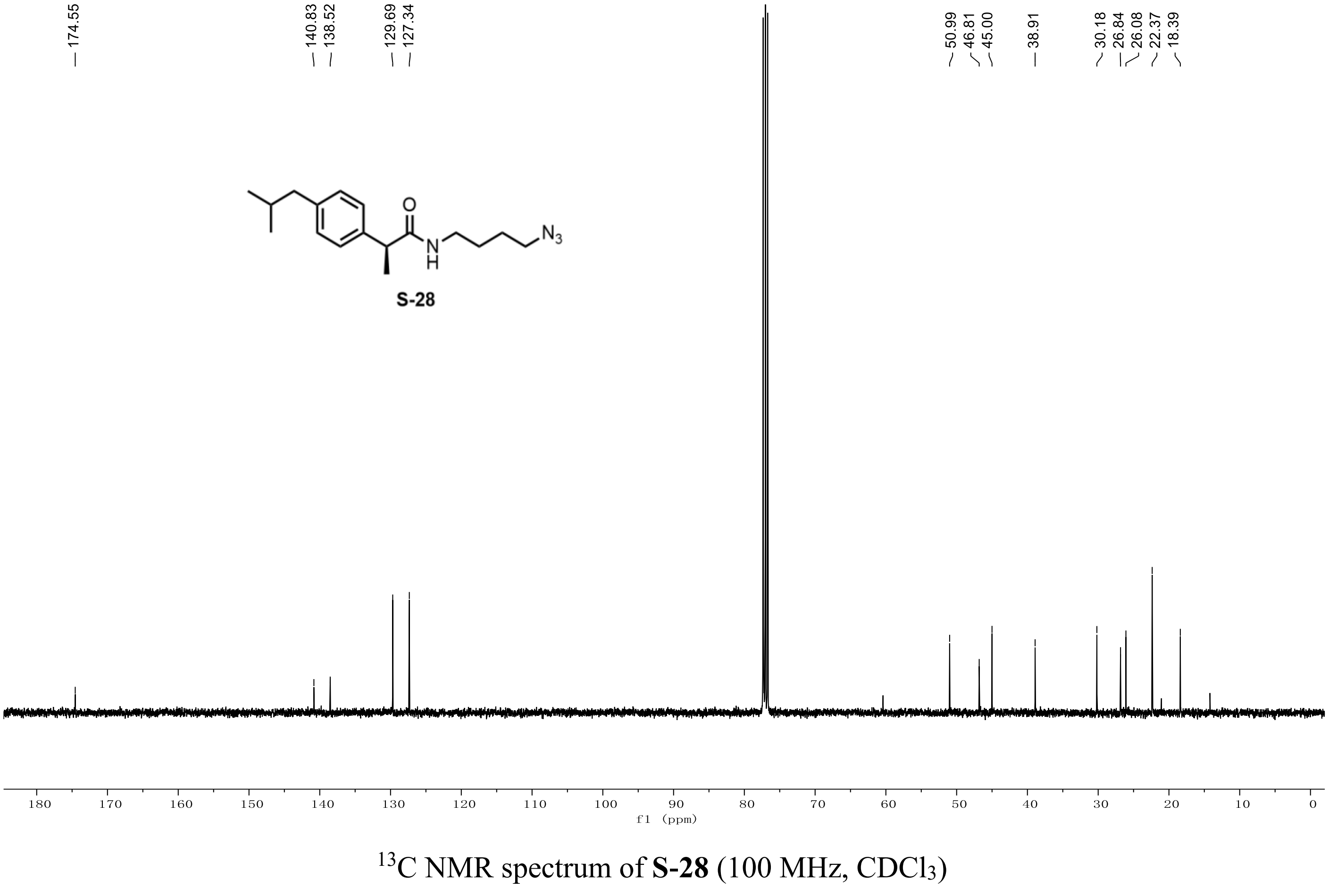

^{13}C NMR spectrum of **S-28** (100 MHz, $CDCl_3$)

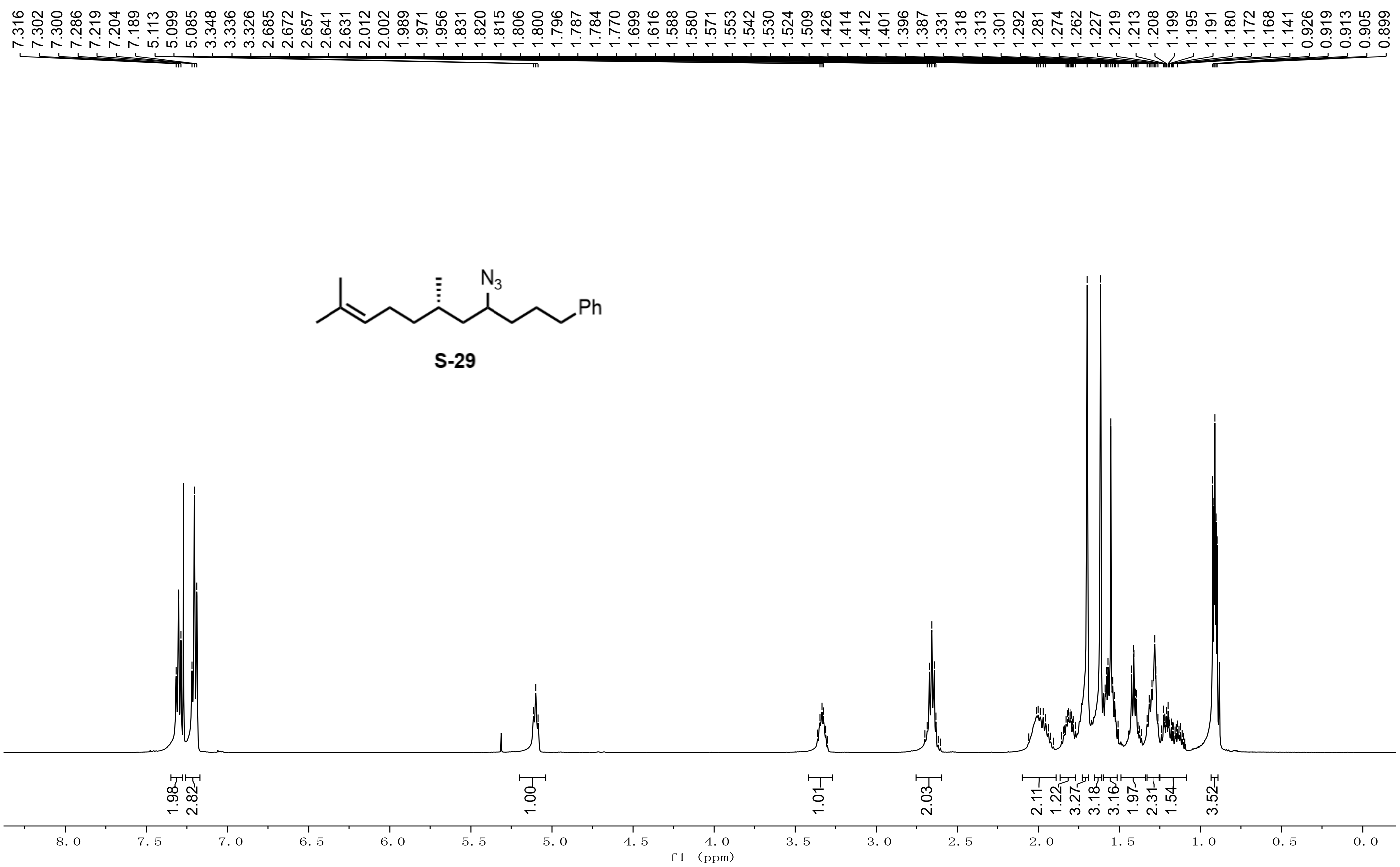

^{1}H NMR spectrum of **S-29** (500 MHz, $CDCl_3$)

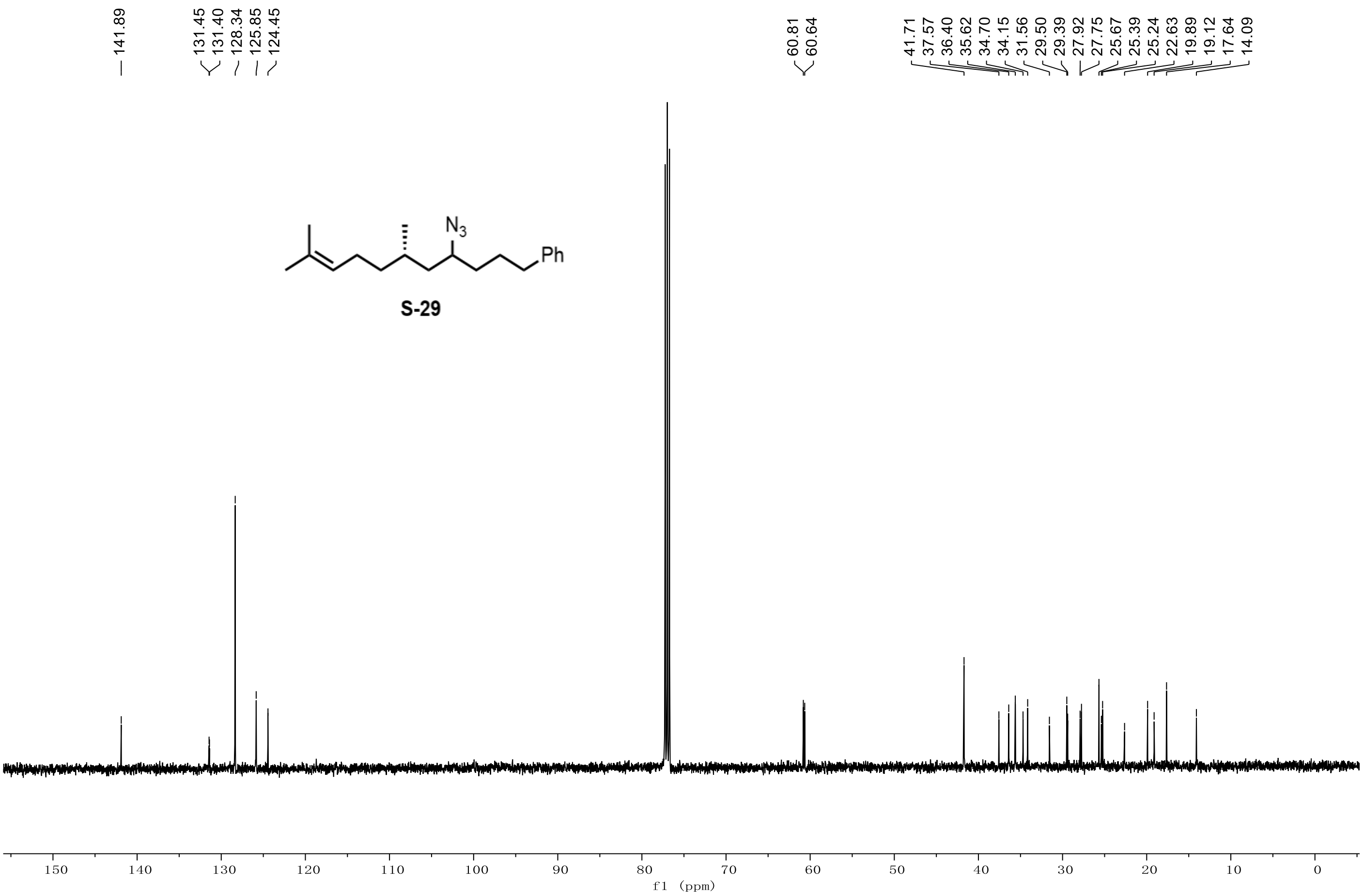

^{13}C NMR spectrum of **S-29** (125 MHz, $CDCl_3$)

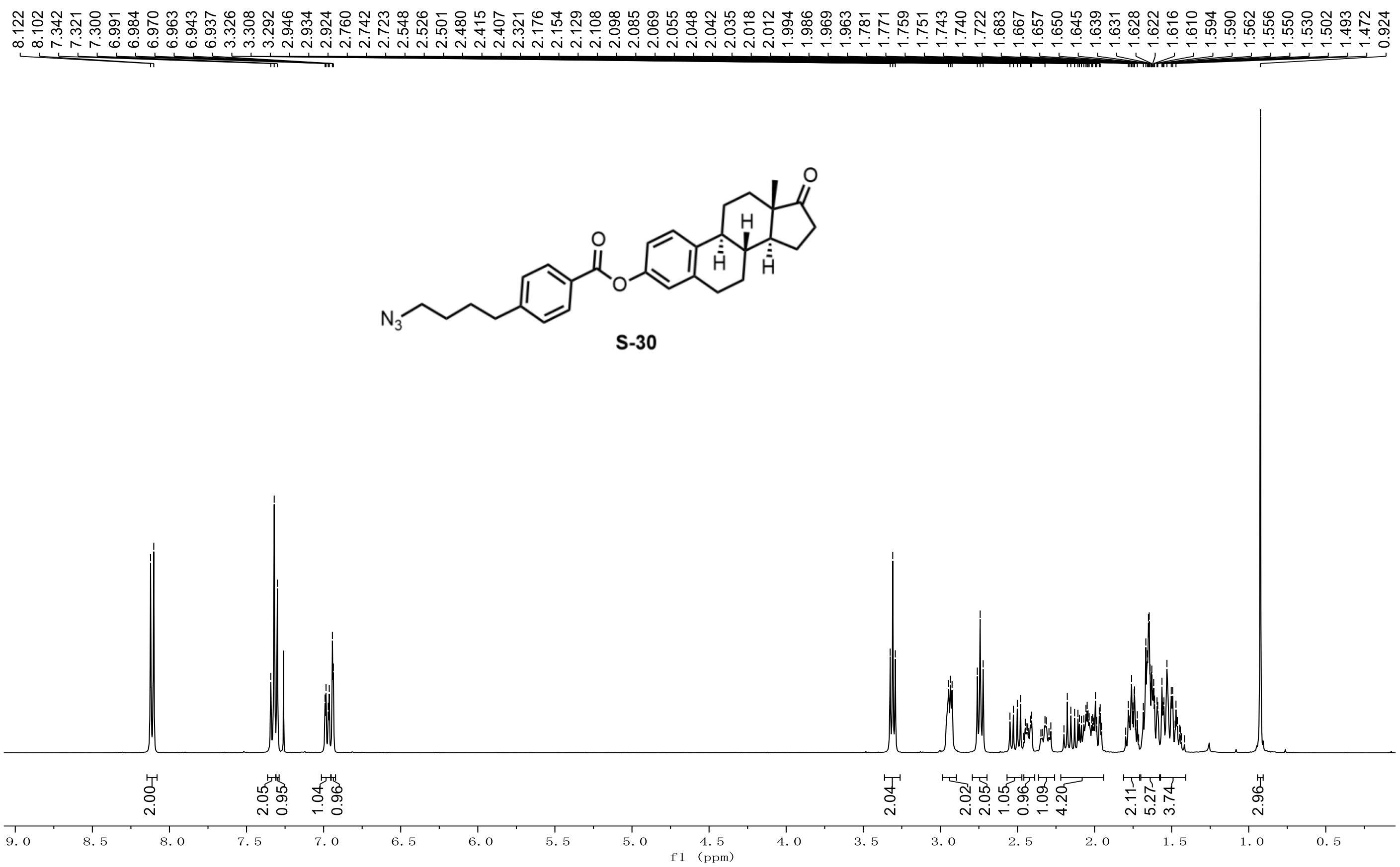

^{1}H NMR spectrum of **S-30** (400 MHz, $CDCl_3$)

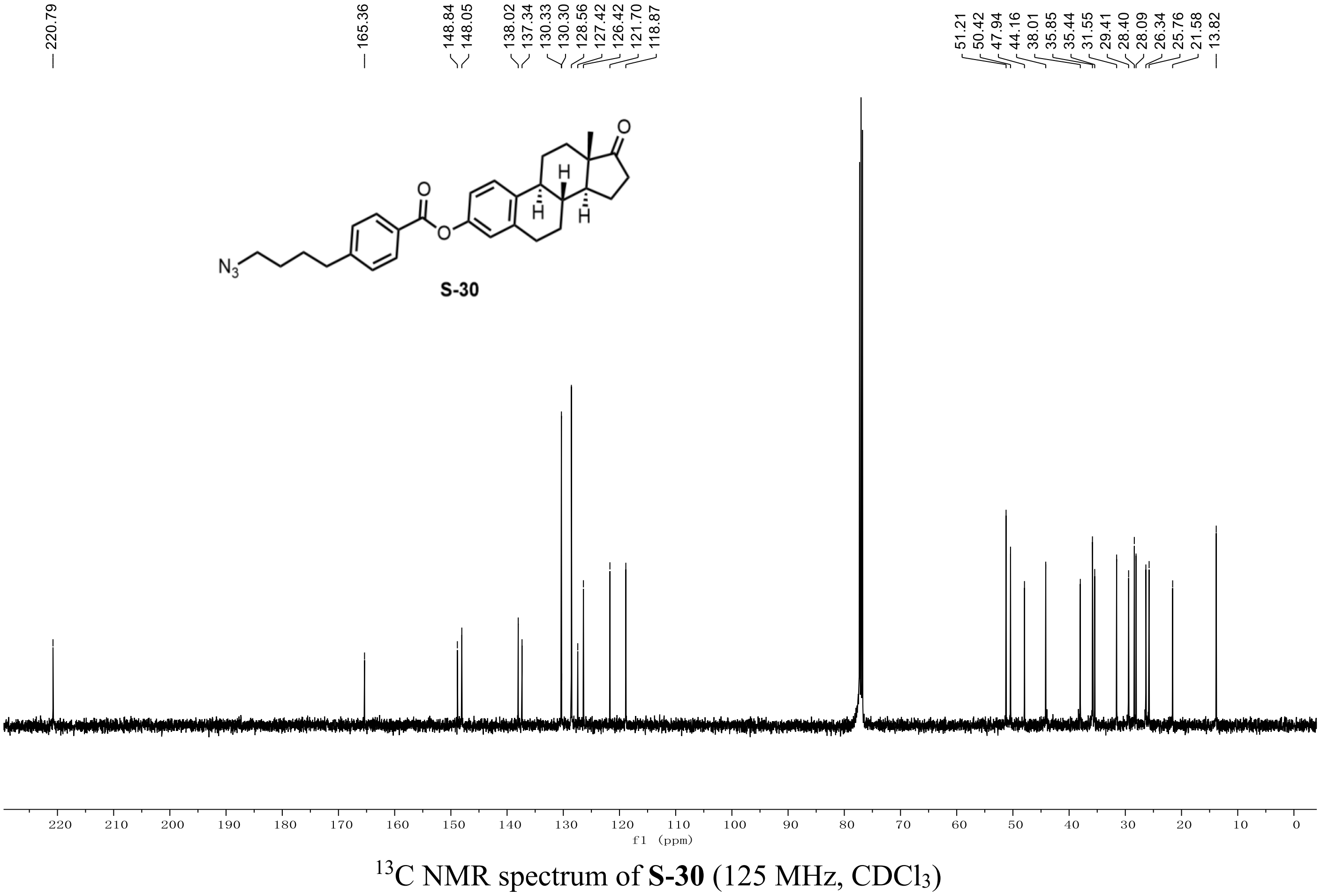

^{13}C NMR spectrum of **S-30** (125 MHz, $CDCl_3$)

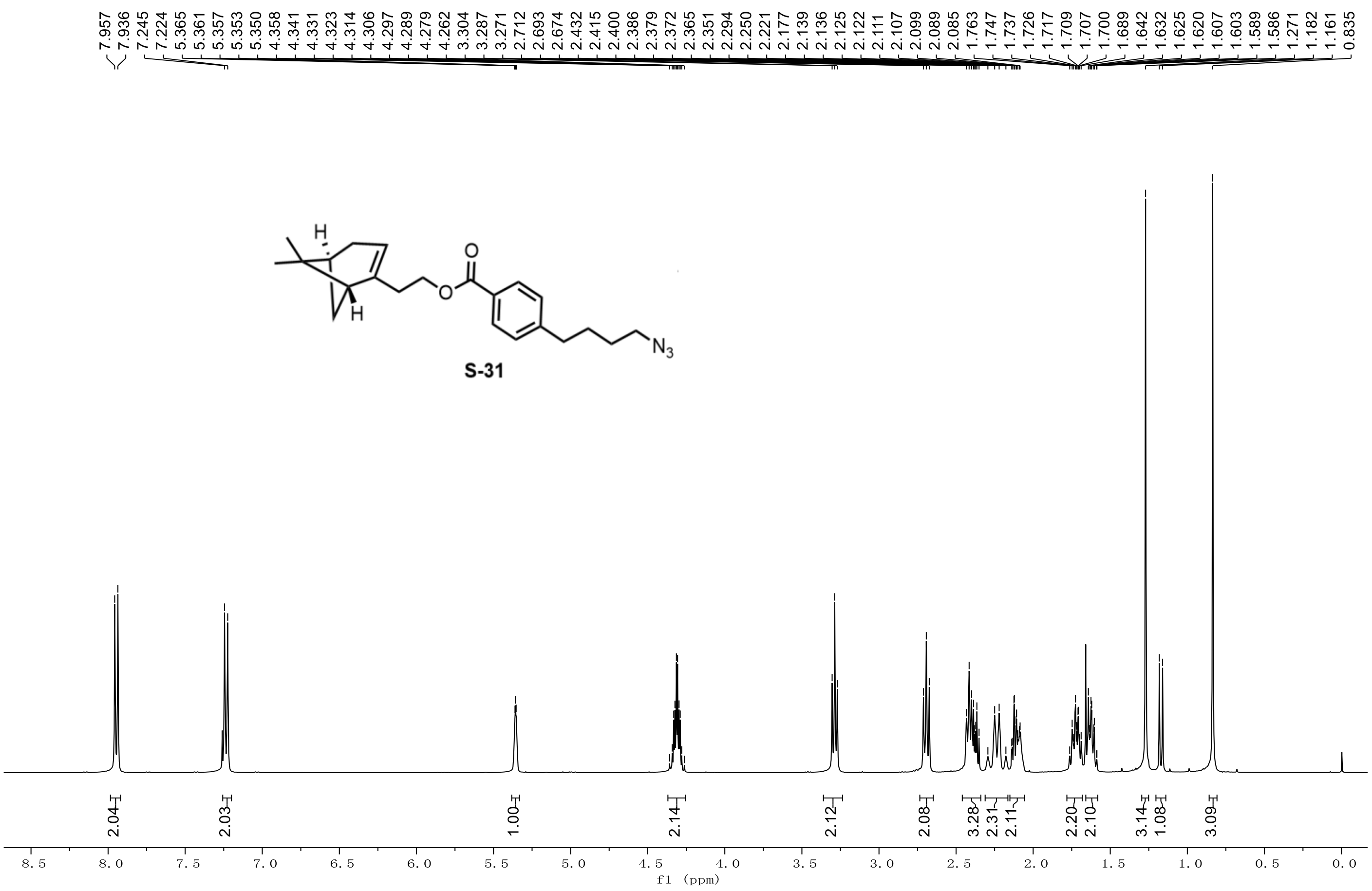

^{1}H NMR spectrum of **S-31** (400 MHz, $CDCl_3$)

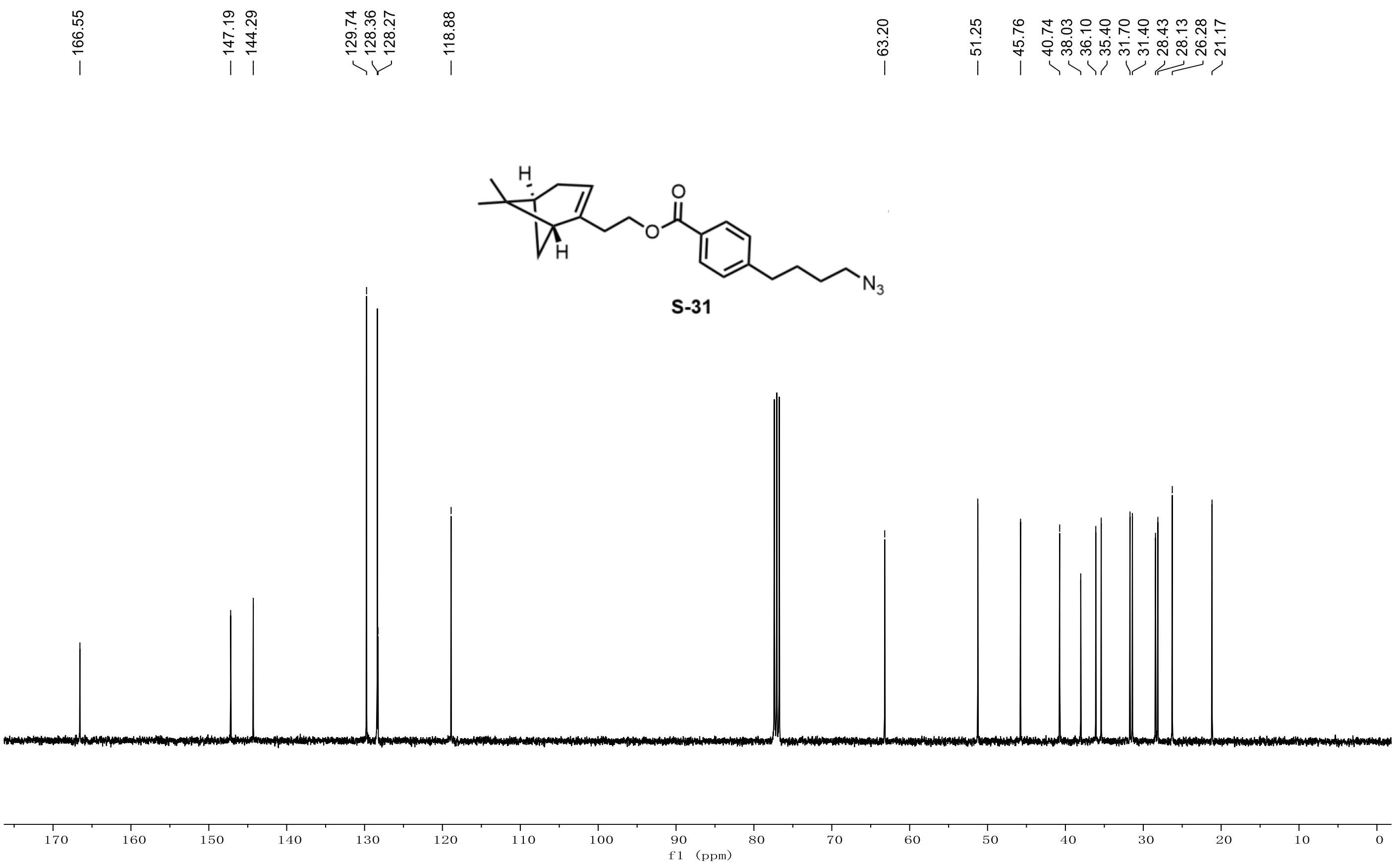

^{13}C NMR spectrum of **S-31** (100 MHz, $CDCl_3$)

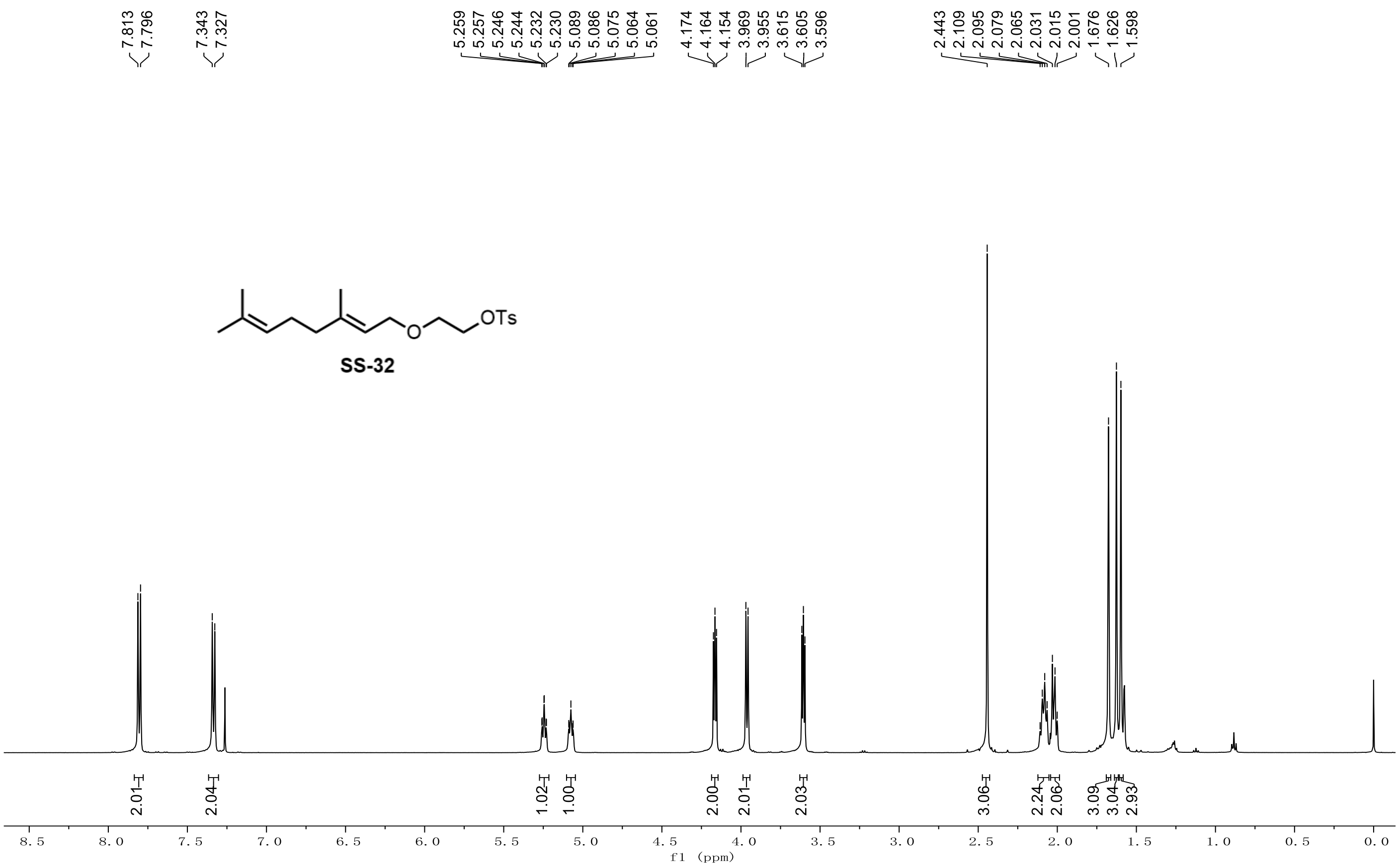

^{1}H NMR spectrum of **SS-32** (500 MHz, $CDCl_3$)

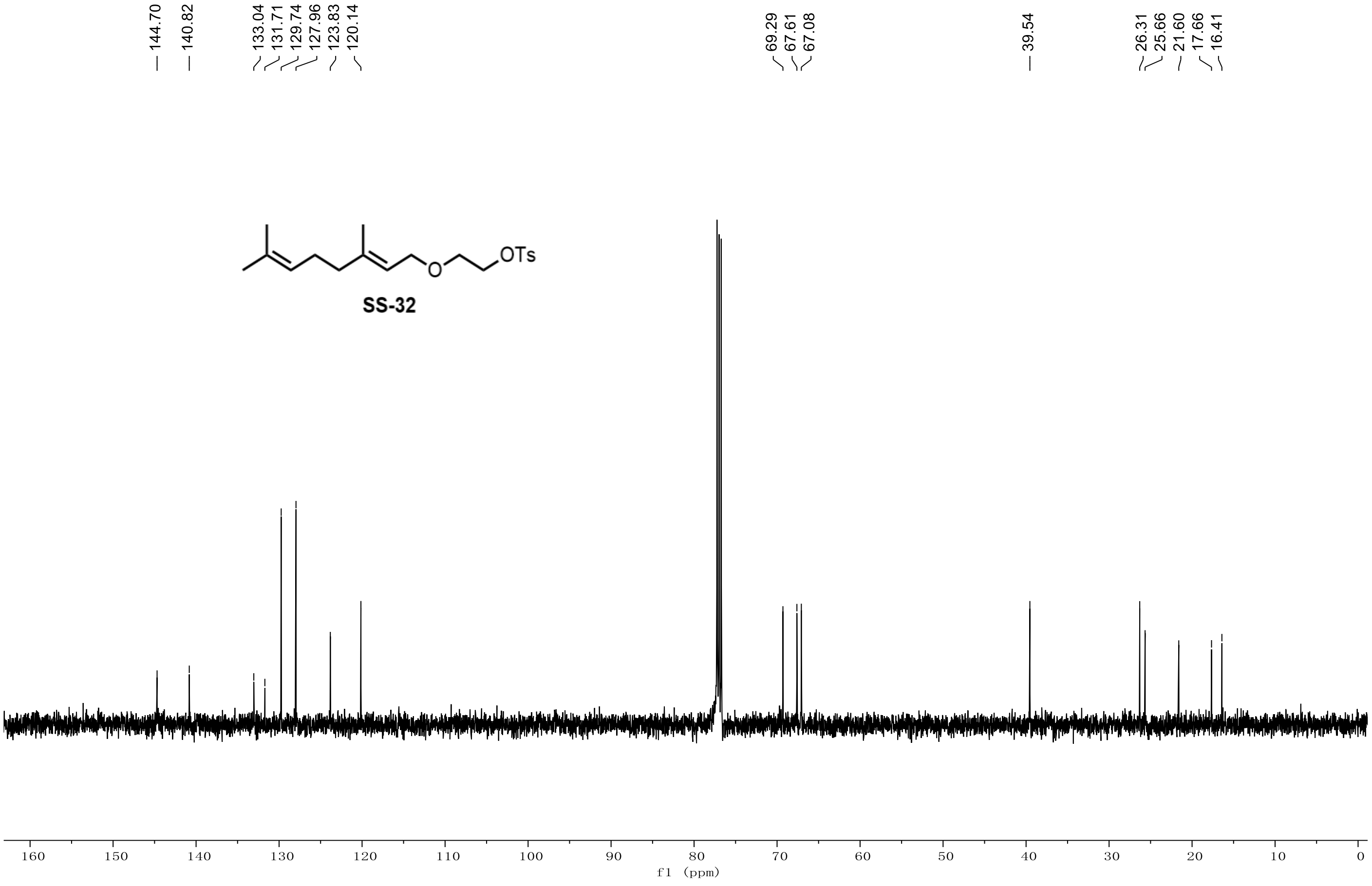

^{13}C NMR spectrum of **SS-32** (125 MHz, $CDCl_3$)

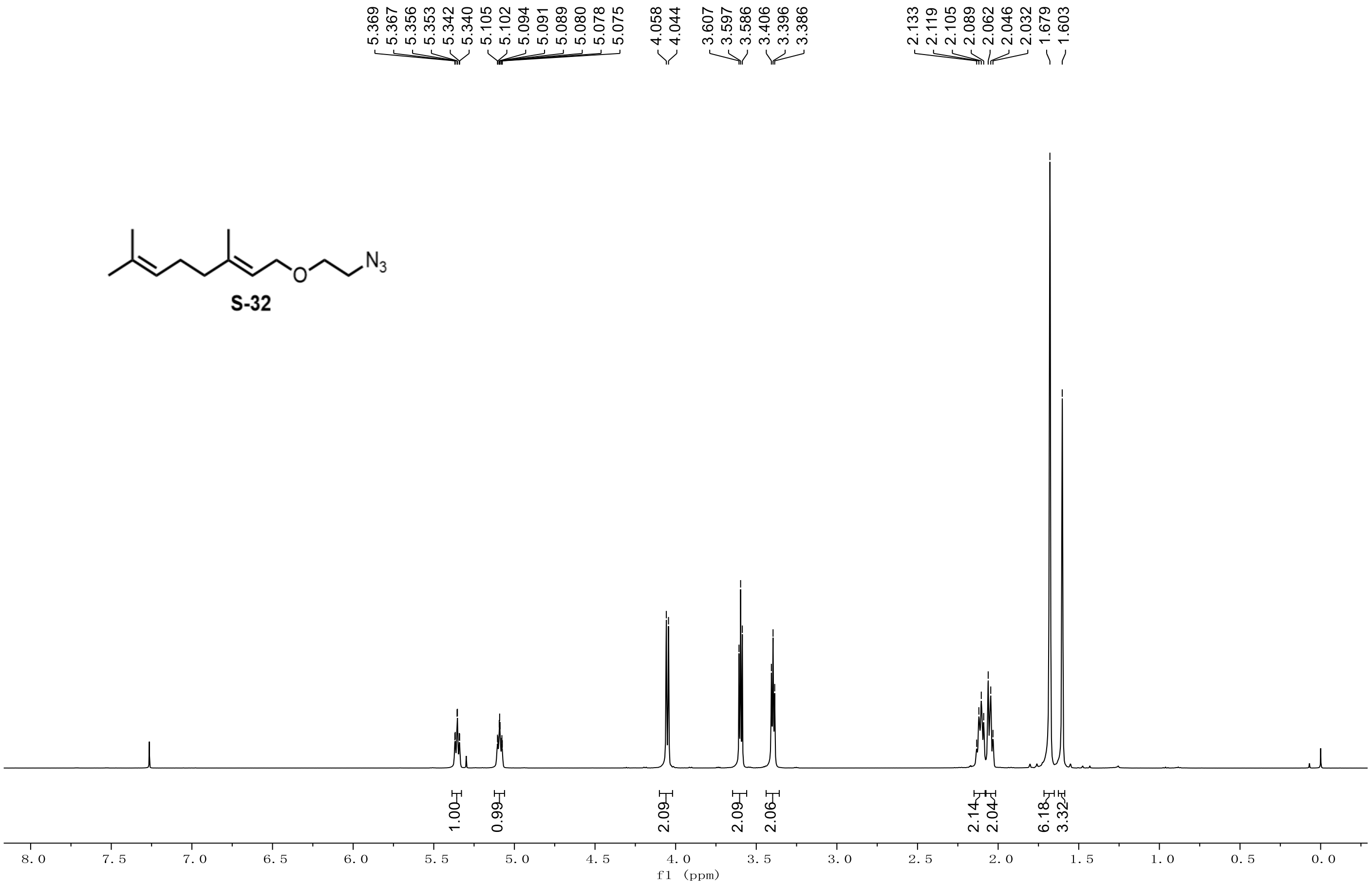

^{1}H NMR spectrum of **S-32** (500 MHz, $CDCl_3$)

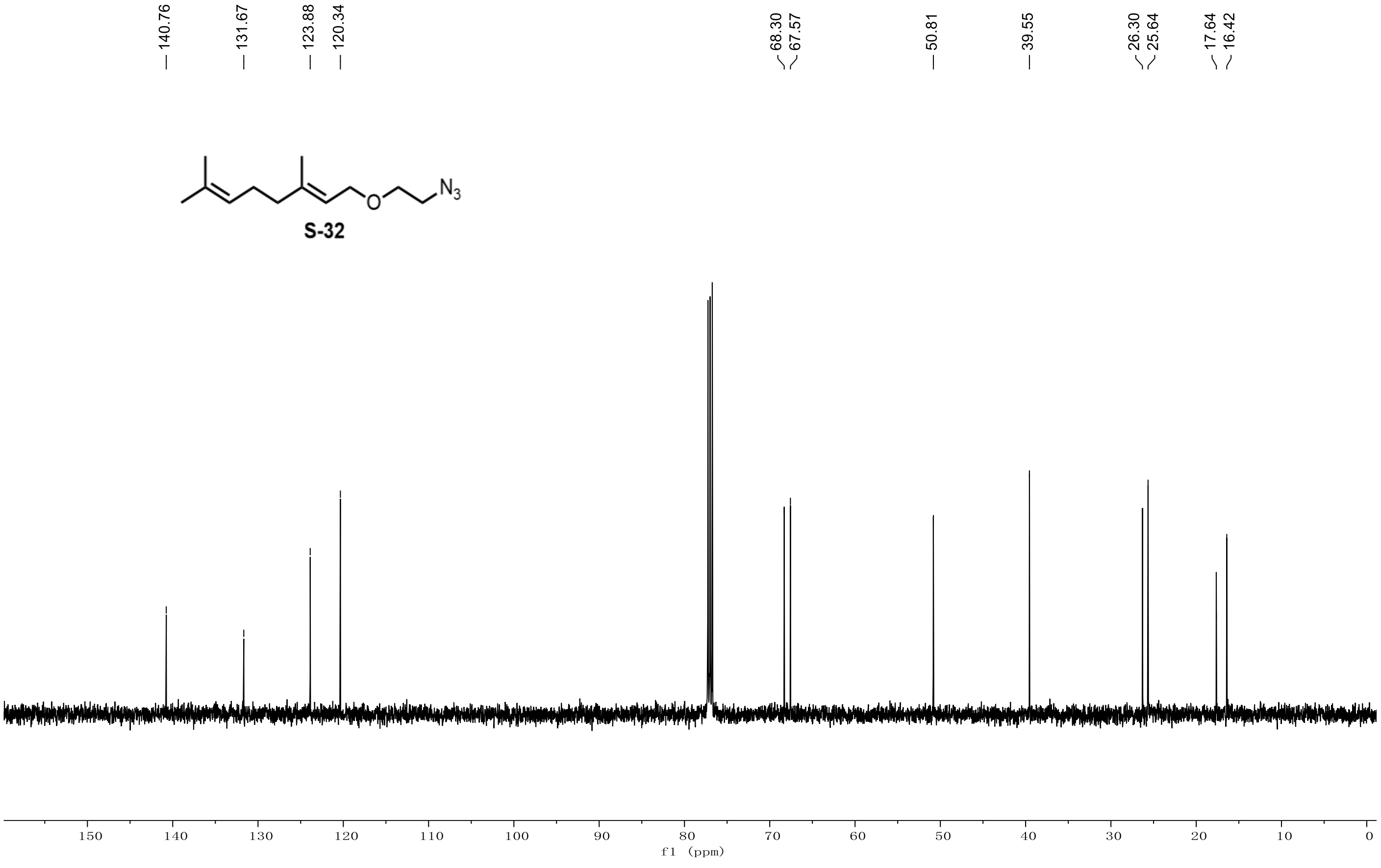

^{13}C NMR spectrum of **S-32** (125 MHz, $CDCl_3$)

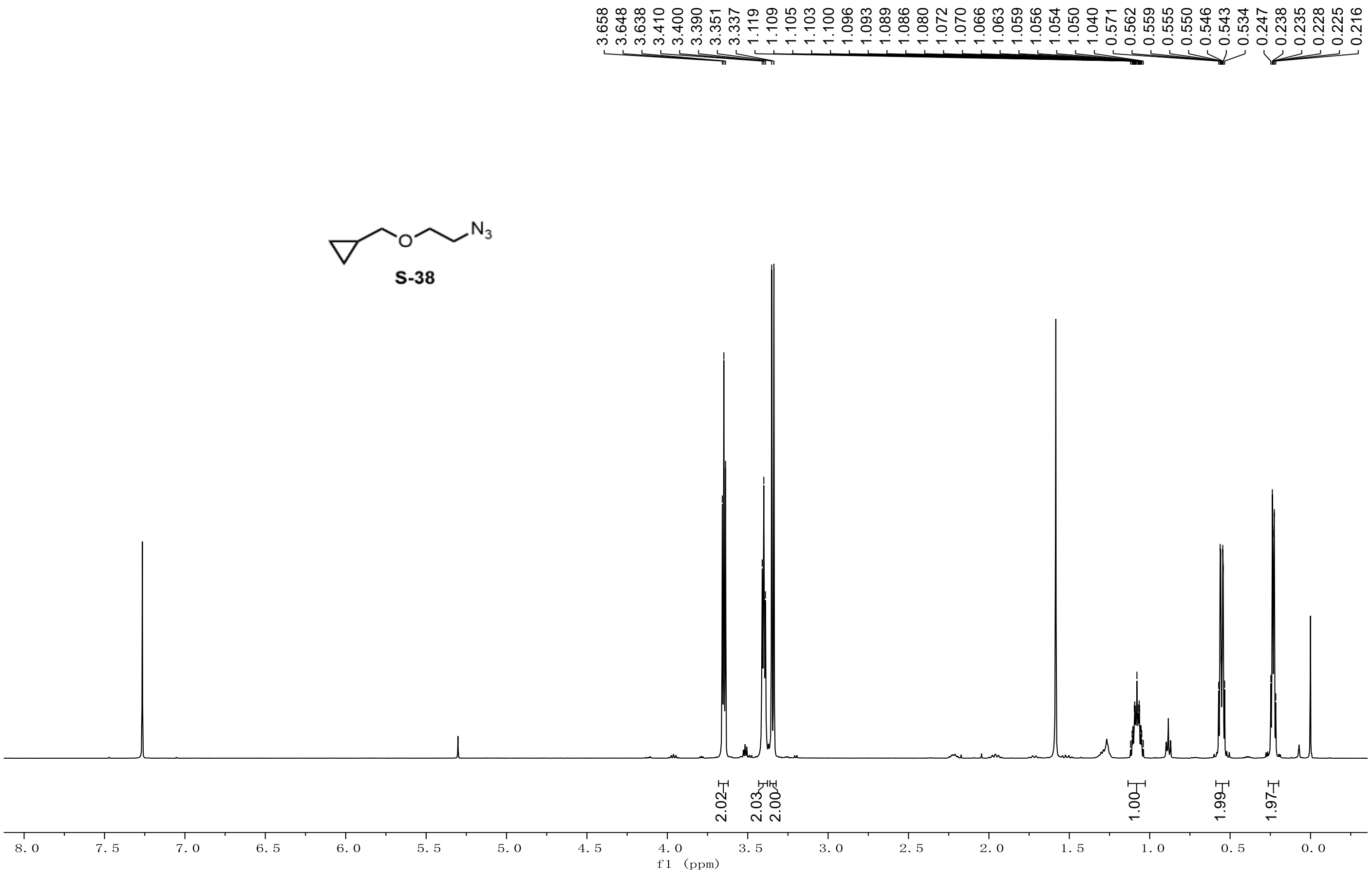

^{1}H NMR spectrum of **S-38** (500 MHz, $CDCl_3$)

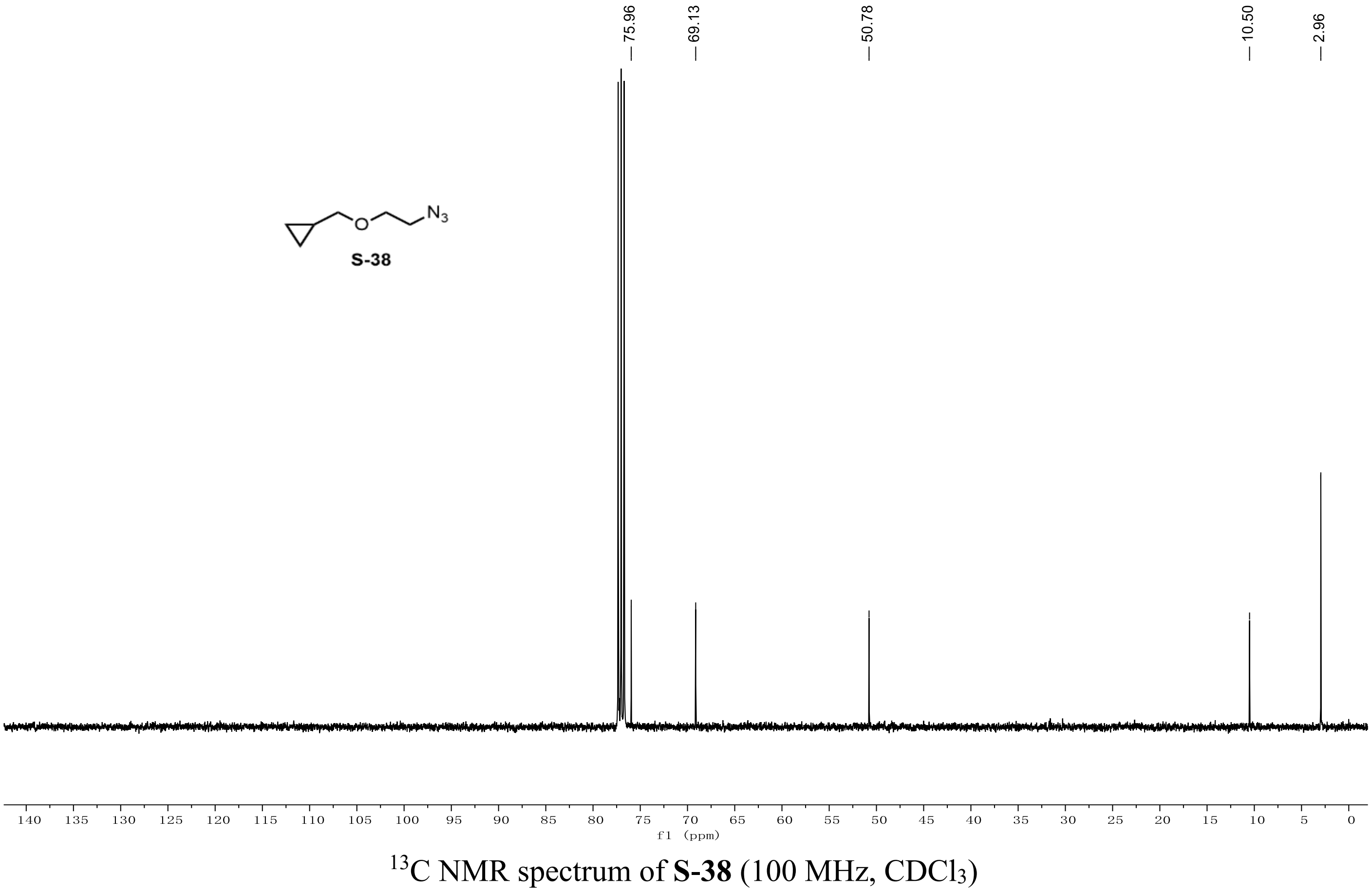

^{13}C NMR spectrum of **S-38** (100 MHz, $CDCl_3$)

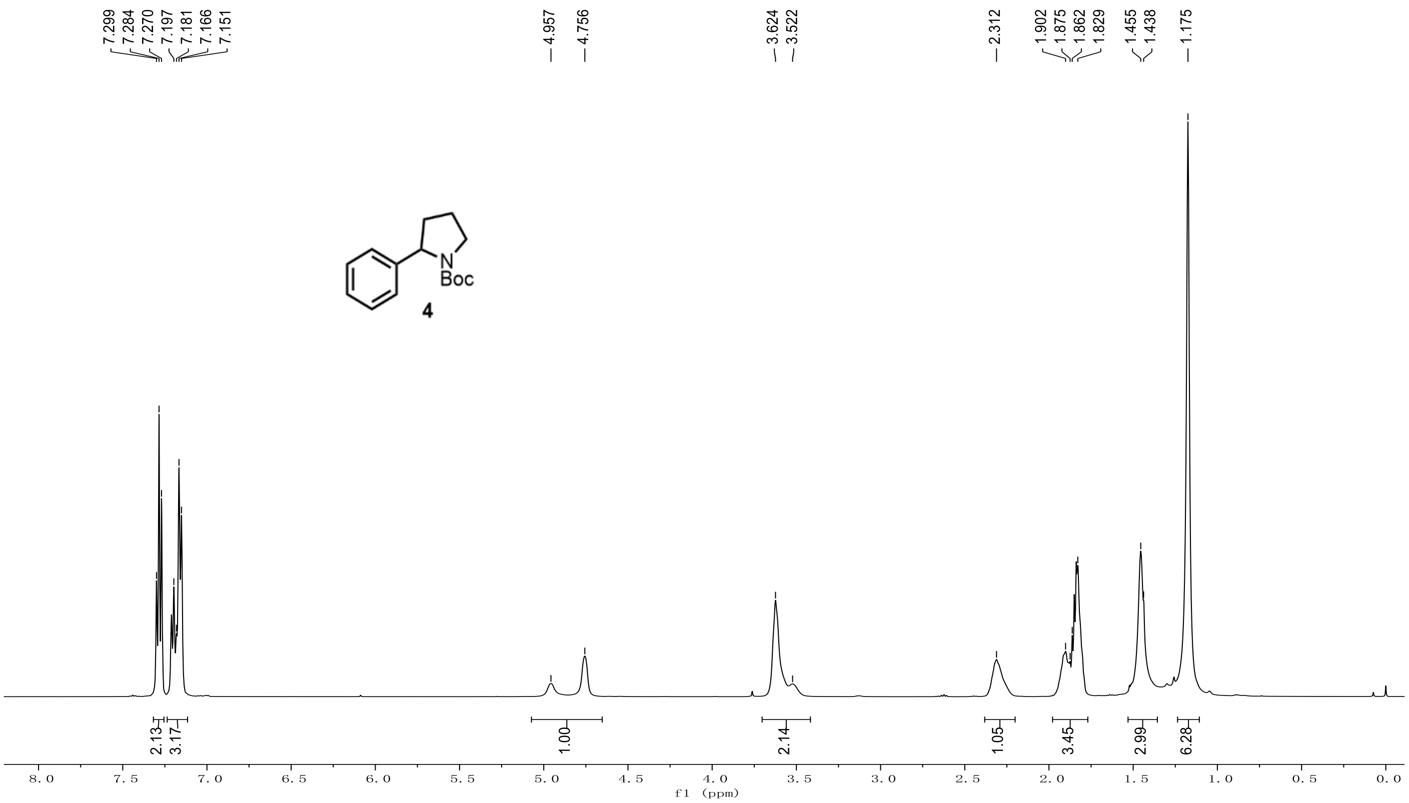

^{1}H NMR spectrum of **4** (500 MHz, $CDCl_3$)

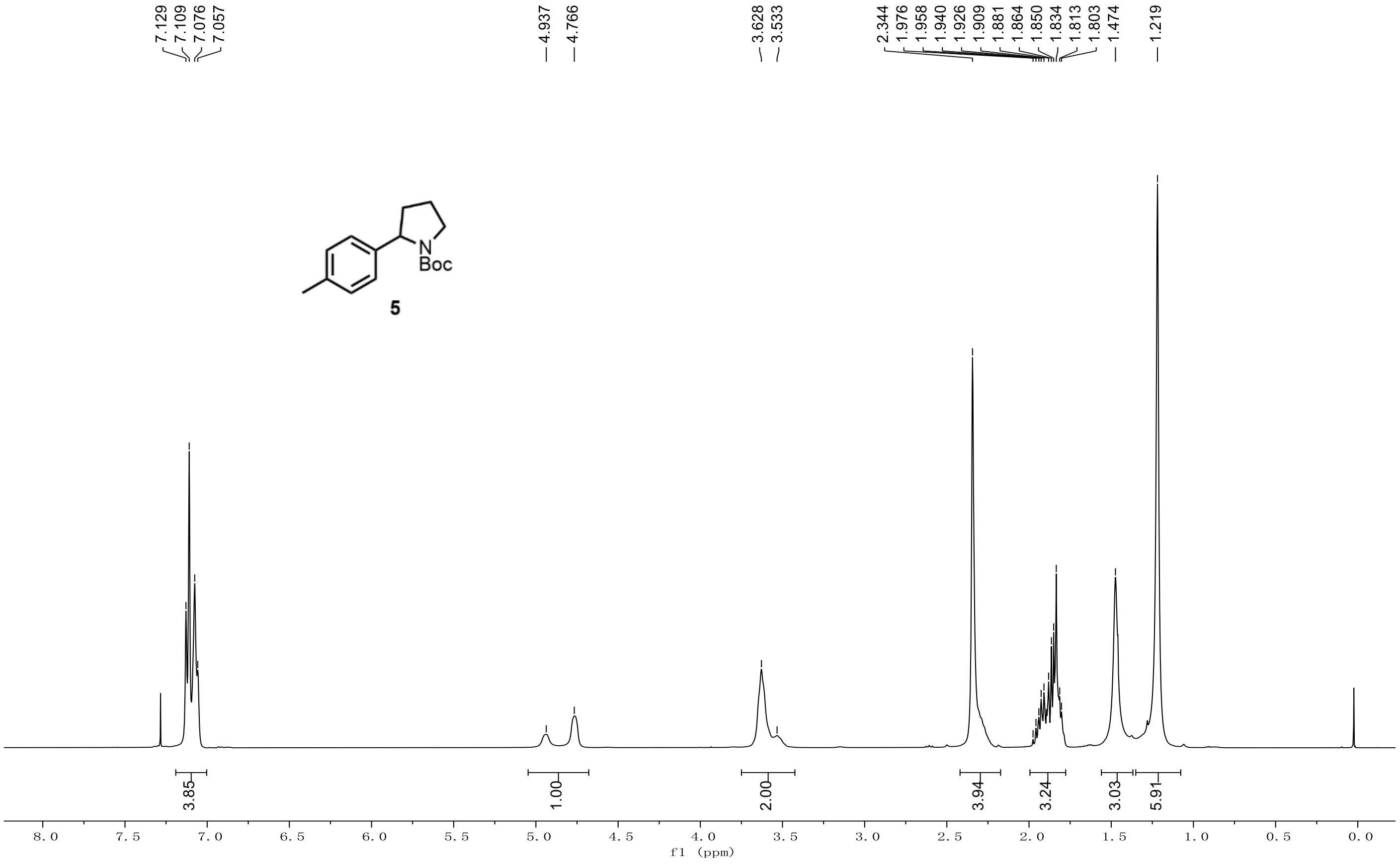

^{1}H NMR spectrum of **5** (400 MHz, $CDCl_3$)

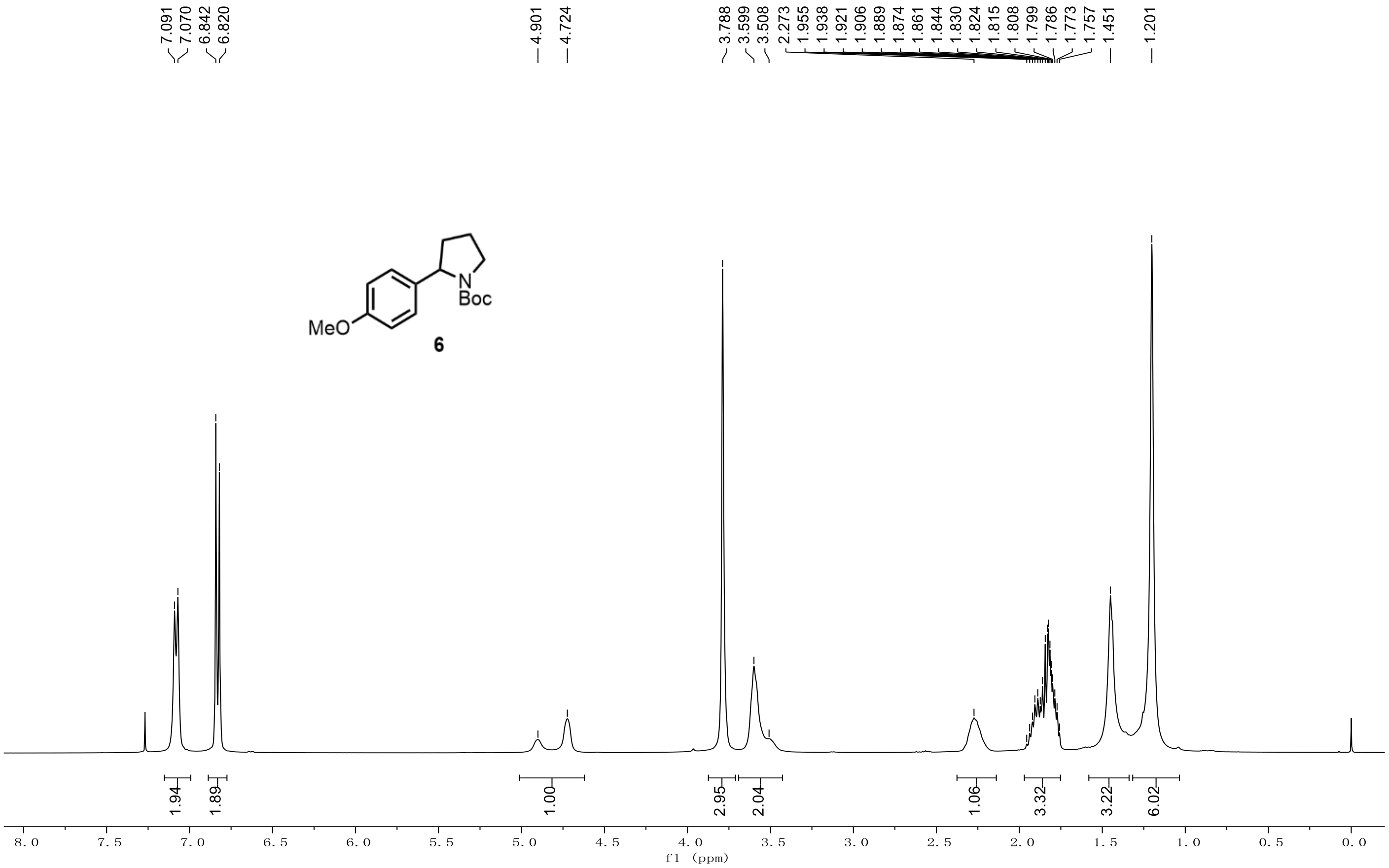

^{1}H NMR spectrum of **6** (400 MHz, $CDCl_3$)

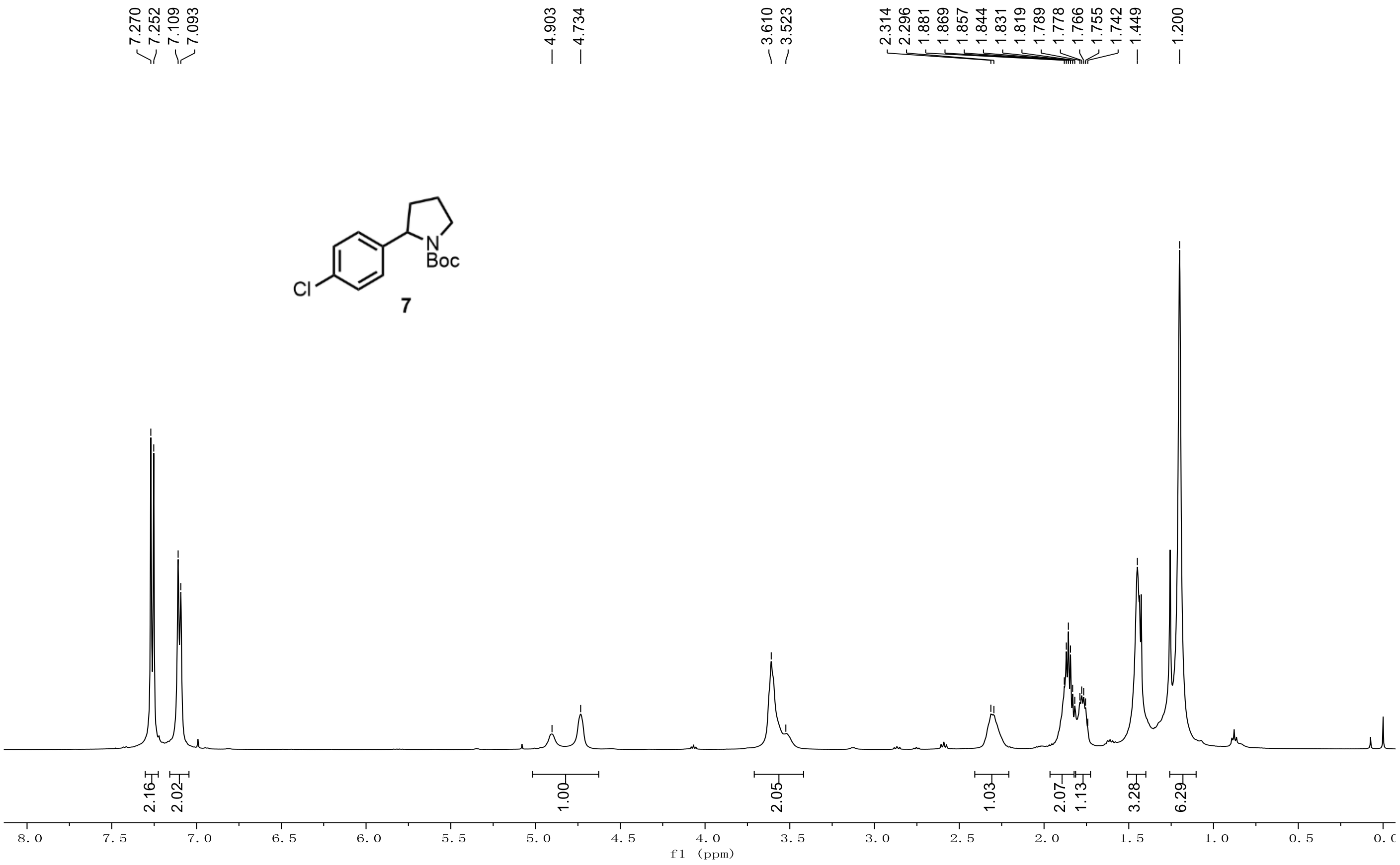

^{1}H NMR spectrum of **7** (500 MHz, $CDCl_3$)

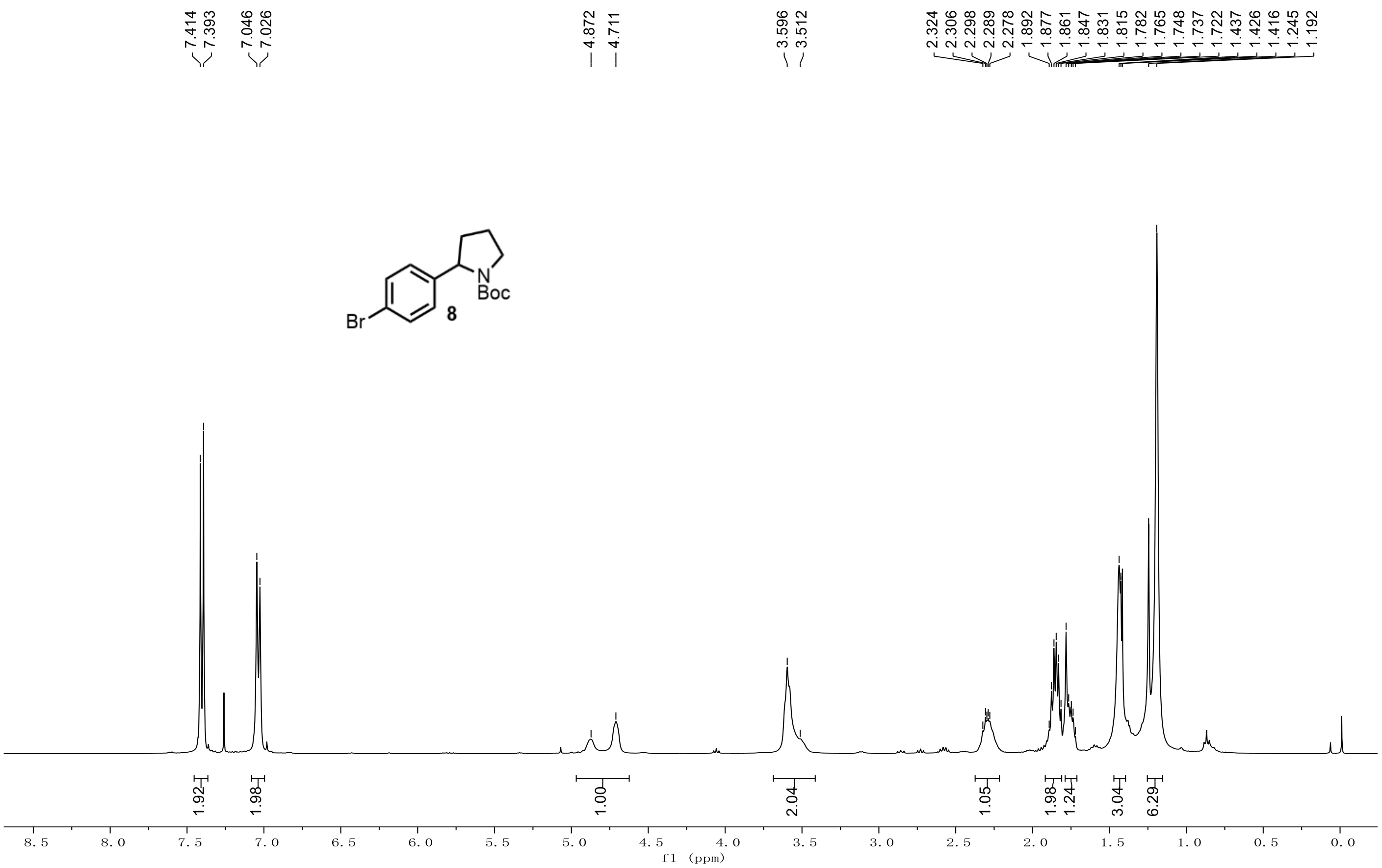

^{1}H NMR spectrum of **8** (400 MHz, $CDCl_3$)

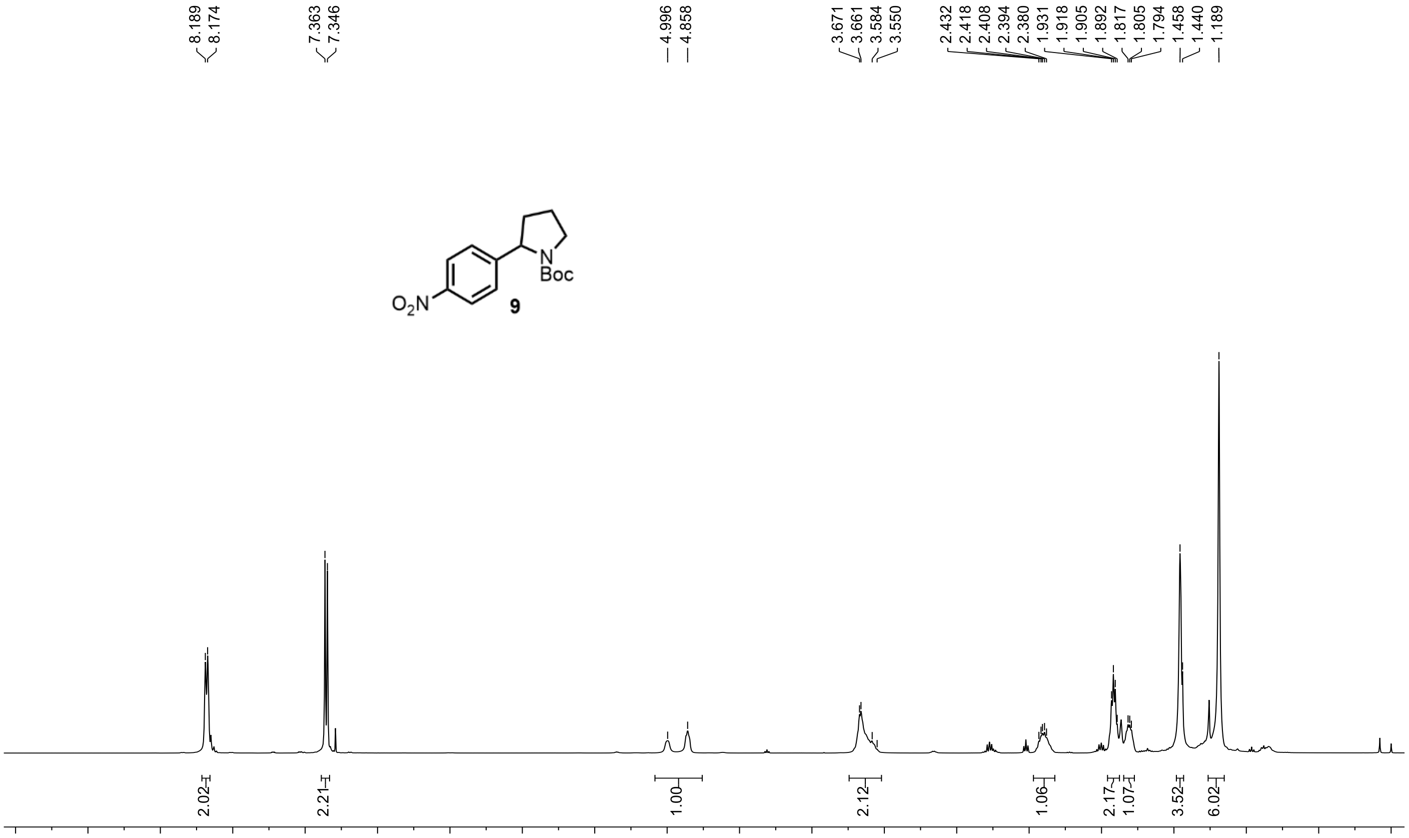

^{1}H NMR spectrum of **9** (500 MHz, $CDCl_3$)

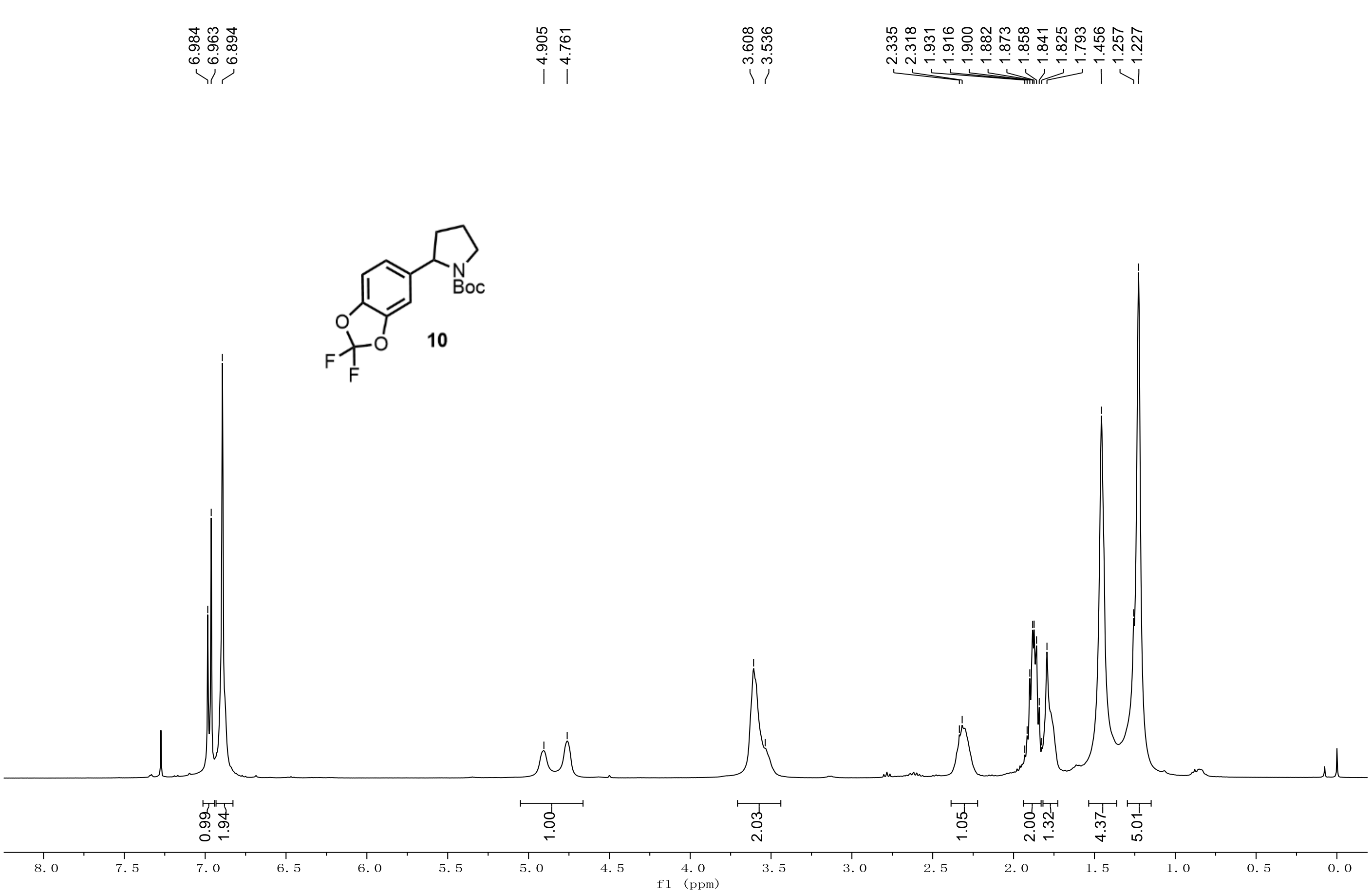

^{1}H NMR spectrum of **10** (400 MHz, $CDCl_3$)

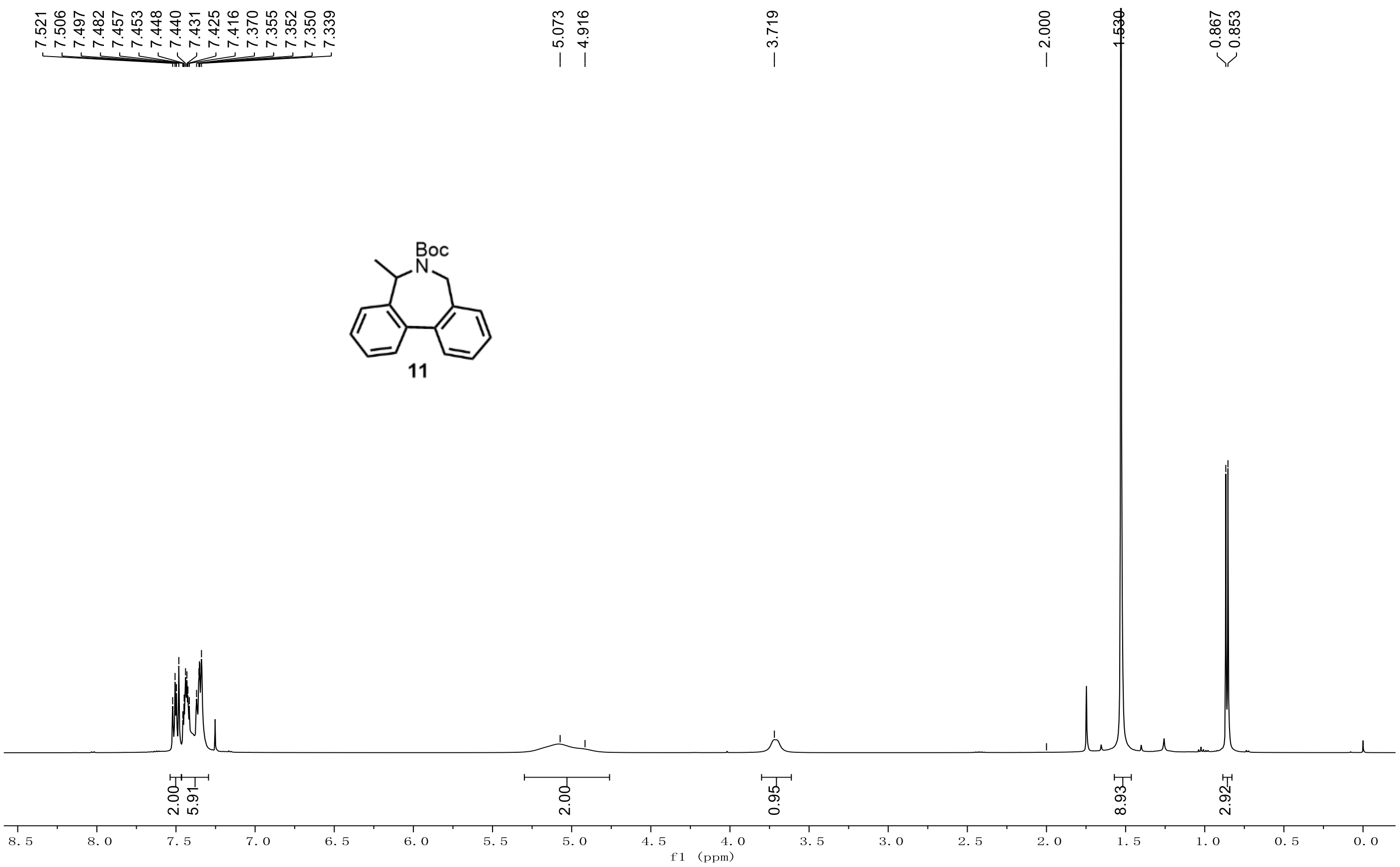

^{1}H NMR spectrum of **11** (500 MHz, $CDCl_3$)

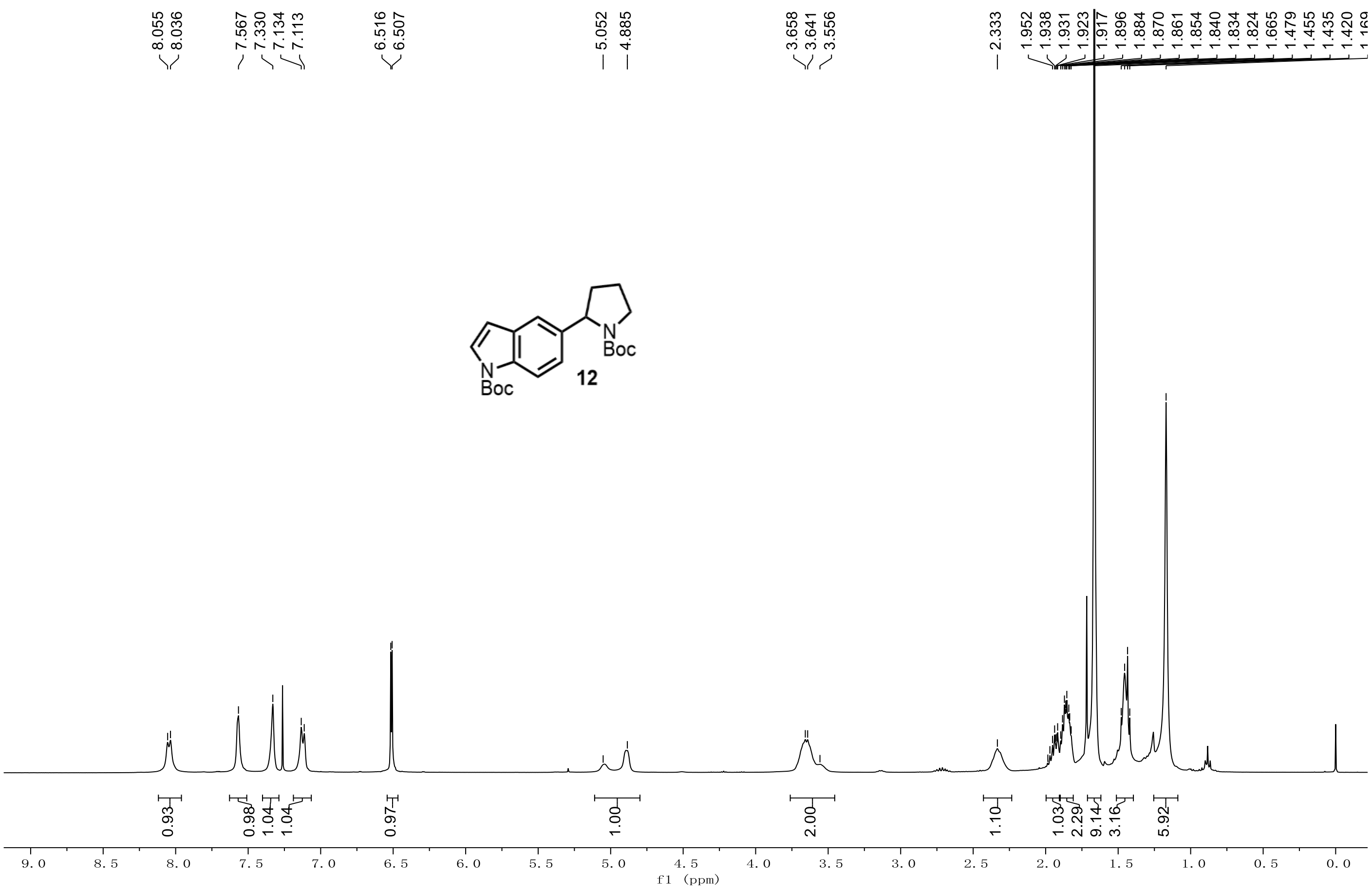

^1H NMR spectrum of **12** (400 MHz, $CDCl_3$)

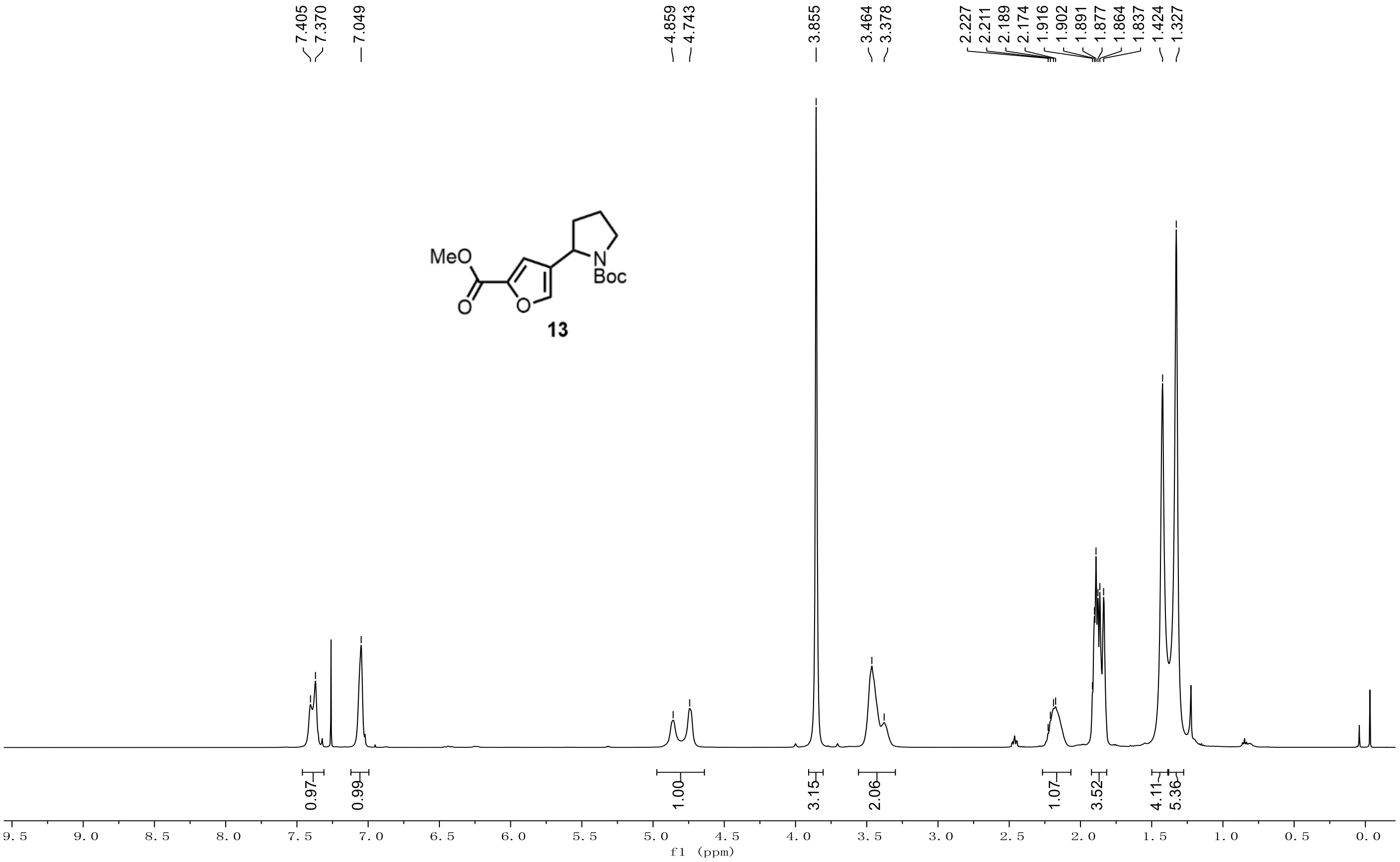

^1H NMR spectrum of **13** (500 MHz, $CDCl_3$)

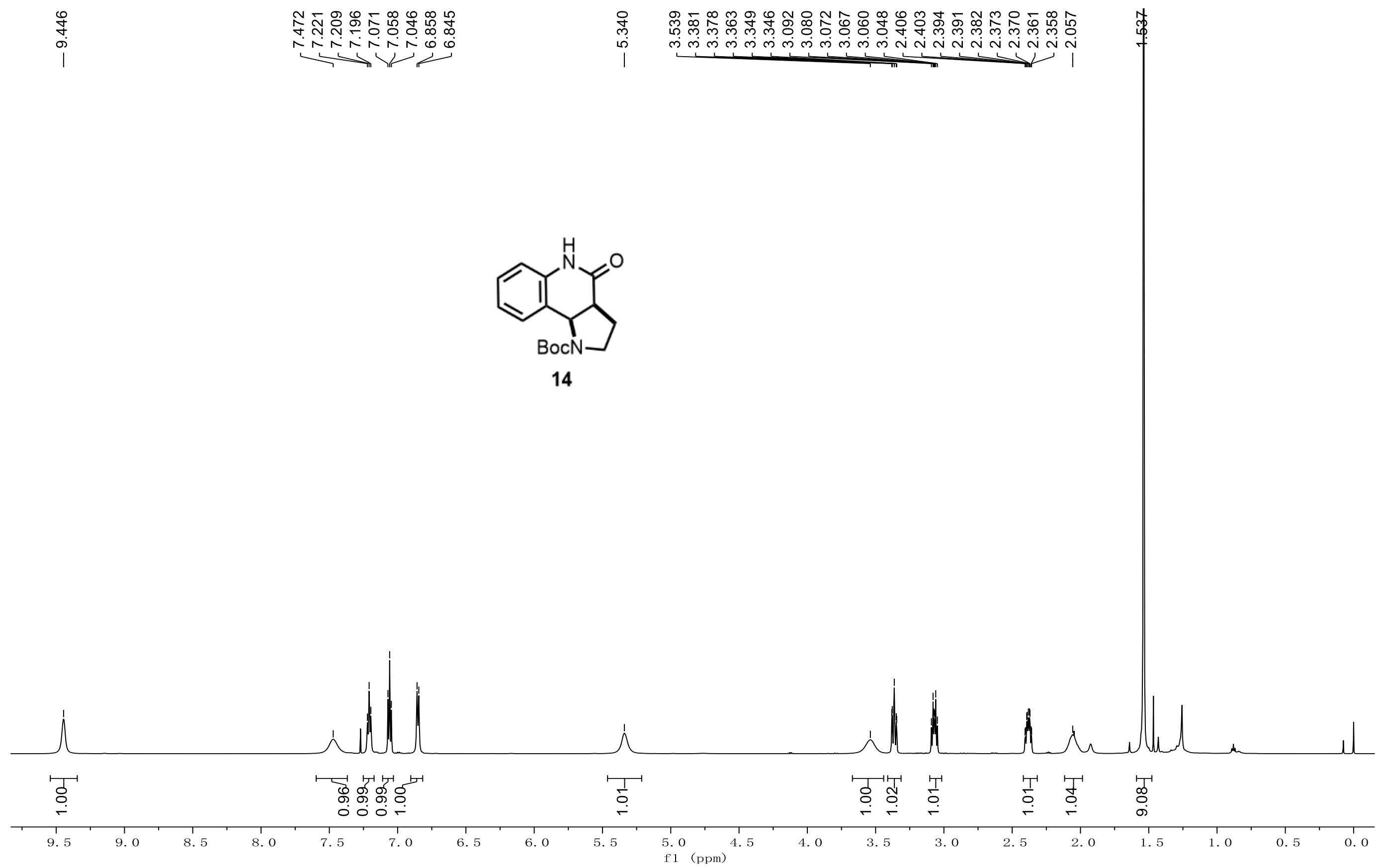

^{1}H NMR spectrum of **14** (600 MHz, $CDCl_3$)

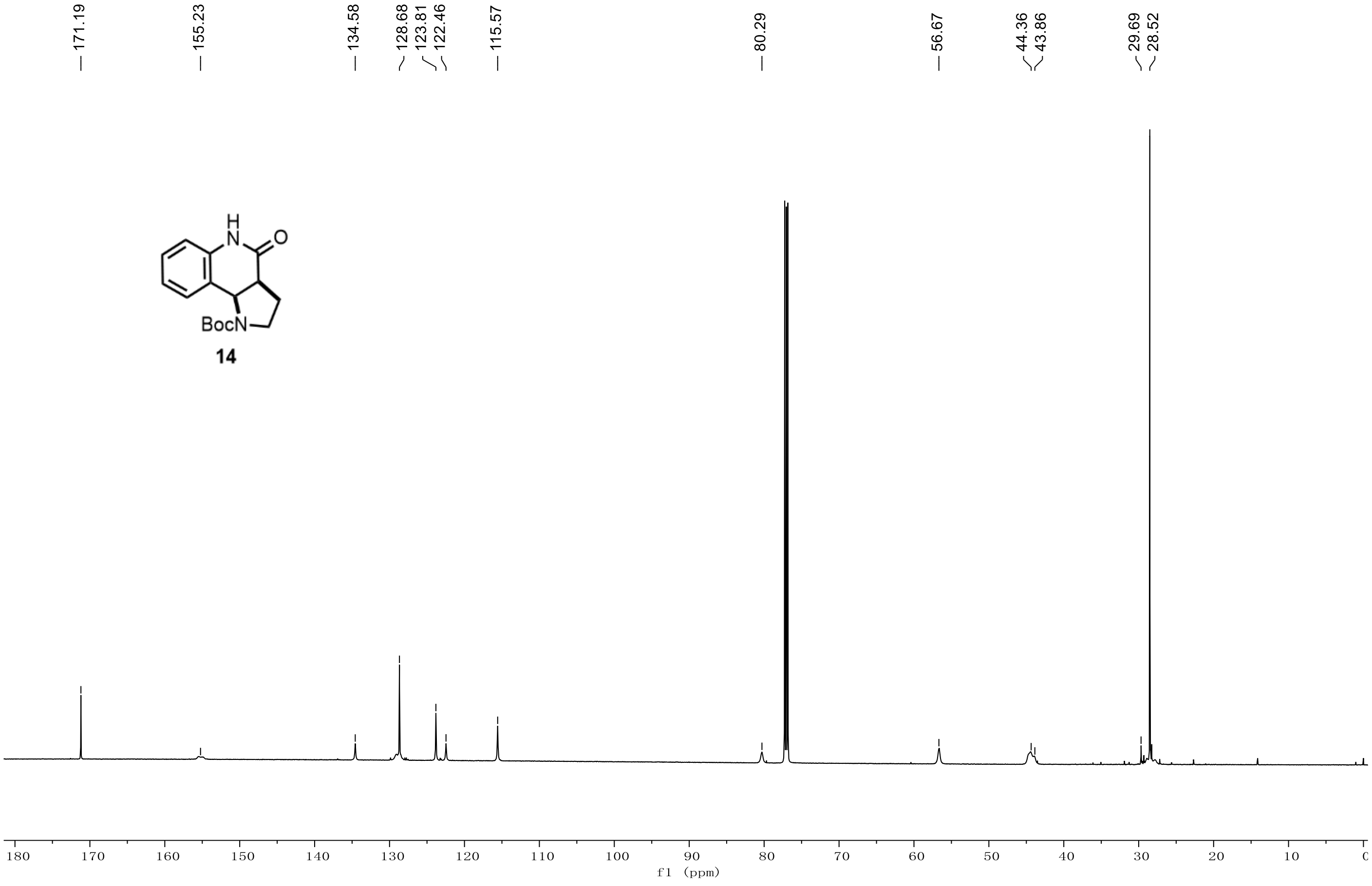

^{13}C NMR spectrum of **14** (150 MHz, $CDCl_3$)

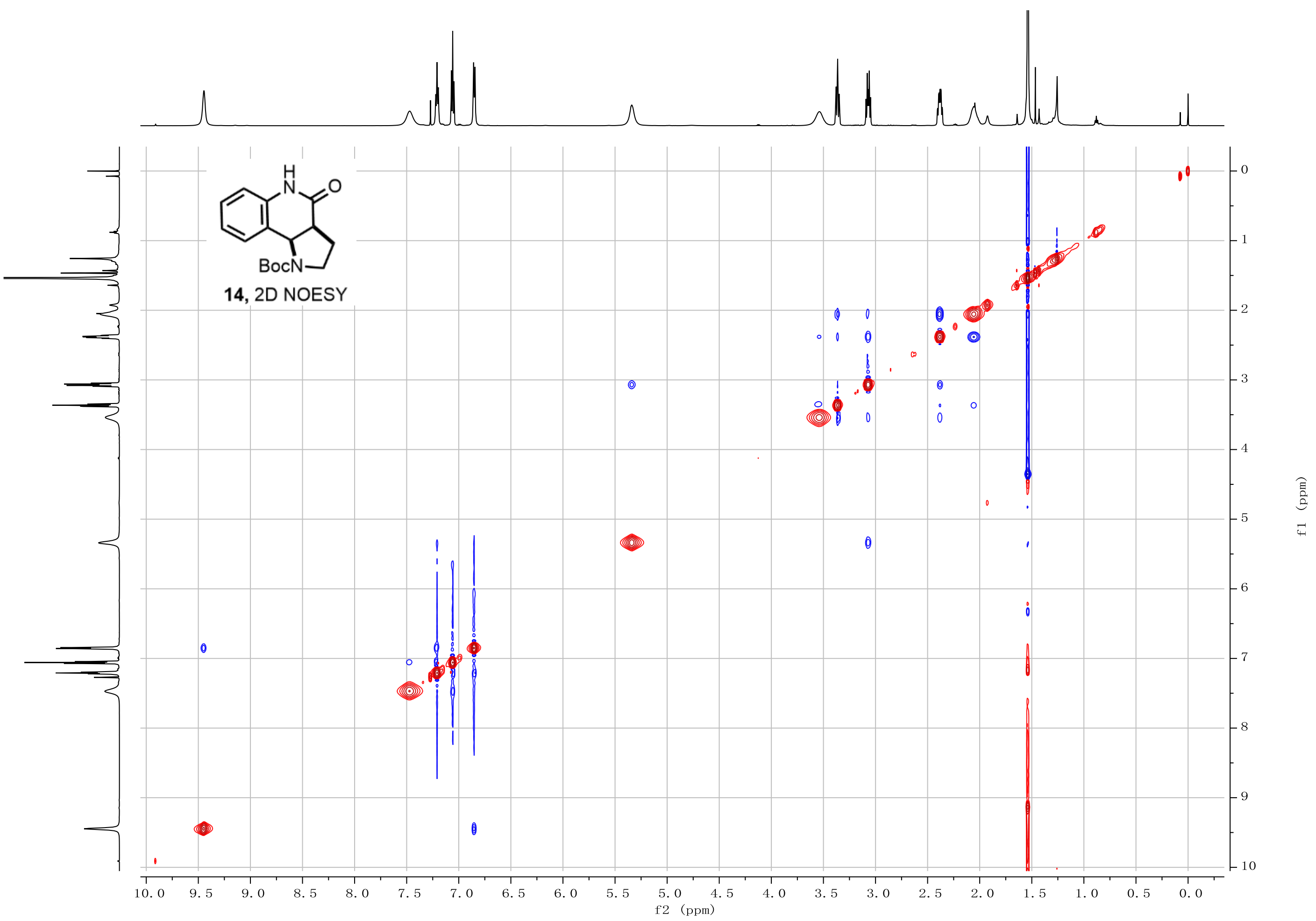

2D ^{1}H-^{1}H NOESY spectrum of **14** (600 MHz, $CDCl_3$)

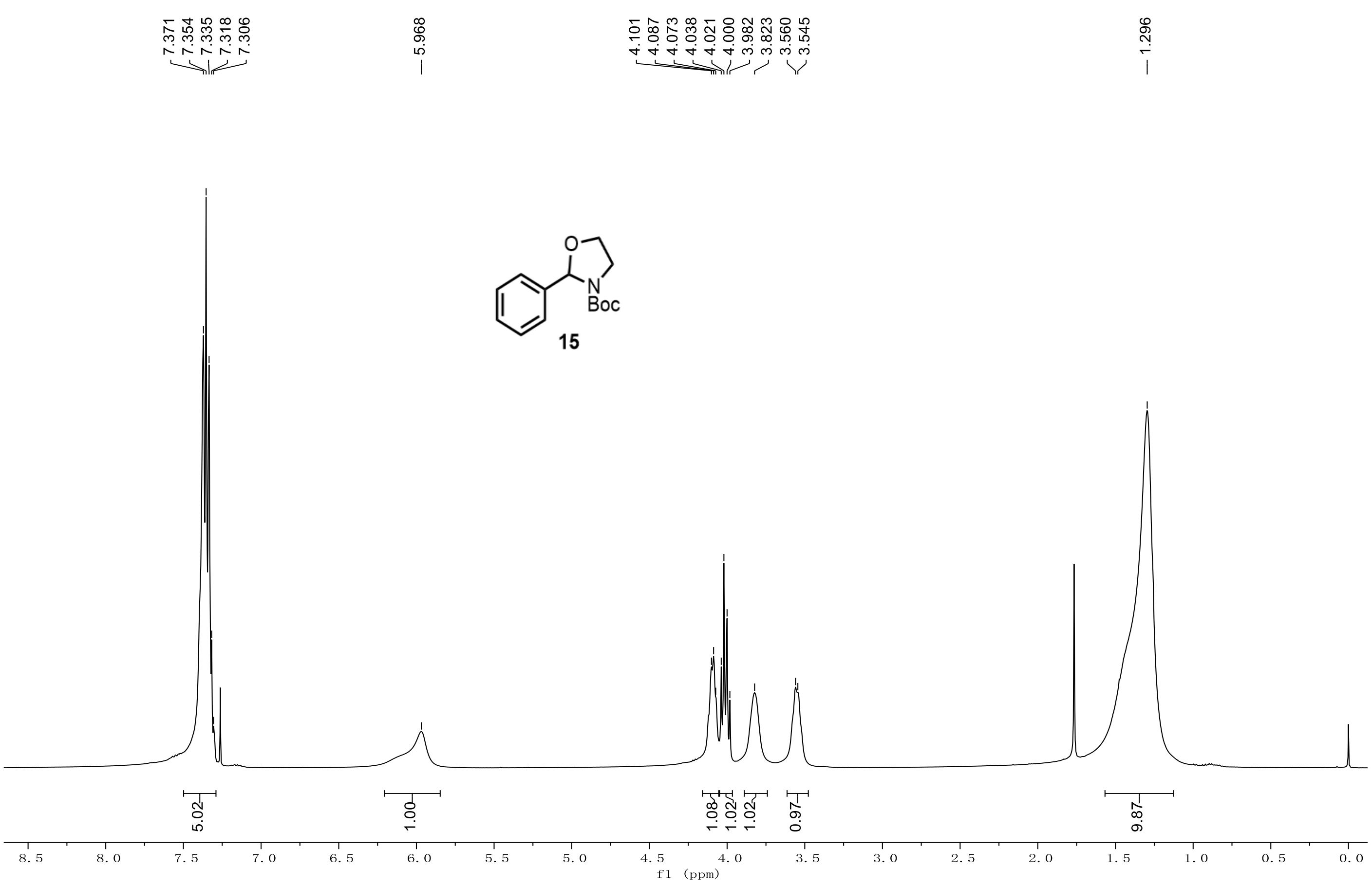

^{1}H NMR spectrum of **15** (400 MHz, $CDCl_3$)

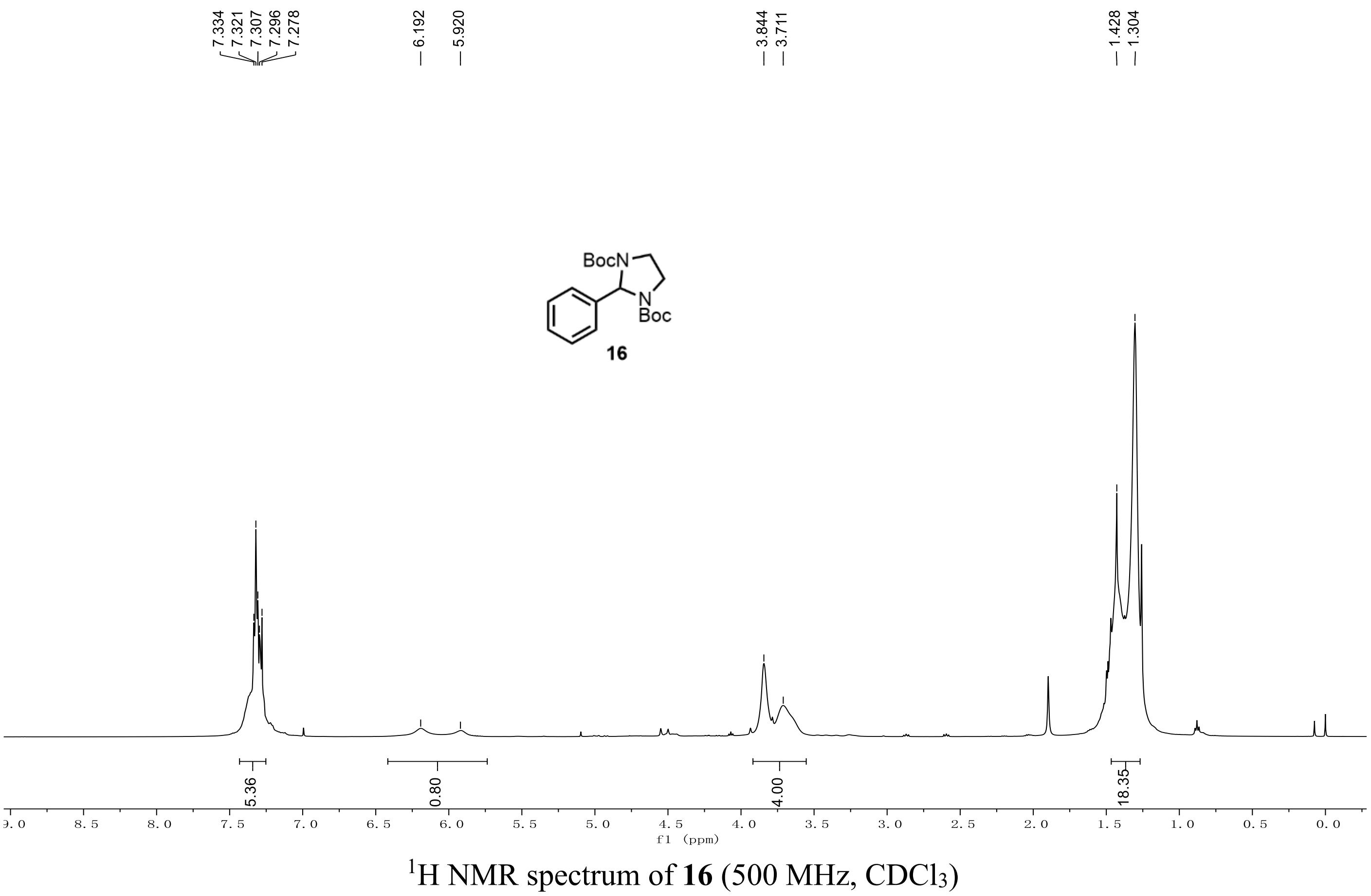

^{1}H NMR spectrum of **16** (500 MHz, $CDCl_3$)

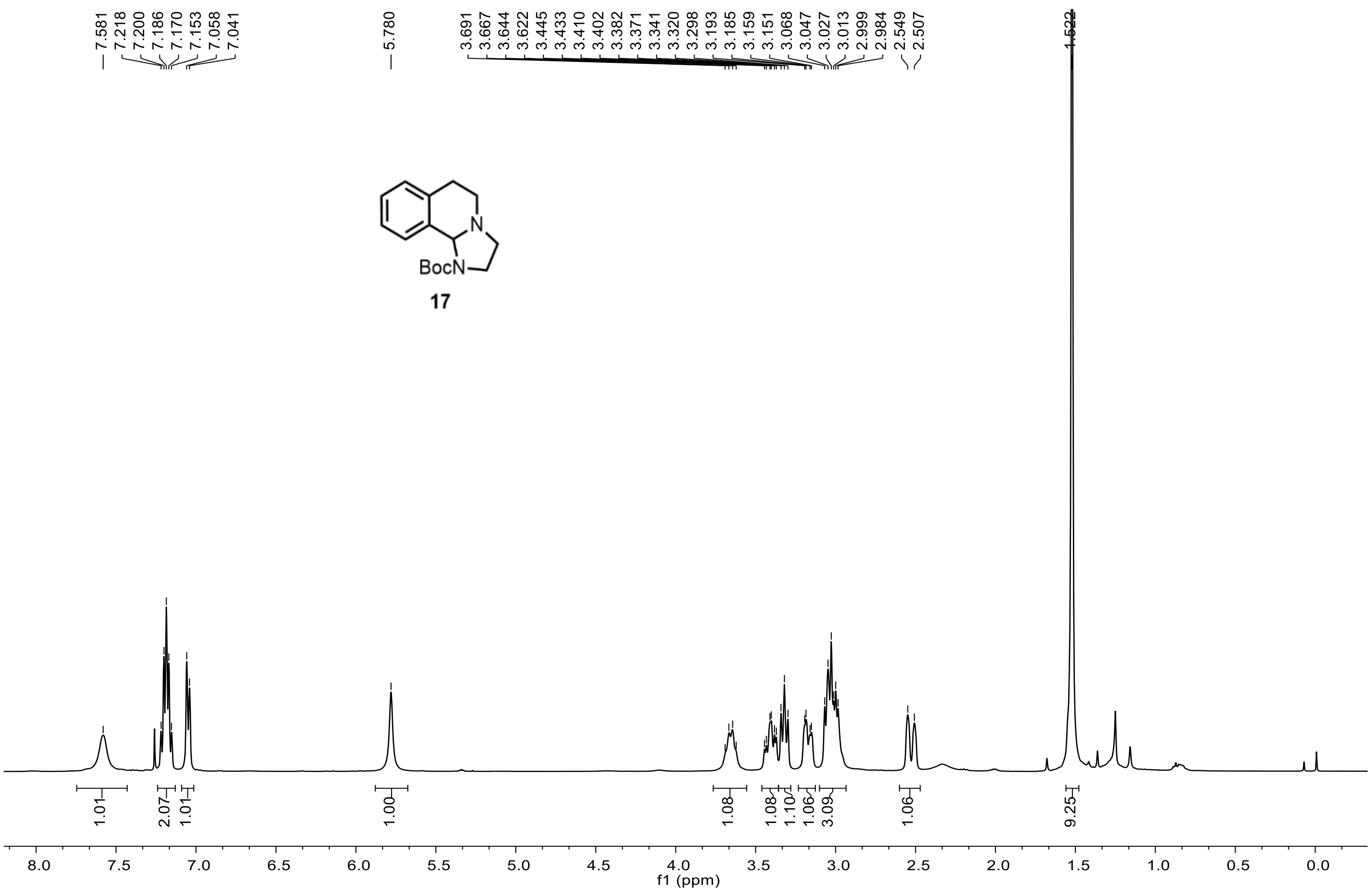

^{1}H NMR spectrum of **17** (400 MHz, CDCl$_3$)

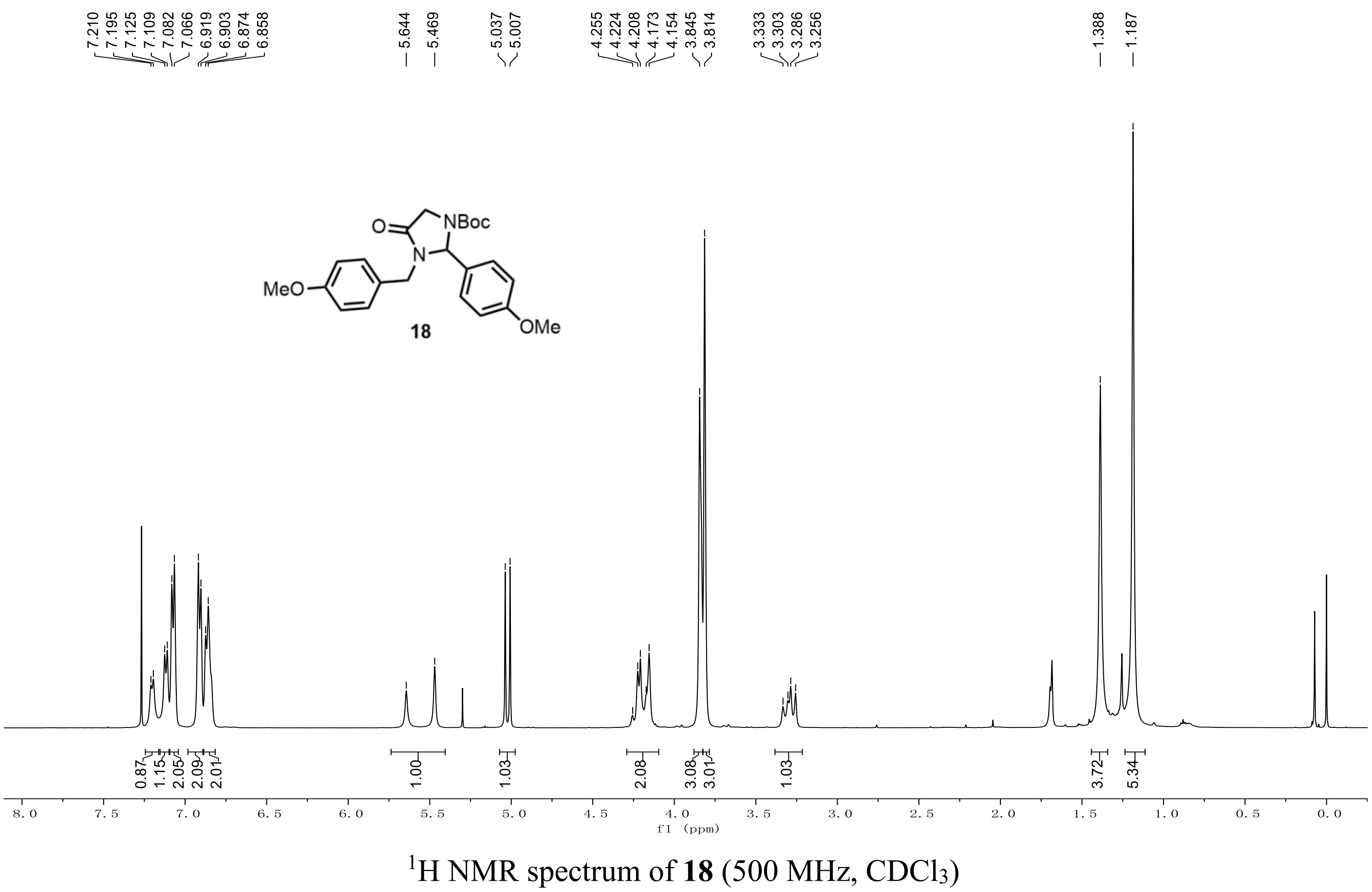

^{1}H NMR spectrum of **18** (500 MHz, CDCl$_3$)

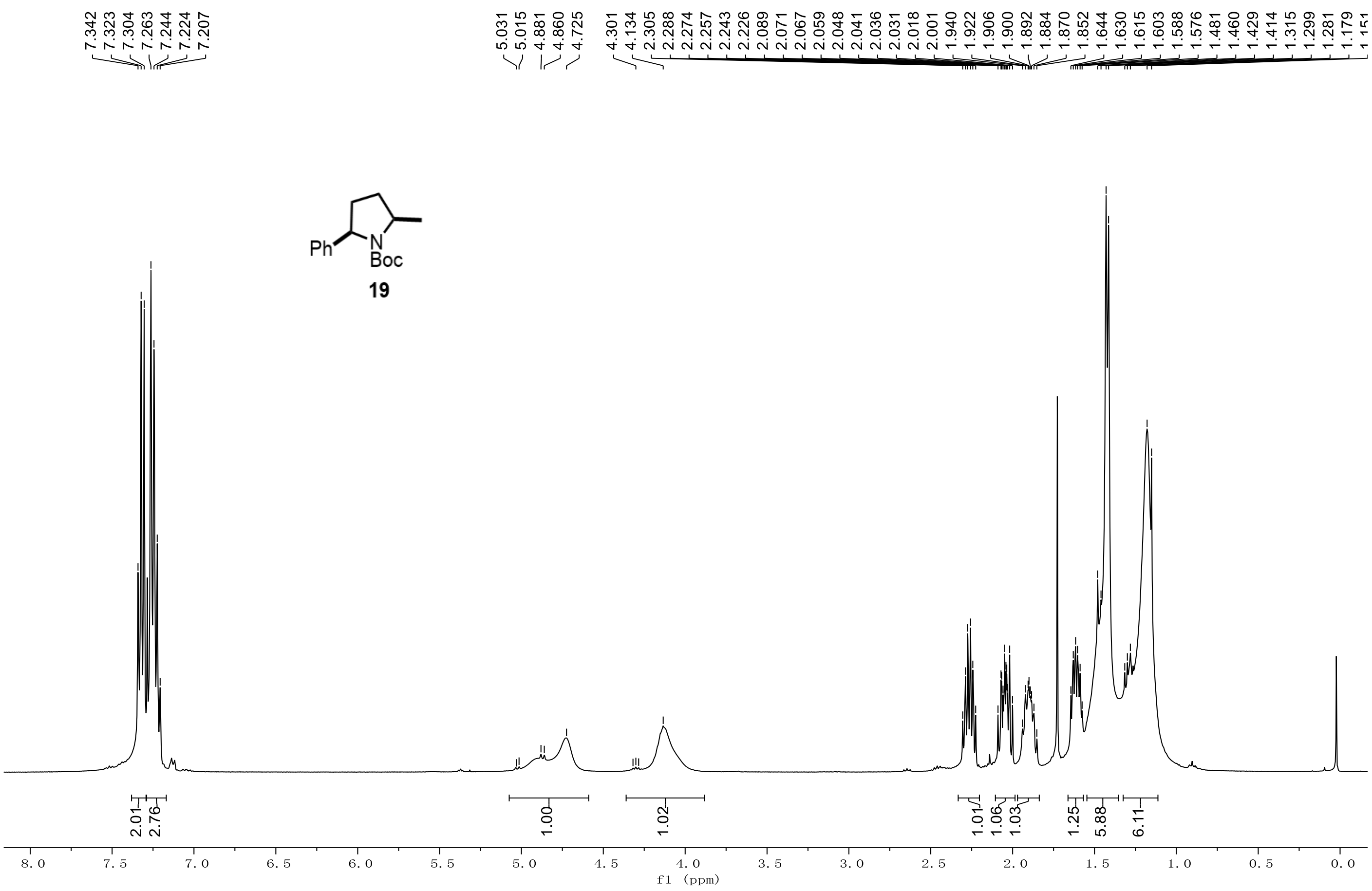

^{1}H NMR spectrum of **19** (400 MHz, $CDCl_3$)

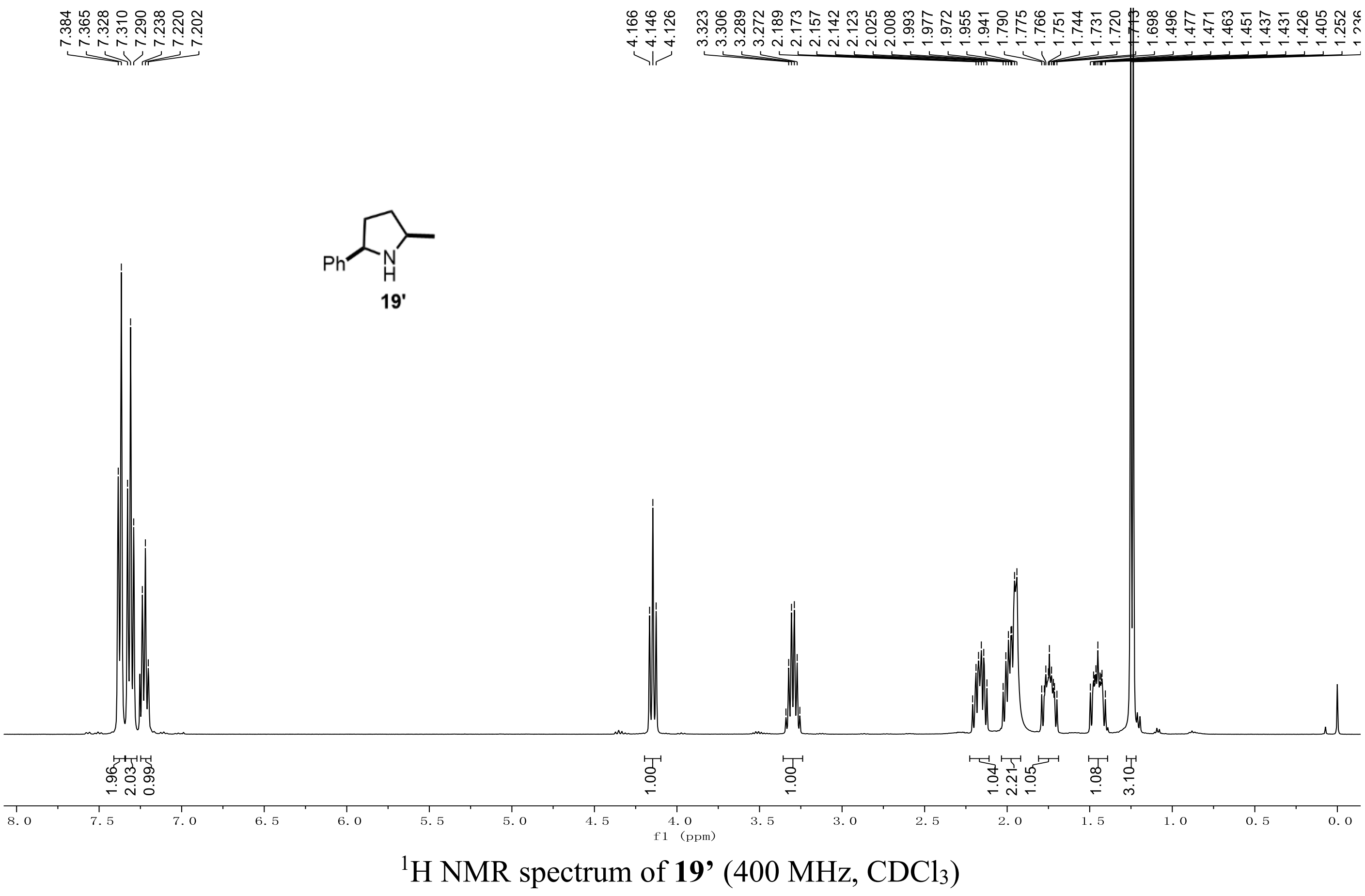

^{1}H NMR spectrum of **19’** (400 MHz, $CDCl_3$)

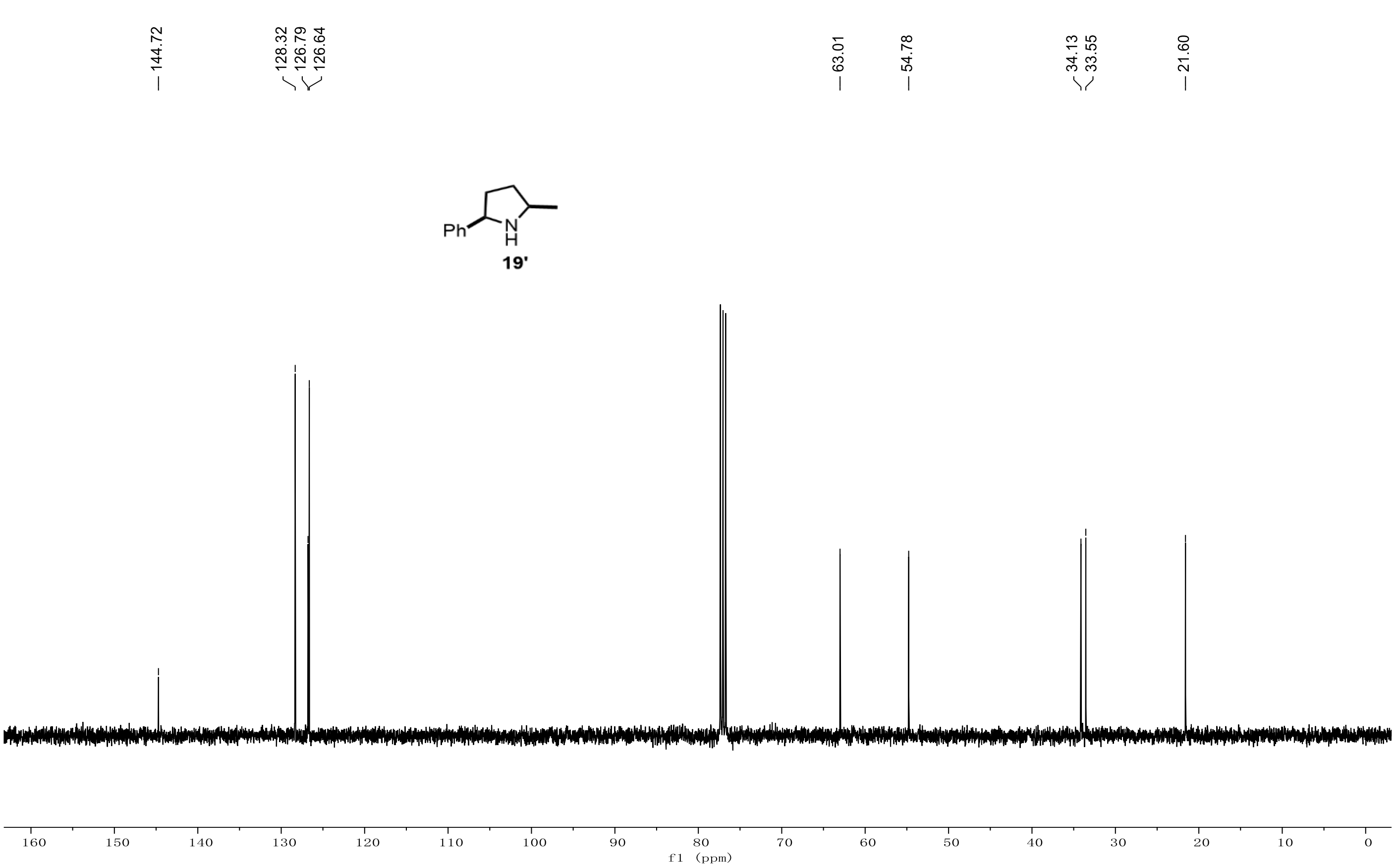

^{13}C NMR spectrum of **19'** (100 MHz, $CDCl_3$)

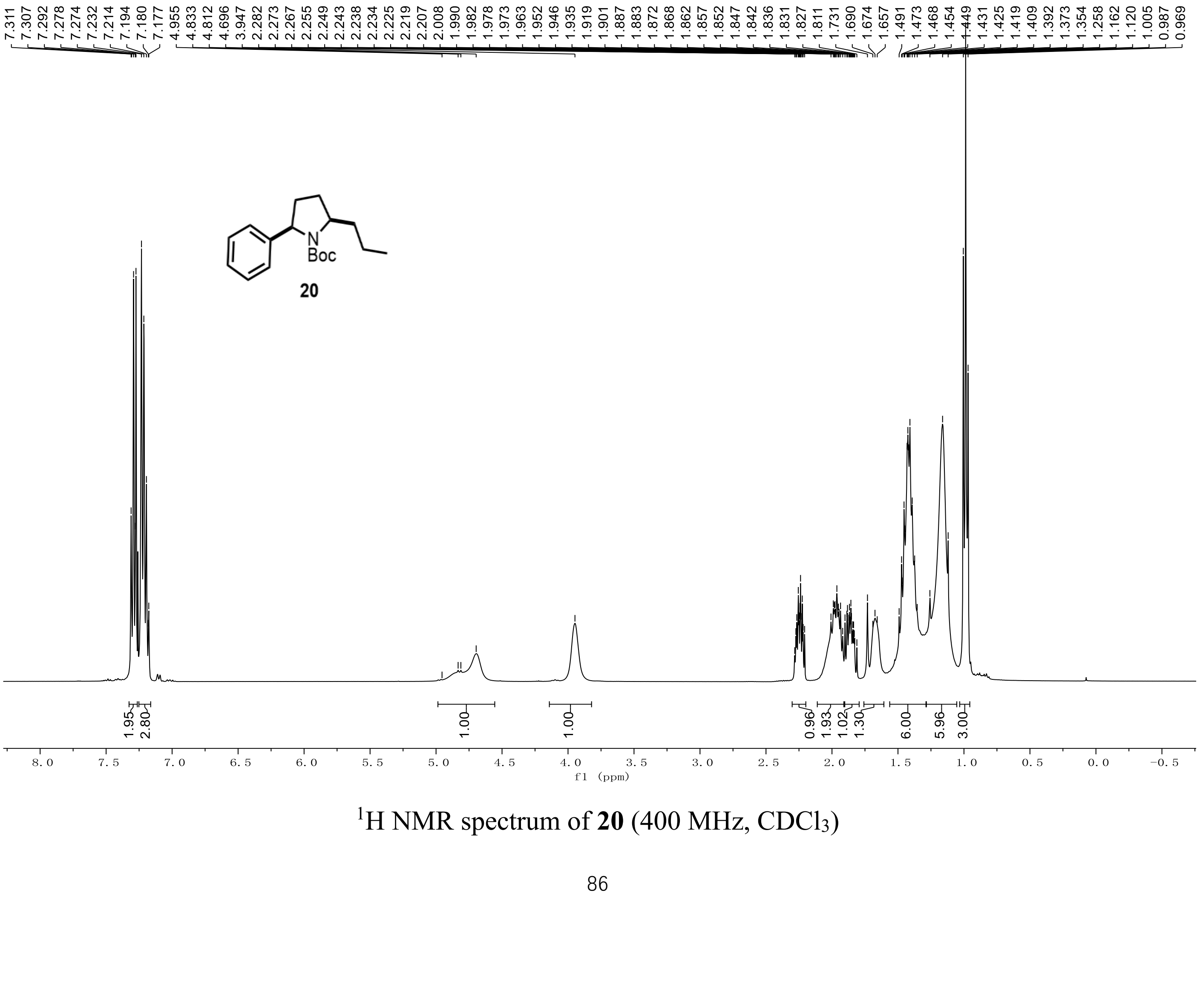

^{1}H NMR spectrum of **20** (400 MHz, $CDCl_3$)

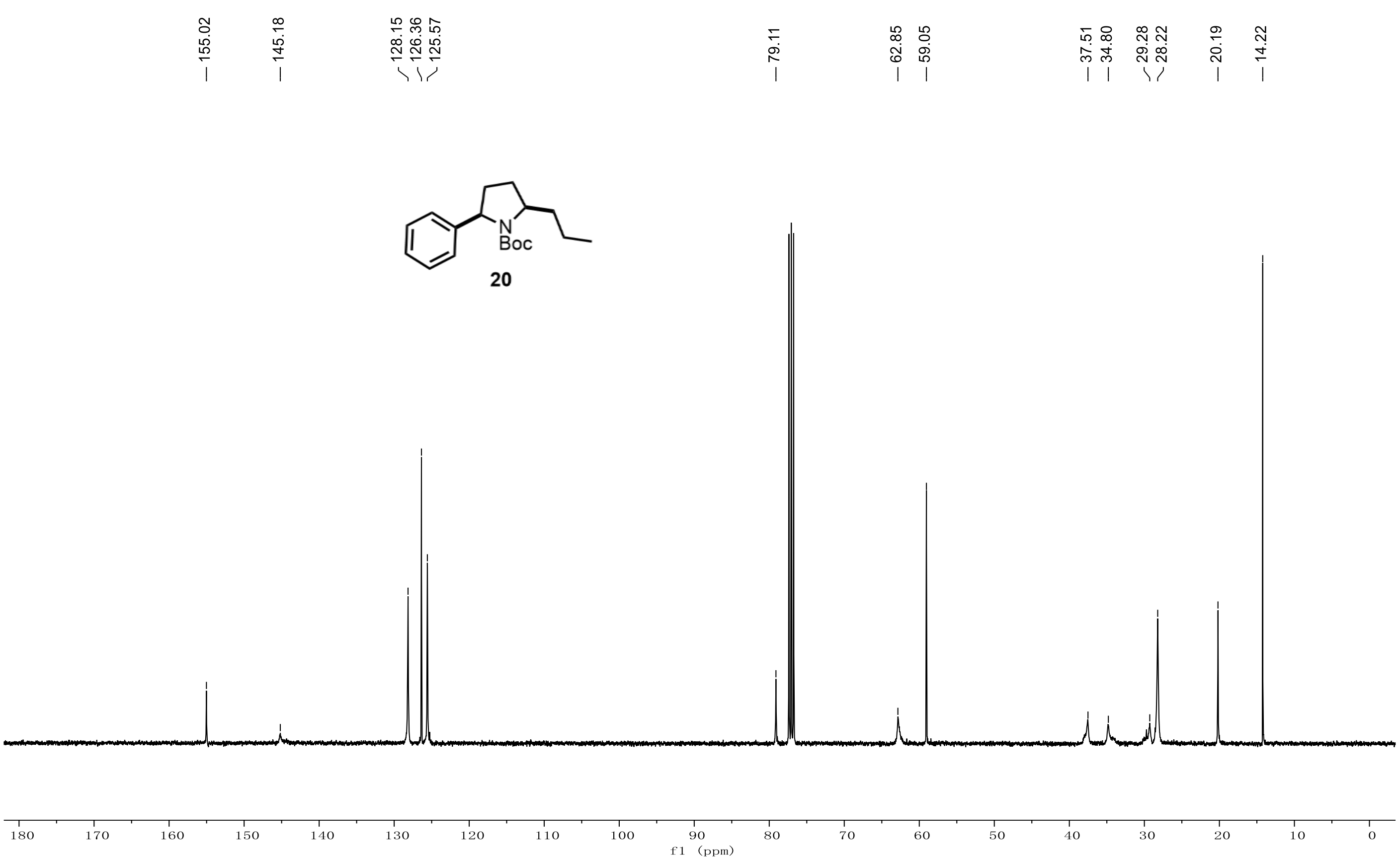

^{13}C NMR spectrum of **20** (100 MHz, $CDCl_3$)

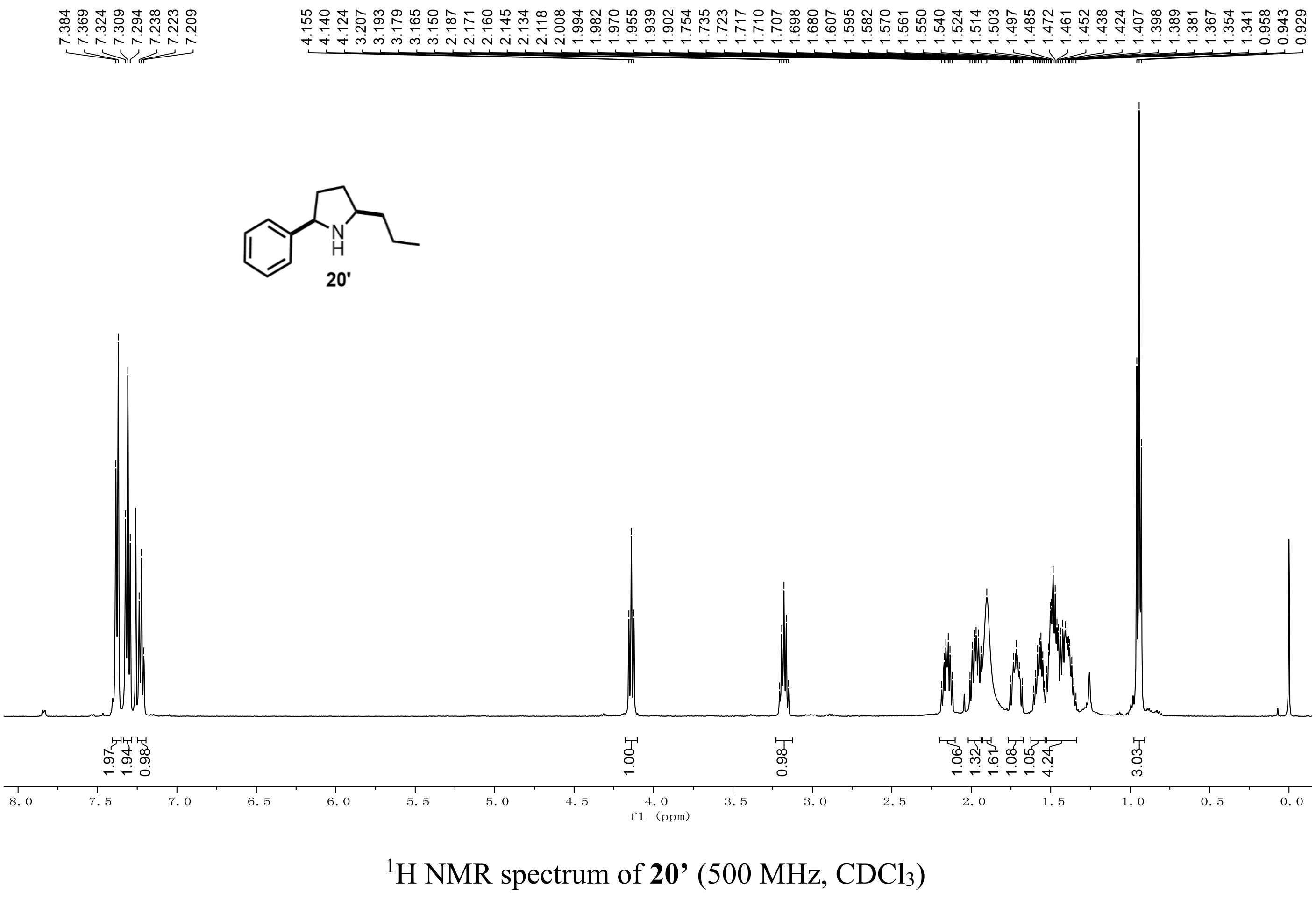

^{1}H NMR spectrum of **20’** (500 MHz, $CDCl_3$)

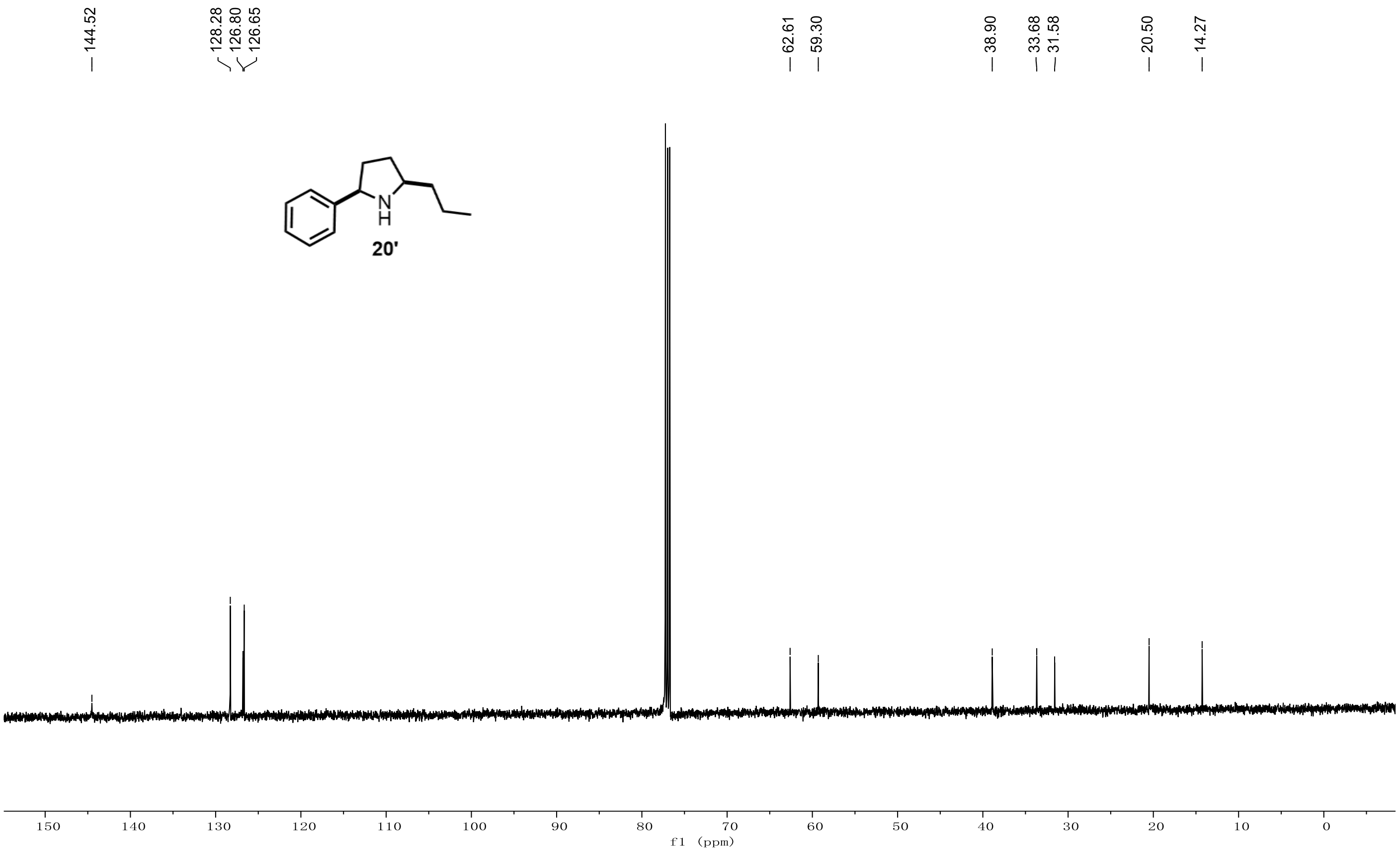

^{13}C NMR spectrum of **20'** (125 MHz, $CDCl_3$)

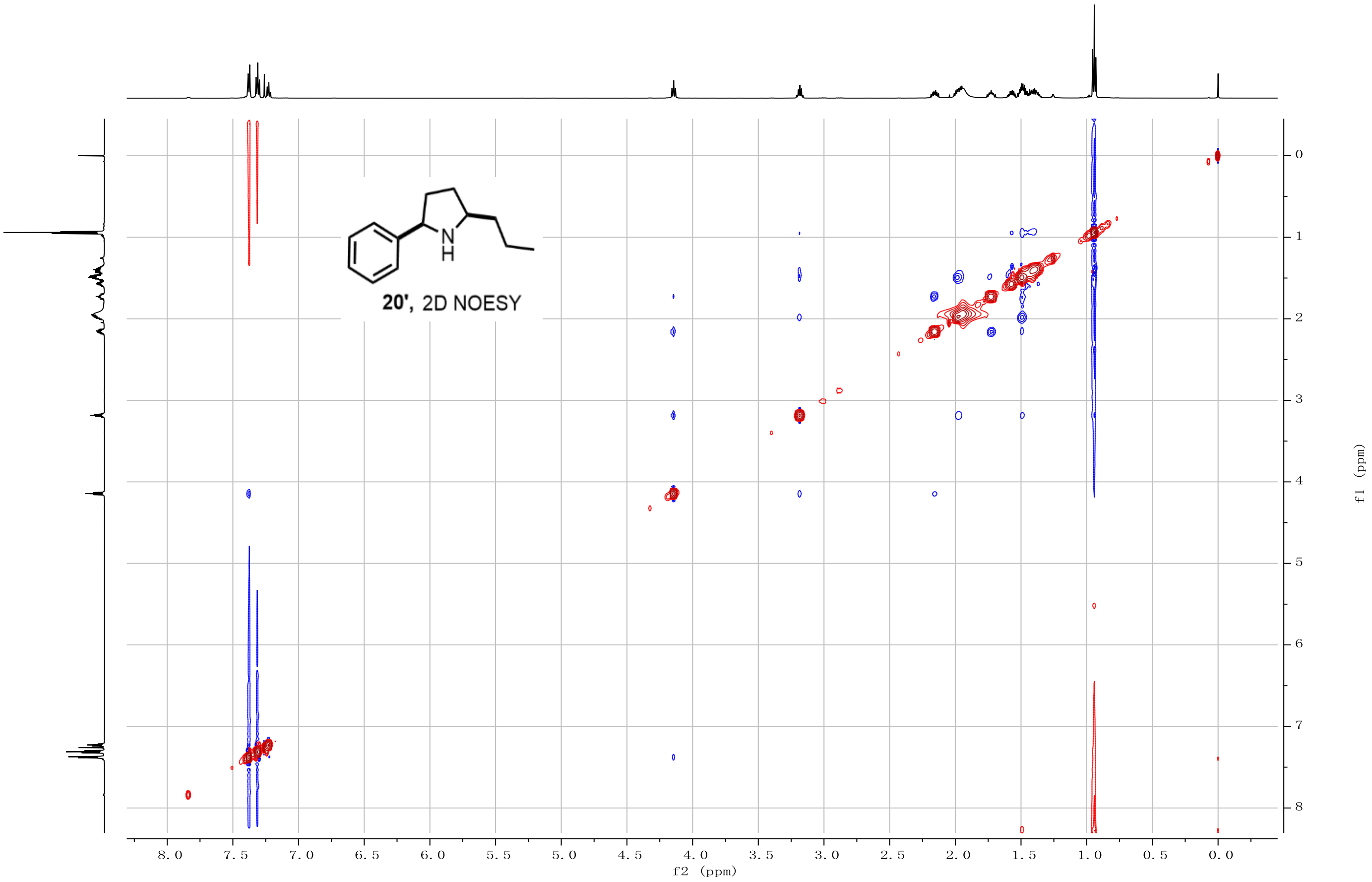

2D ^{1}H-^{1}H NOESY spectrum of **20'** (600 MHz, $CDCl_3$)

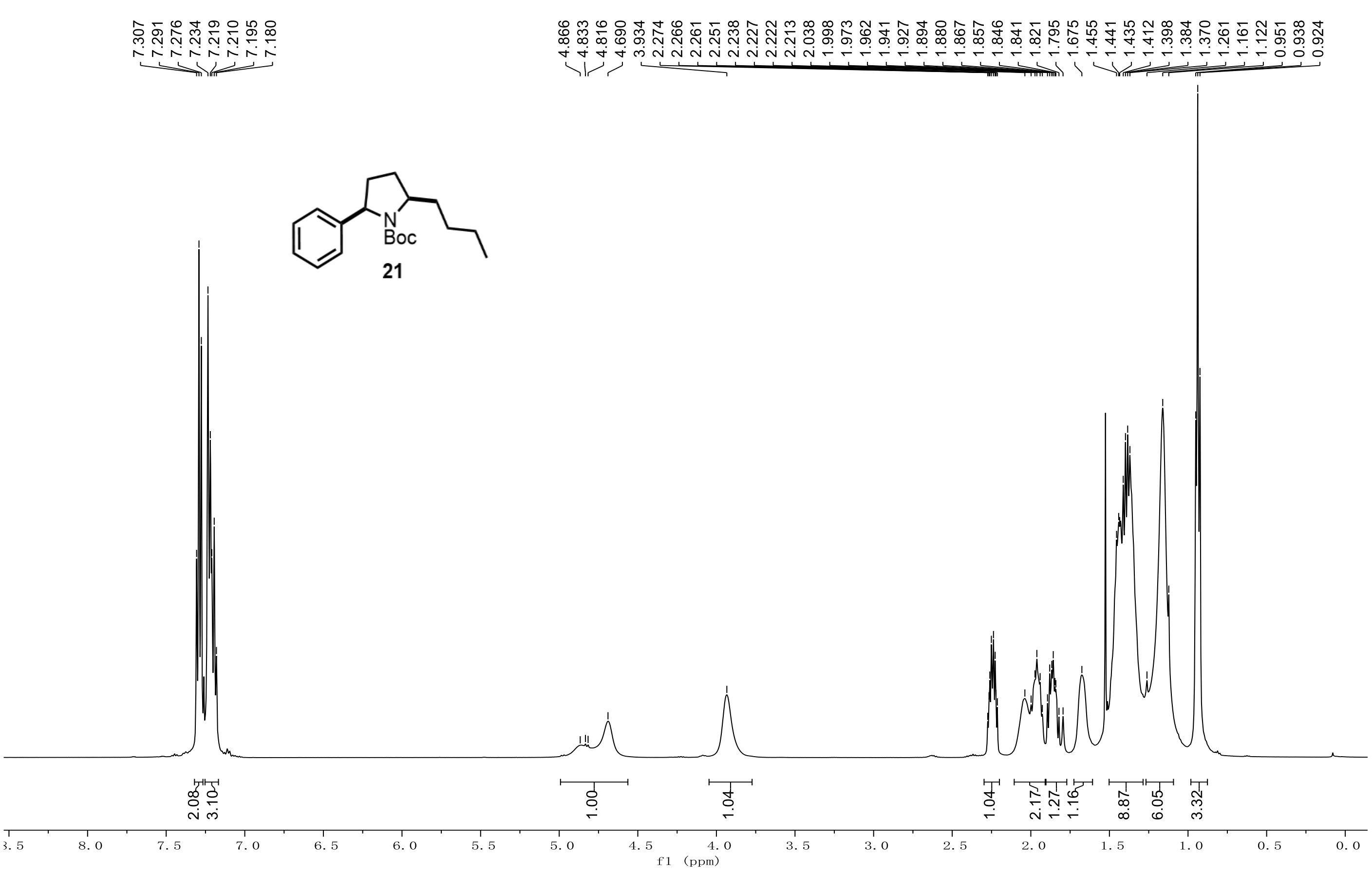

^{1}H NMR spectrum of **21** (500 MHz, $CDCl_3$)

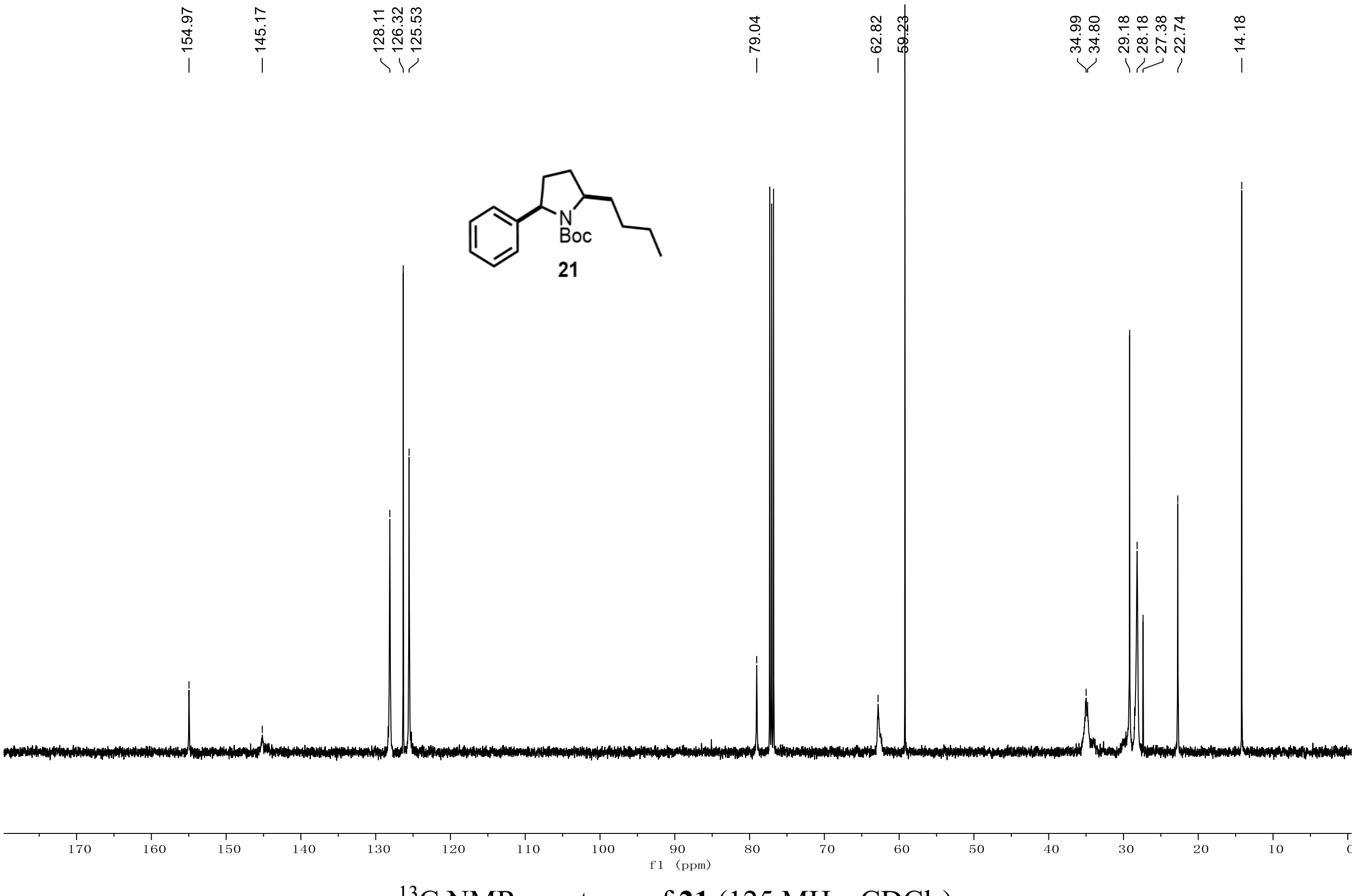

^{13}C NMR spectrum of **21** (125 MHz, $CDCl_3$)

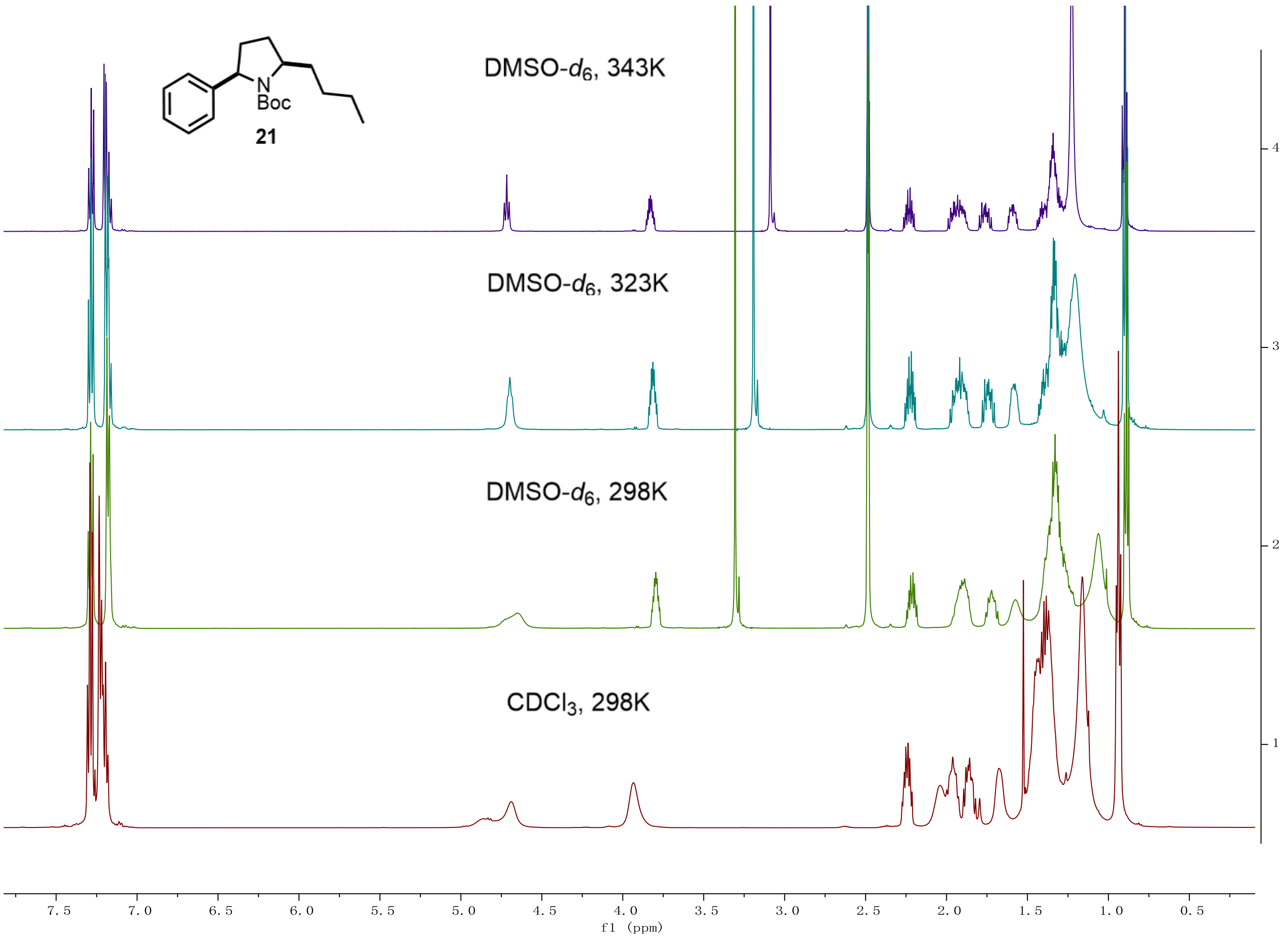

Variable-temperature ^{1}H NMR spectra of **21** (500 MHz)

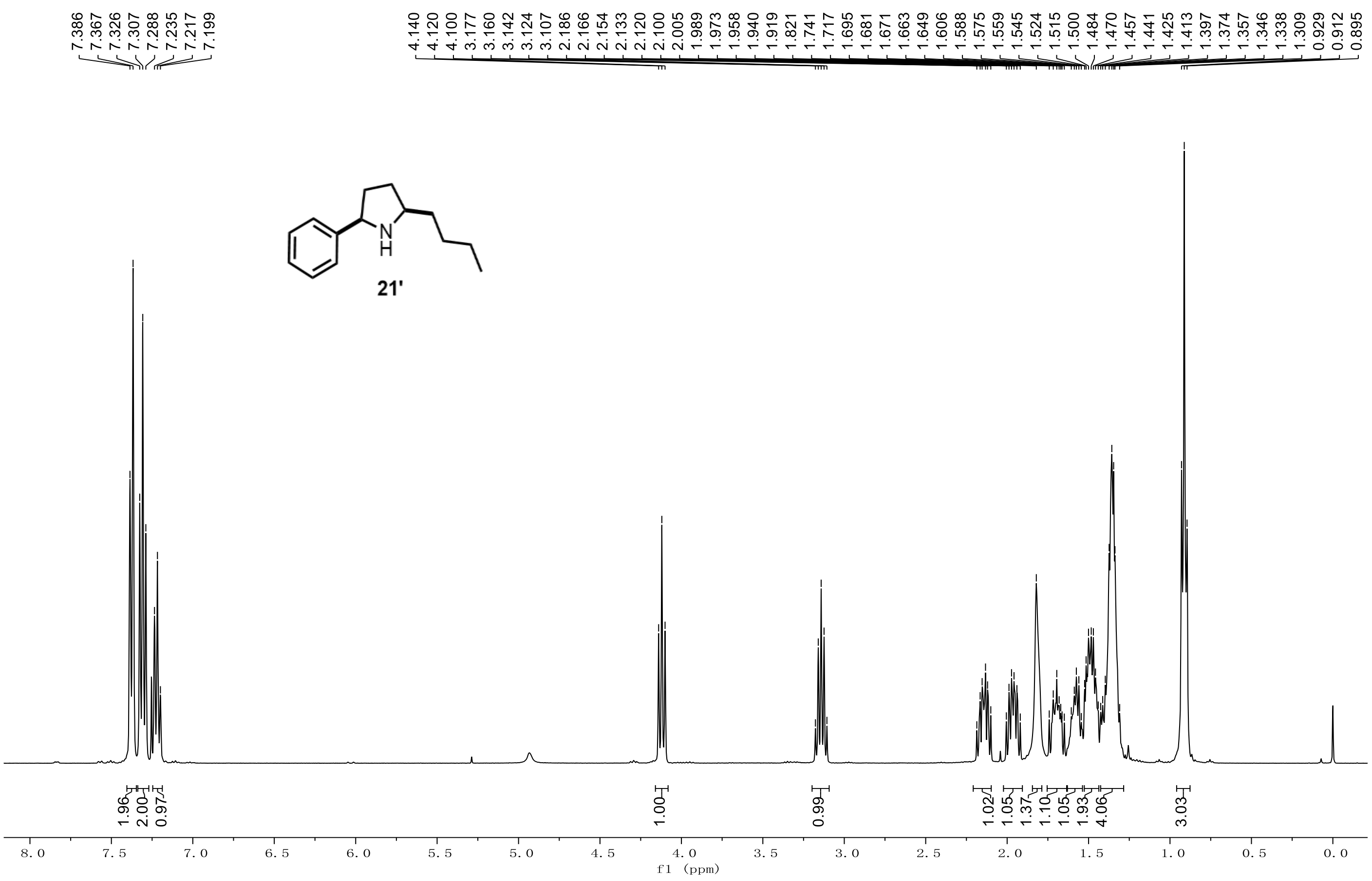

^{1}H NMR spectrum of **21’** (400 MHz, $CDCl_3$)

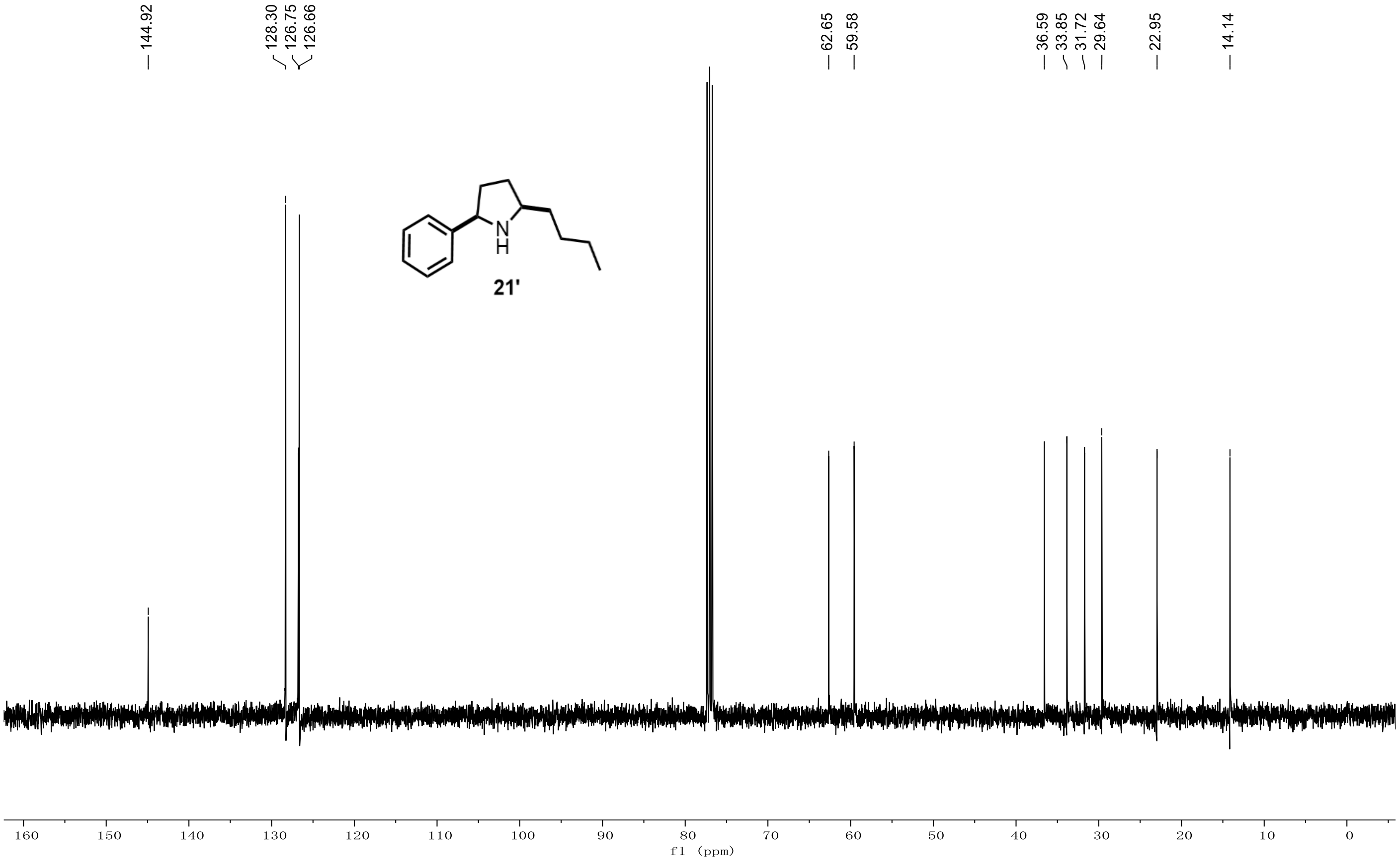

^{13}C NMR spectrum of **21’** (100 MHz, $CDCl_3$)

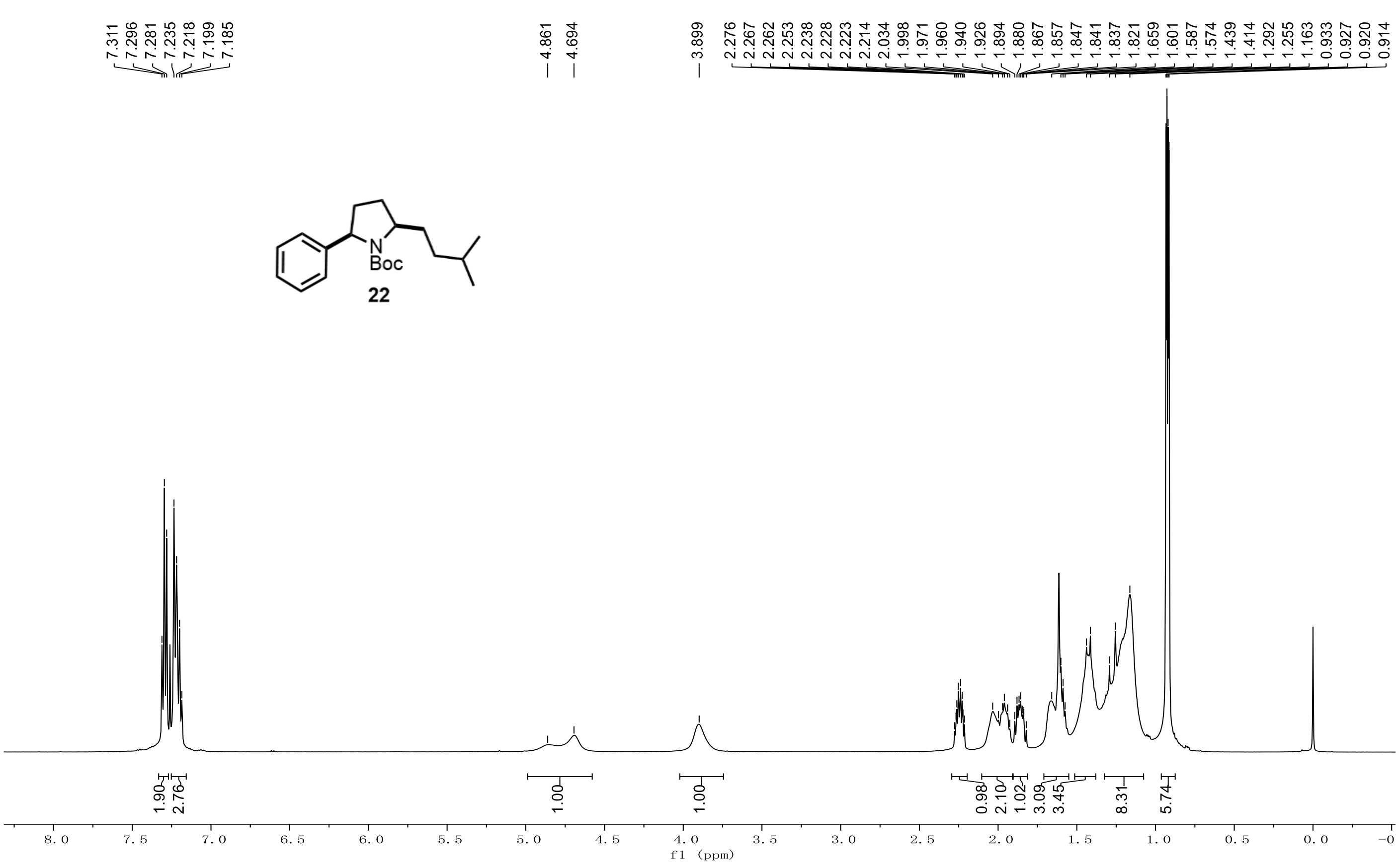

^{1}H NMR spectrum of **22** (500 MHz, $CDCl_3$)

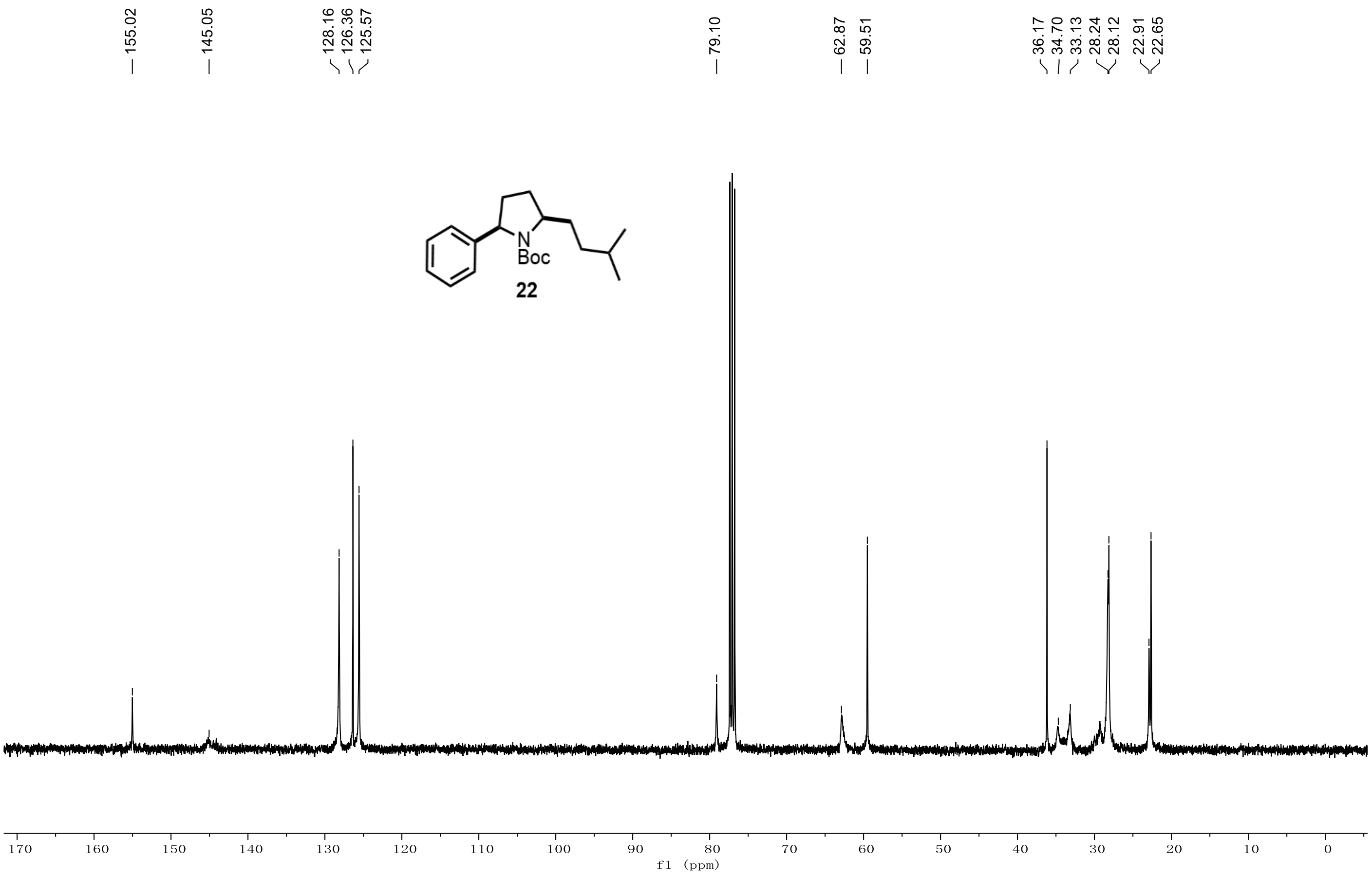

^{13}C NMR spectrum of **22** (100 MHz, $CDCl_3$)

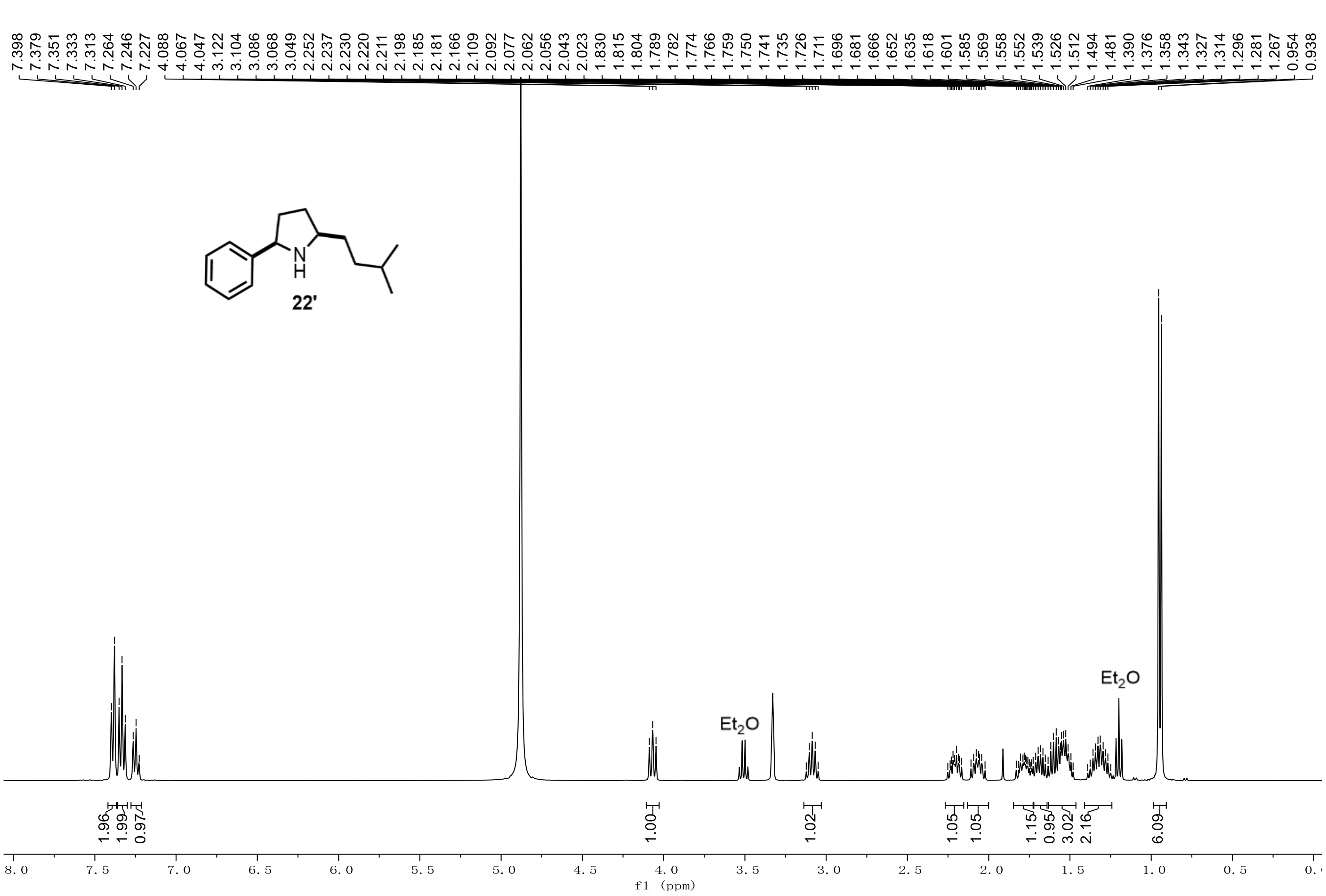

^{1}H NMR spectrum of **22’** (400 MHz, MeOD)

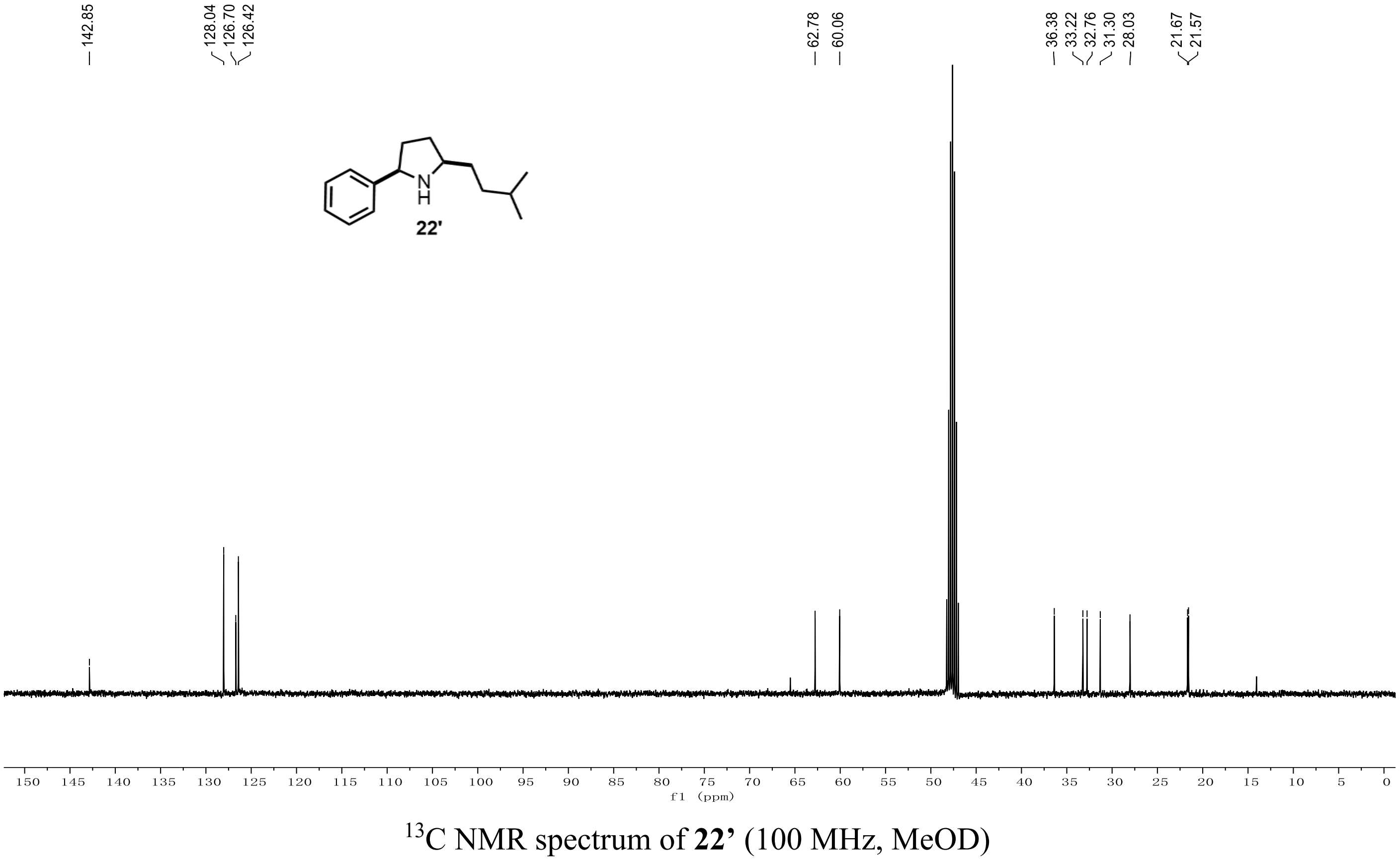

^{13}C NMR spectrum of **22’** (100 MHz, MeOD)

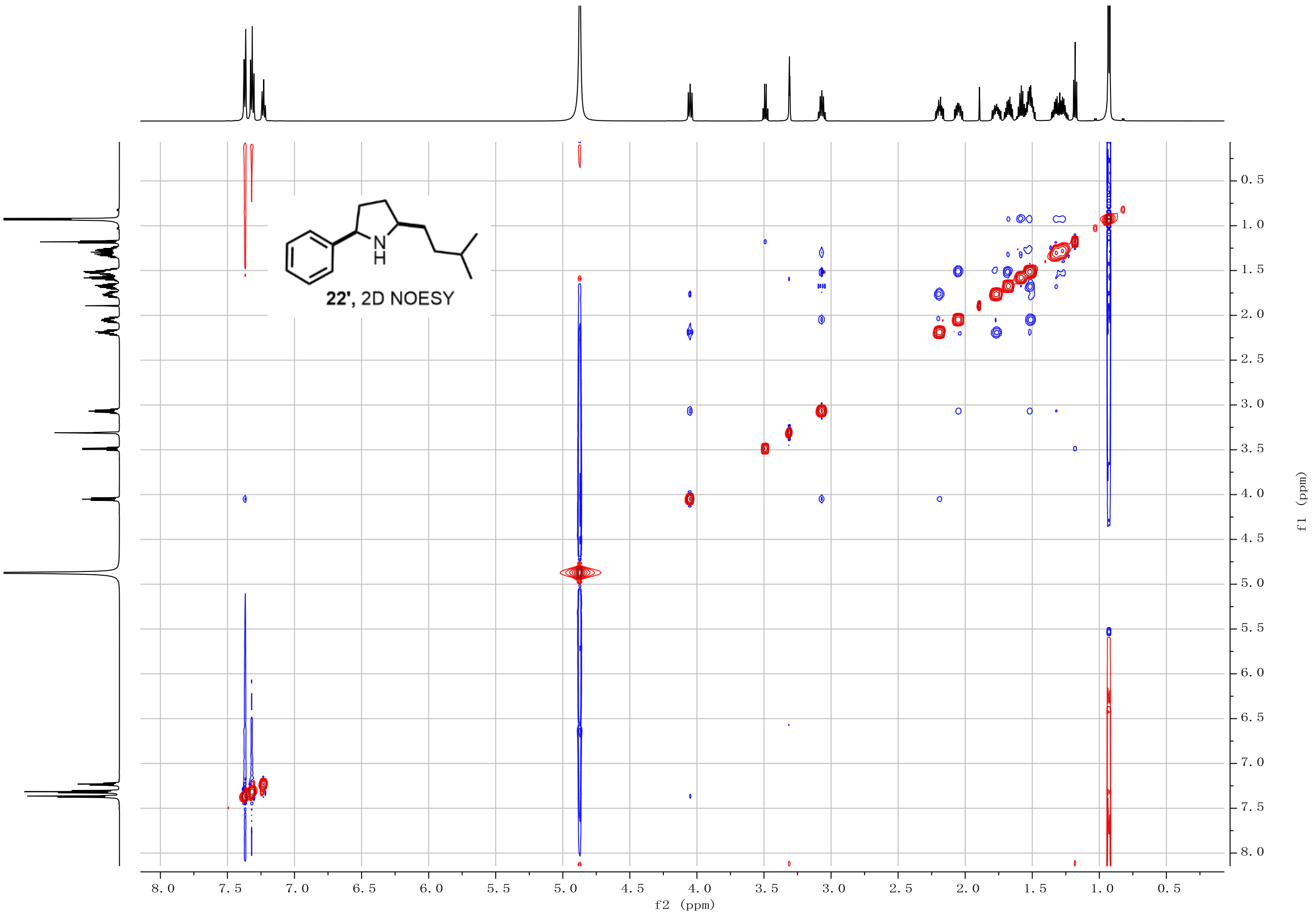

2D ^{1}H-^{1}H NOESY spectrum of **22’** (600 MHz, MeOD)

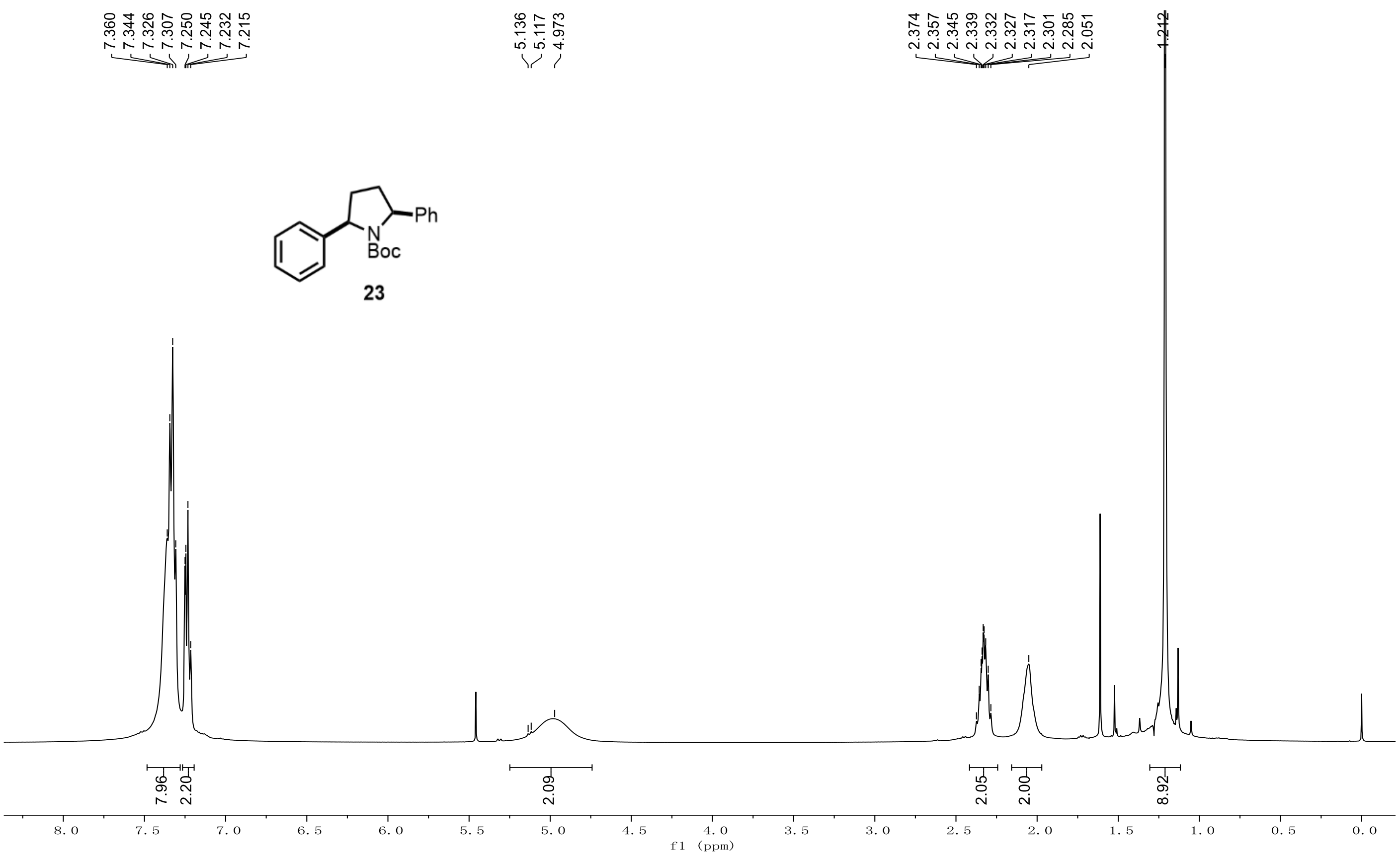

^{1}H NMR spectrum of **23** (400 MHz, $CDCl_3$)

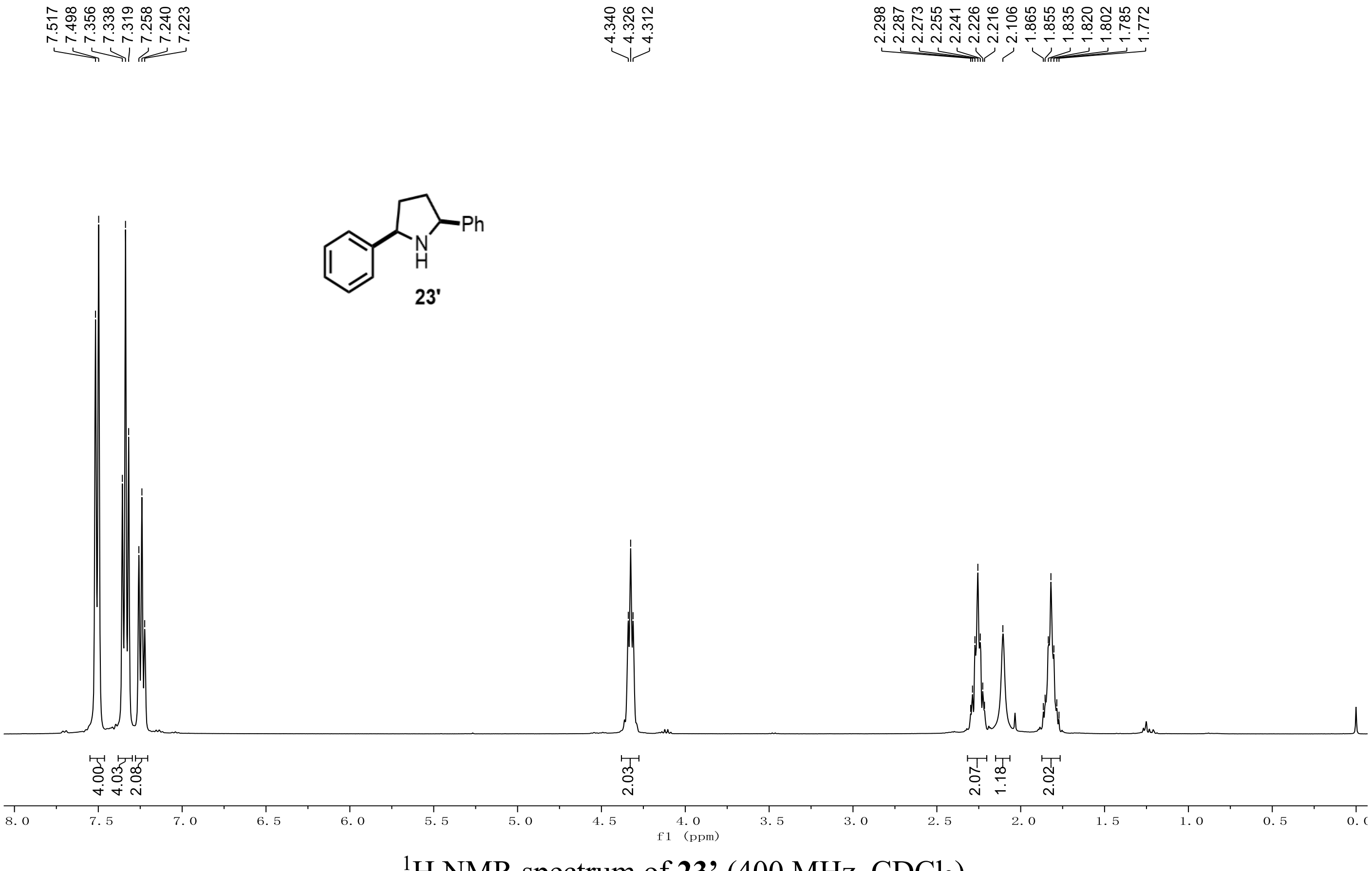

^{1}H NMR spectrum of **23’** (400 MHz, $CDCl_3$)

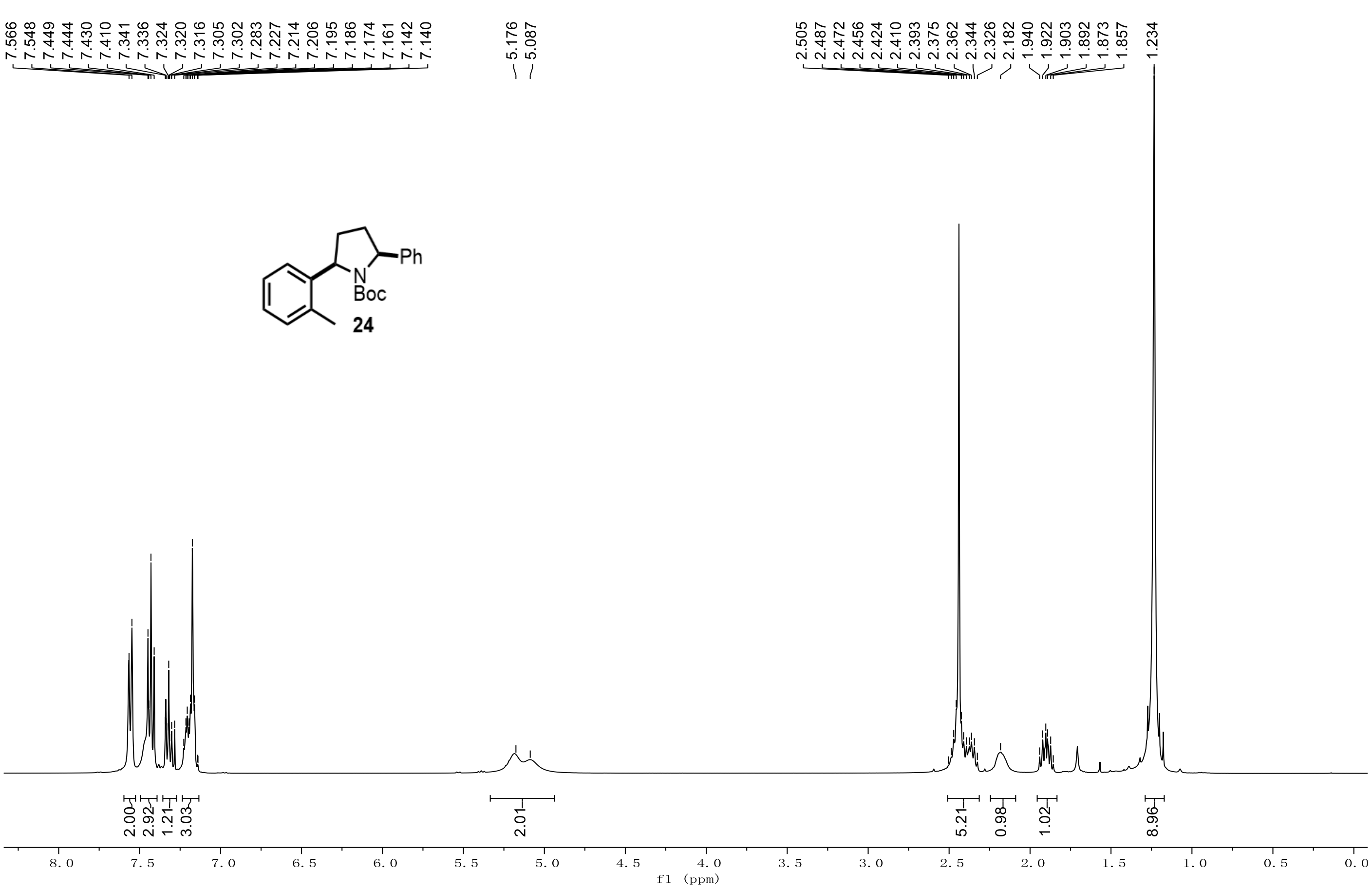

^{1}H NMR spectrum of **24** (400 MHz, $CDCl_3$)

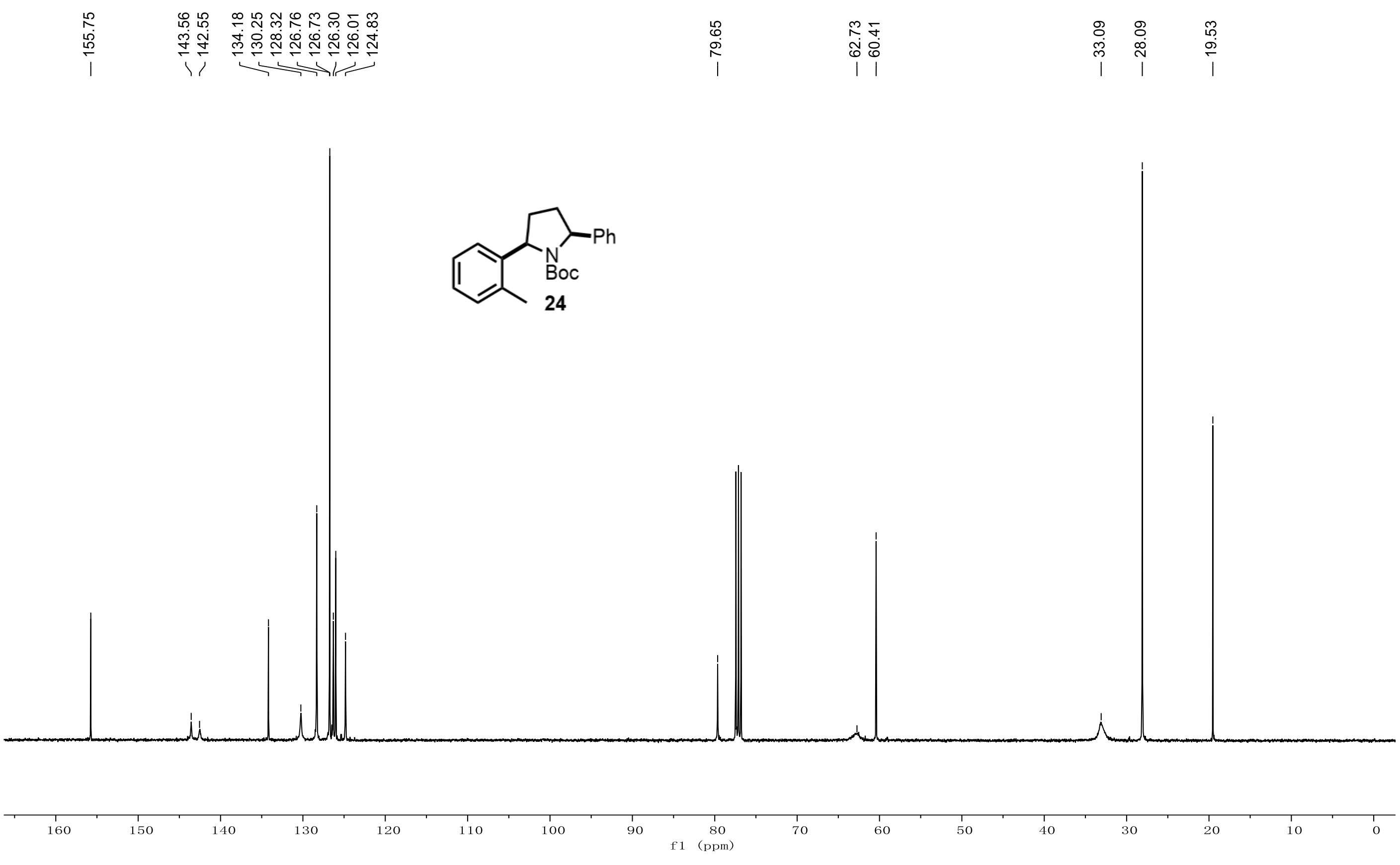

^{13}C NMR spectrum of **24** (100 MHz, $CDCl_3$)

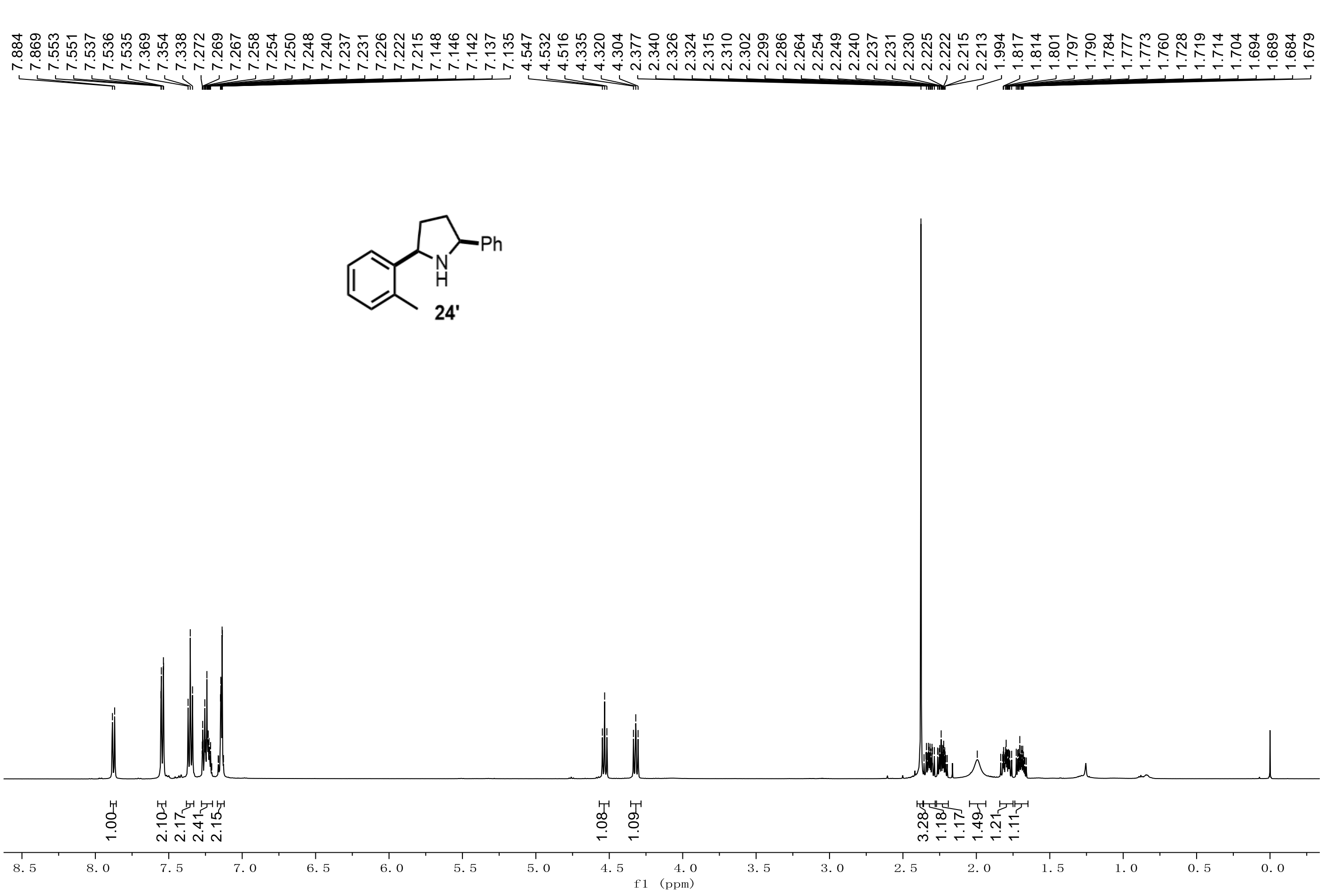

^{1}H NMR spectrum of **24’** (500 MHz, $CDCl_3$)

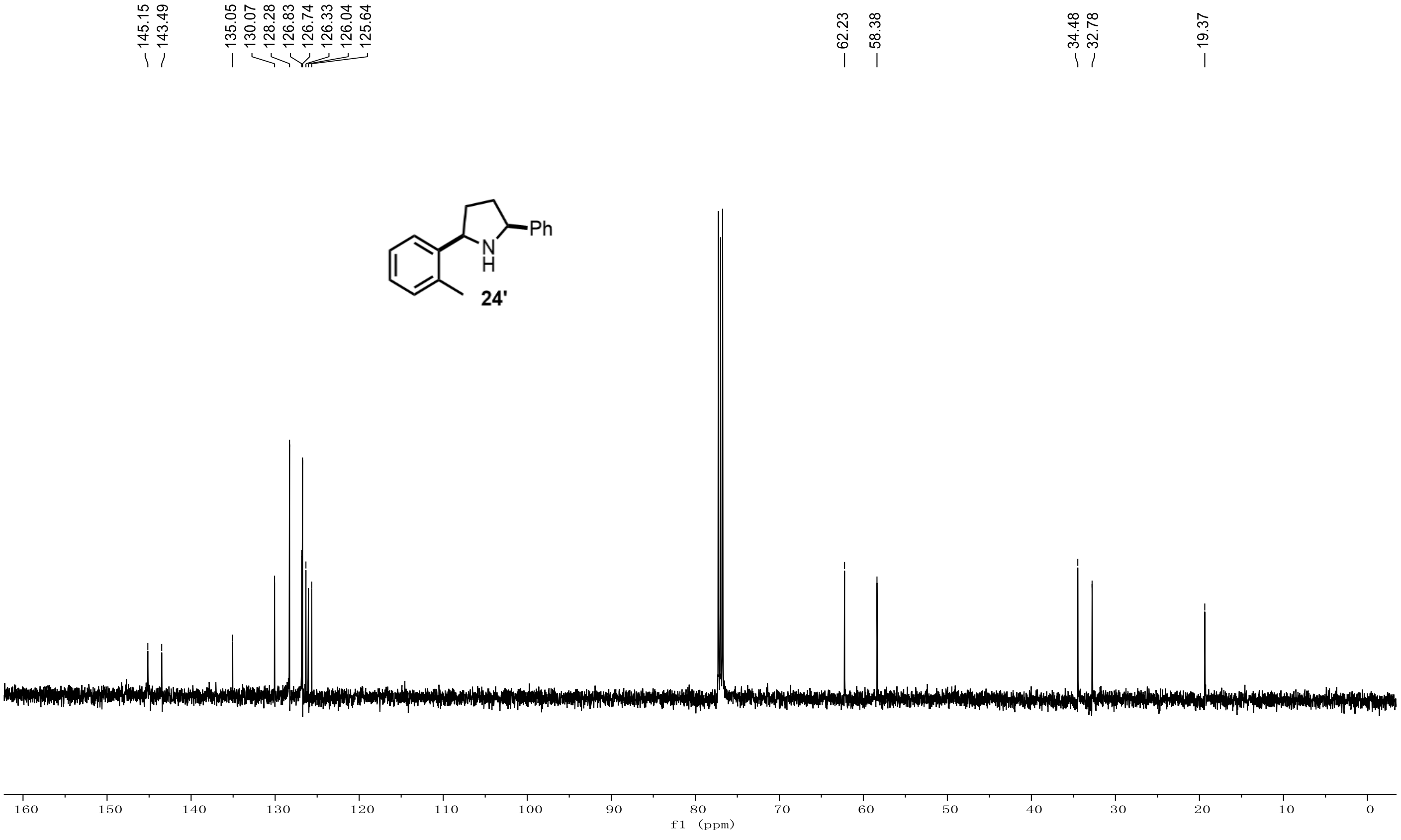

^{13}C NMR spectrum of **24’** (125 MHz, $CDCl_3$)

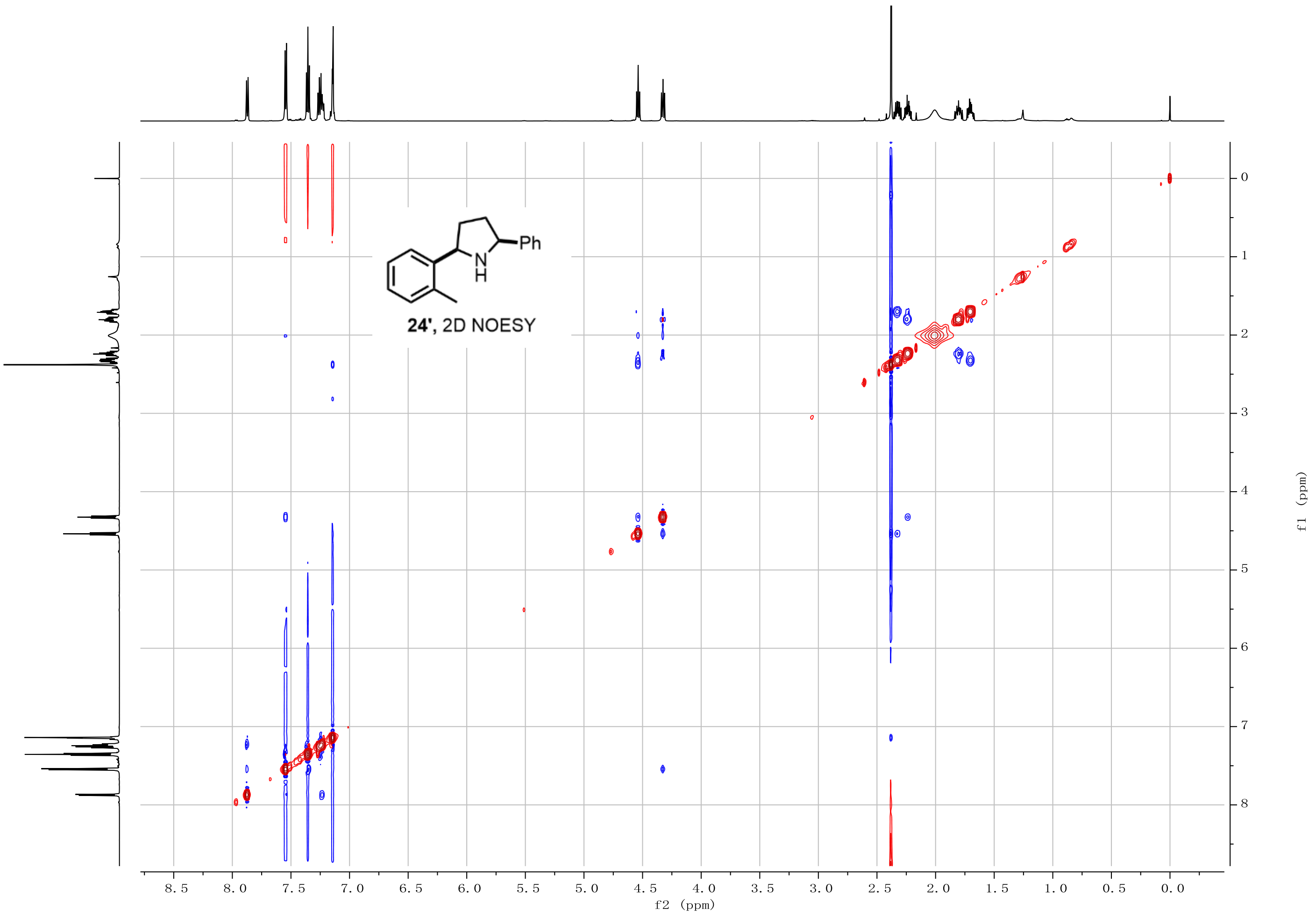

2D ^{1}H-^{1}H NOESY spectrum of **24’** (600 MHz, $CDCl_3$)

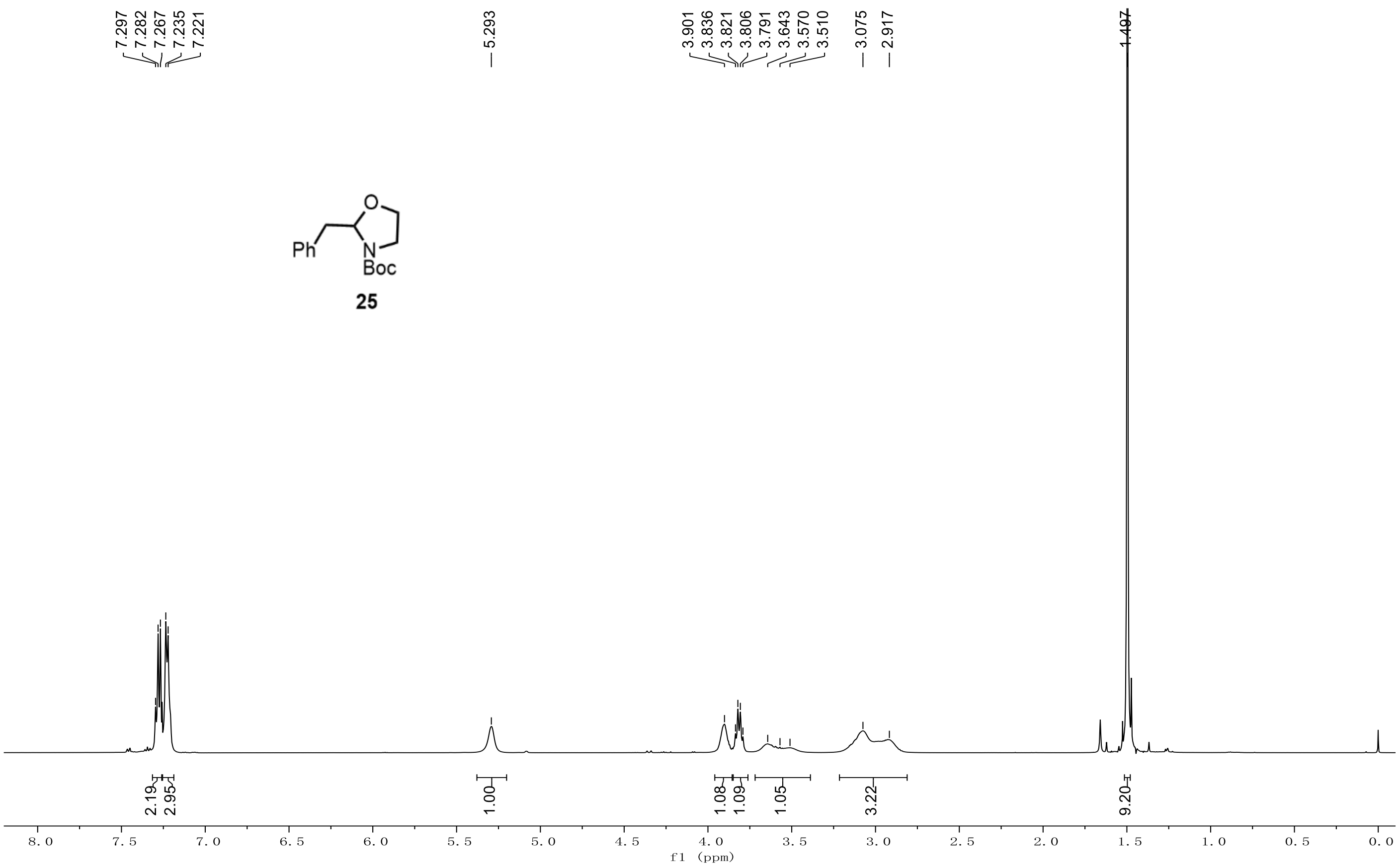

^{1}H NMR spectrum of **25** (500 MHz, $CDCl_3$)

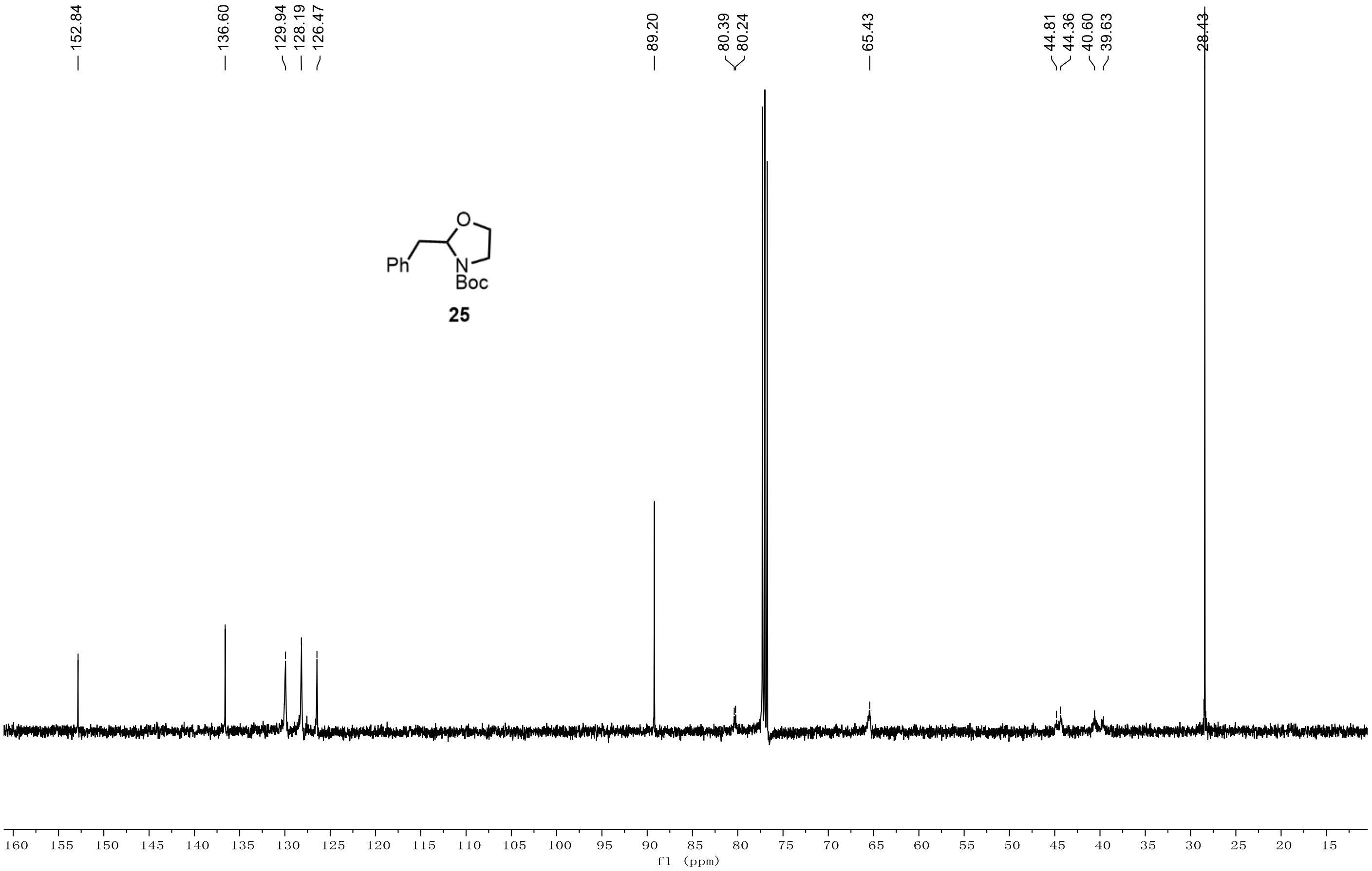

^{13}C NMR spectrum of **25** (125 MHz, $CDCl_3$)

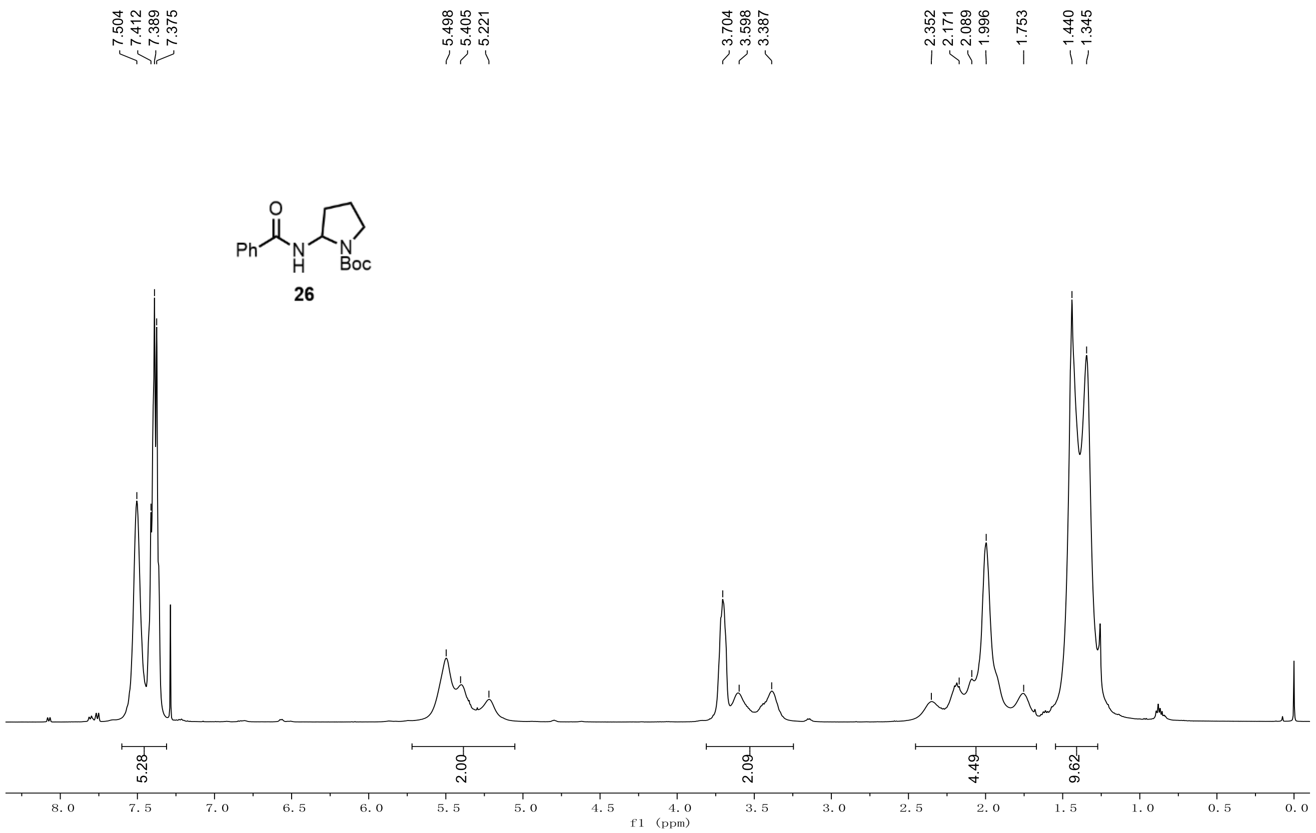

^{1}H NMR spectrum of **26** (500 MHz, $CDCl_3$)

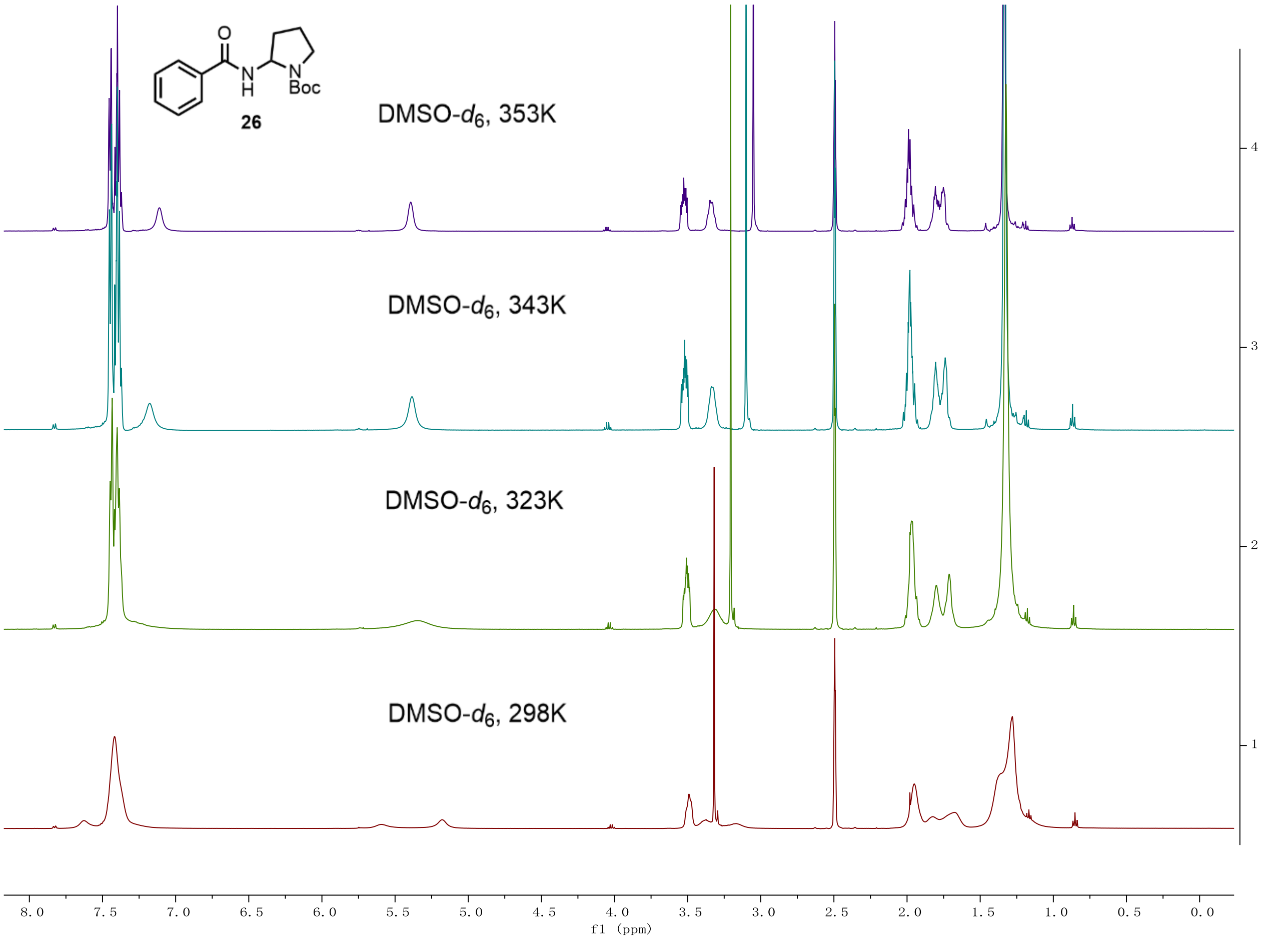

Variable-temperature ^{1}H NMR spectra of **26** (500 MHz)

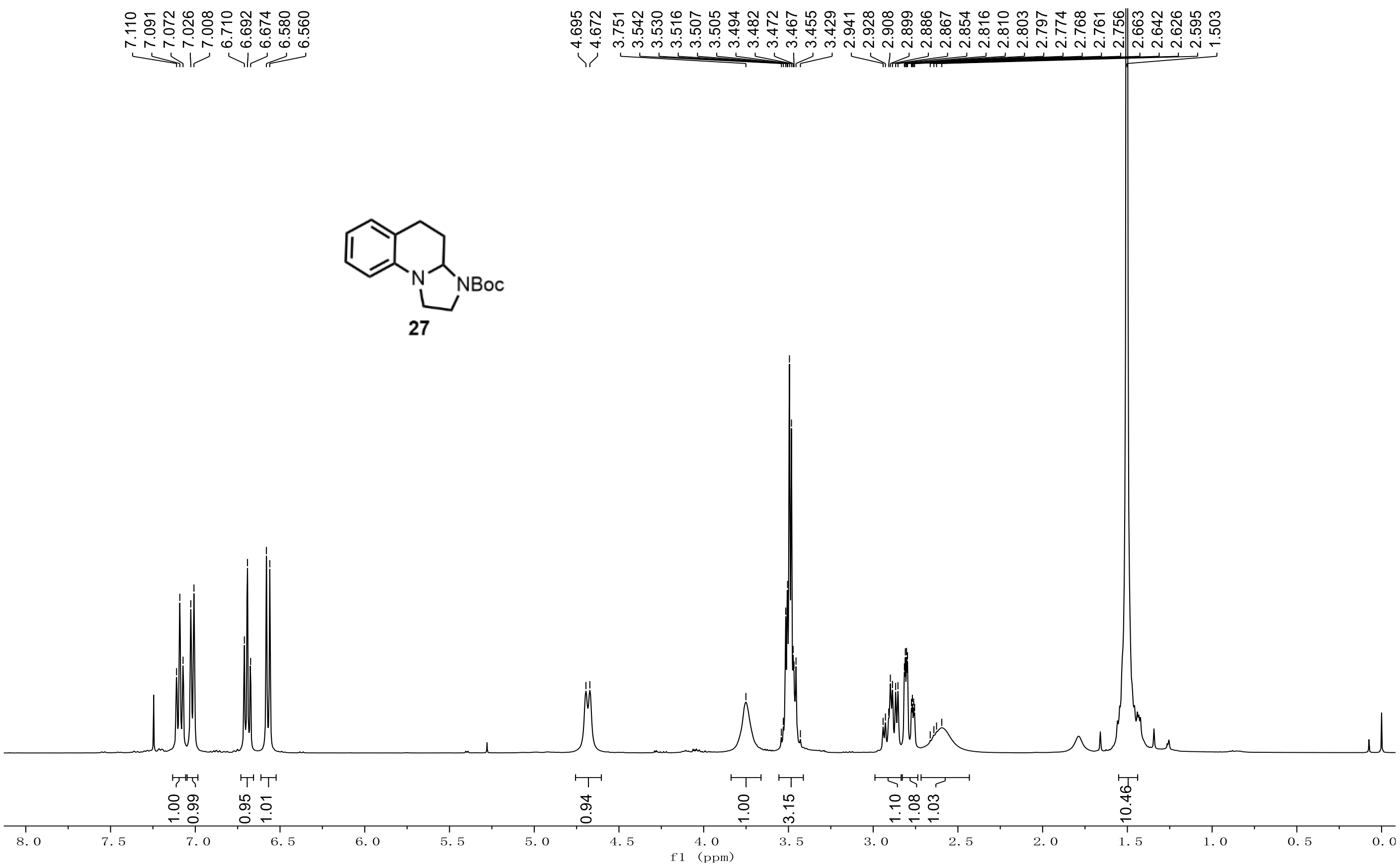

^{1}H NMR spectrum of **27** (400 MHz, $CDCl_3$)

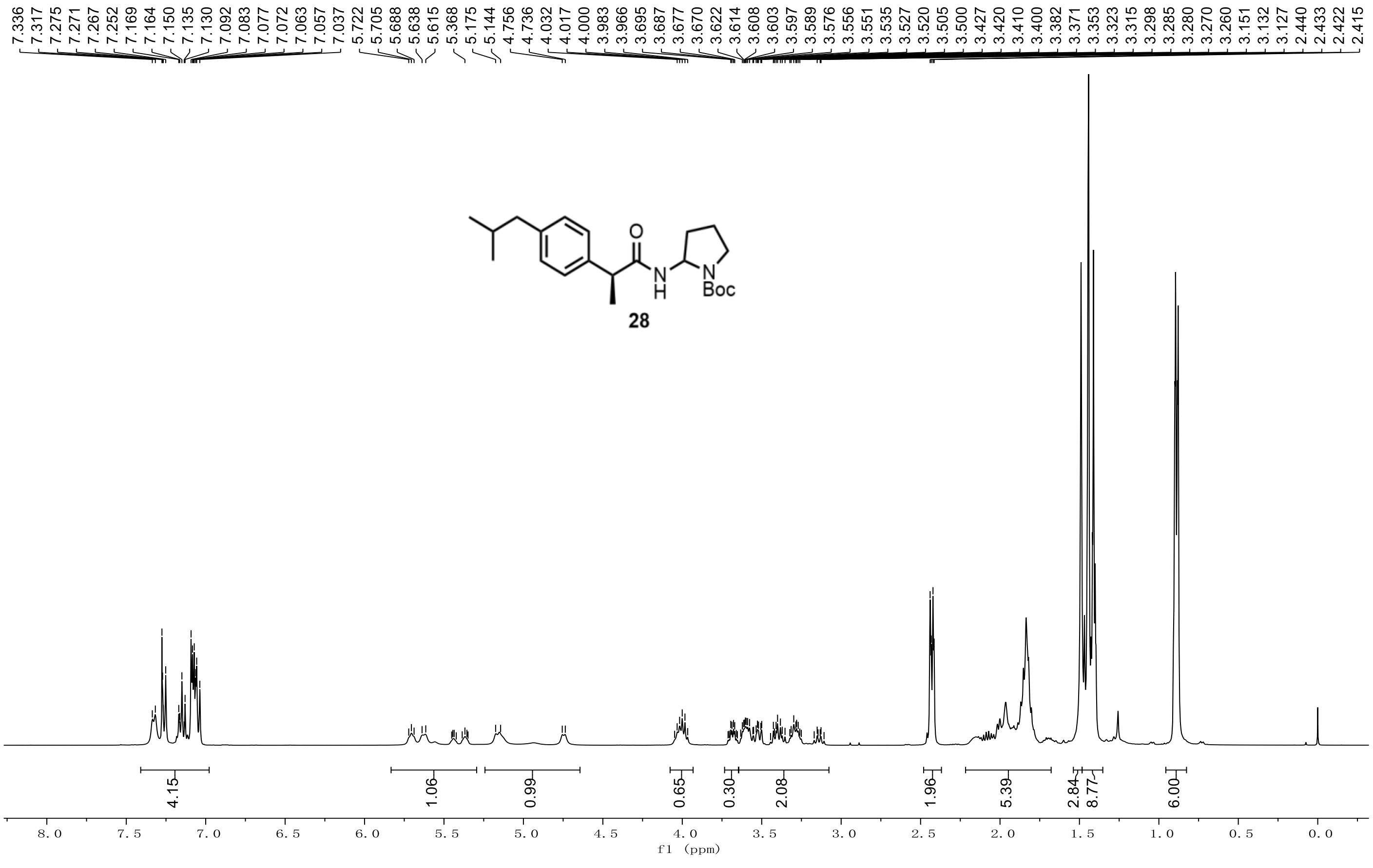

^{1}H NMR spectrum of **28** (400 MHz, $CDCl_3$)

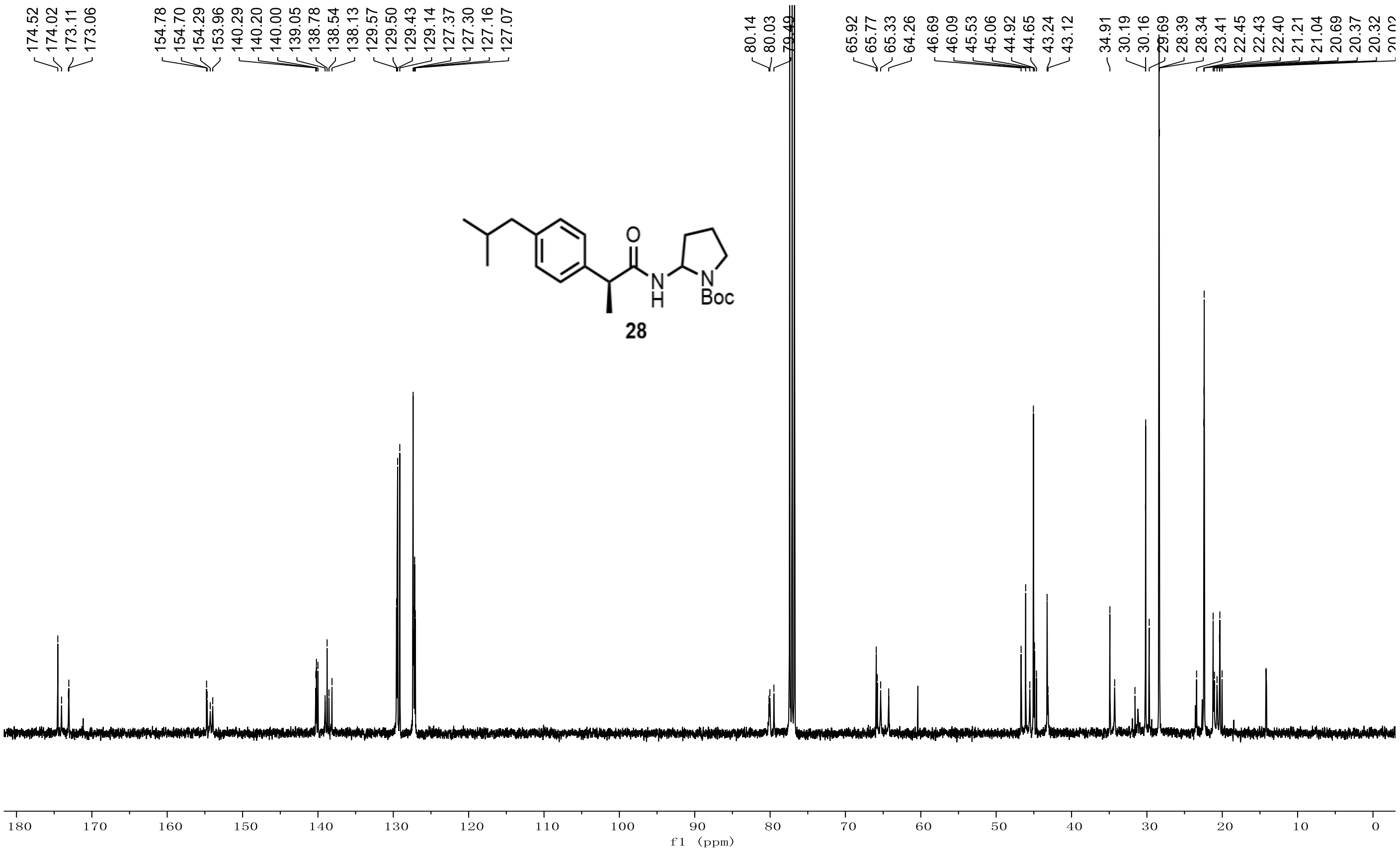

^{13}C NMR spectrum of **28** (100 MHz, $CDCl_3$)

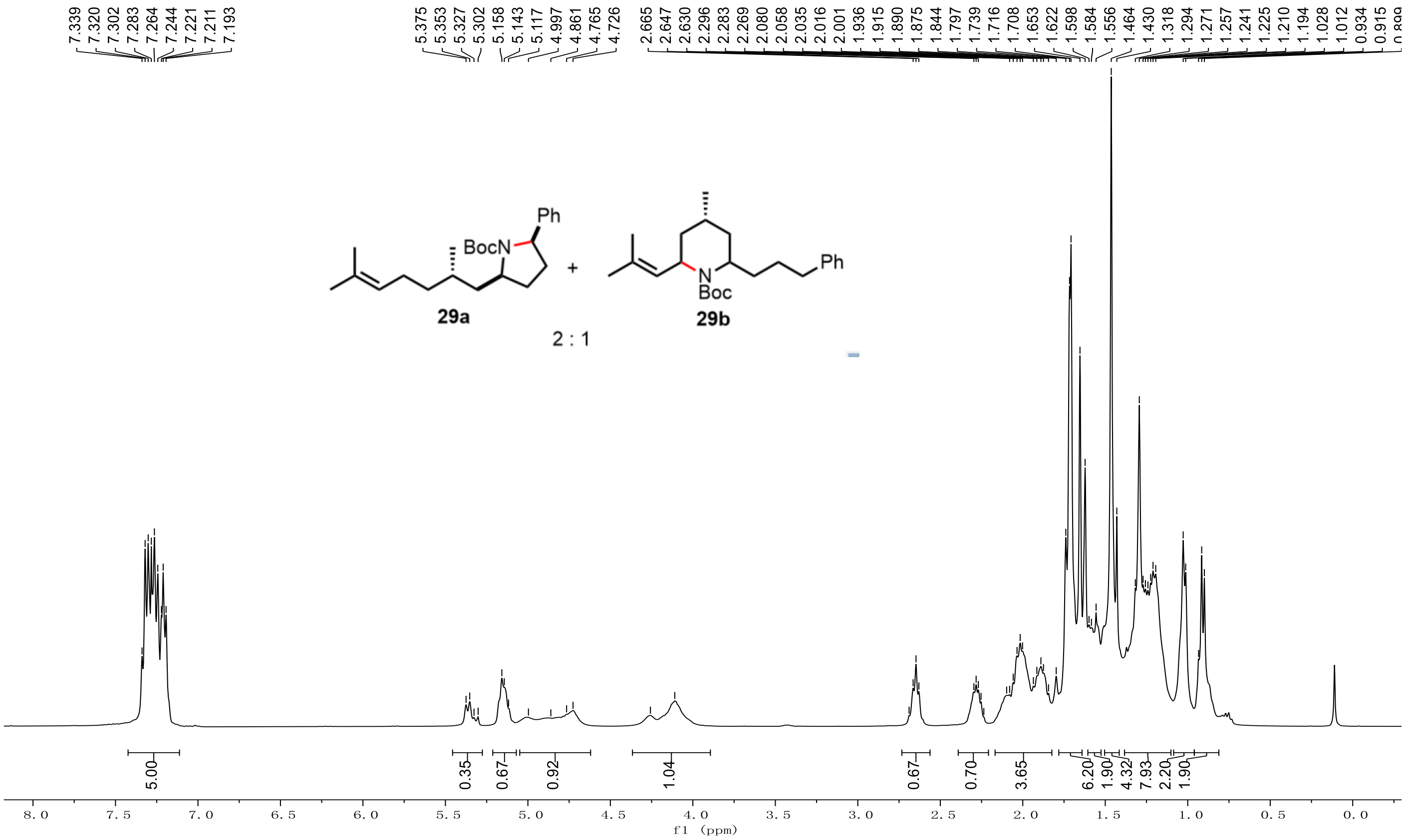

^{1}H NMR spectrum of **29** (400 MHz, $CDCl_3$)

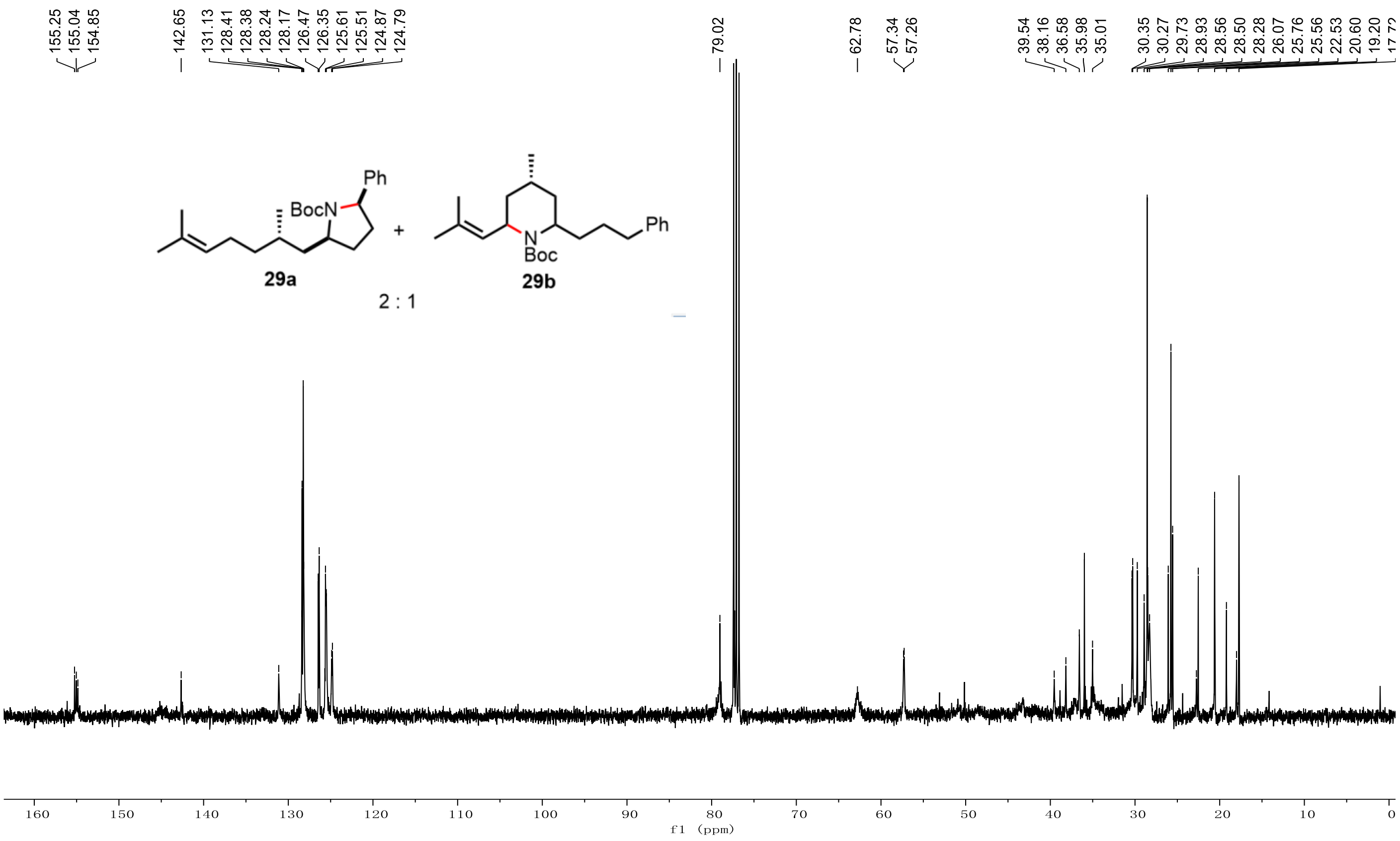

^{13}C NMR spectrum of **29** (100 MHz, $CDCl_3$)

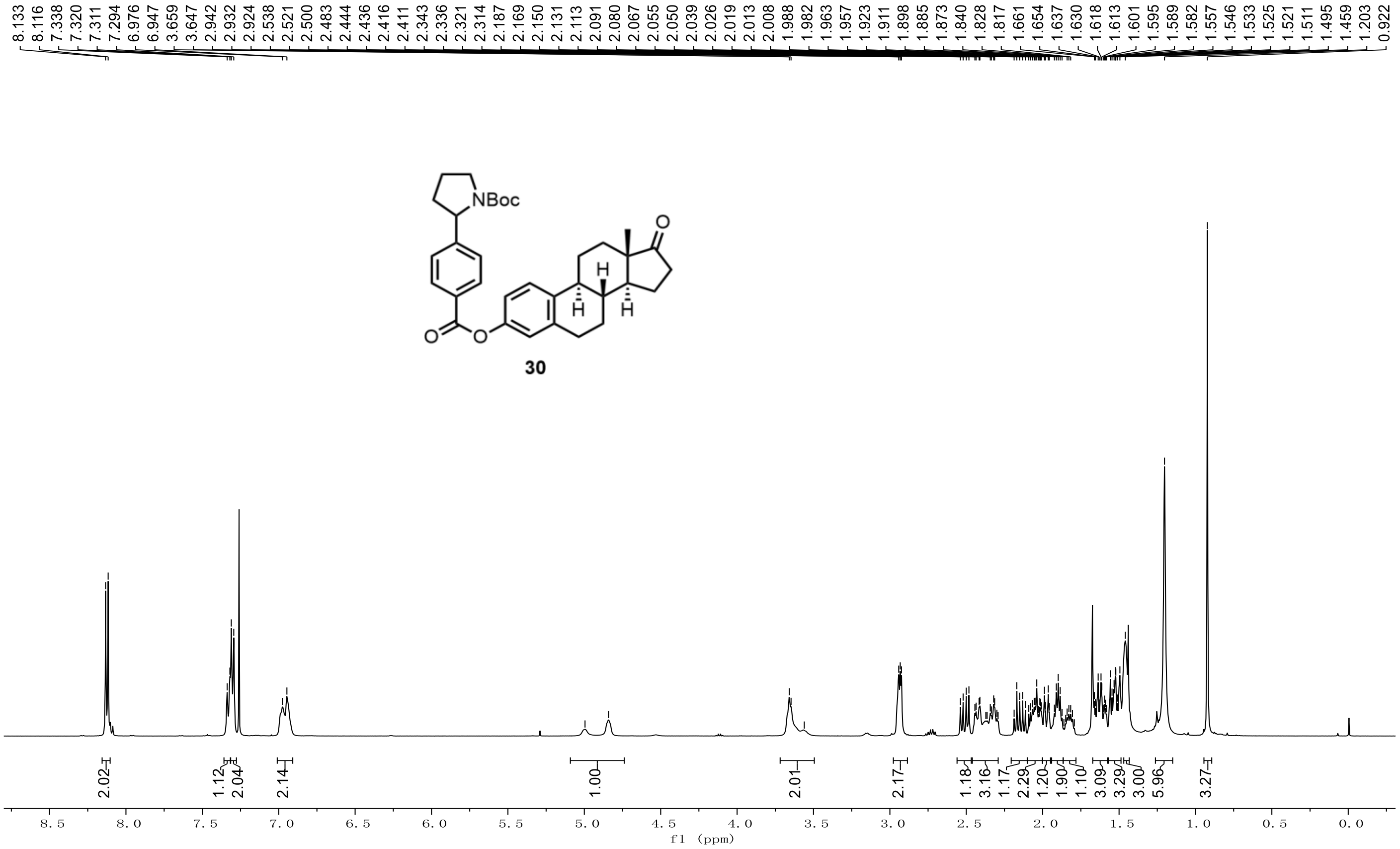

^{1}H NMR spectrum of **30** (500 MHz, $CDCl_3$)

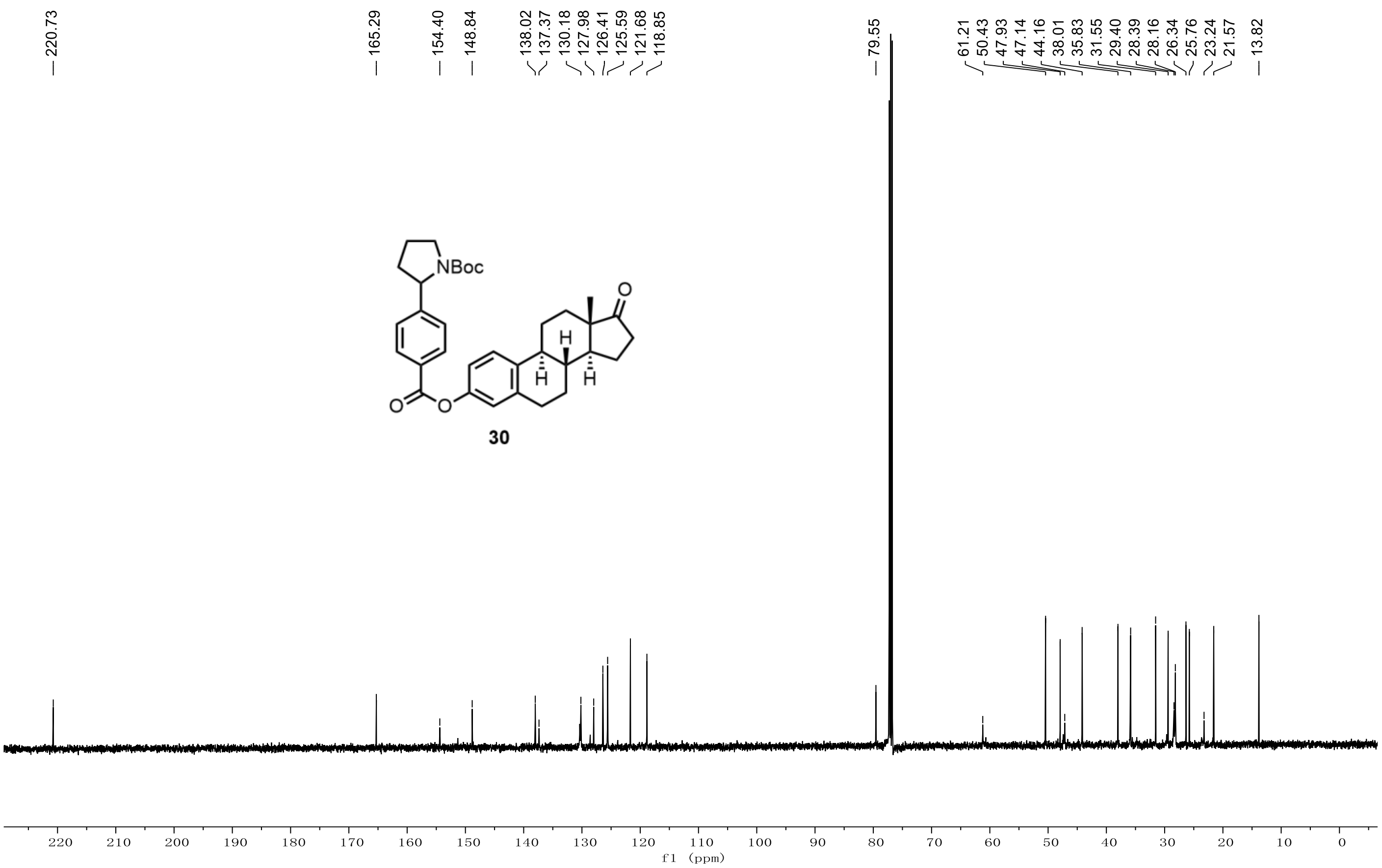

^{13}C NMR spectrum of **30** (125 MHz, $CDCl_3$)

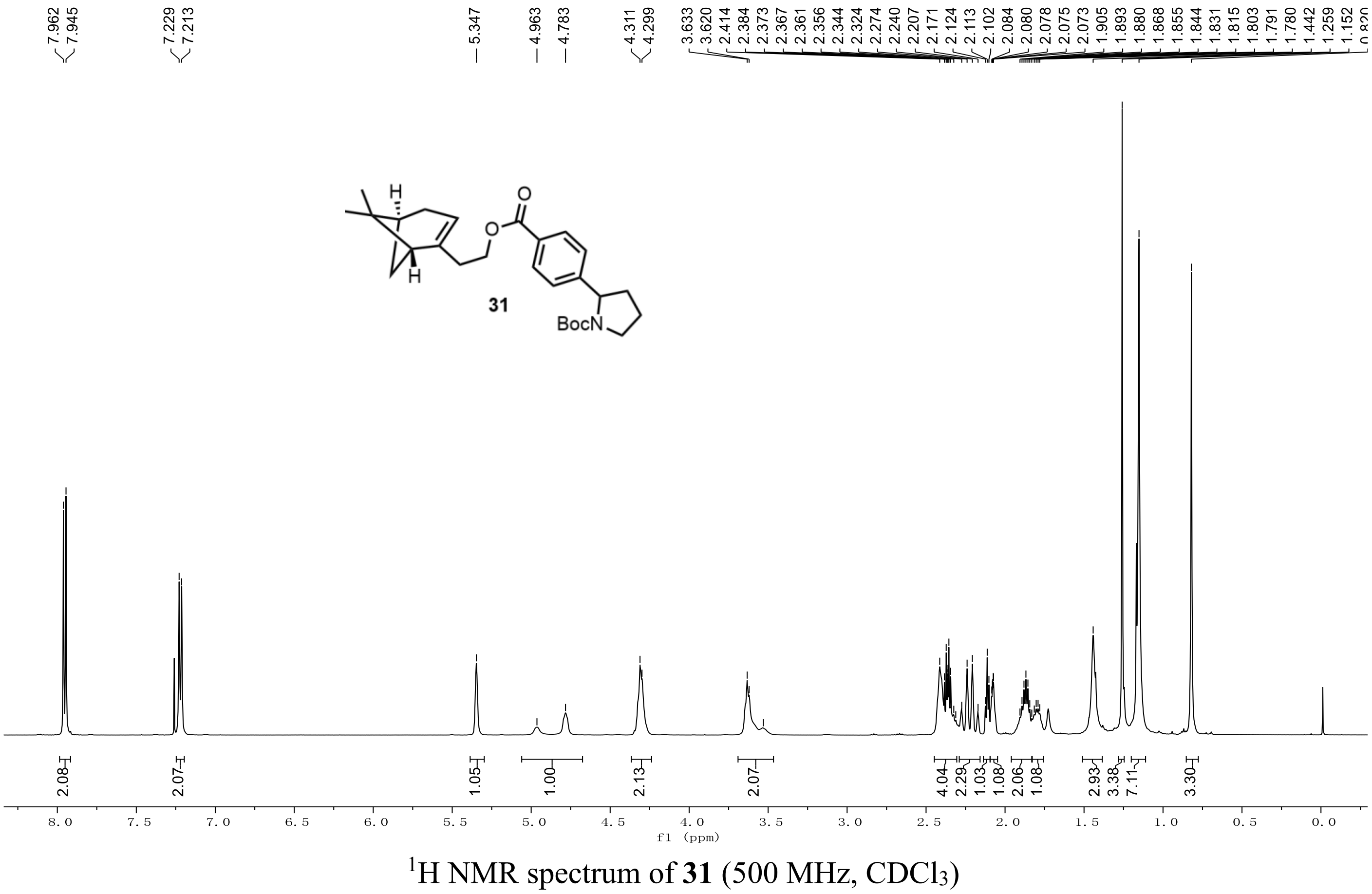

^{1}H NMR spectrum of **31** (500 MHz, $CDCl_3$)

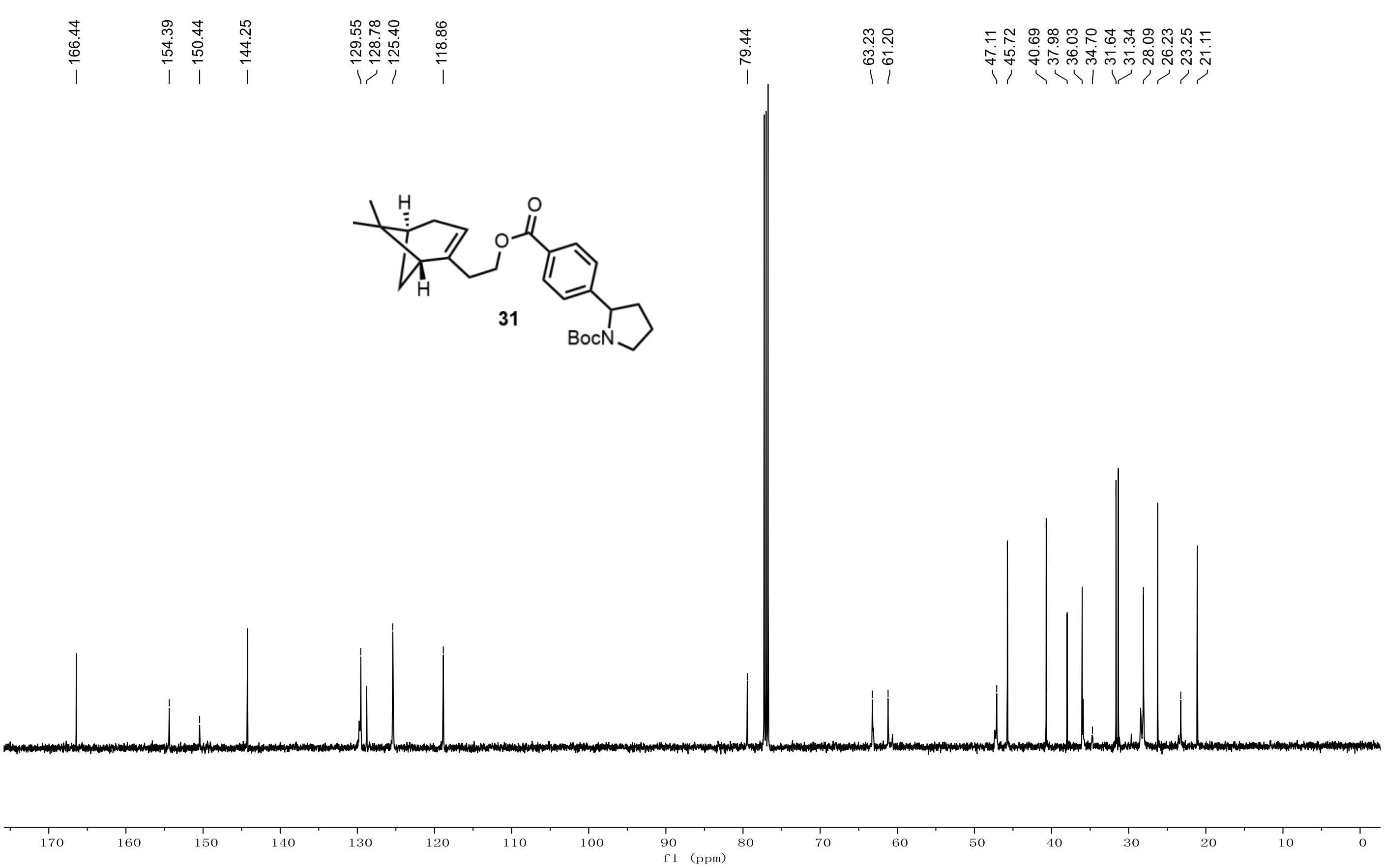

^{13}C NMR spectrum of **31** (125 MHz, $CDCl_3$)

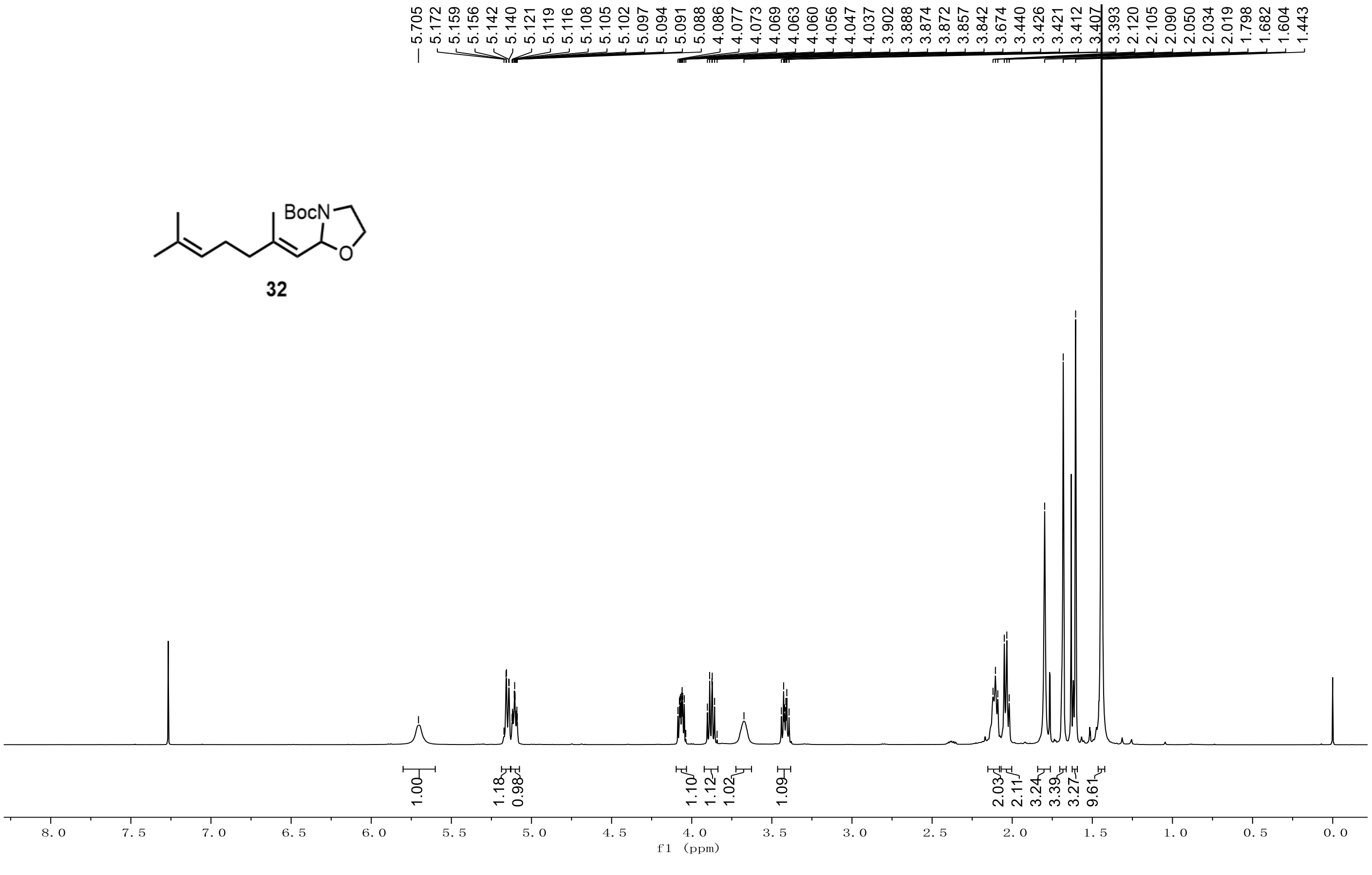

^{1}H NMR spectrum of **32** (500 MHz, $CDCl_3$)

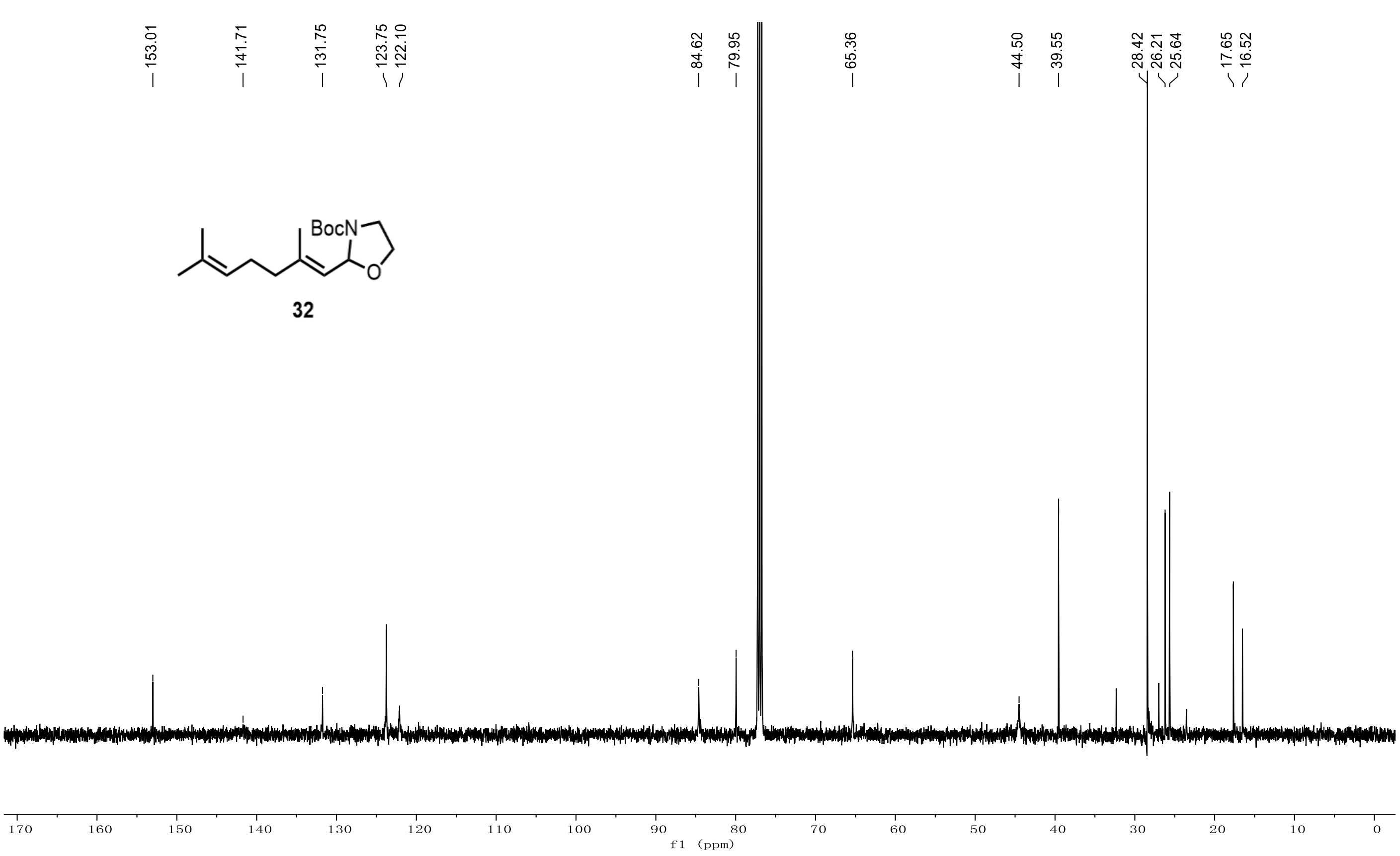

^{13}C NMR spectrum of **32** (125 MHz, $CDCl_3$)

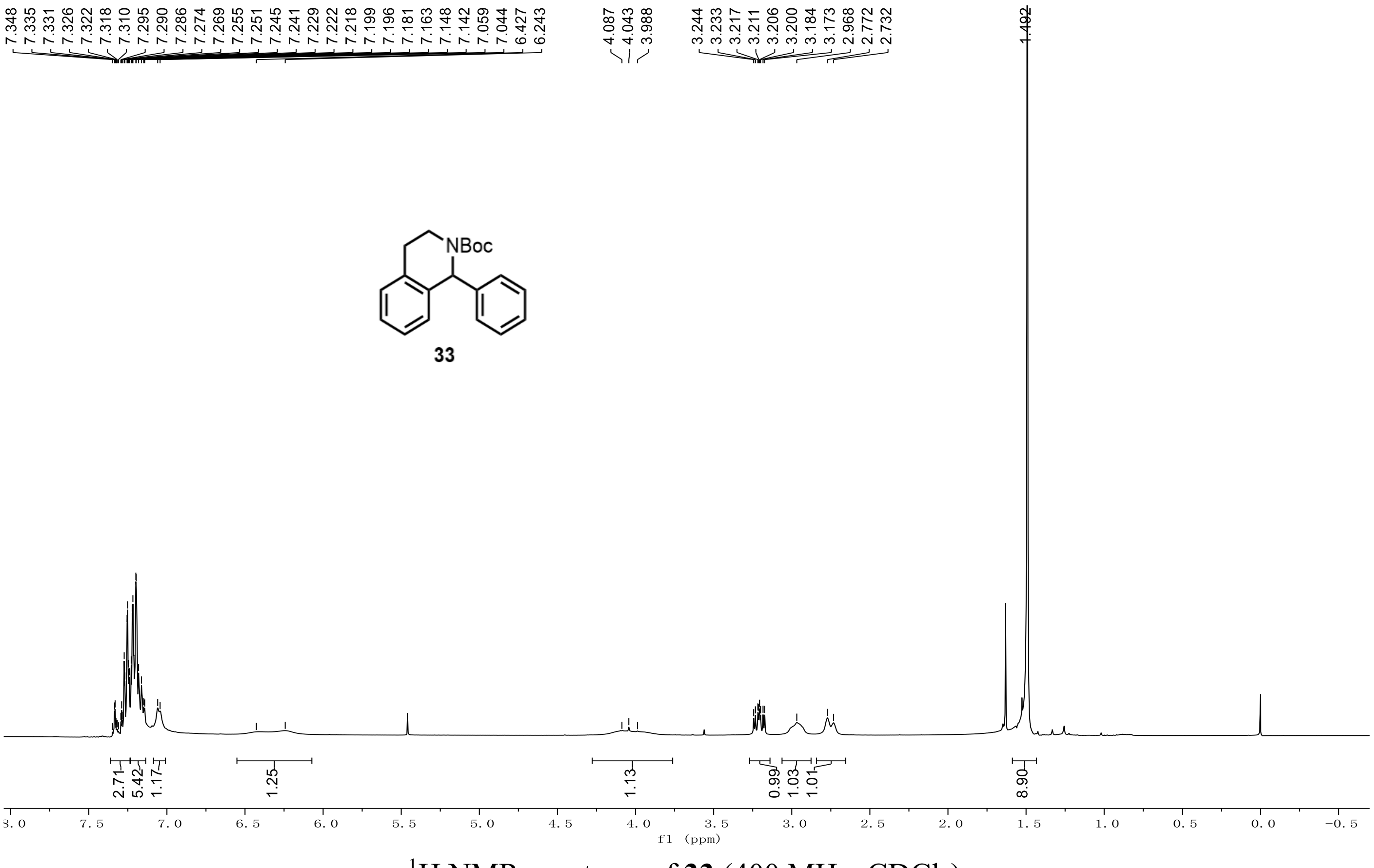

^{1}H NMR spectrum of **33** (400 MHz, $CDCl_3$)

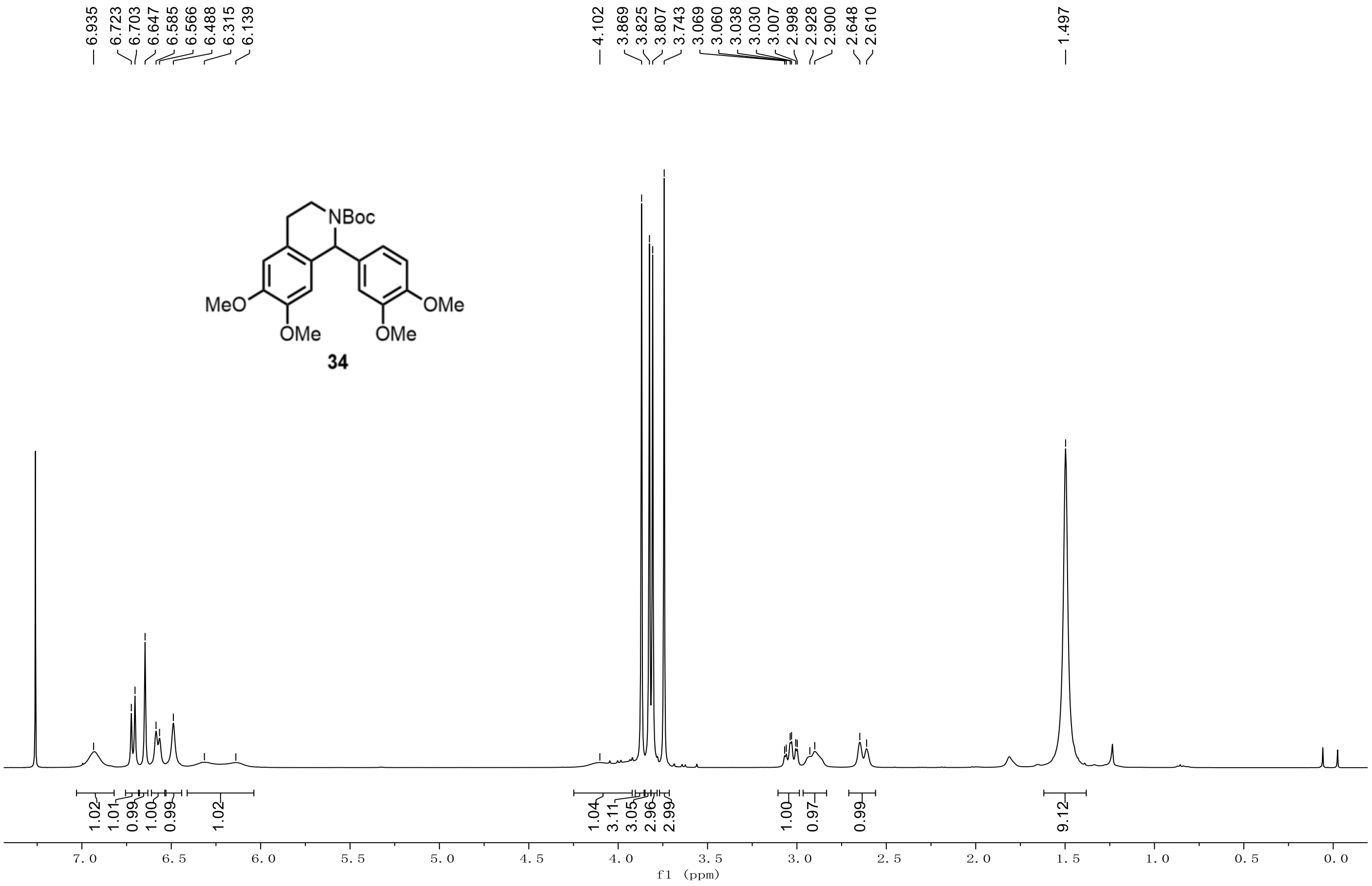

^{1}H NMR spectrum of **34** (400 MHz, $CDCl_3$)

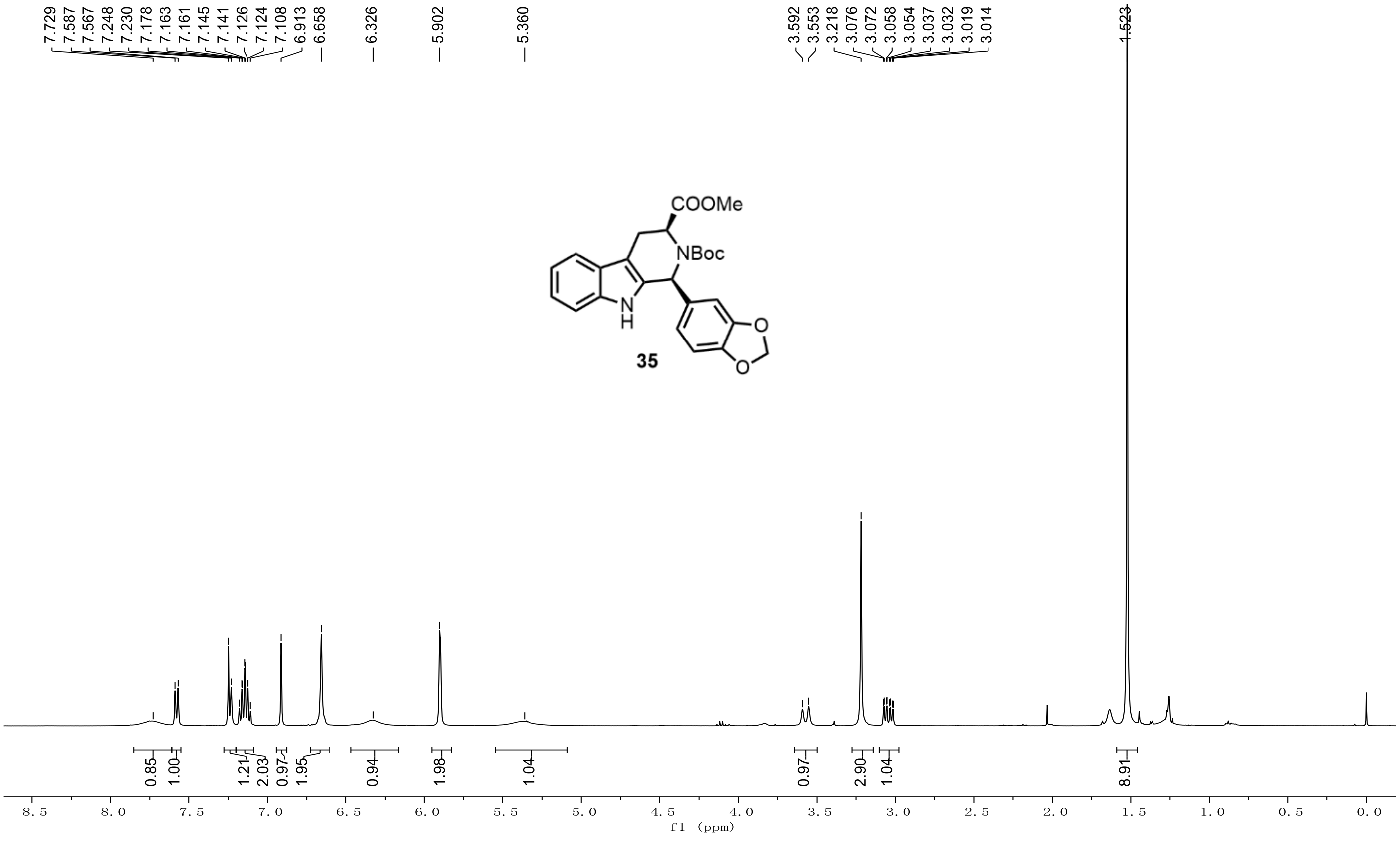

^{1}H NMR spectrum of **35** (400 MHz, $CDCl_3$)

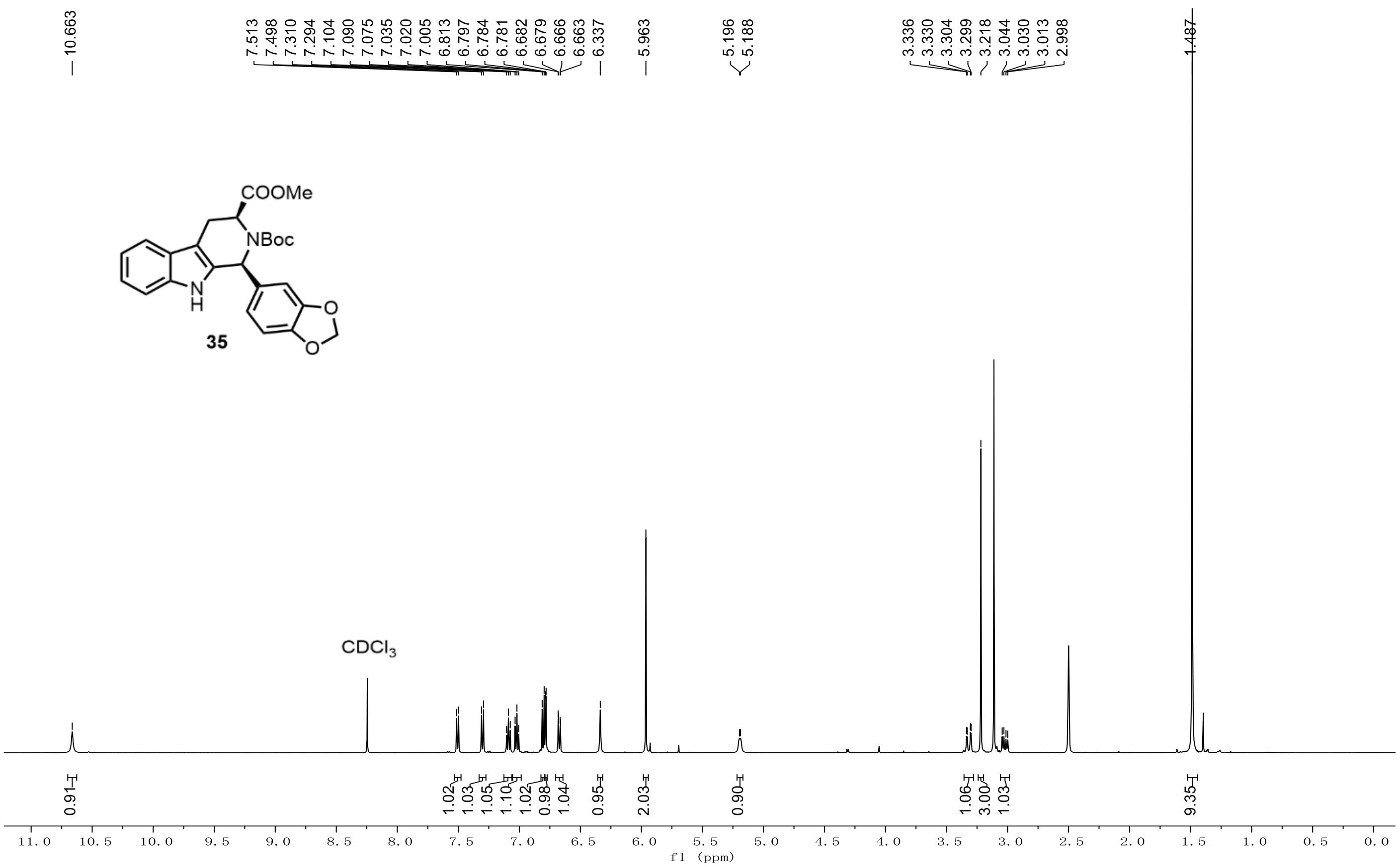

^{1}H NMR spectrum of **35** (500 MHz, DMSO-d_6, 343K)

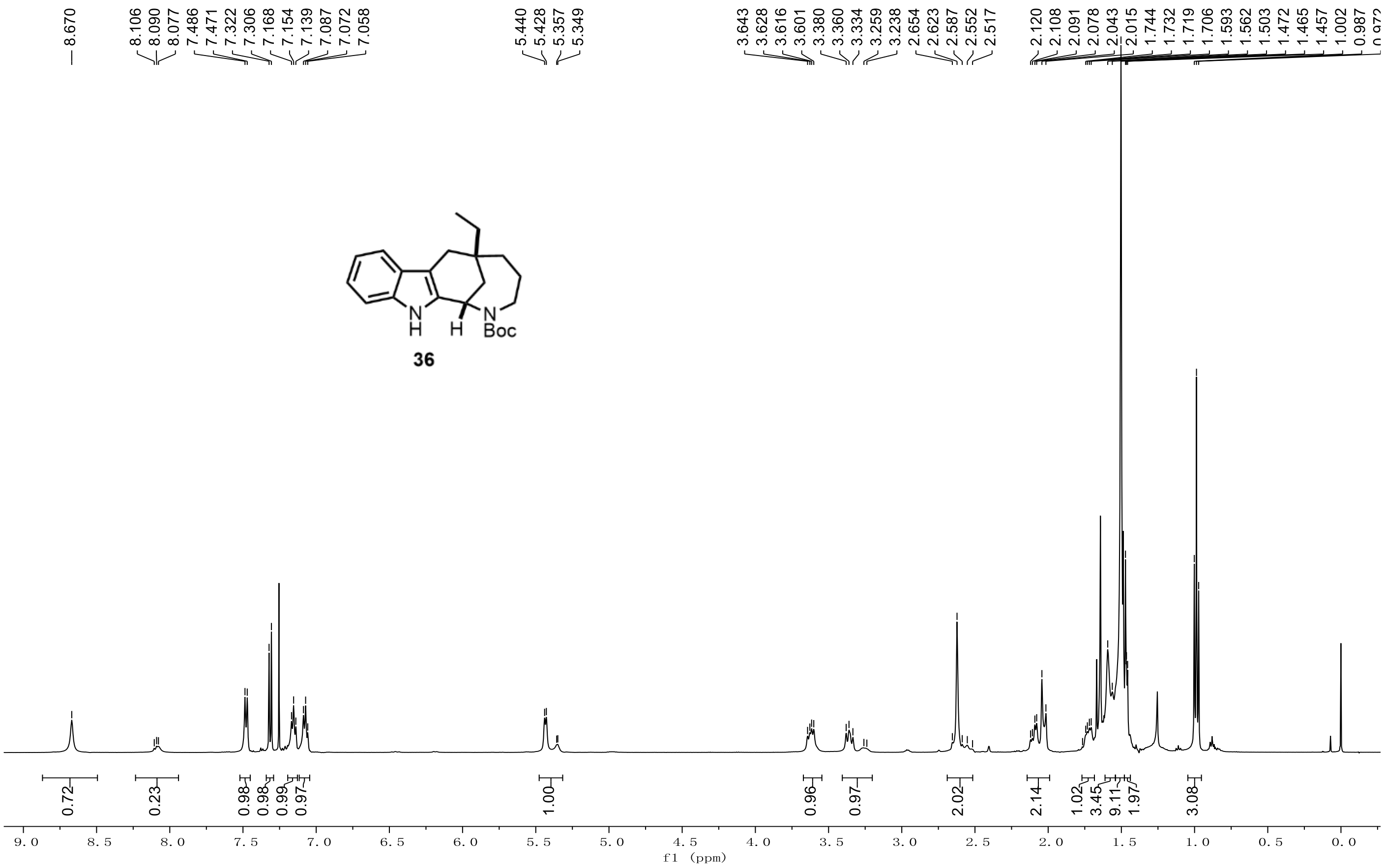

^{1}H NMR spectrum of **36** (500 MHz, $CDCl_3$)

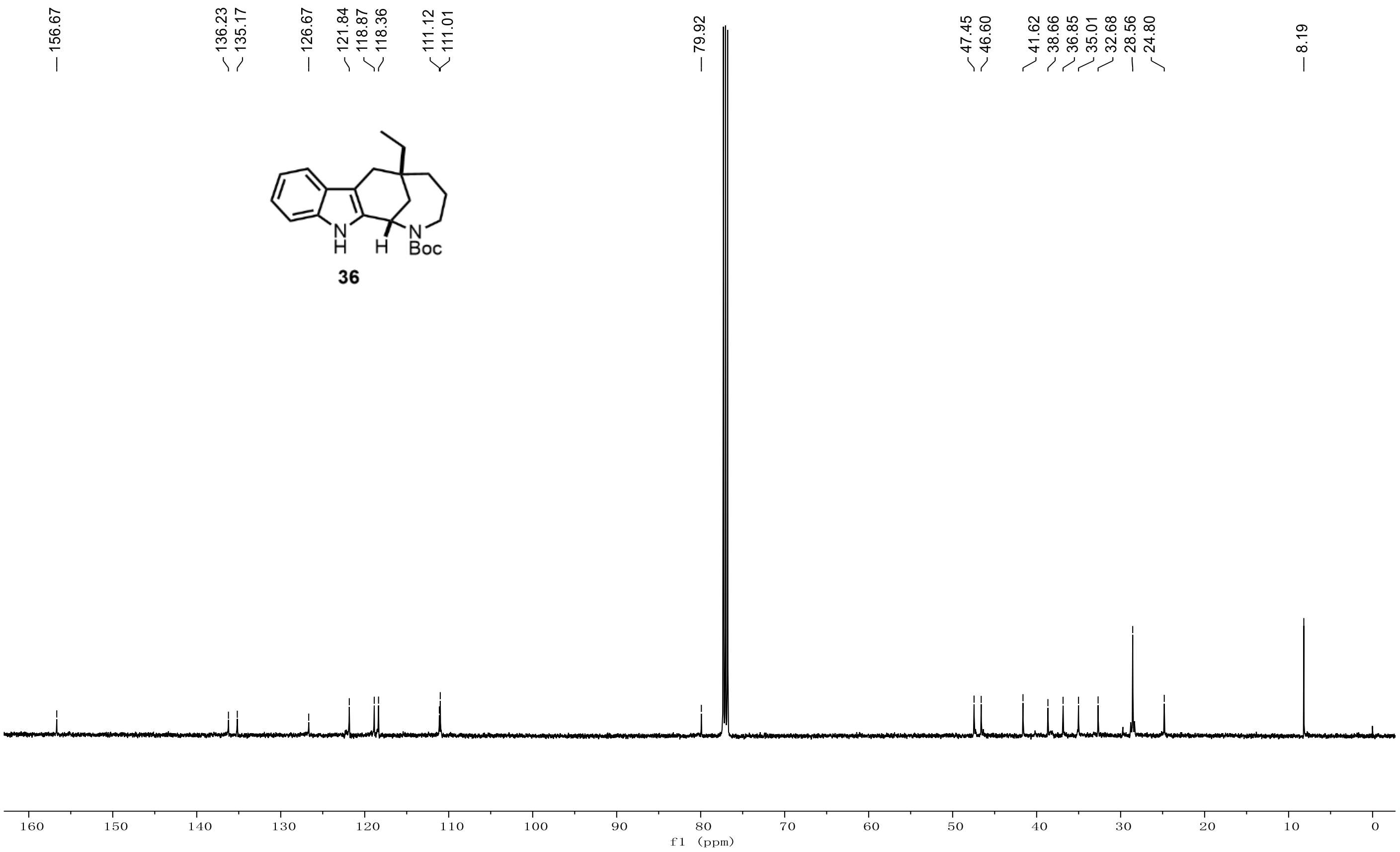

^{13}C NMR spectrum of **36** (125 MHz, $CDCl_3$)

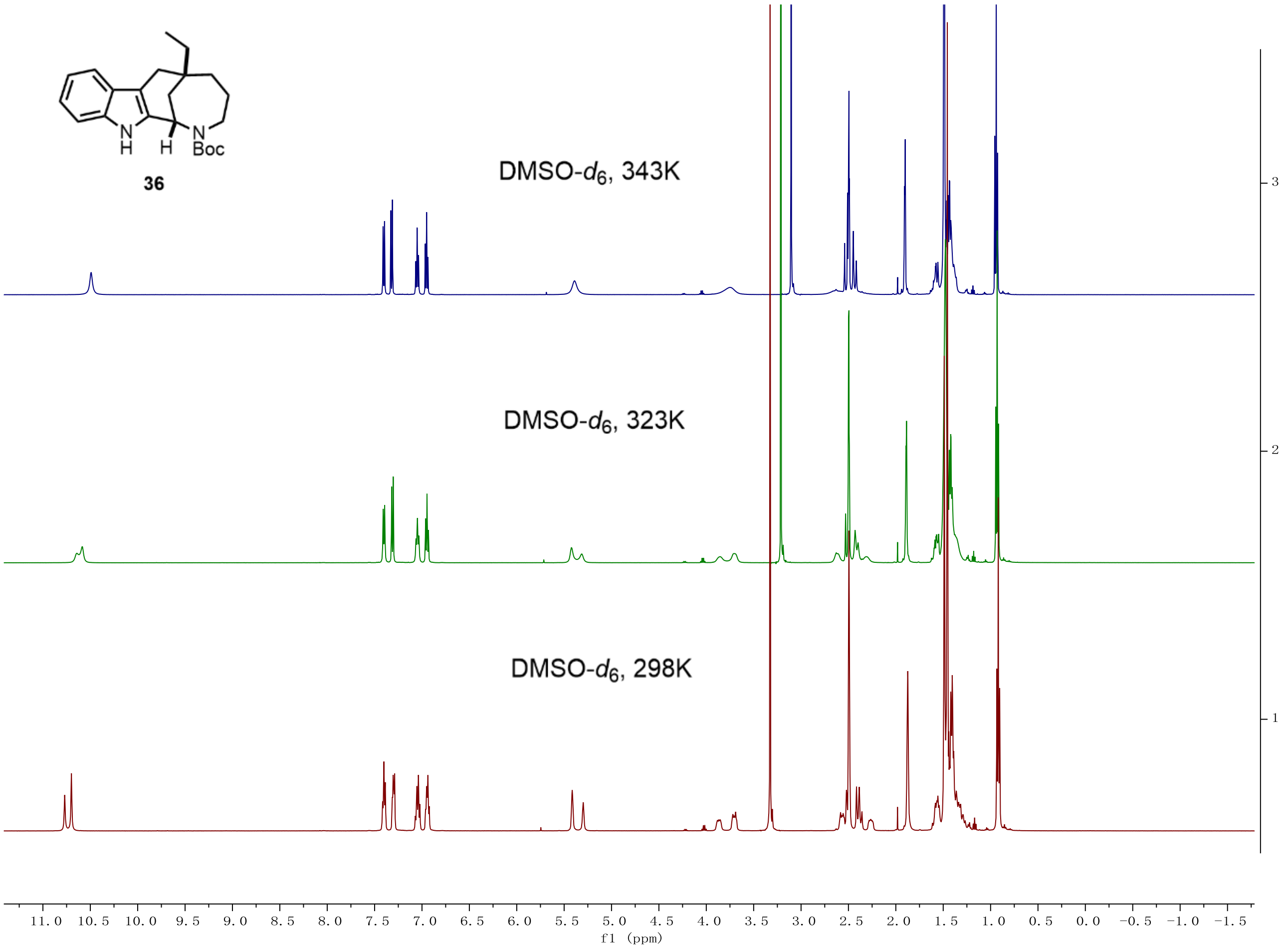

Variable-temperature ^{1}H NMR spectra of **36** (500 MHz, DMSO-d_6)

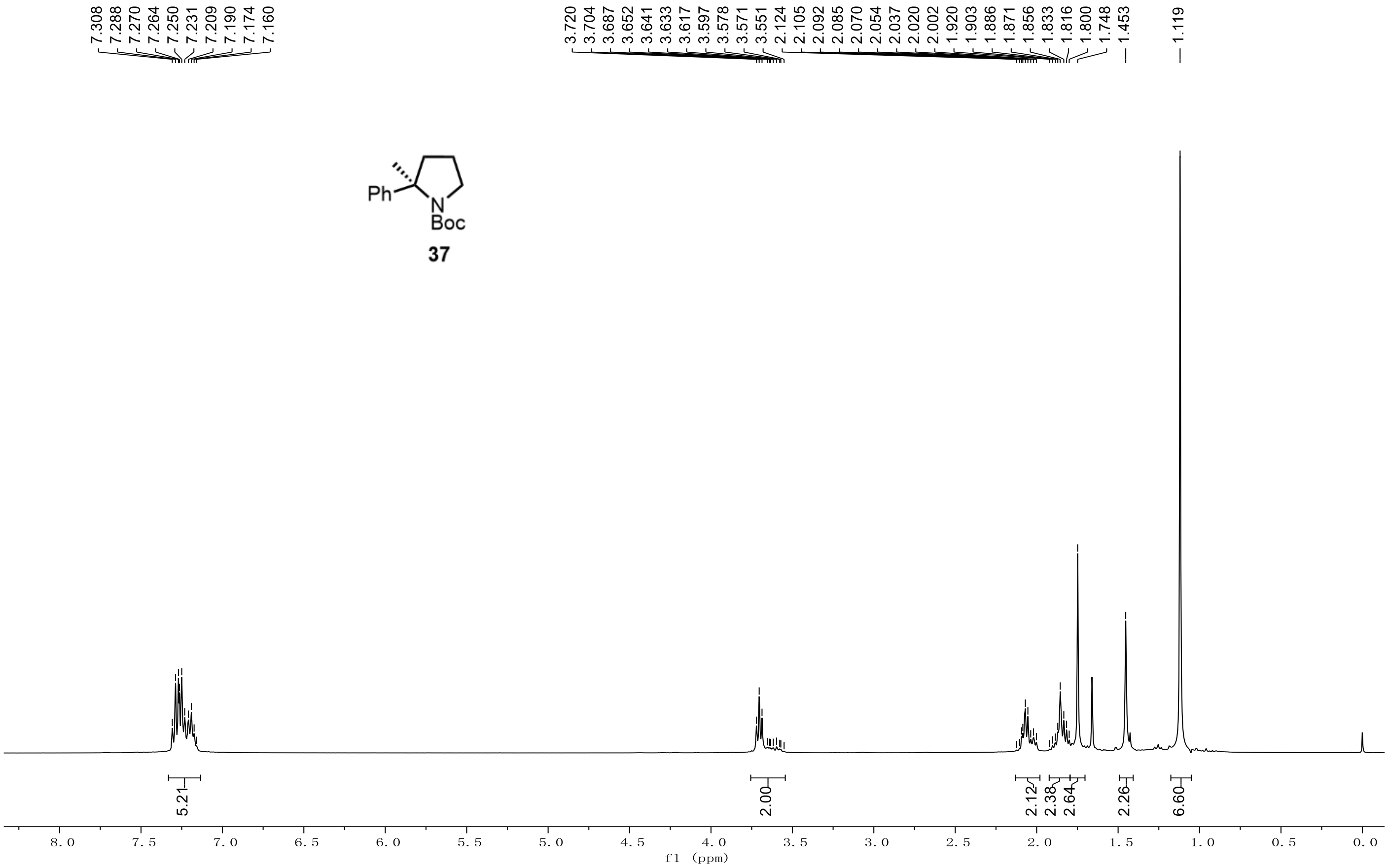

^{1}H NMR spectrum of **37** (400 MHz, $CDCl_3$)

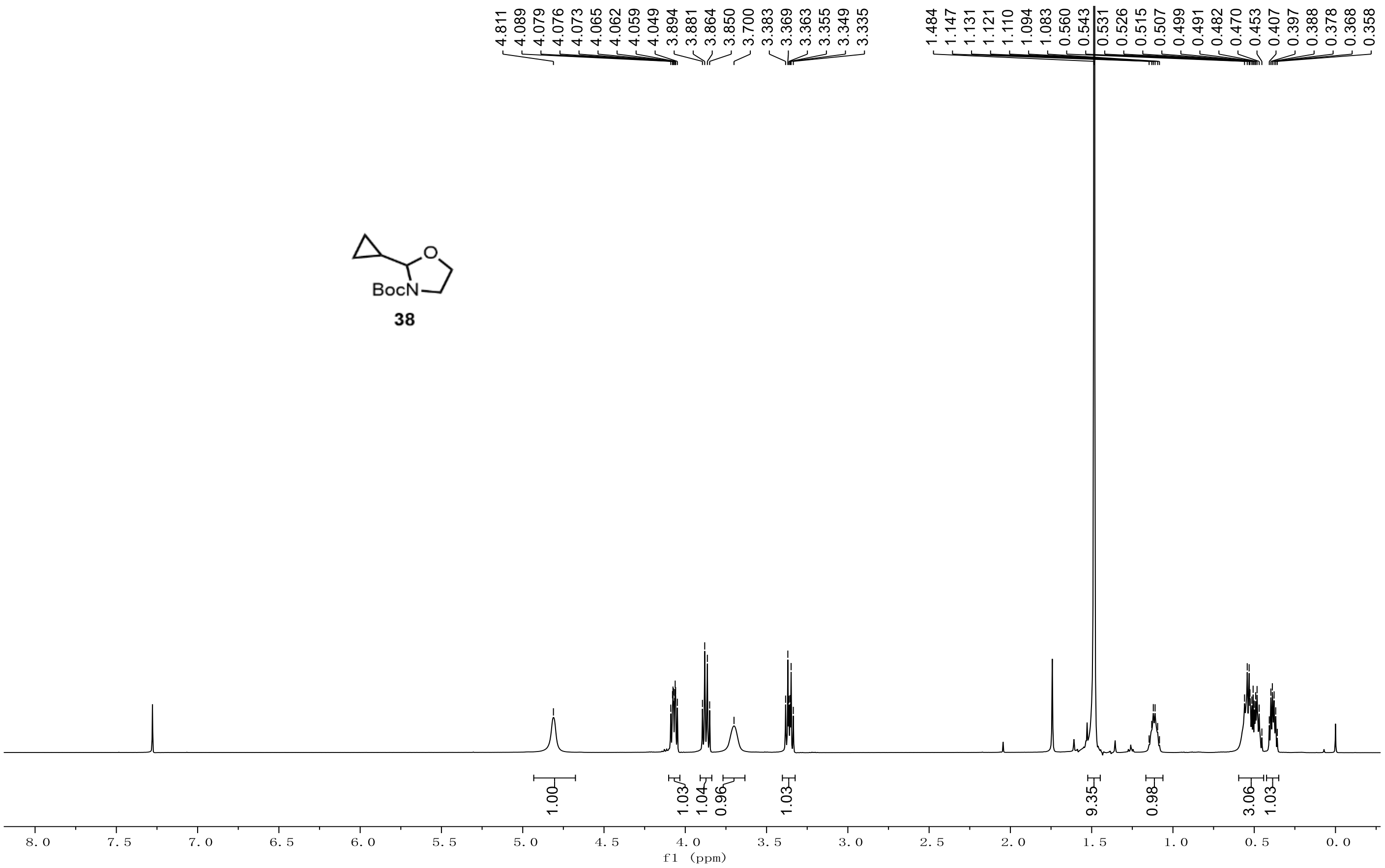

^{1}H NMR spectrum of **38** (500 MHz, $CDCl_3$)

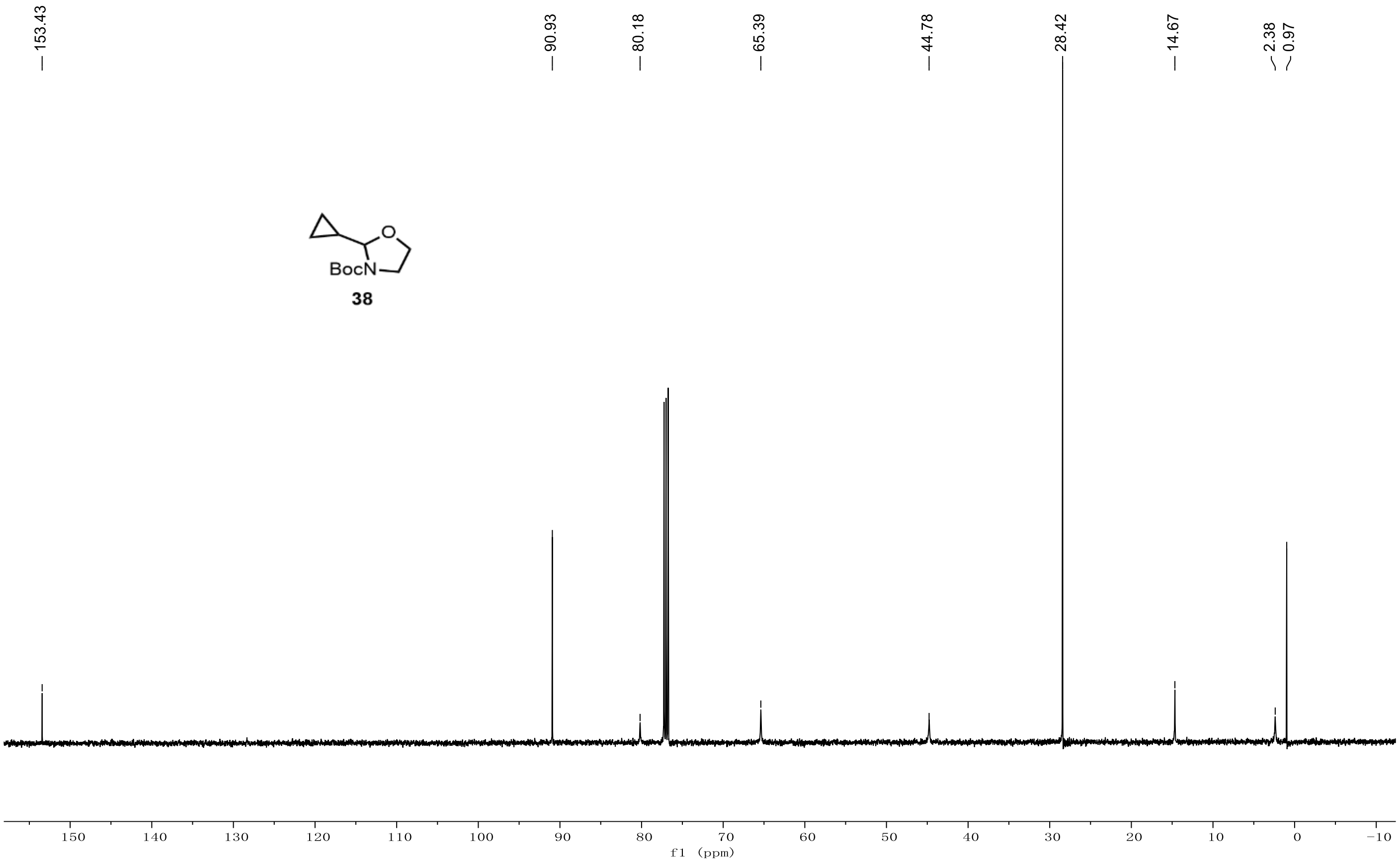

^{13}C NMR spectrum of **38** (125 MHz, $CDCl_3$)

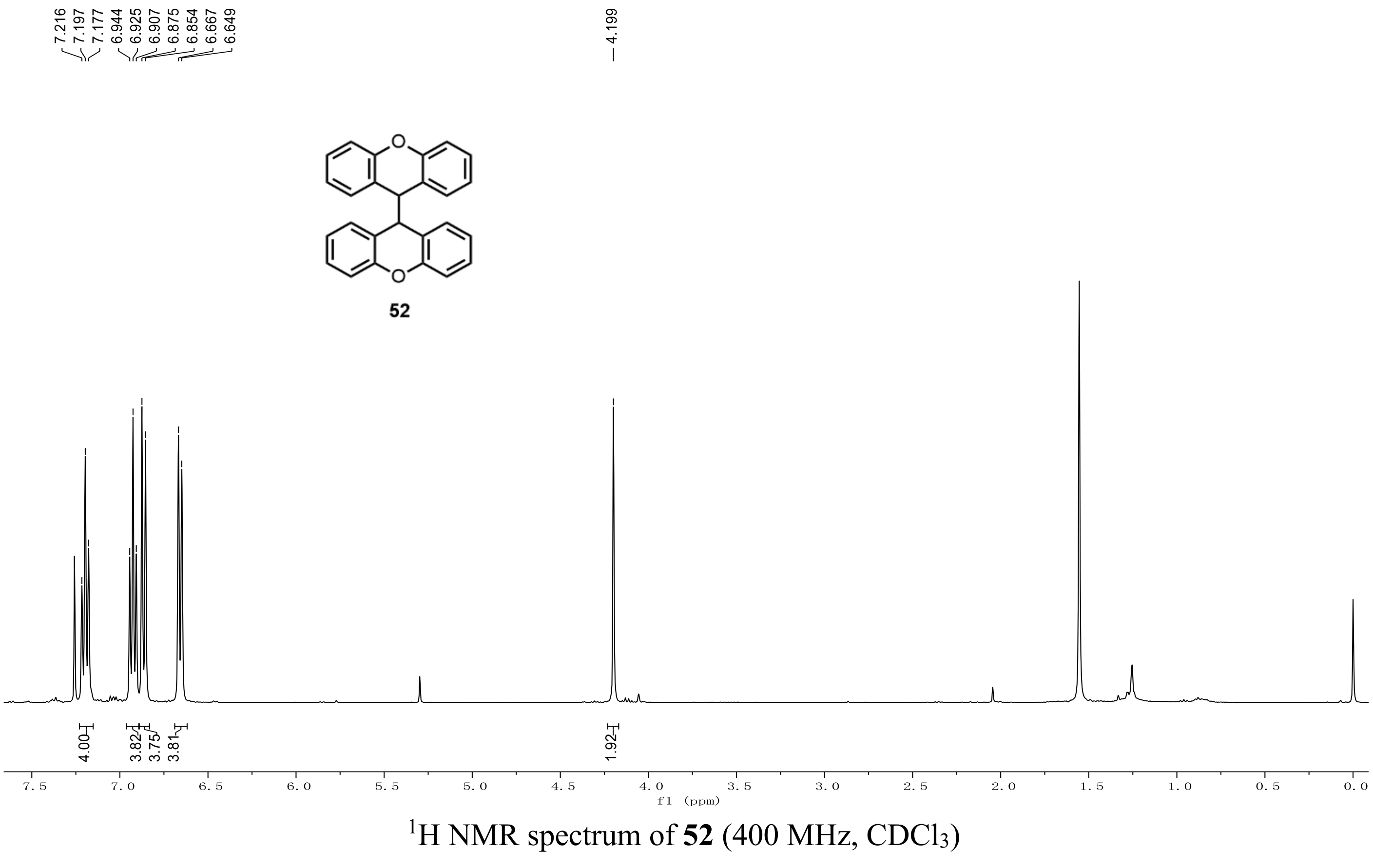

^{1}H NMR spectrum of **52** (400 MHz, $CDCl_3$)

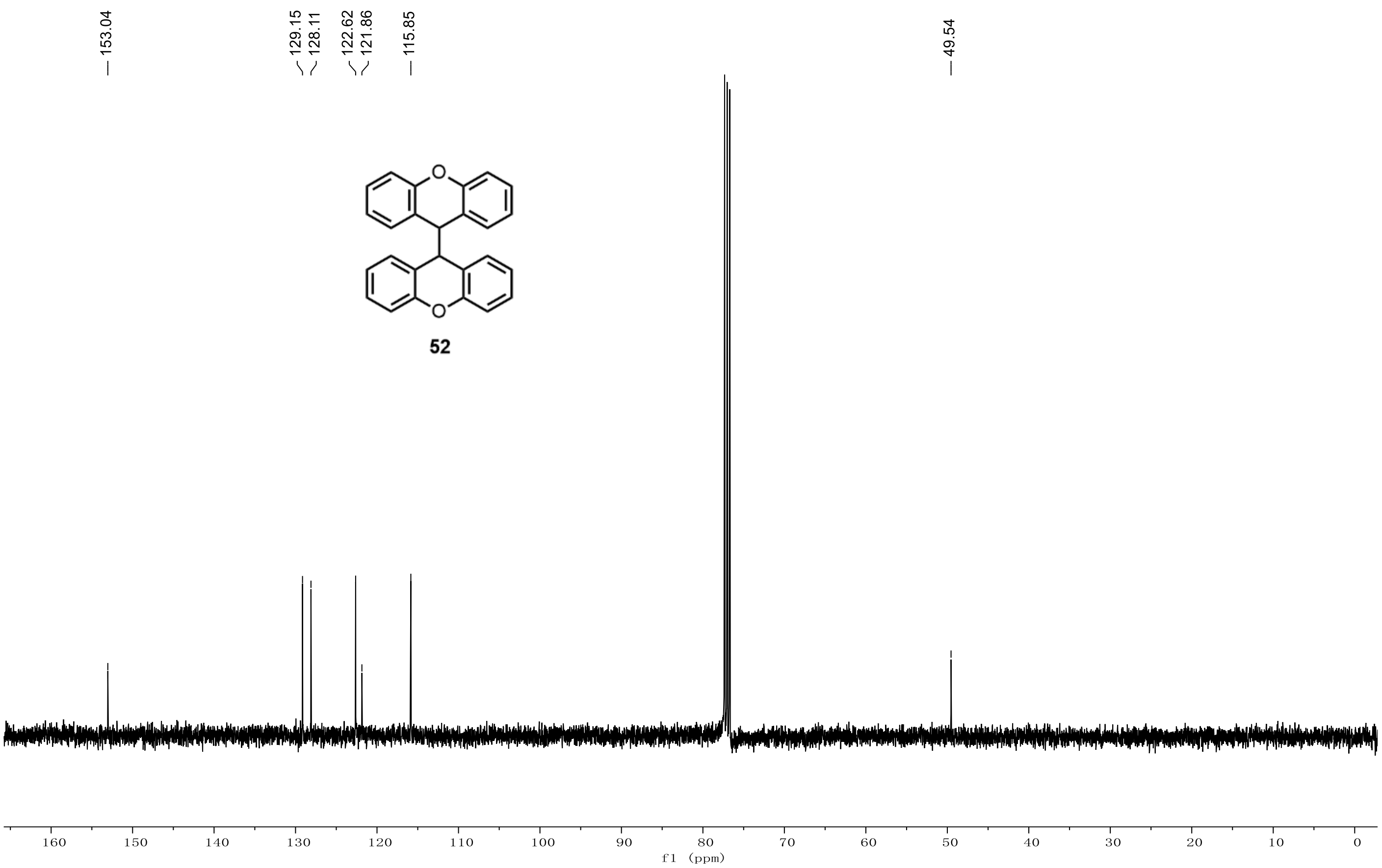

^{13}C NMR spectrum of **52** (100 MHz, $CDCl_3$)
